# Synthetic Receptors for the High‐Affinity Recognition of O‐GlcNAc Derivatives

**DOI:** 10.1002/anie.201510611

**Published:** 2016-01-28

**Authors:** Pablo Rios, Tom S. Carter, Tiddo J. Mooibroek, Matthew P. Crump, Micke Lisbjerg, Michael Pittelkow, Nitin T. Supekar, Geert‐Jan Boons, Anthony P. Davis

**Affiliations:** ^1^ School of ChemistryUniversity of BristolCantock's CloseBristolBS8 1TSUK; ^2^ Department of ChemistryUniversity of CopenhagenUniversitetsparken 52100Copenhagen ØDenmark; ^3^ Complex Carbohydrate Research CenterUniversity of Georgia315 Riverbend RoadAthensGA30602USA

**Keywords:** biomimetic hosts, carbohydrates, molecular recognition, receptors, supramolecular chemistry

## Abstract

The combination of a pyrenyl tetraamine with an isophthaloyl spacer has led to two new water‐soluble carbohydrate receptors (“synthetic lectins”). Both systems show outstanding affinities for derivatives of *N*‐acetylglucosamine (GlcNAc) in aqueous solution. One receptor binds the methyl glycoside GlcNAc‐β‐OMe with *K*_a_≈20 000 m ^−1^, whereas the other one binds an O‐GlcNAcylated peptide with *K*_a_≈70 000 m ^−1^. These values substantially exceed those usually measured for GlcNAc‐binding lectins. Slow exchange on the NMR timescale enabled structural determinations for several complexes. As expected, the carbohydrate units are sandwiched between the pyrenes, with the alkoxy and NHAc groups emerging at the sides. The high affinity of the GlcNAcyl–peptide complex can be explained by extra‐cavity interactions, raising the possibility of a family of complementary receptors for O‐GlcNAc in different contexts.

Binding carbohydrates in water is a notoriously difficult challenge.^1^ Saccharides are polar units that are both hydrophilic (therefore happy to remain in water) and hydromimetic (hard to distinguish from aqueous solvent). As a result, both natural and synthetic receptors tend to show low affinities, especially towards neutral carbohydrates. Lectins, the major class of carbohydrate‐binding proteins, often bind monosaccharides with *K*_a_<10^3^ m ^−1^.^2^ Biomimetic analogues (“synthetic lectins”) have shown *K*_a_ values of approximately 10^4^ m ^−1^ for charged substrates,^3^ but well‐characterized binding to neutral monosaccharides in water is generally much weaker.1b–1g

We have previously described synthetic lectins that bind all‐equatorial monosaccharides (glucose and close relatives) with affinities from approximately 10 to 600 m ^−1^.3a, 4 A particular target has been the β‐*N*‐acetylglucosaminyl (β‐GlcNAc or O‐GlcNAc) unit **1**. This moiety is a dynamic post‐translational modification of proteins that is involved in many cellular processes and has been linked to major diseases such as diabetes and Alzheimer's disease.^5^ There is much interest in agents that bind O‐GlcNAc for use in detection and separation methods for modified proteins.^6^ A few years ago, we reported that the tricyclic receptor **4** (Figure 1) binds the simplest O‐GlcNAc model **2** with *K*_a_=630 m ^−1^, and the O‐GlcNAcylated peptide **3** with *K*_a_=1000 m ^−1^.4e These affinities were encouraging and competitive with the GlcNAc‐binding lectin wheat germ agglutinin (WGA), but probably insufficient for most biological applications. We now describe two new systems, which show strongly increased binding to **2** and **3**. These new synthetic lectins are accessible in just eight synthetic steps and may point to a practical solution to O‐GlcNAc recognition in glycobiology.
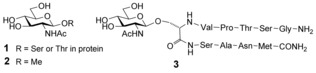



**Figure 1:**
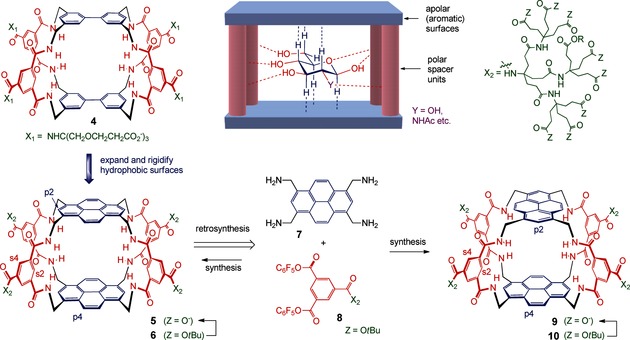
Design and synthetic strategy. Top middle: Cartoon showing interactions in “temple” synthetic lectins for all‐equatorial carbohydrates. Anticlockwise from the top left: The successful design **4** can be improved by converting the biphenyls into pyrenes, giving receptor **5**. Receptor **5** can be deconstructed into **7** and **8**, but in the forward direction, this combination leads to both **5** and **9**. The external groups X_1_ (in **4**) were expanded to X_2_ (in **5** and **9**) to ensure water solubility.

The conceptual basis for this work is summarized in Figure 1. Synthetic lectin **4** is composed of parallel aromatic units (blue) held apart by polar spacers (red). The former can experience hydrophobic/CH–π interactions with axial CH groups in all‐equatorial substrates while the spacers can hydrogen‐bond to equatorial substituents. The cartoon used to describe this arrangement (Figure 1, top) inspired the “temple” description for this family of molecules.1f In **4**, the hydrophobic surfaces are provided by biphenyls, which are readily introduced and provide adequate CH–π interactions. However, biphenyls are prone to twisting, which disrupts contact with axial CH groups, while condensed aromatic compounds are planar.4a,4b A 1,3,6,8‐tetrasubstituted pyrene is geometrically equivalent to the biphenyls in **4**, so tricyclic cage **5** was identified as a promising design. Based on recent work,3a, 4a receptor **5** was provided with more powerful water‐solubilizing groups than **4**; pyrenes are strongly inclined to self‐associate in water, and this needed to be countered for reliable binding characterization.

Whereas the design of **5** might seem obvious, the synthesis appeared problematic. Retrosynthesis leads to tetraamine **7** and an isophthaloyl derivative such as **8**, but in the forward direction this combination could lead to a variety of products including a second cage **9** (see Figure 1). It was not clear whether **5** and **9** could be formed in good yield, then separated, and distinguished from each other. Nonetheless, in the absence of an alternative, we chose to make an attempt. As shown in Scheme 1, tetraamine **7** was prepared as its hydrochloride salt in 42 % overall yield by a five‐step procedure from pyrene **11**.^7^ Tetraamine **7** was insoluble in organic solvents, but the hydrochloride **7**⋅4 HCl could be dissolved in THF/water (5:1). This medium was used to convert **7** into **12** and thence to a mixture of the cages **6** and **10**, which were separable by HPLC. As anticipated, the NMR spectra of the two cages were almost identical. However, a NOESY spectrum of a 1:1 mixture showed a difference between the pyrene p2/p4 cross peaks for the two isomers (for numbering, see Figure 1 and also the Supporting Information, Scheme S1). For the more quickly eluted isomer, this cross peak was significantly larger, as expected for “staggered” cage **10**, and provisional assignments were made on this basis.^7^

**Scheme 1:**
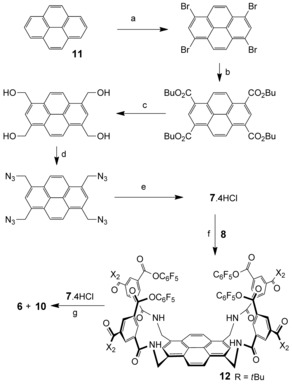
Synthesis of the protected receptors **6** and **10**. Reaction conditions: a) Br_2_, PhNO_2_, 96 %; b) BuOH, CO (30 bar), DIPEA, Pd(OAc)_2_, BINAP, xylenes, 89 %; c) LiBH_4_, THF, MeOH, ca. 100 %; d) (PhO)_2_PON_3_, DMF, DBU, 52 %; e) Ph_3_P, THF, H_2_O; then HCl (aq.), 93 %; f) DIPEA, H_2_O, THF, 47 %; g) DIPEA, H_2_O, THF, 57 %.

Samples of **6** and **10** were treated with TFA, dried, and dissolved in NaOD/D_2_O (pH 7) to give solutions of **5** and **9** for NMR analysis.^7^ ^1^H NMR spectra of eclipsed receptor **5** appeared broadened at 25 °C and unexpectedly complex, implying a less symmetric structure than the *D*_2*h*_ cage. The spectra were unchanged between 16 and 500 μm, suggesting that **5** is monomeric within this concentration range. Diffusion‐ordered NMR spectroscopy (DOSY) implied a hydrodynamic diameter of 2.1–3.8 nm, which is also consistent with monomeric **5**. Upon warming to 80 °C the spectra sharpened and simplified, becoming consistent with the *D*_2*h*_ structure. We concluded that the room‐temperature spectra are affected by slow conformational exchange, with a ground‐state conformation of low symmetry. The behavior of **9** was similar, except that the spectra at 25 °C showed minor concentration dependence. However, any aggregation appeared to be limited as DOSY was again consistent with the presence of a monomeric receptor (hydrodynamic diameter: 2.6–3.5 nm).

Both **5** and **9** were studied as carbohydrate receptors by ^1^H NMR titrations in D_2_O.^7^ In most cases, the host spectra showed significant changes upon carbohydrate addition. For many substrates, the spectra implied complex formation at fast or intermediate rates on the ^1^H NMR timescale. Given the complexity of the host spectra, the changes were difficult to interpret, and no attempt was made to quantify binding in these cases. However, for some substrates, a new set of signals appeared, implying binding with slow exchange on the NMR timescale. In these cases, integration of the new signals could be used with confidence to follow the concentrations of the complexes. The data could be fitted to a 1:1 binding model to give the binding constants listed in Table 1.^7^, ^8^ Supporting values were obtained by isothermal titration calorimetry (ITC).^7^ The NMR spectra, binding analysis curves, and ITC data for staggered receptor **9** and GlcNAc derivative **2** are shown in Figure 2. A few combinations that were quantified solely by ITC are also included in Table 1.

**Figure 2:**
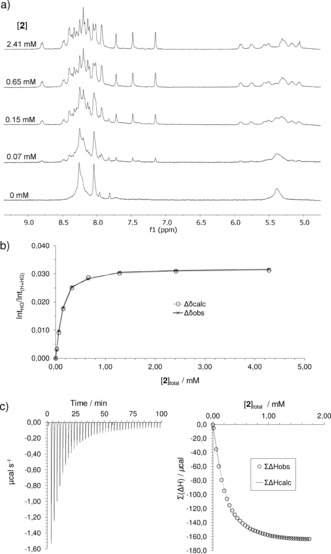
Binding studies of receptor **9** with GlcNAc‐β‐OMe (**2**). a) Partial spectra from the ^1^H NMR titration in D_2_O. Signals due to **9** were replaced by new ones assigned to the complex. b) Binding curve based on integrating the signal at 8.8 ppm versus those in the region of 7–9.5 ppm. *K*_a_=18 200 m ^−1^. c) ITC output and binding analysis plot for a titration in water. *K*_a_=16 600 m ^−1^.

**Table 1:** Association constants (*K*_a_) for 1:1 complexes of the receptors **5** and **9** with carbohydrates in water, as determined by ^1^H NMR spectroscopy and ITC. Data for **4** are listed for comparison.^[a]^

Carbohydrate	*K*_a_ [m ^−1^] determined by NMR (ITC)		
	**4** ^[b]^	**5**	**9**
GlcNAc‐β‐OMe (**2**)	630	2100 (2200)	18200 (16600)
GlcNAc‐α‐OMe (**13**)	24^[c]^	–^[d]^	1550 (1520)
GlcNAc (**14**)	56	–^[d]^	520 (520)
methyl β‐d‐glucoside (**15**)	28	– (1440)	1180 (1230)
d‐glucose (**16**)	9	– (120)	– (190)

[a] *T*=298 K, errors estimated from curve fitting: ≤5 %.^7^ NMR experiments were performed in D_2_O and ITC titrations in H_2_O. Values for reducing sugars are weighted averages of those for the two anomers, as discussed in Ref. 4c. [b] See Ref. 4e. [c] Measured by induced circular dichroism. [d] Could not be determined accurately. 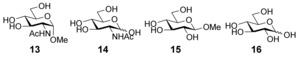

As shown in Table 1, the binding studies with **5** and **9** yielded some exceptionally high affinities. As expected, the eclipsed cage **5** (our initial target) was found to be a good receptor for the simple O‐GlcNAc model compound **2**, showing a three‐fold increase in binding power over biphenyl‐based prototype **4**. However, the staggered cage **9** proved even more effective. Receptor **9** bound O‐GlcNAc model compound **2** with a *K*_a_ value of 18 200 m ^−1^, which is 30 times larger than that of **4** and 25 times larger than that of the natural lectin WGA (*K*_a_=730 m ^−1^).4e, 9 To the best of our knowledge, this is the highest affinity that has been measured for a biomimetic receptor binding a small, electrically neutral monosaccharide derivative in water. Receptor **9** also showed good affinities for GlcNAc **14** (10 times higher than **4**)4e and methyl β‐d‐glucoside (**15**; 5 times higher than the previous record).4a Surprisingly, α‐GlcNAc derivative **13**, with an axial OMe group, was also bound fairly strongly. The selectivity of **5** and **9** against other common monosaccharides was difficult to assess, as affinities for the latter could not be measured by NMR spectroscopy (see above). However, ITC measurements on **5** and **9** with mannose, galactose, *N*‐acetylgalactosamine, and *N*‐acetylmannosamine gave low thermal output, suggesting weak binding at best.^10^

As illustrated in Figure 2 a, binding of carbohydrates by **5** and **9** with slow exchange on the NMR timescale yields complex spectra owing to desymmetrization of the receptors by the asymmetric substrates. We have previously shown for **4**+**2** that such spectra can be exploited to obtain detailed 3D structures.4e Here, we were interested both in determining the mode of binding and confirming the identities of the receptors. Desymmetrization of the receptor cores would allow connections to be followed within the macropolycyclic frameworks, distinguishing between the two possibilities. Complexes **5**⋅**2**, **9**⋅**2**, and **9**⋅**13** were subjected to detailed NMR analysis by NOESY, TOCSY, and COSY.^7^ In all cases, full assignments could be obtained for the carbohydrate and receptor core protons. The structures of **5** and **9** were indeed confirmed unambiguously. In the former case, the p4 and s2 signals (see Figure 1) formed two discrete networks, whereas in the latter case, all of these signals were connected in a single sequence. Integrated NOESY signals were employed to build models of all three complexes. For each structure, >70 cross peaks were employed to obtain inter‐ and intramolecular distances. These were used as constraints to obtain the energy‐minimized structures shown in Figure 3.^11^ As expected, the carbohydrates are sandwiched between the pyrene units with the NHAc and OMe groups emerging from the portals. This arrangement is reflected in remarkable upfield shifts for the carbohydrate protons, approaching 5 ppm for some CH protons (see Table S2). Interestingly, the data for **9**⋅**13** are most consistent with a twist‐boat structure for the carbohydrate, which is consistent with the all‐equatorial preference of the temple architecture (see Figure 1).

**Figure 3:**
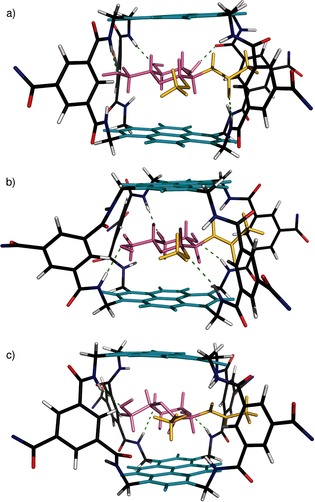
NMR structures of a) eclipsed receptor **5** and GlcNAc‐β‐OMe (**2**), b) staggered receptor **9** and GlcNAc‐β‐OMe (**2**), and c) staggered receptor **9** and GlcNAc‐α‐OMe (**13**). Pyrene units are shown in cyan and carbohydrates in pink, with OMe and NHAc groups highlighted in yellow. Hydrogen bonds are shown as green dashed lines. The water‐solubilizing side chains are omitted.

As mentioned earlier, we had previously shown that biphenyl‐based receptor **4** is capable of binding glycopeptide **3** with *K*_a_=10^3^ m ^−1^. It was interesting to determine whether **5** and **9** would show higher affinities for this substrate. An NMR titration of **9** and **3** suggested binding that was fast on the NMR timescale and could not be quantified. However, addition of **3** to eclipsed receptor **5** in D_2_O at pH 7 yielded new signals, implying binding with slow exchange.^7^ The growth of these signals was analyzed as above to give a remarkable binding constant of *K*_a_=67 000 m ^−1^.^12^ An NMR structure was determined for the core region of the complex (macrotricycle+GlcNAc) based on 89 integrated signals from a 900 MHz NOESY spectrum (see Figure 4).^7^, ^10^ As expected, the orientation of the O‐GlcNAc unit in the cavity is essentially similar to that in **5**⋅**2**. The positioning of the peptide backbone could not be determined, as the signals could not be assigned owing to overlap between complex and unbound glycopeptide. However, Figure 4 shows a reasonable option derived from energy minimization. The structure reveals that the peptidic portion of **3** can undergo various interactions with the receptor, including the dendritic side chains, presumably accounting for the extra affinity.

**Figure 4:**
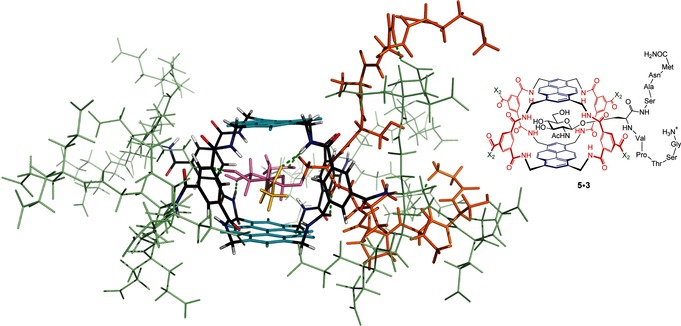
NMR structure of the complex between eclipsed receptor **5** and glycopeptide **3**. Color coding as in Figure 3 except that the peptide backbone is shown in orange and the receptor side chains in pale green. The positioning of the glycopeptide is indicated in the drawing.

The high affinity of **5** for **3** is notable on two counts. First, it approaches the level required for practical applications, such as staining O‐GlcNAcylated proteins in Western blotting (estimated at ca. 10^5^ m ^−1^).^13^ Second, it shows that binding to O‐GlcNAc is context‐selective; the binding to simple model compound **2** is much weaker, and it is only when the additional interactions to the peptide are included that these high levels are achieved. Receptors **5** and **9** provide scope for modifying the side chains, and the synthetic method should be adaptable to related core structures with alternative roof/floor combinations. This raises the prospect of a family of O‐GlcNAc‐binding receptors with useful affinities and complementary selectivities.

In conclusion, we have shown that polycyclic synthetic lectins may be synthesized from rigid, condensed aromatic components by a short and efficient procedure. The method may tend to yield mixtures, but separation was feasible in the present case, and all cage products are likely to have interesting binding properties. The pyrene‐based cages prepared in this work are outstanding receptors for O‐GlcNAc derivatives. Staggered receptor **9** binds the simple glycoside **2** with *K*_a_≈20 000 m ^−1^, a 25‐fold advance on the lectin most commonly used to bind GlcNAc units (WGA). Eclipsed receptor **5** is less effective for **2** but shows even stronger binding to O‐GlcNAc glycopeptide **3** (*K*_a_≈70 000 m ^−1^). These results further demonstrate that biomimetic carbohydrate receptors can show affinities comparable to those of natural lectins and may point to a general strategy for discovering tools for studying O‐GlcNAc and other forms of glycosylation.

## Supporting information

As a service to our authors and readers, this journal provides supporting information supplied by the authors. Such materials are peer reviewed and may be re‐organized for online delivery, but are not copy‐edited or typeset. Technical support issues arising from supporting information (other than missing files) should be addressed to the authors.

Supplementary
