# Supplementary material for: Synthetic Receptors for the High‐Affinity Recognition of O‐GlcNAc Derivatives
Source: Angew Chem Int Ed Engl. 2016 Jan 28;55(10):3387–92. doi: 10.1002/anie.201510611 (PMC5026062; doi:10.1002/anie.201510611)

Angewandte Chemie
Eine Zeitschrift der Gesellschaft Deutscher Chemiker

Supporting Information

Synthetic Receptors for the High-Affinity Recognition of O-GlcNAc Derivatives

Pablo Rios, Tom S. Carter, Tiddo J. Mooibroek, Matthew P. Crump, Micke Lisbjerg, Michael Pittelkow, Nitin T. Supekar, Geert-Jan Boons, and Anthony P. Davis**

anie_201510611_sm_miscellaneous_information.pdf

Contents

1. Synthetic Procedures **3**
General Experimental 3
1,3,6,8-Tetrabromopyrene 5
1,3,6,8-Tetrakis(hydroxymethyl)pyrene 7
1,3,6,8-Tetrakis(azidomethyl)pyrene 8
Hydrochloride salt of 1,3,6,8-tetrakis(aminomethyl)pyrene (7) 9
Pyrenyl "half-receptor" 12 10
Bis-pyrenyl cage receptors 5 and 9 12
O-Protected receptors 6 and 10 13
Water-soluble receptors 5 and 9 21
Glycopeptide 3 22

2. NMR studies on receptors 5 and 9 .... **23**
Eclipsed receptor 5 23
Staggered receptor 9 27

3. Binding Studies **31**
Eclipsed receptor 5 34
Methyl *N*-acetyl-β-D-glucosaminide (2) 34
Glycopeptide (3) 37
Methyl *N*-acetyl-α-D-glucosaminide (13) 39
N-Acetyl-D-glucosamine (14) 39
Methyl β-D-glucoside (15) 40
D-Glucose (16) 41
D-Mannose 42
D-Galactose 42
D-Cellobiose 43
N-Acetyl-D-galactosamine 43
N-Acetyl-D-mannosamine 44
N,N′-Diacetyl-D-chitobiose 44
Staggered receptor 9 45
Methyl *N*-acetyl-β-D-glucosaminide (2) 45
Glycopeptide (3) 47
Methyl *N*-acetyl-α-D-glucosaminide (13) 47
N-Acetyl-D-glucosamine (14) 49
Methyl β-D-glucoside (15) 51

D-Glucose (16) 53
D-Mannose 54
D-Galactose 54
D-Cellobiose 55
N-Acetyl-D-galactosamine 55
N-Acetyl-D-mannosamine 56
N,*N′*-Diacetyl-D-chitobiose 57

4. Structural studies on receptor-guest complexes 58

Table S2. Chemical shifts (δ in ppm) in 9:1 H_2O:D_2O of GlcNAc-β-OMe (2) unbound and when bound to receptors 4, 5, or 9. The values in brackets denote the change in chemical shift (Δδ) on complex formation. All CH protons of the sugar's core are much more shielded when bound by bis-pyrenyls 5 and 9 (up to -4.73) compared to bis-biphenyl 4 (up to-2.40) 59

Eclipsed receptor 5 with methyl *N*-acetyl-β-D-glucosaminide (2) 60

Assignment of ^{1}H NMR peaks 60
2D ^{1}H NMR Spectra with assignments (600 MHz) 61
Distances obtained from NOE data and subsequent molecular modeling 68
Images of molecular model of 5 + GlcNAc-β-OMe (2) 69

Eclipsed receptor 5 with glycopeptide 3 72

Assignment of ^{1}H NMR peaks 73
2D ^{1}H NMR Spectra with assignments (900 MHz) 74
Distances obtained from NOE data and subsequent molecular modeling 83
Images of molecular model of 5 + glycopeptide 3 84

Staggered receptor 9 with methyl *N*-acetyl-β-D-glucosaminide 87

Assignment of ^{1}H NMR peaks 87
2D ^{1}H NMR Spectra with assignments (600 MHz) 88
Distances obtained from NOE data and subsequent molecular modelling 93
Images of molecular model of 9 + GlcNAc-*β*-OMe (2) 94

Staggered receptor 9 with methyl *N*-acetyl-α-D-glucosaminide (13) 97

Assignment of ^{1}H NMR peaks 97
2D ^{1}H NMR Spectra with assignments (600 MHz) 98
Distances obtained from NOE data and subsequent molecular modeling 109
Images of Molecular Model of 9 + GlcNAc-*α*-OMe (13) 110

1. Synthetic Procedures

General Experimental

Commercial reagents were purchased from Sigma-Aldrich, Alfa-Aesar or Acros Organics and were used without further purification unless otherwise specified. Carbohydrates employed in binding studies were purchased from Sigma-Aldrich or Carbosynth Ltd.

Solvents were utilised as supplied unless otherwise stated. Anhydrous THF or CH_2Cl_2 were dried by passing through alumina, using a system manufactured by Anhydrous Engineering. Anhydrous DMF and DMSO were dried by distillation from P_2O_5 and CaH_2, respectively.

^{1}H, ^{13}C, ^{19}F and ^{31}P-NMR spectra were acquired on Varian 400-MR, Jeol Eclipse (300 MHz), Varian VNMRS500a, Varian VNMRS500b or Varian VNMRS600 Cryo spectrometers. All spectra were referenced to their residual internal solvent peak and were acquired at 298 K, unless otherwise specified.

DOSY NMR spectra were run on a Varian VNMRS500a spectrometer, using a one-shot pulse sequence set with gradient length 2 ms, diffusion time 100 ms, maximum gradient strength 25000 and minimum gradient strength 2000. The data was then processed using DOSY Toolbox.[1]

Mass spectra were recorded in the Mass Spectrometry Facility of the University of Bristol. ESI-MS spectra were recorded either on a Orbitrap Elite (Thermo Scientific), Synapt G2S (Waters), Apex IV (Bruker Daltonics) or a MicrOTOF II (Bruker Daltonics). MALDI-MS spectra were recorded either on a UltrafleXtreme (Bruker Daltonics) or a 4700 Proteomics Analyser (Applied Biosystems).

HPLC chromatography was performed using a Waters 600 Controller with a Waters 2998 Photodiode Array Detector. For analytical runs a XSELECT CSH C18 5µm (4.6x150mm) column was used, and for preparative runs a XSELECT CSH Prep C18 5µm OBD (19x250mm) column was utilised, normally with an Acetone-Water solvent mixture.

Peptide synthesis. Fmoc-*L*-Amino acid derivatives and resins were purchased from NovaBioChem and ACS grade *N, N*-dimethylformamide (DMF) from Fisher Chemicals. All other chemical reagents were purchased from Aldrich, Acros, Alfa Aesar and Fischer and used without further purification. All solvents employed were reagent grade. Reversed phase high performance liquid chromatography (RP-HPLC) was performed on an Agilent 1100 series system equipped with an auto-injector, fractio*N*-collector and UVdetector (detecting at 220 nm) using Agilent Zorbax EclipseTM C18 semi preparative column (5 µm, 10 x 250 mm) at a flow rate of 1.5 mL/min. All runs were performed using linear gradients of 0-100% solvent B over 40 min; solvent A = 50 mM ammonium bicarbonate in water, solvent B = acetonitrile. High resolution mass spectra were obtained

1. M. Nilsson, *J. Magn. Reson.* **2009**, *200*, 296.

by using MALDI-ToF (Applied Biosystems 5800 Proteomics Analyzer) with 2,5-dihydroxybenzoic acid or α-cyano-4-hydroxycinnamic acid as an internal standard matrix.

General methods for automated microwave-assisted solid-phase peptide synthesis (MW-SPPS). Peptides were synthesized by established protocols on a CEM Liberty Automated Microwave Peptide Synthesizer equipped with a UV detector using *N*-α-Fmoc-protected amino acids and 2-(1H-benzotriazole-1-yl)-1,1,3,3-tetramethyluronium hexafluorophosphate (HBTU)/1-hydroxybenzotriazole (HOBt) as the activating reagents. Deprotection of the *N*-α-Fmoc was achieved using 20% 4-methyl piperidine in DMF.

General methods for manual MW-SPPS. The couplings of the glycosylated amino acid *N*-α-Fmoc-Ser-(AcO_3-β-d-GlcNAc) was carried out using (2-(7-aza-1H-benzotriazole-1-yl)-1,1,3,3-tetramethyluronium hexafluorophosphate) (HATU) and 1-hydroxy-7-azabenzotriazole (HOAt). Each manual coupling was monitored by standard Kaiser Test. Deprotection of the *N*-α-Fmoc was achieved using 20% 4-methyl piperidine in DMF.

1,3,6,8-Tetrabromopyrene

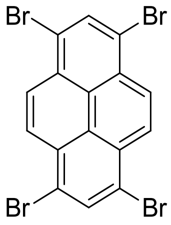

This method is based on that reported by Venkataramana *et al.* with minor modifications.[2] A two-necked 1 L round-bottom flask equipped with a dropping funnel was charged with an external stirrer, pyrene (10.1 g, 50 mmol) and nitrobenzene (350 mL). The dropping funnel was charged with 100 mL of a Br_2 solution in nitrobenzene (2.25 mM, 225 mmol), which was then added dropwise to the pyrene solution at 80 °C. After the addition was complete, the yellow suspension was heated at 120 °C for 16 hours and then cooled to laboratory temperature. The resulting suspension was filtered, the solid washed thoroughly with ethanol and dried *in vacuo* to yield 1,3,6,8-tetrabromopyrene as a pale yellow solid in 96% yield (25.1 g). FT-IR: υ 1590, 1464, 1452, 1226, 1053, 986, 872, 810, 690, 673 cm^{-1}; Anal. Calc'd for $C_{16}H_{16}Br_4$ = C, 37.11; H, 1.17; Br, 61.72; Found: C, 37.05; H, 1.20; Br, 61.68; LRMS (MALDI): *m/z* calculated for $C_{16}H_6Br_4$ $[M]^+$: 513.72, found 513.90 [lit[2] LRMS (EI): m/z (%) = 522 (12), 520 (50), 518 (70), 516 (48), 514 (12) $[M^+]$].

Tetrabutyl pyrene-1,3,6,8-tetracarboxylate

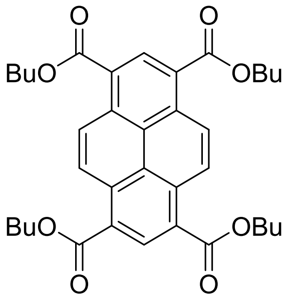

This method is based on that reported by Heck *et al.*, with some modifications.[3] An autoclave was charged with 1,3,6,8–tetrabromopyrene (5.38 g, 10.40 mmol), $Pd(OAc)_2$ (0.23 g, 1.04 mmol) and (±)-BINAP (0.97 g, 1.56 mmol), before applying a N_2 atmosphere. An N_2 saturated mixture of xylenes (120 mL), *N*-butanol (60 mL) and DIPEA (9.1 mL, 52 mmol) was then added and the autoclave contents stirred for 15 min. The autoclave was then pressurized with CO (30 bar), heated to 100 °C and then held at 120 °C for 4 hours. After venting and flushing with N_2, the volatiles were removed *in vacuo*. Purification by column chromatography (EtOAc:Hex, 1:4 to 1:1) gave tetrabutyl pyrene-1,3,6,8-tetracarboxylate (89%, 5.58 g, 9.26 mmol) as a yellow solid. R_f = 0.72 (EtOAc:Hex 1:4).

2. G. Venkataramana, S. Sankararaman, *Eur. J. Org. Chem.* **2005**, 4162.
3. A. Schoenberg, I. Bartoletti, R. F. Heck, *J. Org. Chem.* **1974**, *39*, 3318.

^{1}H-NMR (400 MHz, $CDCl_3$): δ 1.07 (t, 12H, *J* = 7.4 Hz, $-CH_3$), 1.61 (sex, 8H, *J* = 7.4 Hz, $-CH_2C\underline{H}_2CH_3$), 1.91 (quin, 8H, *J* = 6.7 Hz, $CH_2C\underline{H}_2CH_2$-), 4.53 (t, 8H, *J* = 6.6 Hz, $-OC\underline{H}_2CH_2$-), 9.12 (s, 2H, aryl H), 9.24 (s, 4H, aryl H); ^{13}C-NMR (100 MHz, $CDCl_3$): δ 14.0 ($-CH_3$), 19.6 ($-CH_2\underline{C}H_2CH_3$), 31.0 ($-CH_2C\underline{H}_2CH_2$-), 65.8 ($-O\underline{C}H_2CH_2$-), 124.9 (aryl $\underline{C}_q$), 125.6 (aryl $\underline{C}$H), 128.2 (aryl $\underline{C}_q$), 131.3 (aryl $\underline{C}_q$), 132.6 (aryl $\underline{C}_q$), 167.3 ($\underline{C}$O); HRMS (ESI): m/z calculated for $C_{36}H_{42}O_8Na$ $[M+Na]^+$: 625.2777, found 625.2772.

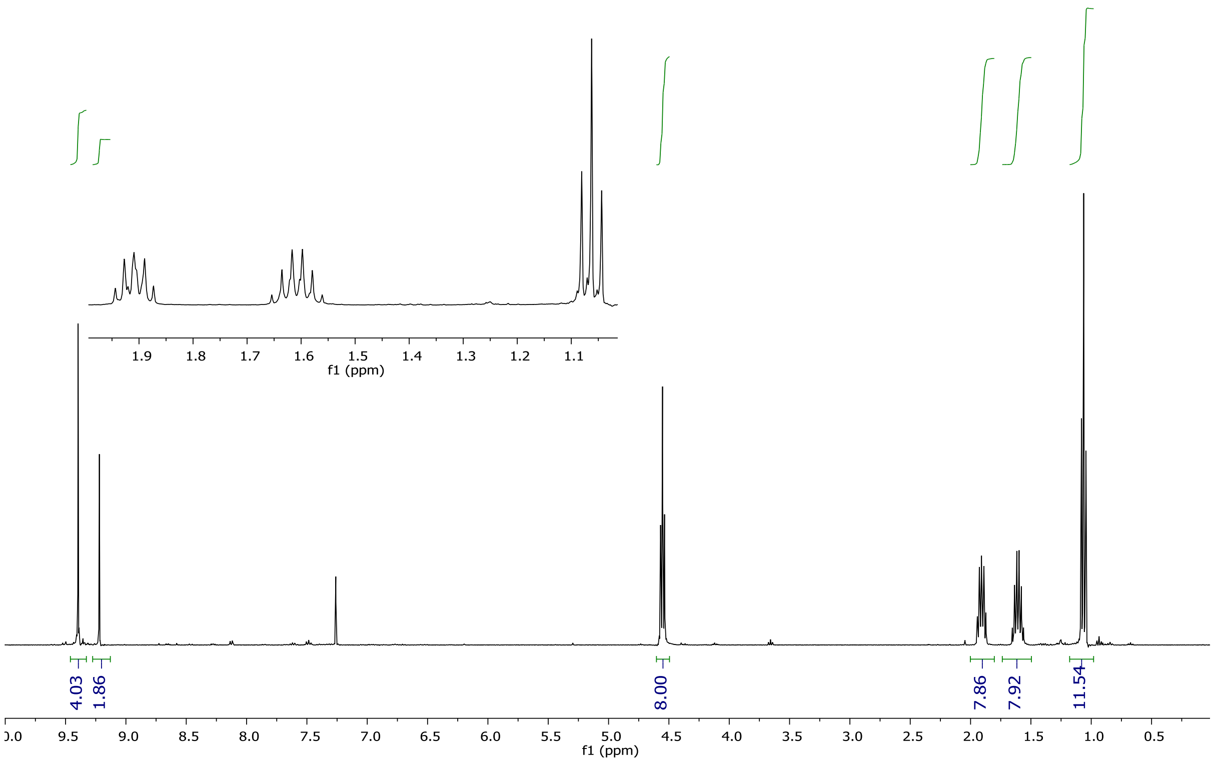

Figure S1. ^{1}H-NMR spectrum (400 MHz, $CDCl_3$) of tetrabutyl pyrene-1,3,6,8-tetracarboxylate..

1,3,6,8-Tetrakis(hydroxymethyl)pyrene

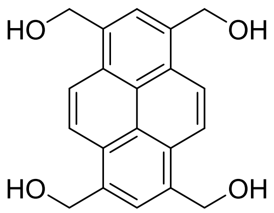

A solution of tetrabutyl pyrene-1,3,6,8-tetracarboxylate (4.56 g, 7.57 mmol), in anhydrous N_2-saturated THF (100 mL) and methanol (7 mL, 171 mmol) was prepared under a N_2 atmosphere. $LiBH_4$ (4 M in THF, 42.5 mL, 170 mmol) was added dropwise to the pale yellow solution, which consequently foamed and turned purple. The reaction mixture was then heated to reflux for 16 hours, before cooling to RT and quenching with water until foaming ceased. The resulting suspension was filtered, washed with water (3 x 25 mL) and dried *in vacuo* to afford 1,3,6,8-tetrakis(hydroxymethyl)pyrene (~100%, 2.44 g, 7.57 mmol) as a pale yellow solid. ^{1}H-NMR (400 MHz, $(CD_3)_2SO$): δ 5.24 (s, 8H, -C$\underline{H}_2$-OH), 5.50 (s, 4H, -O$\underline{H}$), 8.22 (s, 2H, aryl H), 8.34 (s, 4H, aryl H); ^{13}C-NMR (100 MHz, $(CD_3)_2SO$): δ 62.1 (-$\underline{C}H_2OH$), 123.3 (aryl $\underline{C}_q$), 125.3 (aryl $\underline{C}$H), 125.7 (aryl $\underline{C}$H), 127.5 (aryl $\underline{C}_q$), 135.8 (aryl $\underline{C}_q$). HRMS (MALDI): m/z calculated for $C_{20}H_{18}O_4^+$ [M]$^+$: 322.1205, found 322.1195.

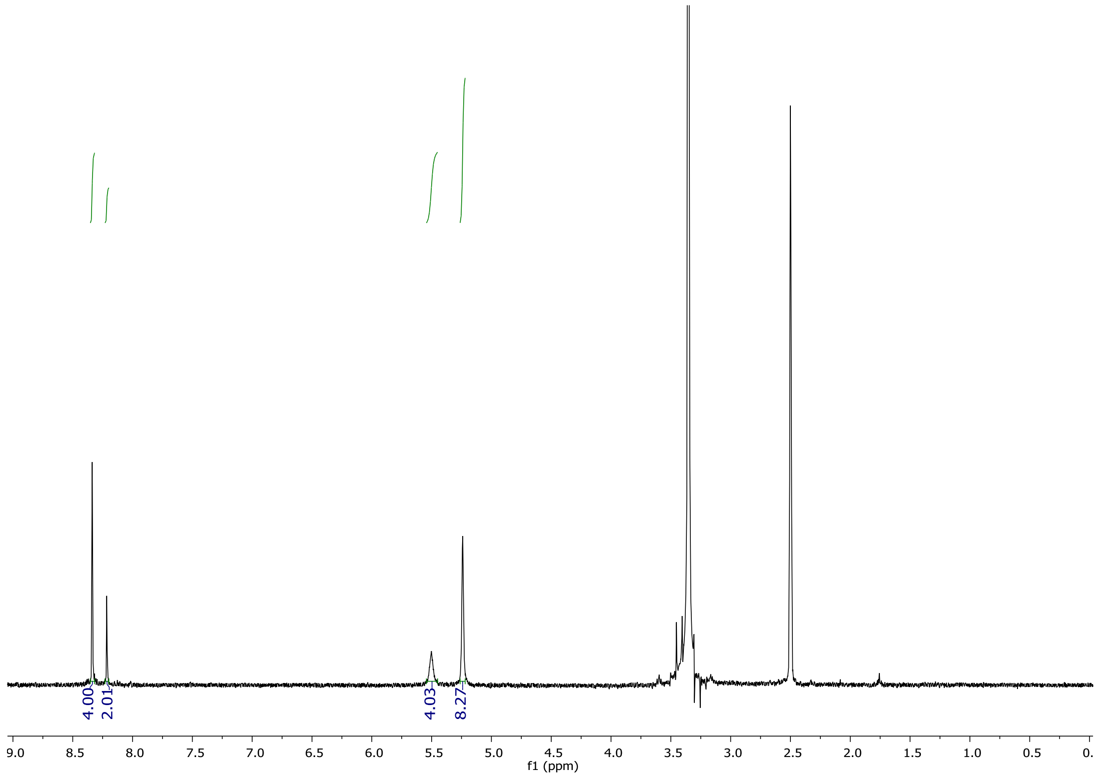

Figure S2. ^{1}H-NMR spectrum (400 MHz, $(CD_3)_2SO$) for 1,3,6,8-tetrakis(hydroxymethyl)pyrene.

1,3,6,8-Tetrakis(azidomethyl)pyrene

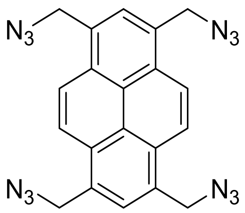

A suspension of 1,3,6,8-tetrakis(hydroxymethyl)pyrene (200 mg, 0.62 mmol) in diphenyl phosphoryl azide (641 µL, 2.98 mmol) and anhydrous DMF (1.5 mL) was cooled to 0 °C. To this suspension was added 1,8-diazabicyclo[5.4.0]undec-7-ene (446 µL, 2.98 mmol) in a dropwise manner. The reaction mixture was stirred at 0 °C for 10 minutes, before warming to RT and stirring for a further 30 minutes. The resulting suspension was coevaporated with toluene (3 x 5 mL) and the residue purified by column chromatography (CH_2Cl_2), affording 1,3,6,8-tetrakis(azidomethyl)pyrene (52 %, 136 mg, 0.32 mmol) as a brown solid. R_f = 0.85 (CH_2Cl_2); ^{1}H-NMR (400 MHz, ($CDCl_3$): δ 5.11 (s, 8H, -C<u>H</u>$_2$-N_3), 8.06 (s, 2H, aryl H), 8.42 (s, 4H, aryl H); ^{13}C-NMR (100 MHz, ($CDCl_3$): δ 52.0 (-<u>C</u>H_2N_3), 124.6 (aryl <u>C</u>$_q$), 125.1 (aryl <u>C</u>H), 129.2 (aryl <u>C</u>H), 130.3 (aryl <u>C</u>$_q$), 130.4 (aryl <u>C</u>$_q$); FT-IR (main absorptions): *υ* 2923, 2990, 2853, 2103 (-N_3), 2067 (-N_3), 1610, 1574, 1459, 1232, 885, 866, 837, 705, 646 cm^{-1}; HRMS-MALDI: m/z calculated for $C_{20}H_{15}N_{10}^+$ [M + H –N_2]$^+$: 395.15, found 395.5, for $C_{20}H_{15}N_8^+$ [M + H –2N_2]$^+$: 367.14, found 367.5, for $C_{20}H_{15}N_6^+$ [M + H –3N_2]$^+$: 339.14, found 339.4.

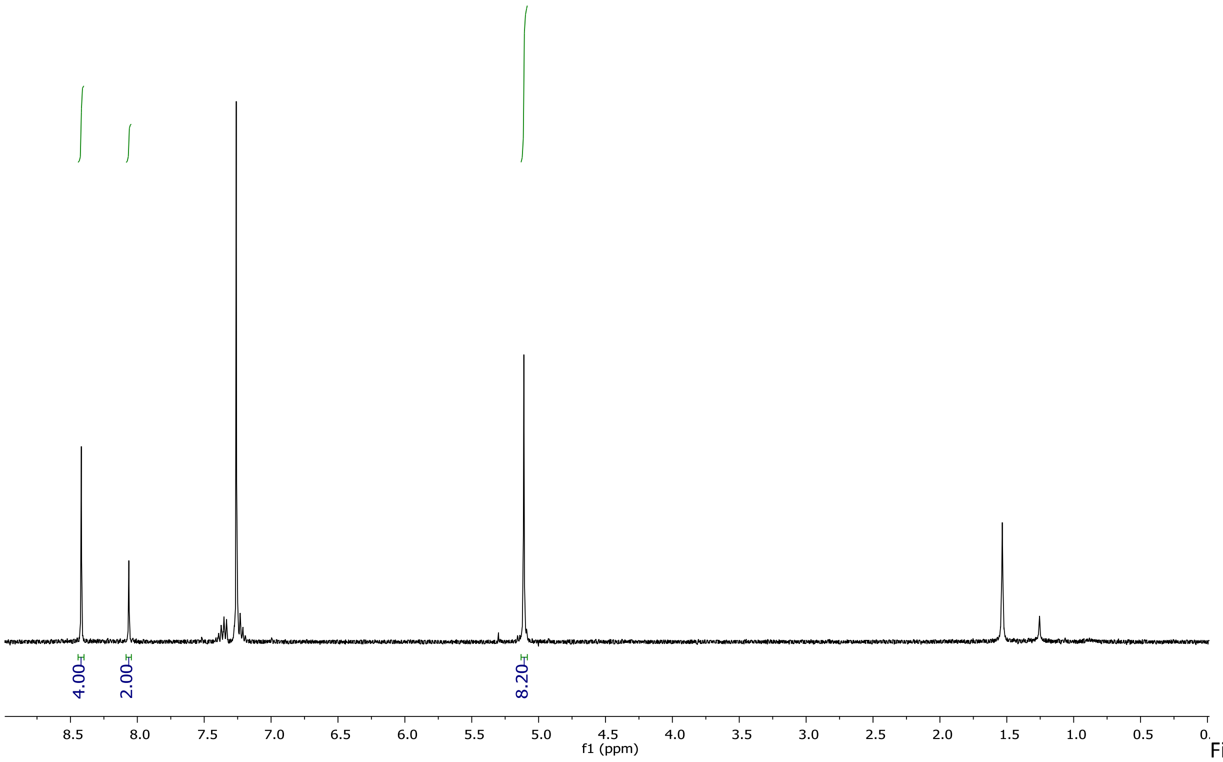

Figure S3. ^{1}H-NMR spectrum (400 MHz, $CDCl_3$) for 1,3,6,8-tetrakis(azidomethyl)pyrene.

Hydrochloride salt of 1,3,6,8-tetrakis(aminomethyl)pyrene (7)

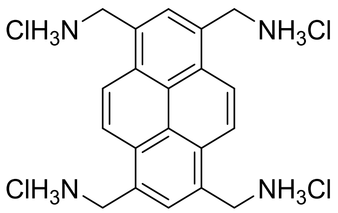

To a suspension of 1,3,6,8-tetrakis(azidomethyl)pyrene (318 mg, 0.75 mmol) in anhydrous THF (20 mL) was added PPh_3 (1.44 g, 5.50 mmol). This was heated to 60°C and stirred for 1 hour, water (5 mL) was then added and the reaction stirred for a further 16 hours. After cooling to RT, hydrochloric acid (37% in water, 5 mL) was added and the solution stirred for 2 hours. The reaction was then washed with DCM (3 x 30 mL), before the aqueous phase was filtered and evaporated under reduced pressure to give the product (93%, 325 mg, 0.70 mmol) as an ochre solid. ^{1}H-NMR (400 MHz, (D_2O): δ 5.07 (s, 8H, -C$\underline{H}_2$-NH_3Cl), 8.34 (s, 2H, aryl H), 8.64 (s, 4H, aryl H); ^{13}C-NMR (100 MHz, (D_2O): δ 40.3 (-$\underline{C}H_2NH_3Cl$), 123.9 (aryl $\underline{C}$H), 124.7 (aryl $\underline{C}_q$), 127.5 (aryl $\underline{C}_q$), 129.5 (aryl $\underline{C}_q$), 130.1 (aryl $\underline{C}$H); MS (MALDI): m/z calculated for $C_{20}H_{22}N_4^+$ [M - 4HCl]$^+$: 318.18, found 318.3.

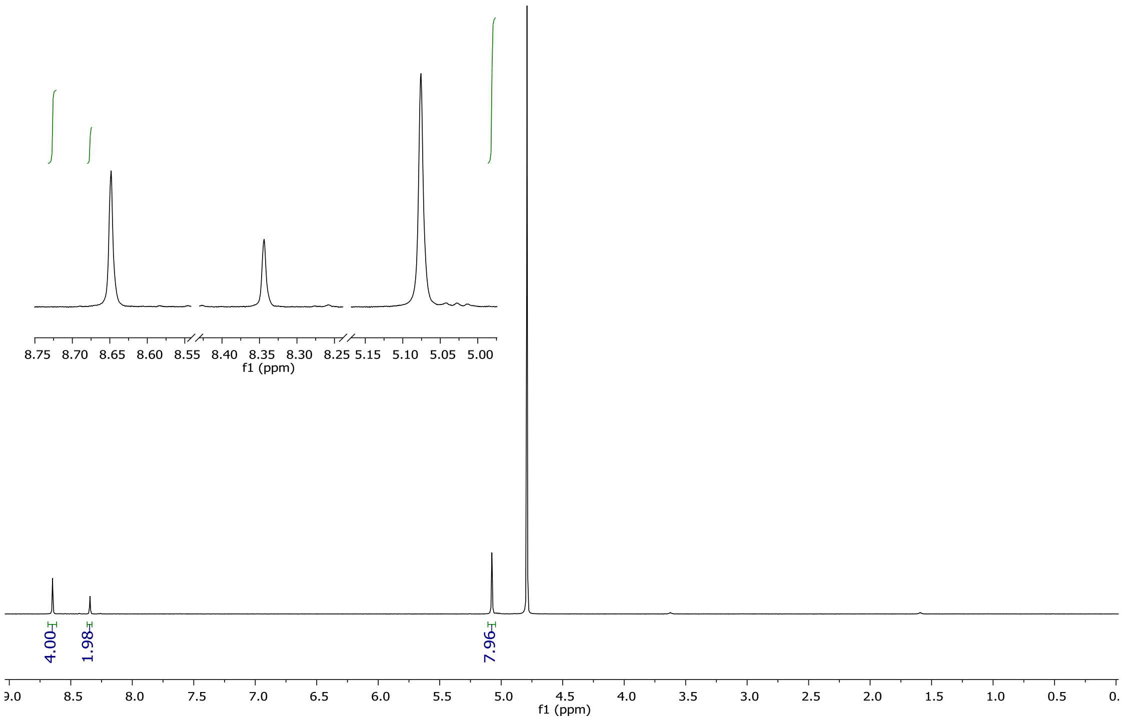

Figure S4. ^{1}H-NMR spectrum (400 MHz, D_2O) for the hydrochloride salt of 1,3,6,8-tetrakis(aminomethyl)pyrene **7**.

Pyrenyl "half-receptor" 12

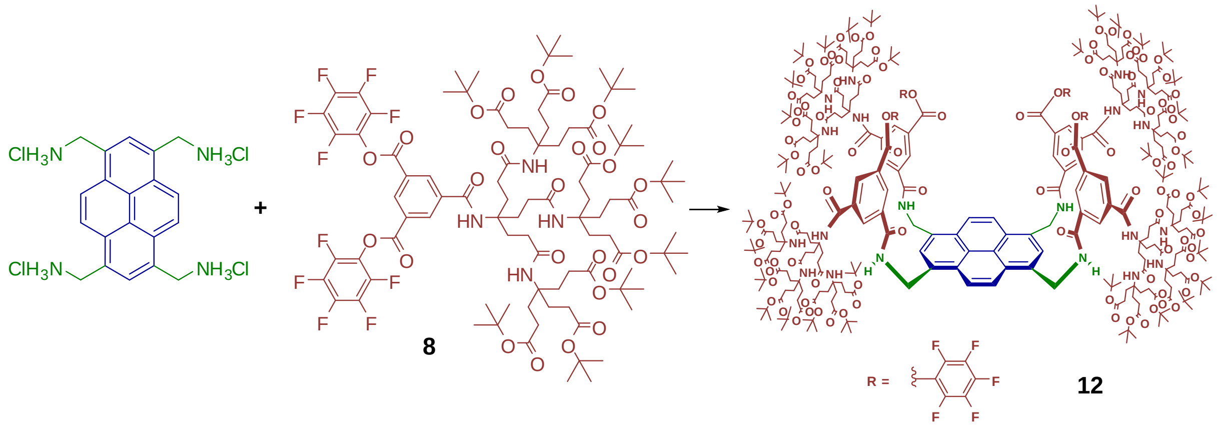

A solution of pyrene-1,3,6,8-tetrayltetramethanammonium chloride (10.3 mg, 22.0 µmol) and bis-pentafluorophenyl ester **8**[4] (350 mg, 0.178 mmol) in a mixture of THF (2.56 mL) and water (0.56 mL) was prepared. A solution of DIPEA (14.7 µL, 89.0 µmol) in THF (1.28 mL) and water (0.28 mL) was added dropwise over 4 hours using a syringe pump. The resulting solution was stirred for 16 hours, before filtering (0.2 µm syringe filter) and purification by HPLC (acetone:H_2O, 70:30 to 100:0 over 45 min; flow rate: 17 mL/min). The component eluting at 39.5 min was collected, the volatiles were evaporated and the sample was freeze-dried to yield the product **12** (78 mg, 10.5 µmol, 47%) as a yellow solid. ^{1}H NMR (500 MHz, $CDCl_3$/CD_3OD 92:8): δ 1.30 (s, 324H, (C<u>H</u>$_3$)$_3$C-), 1.81 (m, 72H, -C<u>H</u>$_2$CH$_2$COO-), 2.05 (m, 96H, -CH$_2$C<u>H</u>$_2$CO), 2.11 (m, 24H, -C<u>H</u>$_2$CH$_2$CONH-), 5.30 (s, 8H, Py-C<u>H</u>$_2$-), 6.98 (s, 12H, -CON<u>H</u>-), 8.10 (s, 2H, Py-H), 8.35 (s, 4H, Ph-CON<u>H</u>-), 8.41 (s, 4H, Py-H), 8.65 (s, 4H, Ph-H), 8.65 (s, 4H, -CH$_2$N<u>H</u>COPh-), 8.68 (s, 4H, Ph-H), 8.74 (s, 4H, Ph-H); ^{13}C-NMR (125 MHz, ($CDCl_3$)): δ 28.0 ((<u>C</u>H$_3$)$_3$C), 29.5 (<u>C</u>H$_2$CH$_2$COO), 29.7 (CH$_2$<u>C</u>H$_2$CO), 31.5 (<u>C</u>H$_2$CH$_2$CONH), 42.4 (Py-<u>C</u>H$_2$), 57.7 (<u>C</u>$_q$-NH), 80.7 ((CH$_3$)$_3$<u>C</u>), 123.4 (Py), 125.7 (Py <u>C</u>$_q$), 127.9 (Py), 128.5 (Py <u>C</u>$_q$), 131.2 (Py <u>C</u>$_q$), 131.7 (Ph), 131.8 (Ph <u>C</u>$_q$), 132.0 (Ph), 132.0 (Ph <u>C</u>$_q$), 132.1 (Ph <u>C</u>$_q$), 132.5 (Ph), 161.5 (Ph-<u>C</u>OO), 165.6 (Ph-<u>C</u>ONH), 165.8 (Ph-<u>C</u>ONH), 172.7 (<u>C</u>O dendrimer); ^{19}F NMR (470 MHz, $CDCl_3$) δ -152.7 (d, 8F, *J* = 19.6 Hz, ortho aryl C-<u>F</u>), -158.0 (t, 4F, *J* = 20.7 Hz, para aryl C-<u>F</u>), -162.5 (t, 8F, *J* = 19.5 Hz, meta aryl C-<u>F</u>); HRMS (ESI): m/z calculated for $C_{384}H_{562}F_{20}N_{20}O_{100}Na_2^{2+}$ [M+2Na]$^{2+}$: 3739.95, found 3740.97.

4. H. Destecroix, C. M. Renney, T. J. Mooibroek, T. S. Carter, P. F. N. Stewart, M. P. Crump, A. P. Davis, *Angew. Chem. Int. Ed.* **2015**, *54*, 2057.

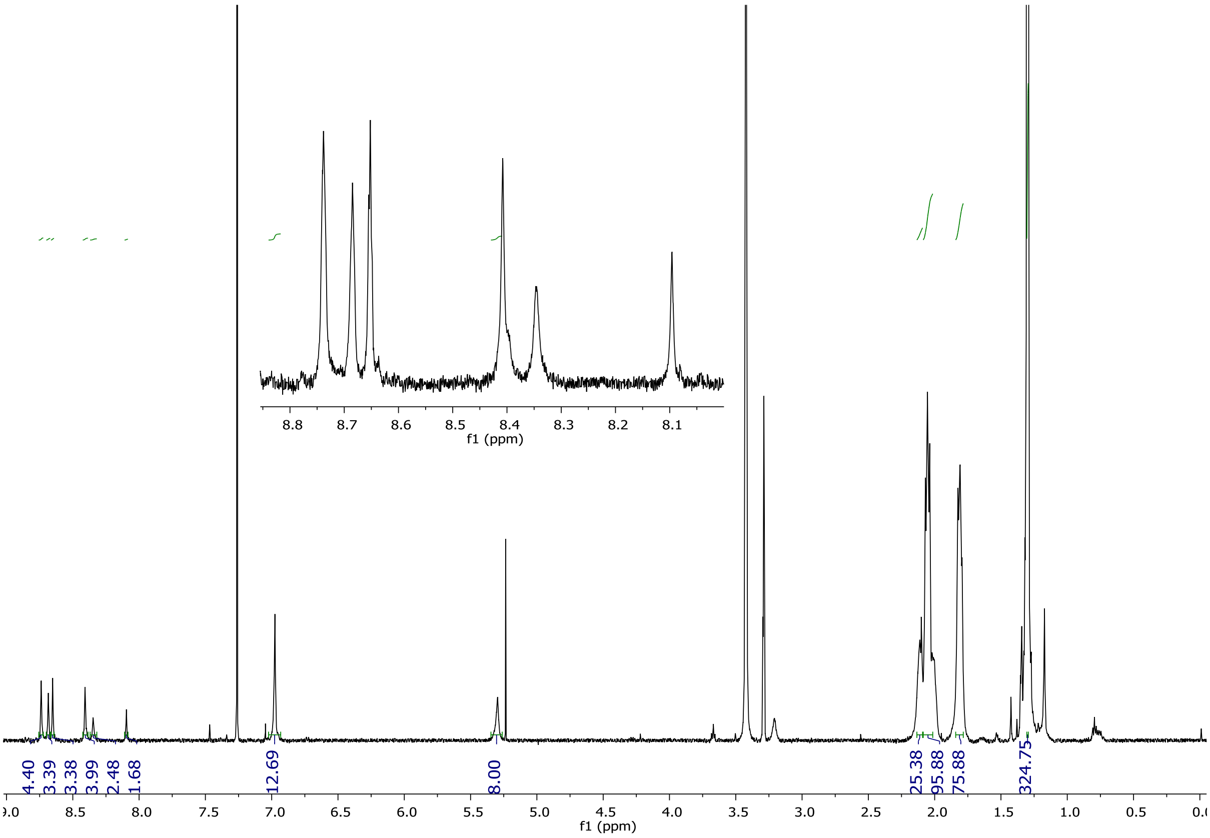

Figure S5. ^{1}H-NMR spectrum (500 MHz, $CDCl_3/CD_3OD$ 92:8) for pyrene "half-receptor" **12**. Contains residual DCM and MeOH (5.24 and 3.42 ppm respectively).

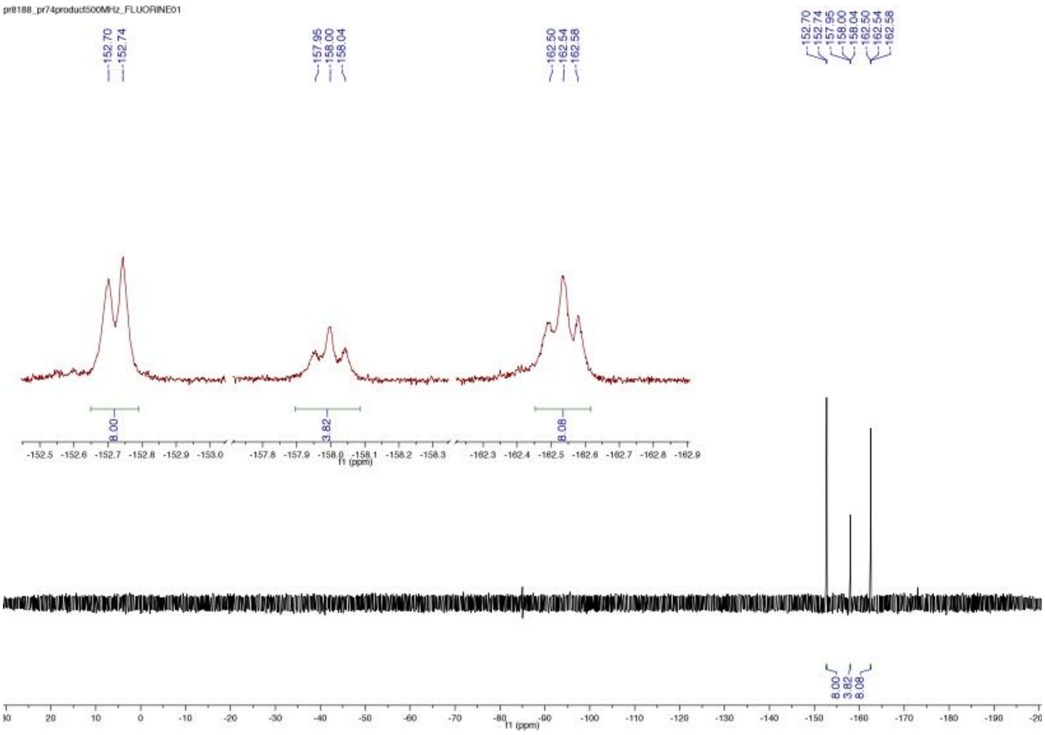

Figure S6. ^{19}F-NMR spectrum (470 MHz, $CDCl_3$) for pyrene "half-receptor" **12**.

Bis-pyrenyl cage receptors 5 and 9

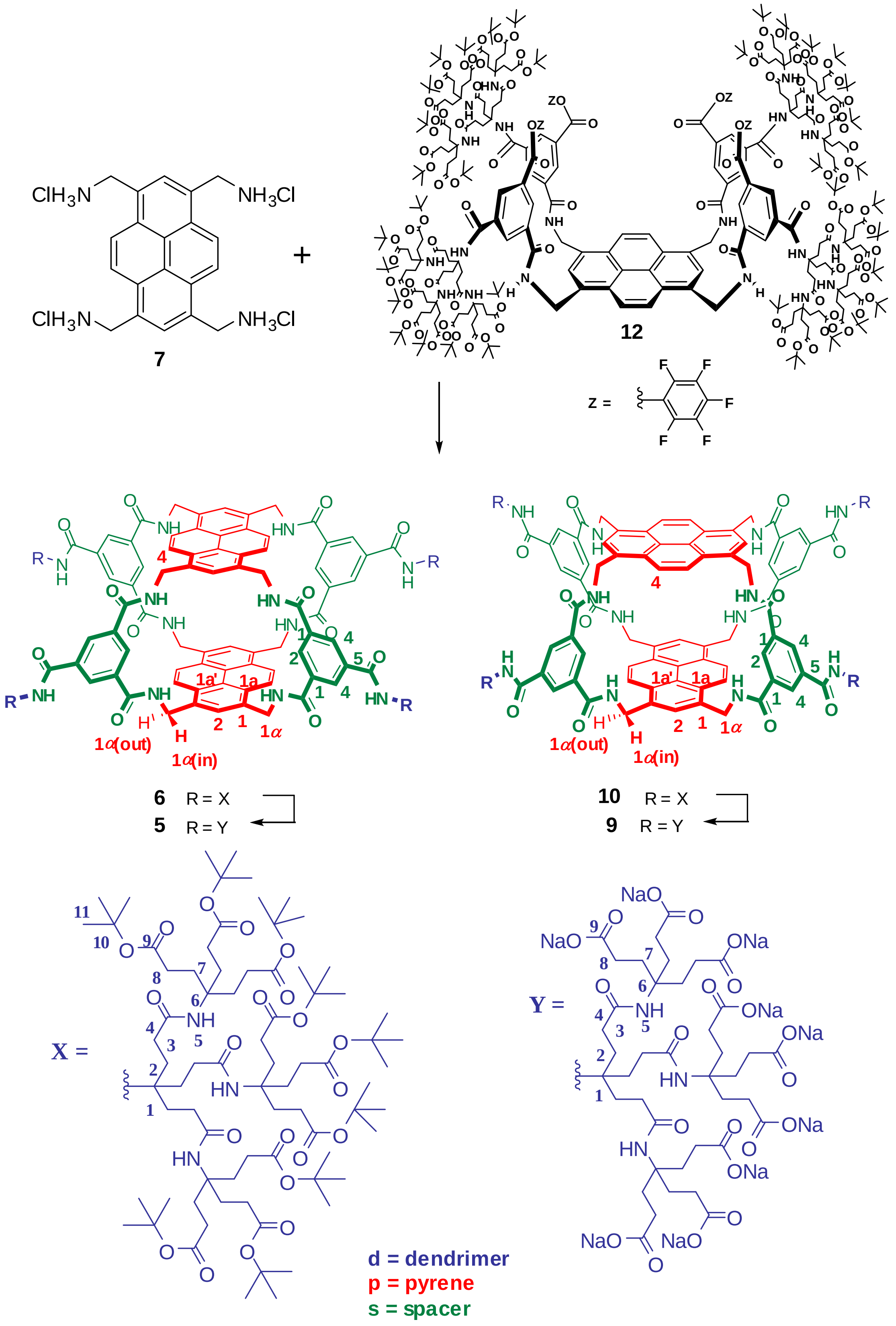

Scheme S1. Macrocyclisation of **12** + **7** to give O-protected cages **6** and **10**, followed by deprotection to receptors **5** and **9**. The numbering system used for the assignment and discussion of NMR spectra is also shown. Pyrenyl proton designations (red) are prefixed with “p” (i.e. p4, p2α etc.), spacer protons (green) are prefixed with “s” , and protons from the dentritic side-chains are prefixed with “d”.

O-Protected receptors 6 and 10

A solution of pyrene "half-receptor" **12** (94 mg, 12.6 μmol), hydrochloride salt of 1,3,6,8-tetrakis(aminomethyl)pyrene (5.87 mg, 12.6 μmol) in THF (5.18 mL) and H_2O (1.14 mL) was prepared. A solution of DIPEA (16.7 μL, 0.101 mmol) in THF (5.18 mL) and H_2O (1.14 mL) was then added in a dropwise manner with a syringe pump over 6 hours. The resulting yellow solution was left stirring for 16 hours, after which the solvent was removed under reduced pressure. The resulting solid was dissolved in DCM and washed with NH_4Cl (sat., 30 mL), brine (30 mL) and H_2O (30 mL). The combined aqueous phases were extracted with DCM (3 x 30 mL), and the combined organic phases were dried over $MgSO_4$, filtered and concentrated under reduced pressure. The sample was dissolved in 18% H_2O in THF, filtered (0.2 μm syringe filter) and purified by HPLC (acetone:H_2O, 81:19 to 92:8 over 130 min; flow rate: 17 mL/min).[5] The components eluting at 113 min and 117 min were collected, the volatiles were evaporated and the samples were freeze-dried to yield the "staggered" cage **10** (20 mg, 2.85 μmol, 23%; from the faster-eluting peak), and the eclipsed cage **6** (30 mg, 4.27 μmol, 34%; from the slower-eluting peak). The structures were initially assigned as explained in Figure S13, and the assignments were confirmed as part of the structural determinations discussed in final section of this supporting information.

Data for **10** (eluting at 113 min);

^{1}H-NMR (MeOD, 500 MHz): δ 1.44 (s, 324H, d11), 1.98 (m, 72H, d7), 2.18 (m, 24H, d3), 2.23 (m, 72H, d8), 2.31 (m, 24H, d2), 4.71 (d, 8H, *J* = 14.9 Hz, p1α(in)), 5.63 (d, 8H, *J* = 14.9 Hz, p1α(out)), 7.83 (s, 4H, s2), 8.12 (s, 4H, p2), 8.37 (s, 8H, p4), 8.60 (s, 8H, s4). ^{13}C-NMR (MeOD, 125 MHz): δ 27.0 (d11), 29.0 (d7), 29.3 (d8), 30.7 (d3), 31.0 (d2), 41.6 (p1α), 57.4 (d6), 80.3 (d10), 123.2 (p4), 126.7 (s5<u>C</u>O), 126.8 (s2), 128.8 (p1), 129.5 (s4), 131.1 (p2), 166.8 (s3<u>C</u>O), 173.0 (d9). HRMS (ESI): m/z calculated for $C_{380}H_{580}N_{24}O_{96}Na_4^{4+}$ $[M+4Na]^{4+}$: 1778.2230, found 1778.0235.

[5]HPLC purification tests using the ACE 5 C18-PFP (150 x 4.6 mm) analytical column, showed superior performance (acetone:H_2O, 84:16 to 92:8 over 60 min; flow rate: 1 mL/min. **P1** = 32 min, **P2** =34 min). This suggests that the separation method could be improved in future work by the use of specialist stationary phases.

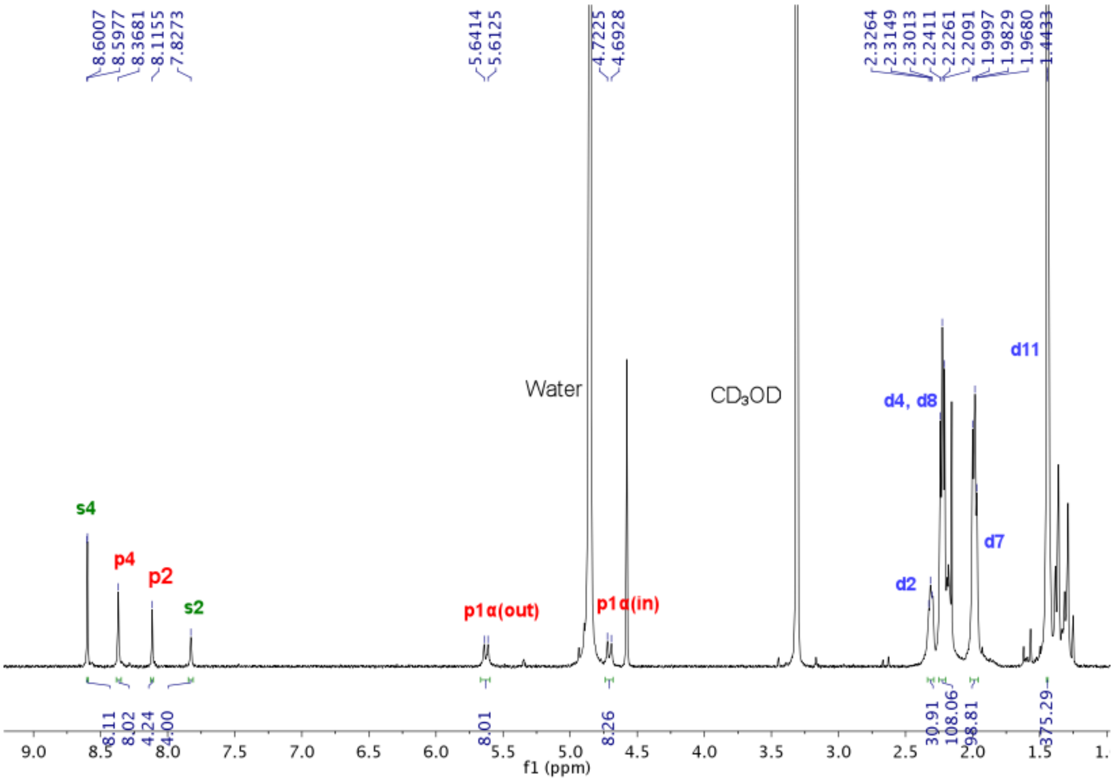

Figure S7. ^{1}H-NMR spectrum (500 MHz, MeOD) for compound **10**.

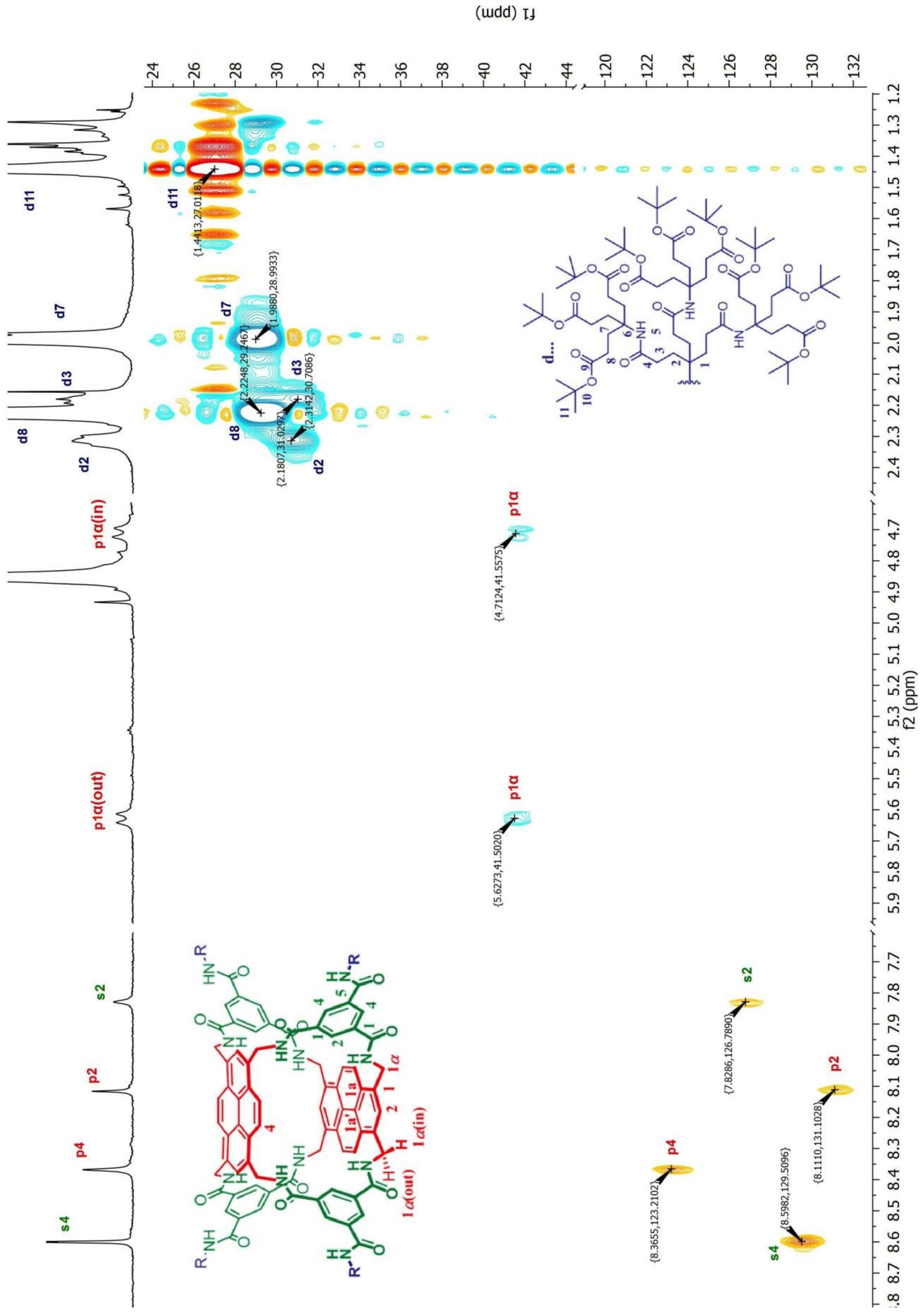

Figure S8. HSQC spectrum (500 MHz, MeOD) for compound **10**.

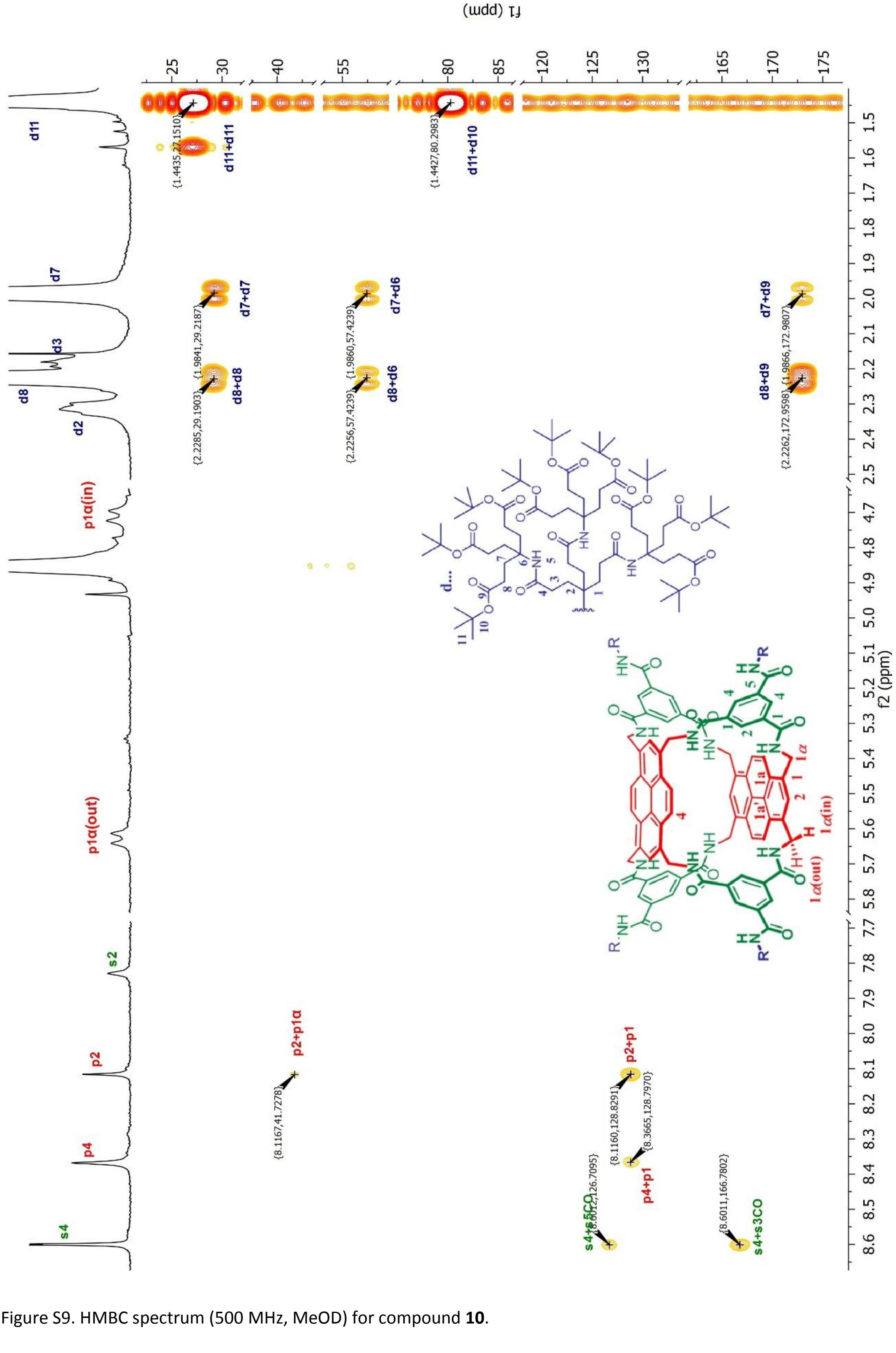

Figure S9. HMBC spectrum (500 MHz, MeOD) for compound **10**.

Data for **6** (eluting at 117 min);

^{1}H-NMR (MeOD, 500 MHz): δ 1.44 (s, 324H, d11), 1.99 (m, 72H, d7), 2.18 (m, 24H, d3), 2.23 (m, 72H, d8), 2.32 (m, 24H, d2), 4.76 (d, 8H, *J* = 14.2 Hz, p1α(in)), 5.57 (d, 8H, *J* = 14.2 Hz, p1α(out)), 7.81 (s, 4H, s2), 8.10 (s, 4H, p2), 8.33 (s, 8H, p4), 8.59 (s, 8H, s4). ^{13}C-NMR (MeOD, 125 MHz): δ 27.1 (d11), 29.1 (d7), 29.4 (d8), 30.9 (d2), 31.1 (d3), 42.0 (p1), 57.5 (d6), 80.3 (d10), 123.2 (p4), 126.4 (s2), 126.5 (s5CO), 128.8 (p1), 129.6 (s4), 131.0 (p2), 167.0 (s3CO), 173.0 (d9); HRMS (ESI): m/z calculated for $C_{380}H_{580}N_{24}O_{96}Na_4^{4+}$ $[M+4Na]^{4+}$: 1778.2230, found 1778.0175.

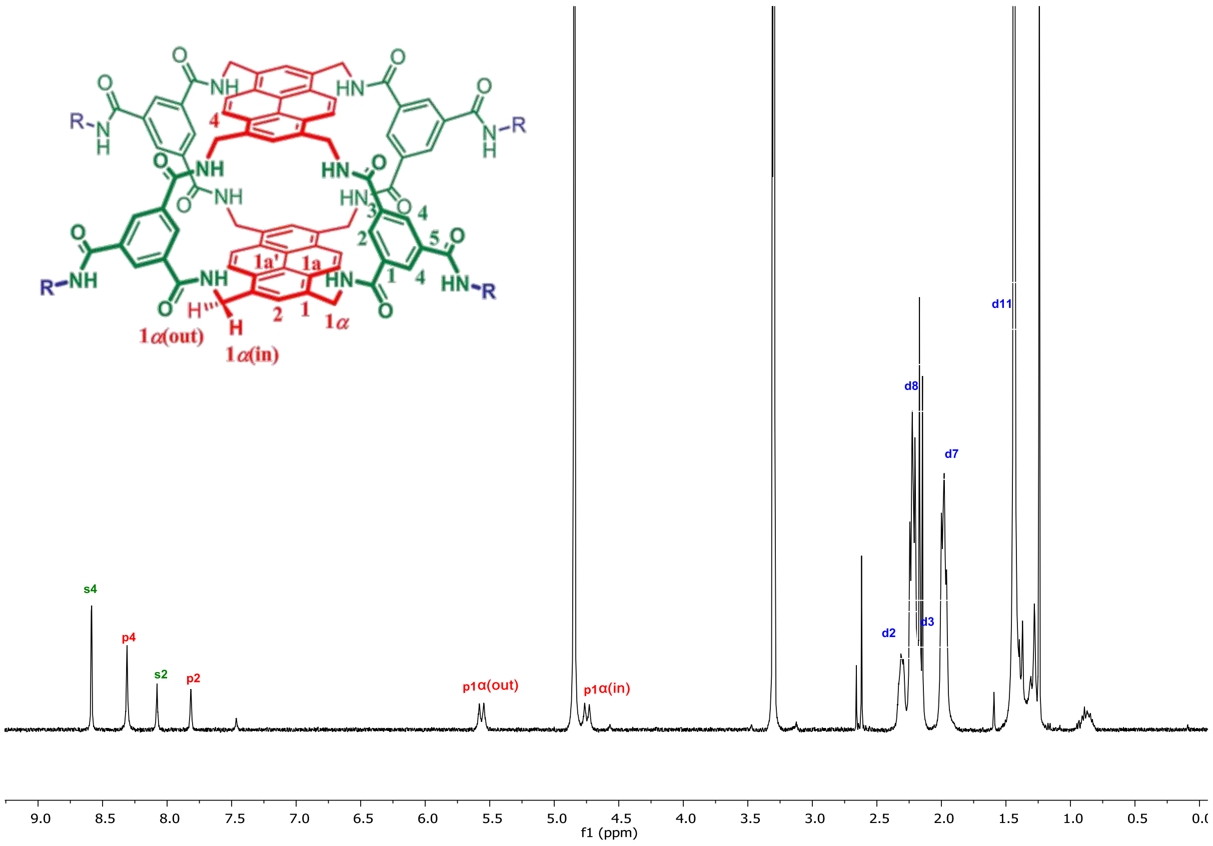

Figure S10. ^{1}H-NMR spectrum (500 MHz, MeOD) for compound **6**.

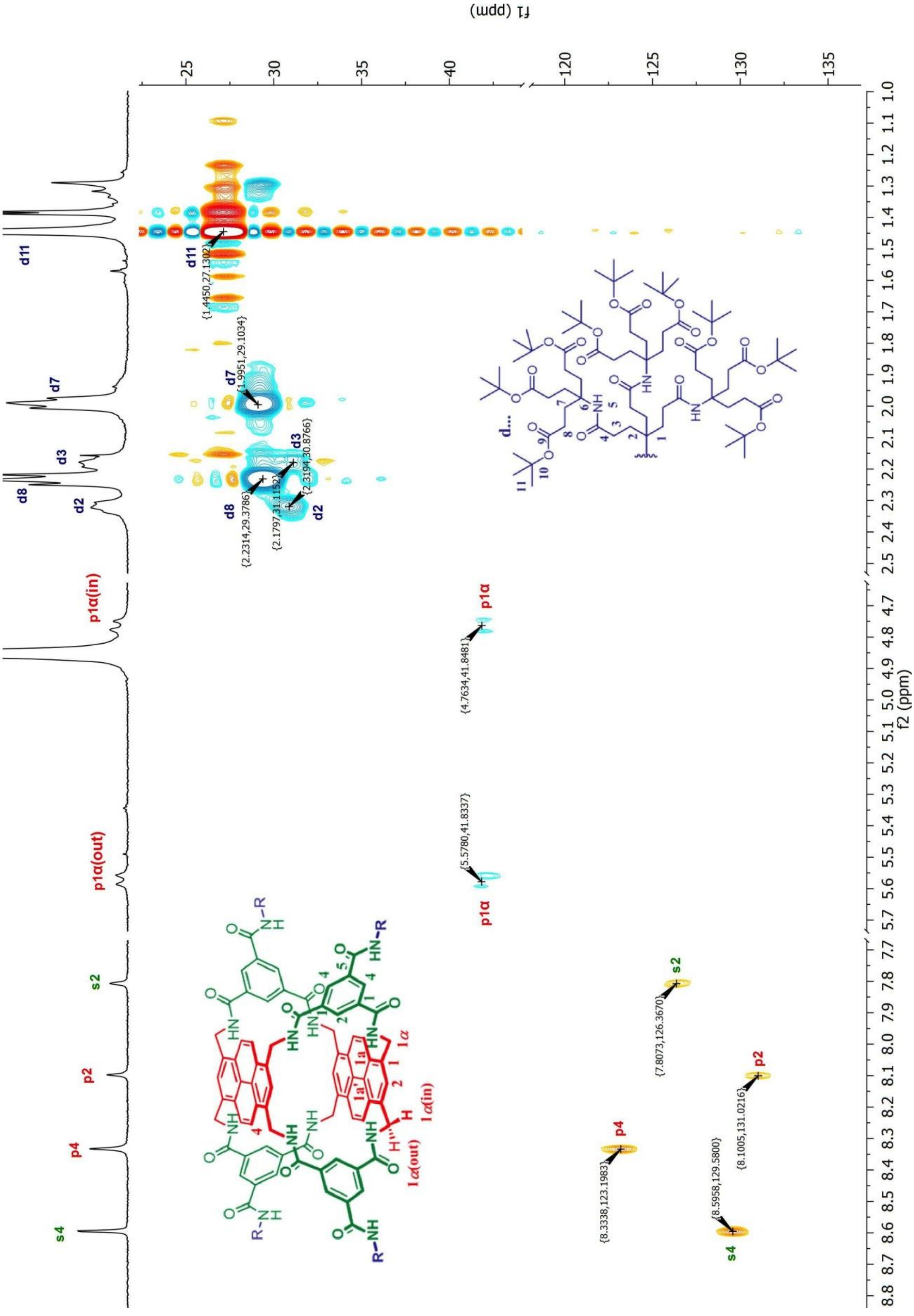

Figure S11. HSQC spectrum (500 MHz, MeOD) for compound **6**.

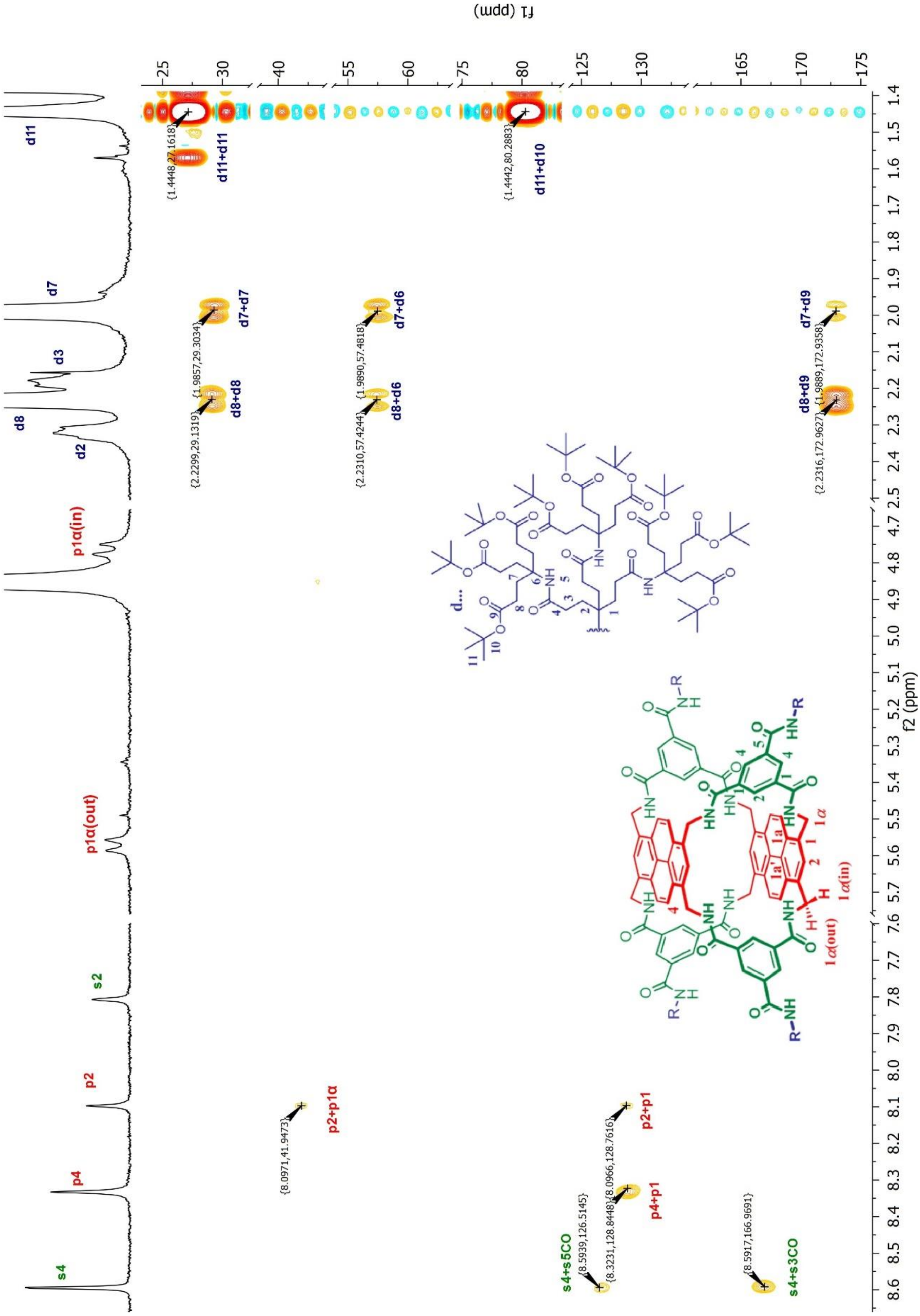

Figure S12. HMBC spectrum (500 MHz, MeOD) for compound **6**.

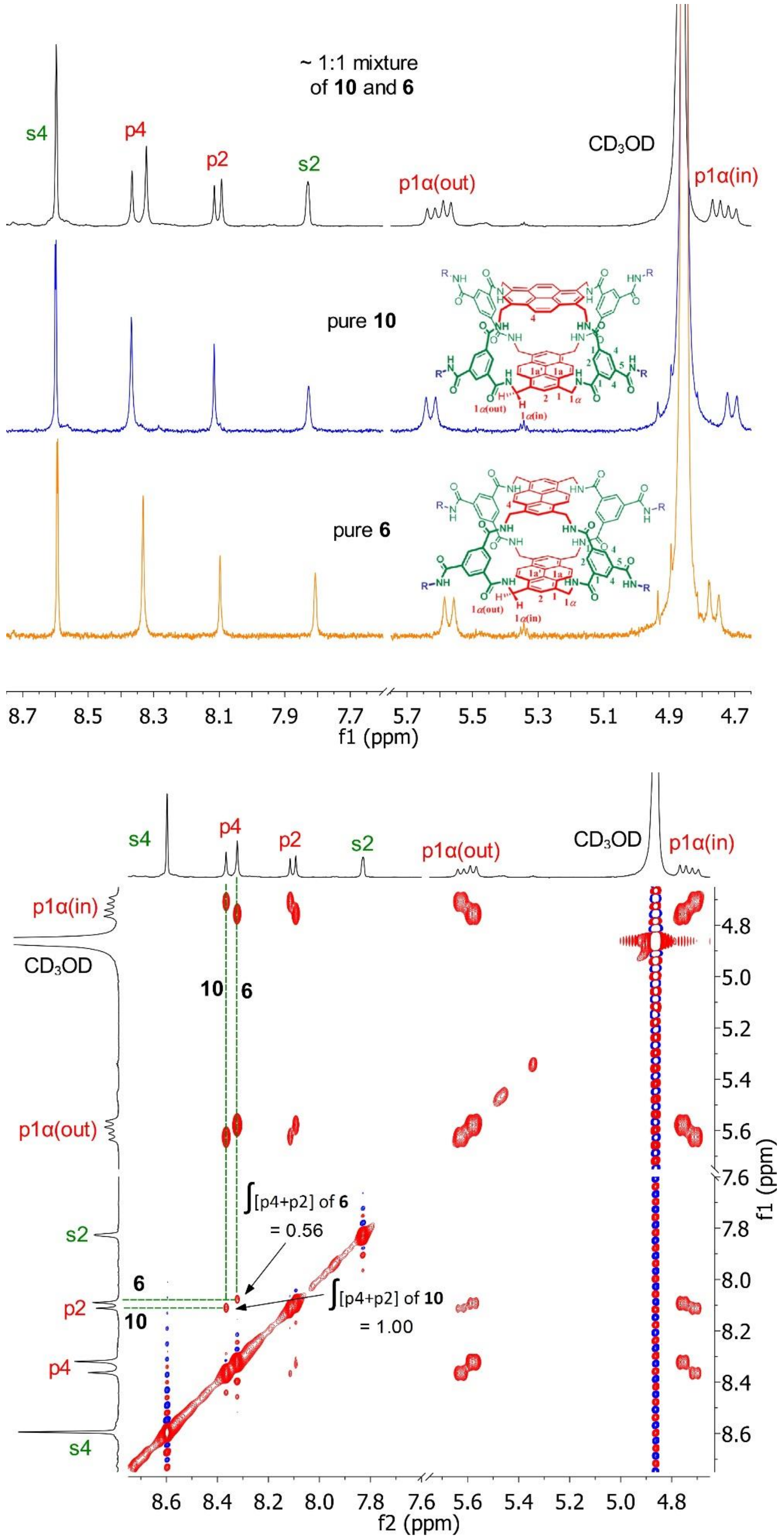

Figure S13. Top: stacked ^{1}H NMR spectra of **10**, **6** and a ~1:1 mixture of both. Bottom: {^{1}H-^{1}H} NOESY of the ~1:1 mixture with integrals of the p2/p4 cross peaks for **10** and **6**. The p2/p4 integral for **10** is about twice that for **6**, despite the former being slightly less concentrated. This supports the assignment, as the distance between p2 and p4 on different pyrene units is expected to be smaller for **10** than for **6**. Monte Carlo Molecular Mechanics conformational searches on **10** and **6** (MMFF force field) yielded global energy minima in which the average p2/p4 distances (considering all possible combinations) were 9.59 and 9.30 Å for **6** and **10** respectively. Spectra were recorded at 500 MHz in CD_3OD.

Water-soluble receptors 5 and 9

The protected receptor **10** or **6** (5 mg, 0.71 µmol) was dissolved in DCM (2 mL) and the solution cooled to 0 °C, before the addition of TFA (1 mL) in a dropwise manner. The solution was then warmed to RT and stirred for 24 hours, before the volatiles were removed under reduced pressure. The product was then suspended in water (2 mL) before freeze drying to give a pale-yellow solid. The solid was suspended in H_2O (2 mL) and the pH adjusted to 7.0 by addition of sodium hydroxide. The resulting solution was then filtered (0.45 µm syringe filter) and freeze-dried, affording the deprotected receptor (4.1 mg, 0.71 µmol, 100%) as a pale yellow solid.

Staggered receptor **9**:

^{1}H NMR (500 MHz, D_2O, 358 K) δ 2.51 – 2.74 (m, 72H, d7), 2.74 – 2.92 (m, 96H, d3, d8), 2.93 – 3.08 (m, 24H, d2), 5.52 (d, *J* = 14.8 Hz, 8H, p1α(in)), 6.13 (d, *J* = 14.5 Hz, 8H, p1α(out)), 8.51 (s, 4H, s2), 8.82 (s, 4H, p2), 8.97 (s, 8H, p4), 9.04 (s, 8H, s4); ^{13}C NMR (126 MHz, D_2O, 343 K) δ 31.3 (d7), 31.5 (d2), 31.5 (d3), 31.6 (d8), 42.6 (1α), 124.0 (p4), 127.2 (s2), 130.2 (s4), 132.5 (p2).

Eclipsed receptor **5**:

^{1}H NMR (500 MHz, D_2O, 358 K) δ 2.58 – 2.66 (m, 72H, d7), 2.78 – 2.85 (m, 24H, d3), 2.85 – 2.94 (m, 72H, d8), 2.97 – 3.06 (m, 24H, d2), 5.46 (d, *J* = 14.3 Hz, 8H, p1α(in)), 6.22 (d, *J* = 14.3 Hz, 8H, p1α(out)), 8.40 (s, 4H, p2), 8.86 (s, 4H, s2), 8.92 (s, 8H, p4), 9.09 (s, 8H, s4); ^{13}C NMR (126 MHz, D_2O, 343 K) δ 30.7 (d7), 30.8 (d8), 31.1 (d3), 31.4 (d2), 42.8 (1α), 124.3 (p4), 127.8 (s2), 130.0 (s4), 132.7 (p2).

The above NMR spectra were obtained at 358 K because spectra at lower temperatures were broadened and did not reflect the symmetry of the cage structures. For details of the NMR studies on **5** and **9** see Section 2 below.

Glycopeptide 3

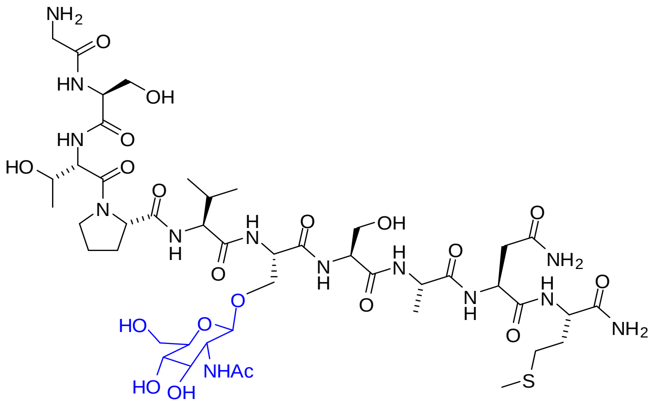

H_2N-GSTPVS (OGlcNAc) SANM-CONH $_2$ **3**

Glycopeptide **3** was prepared employing *N*-α –Fmoc-Ser-(AcO_3- β-D-*O*-GlcNAc) as a building block. Glycopeptide was prepared using MW-SPPS on Rink Amide AM LL Resin (0.1 mmol) using an automated CEM-Liberty instrument equipped with a UV-detector and a CEM-Discover SPS instrument. The first four amino acids, Ser-Ala-As*N*-Met were coupled on the peptide synthesizer using a standard protocol followed by manual coupling of glycosylated amino acid. Glycosylated amino acid, *N*-α –Fmoc-Ser-(AcO_3- β-D-*O*-GlcNAc) (0.2 mmol, 131 mg), with *O*-(7- azabenzotriazol-1-yl)-*N*,*N*,*N'*,*N'*-tetramethyl- uronium hexafluorophosphate (HATU; 0.2 mmol, 76 mg), 1-hydroxy-7-azabenzotriazole (HOAt; 0.2 mmol, 27 mg) and diisopropylethylamine (DIPEA; 0.4 mmol, 70 μL) were premixed in DMF (3 mL) for 2 min and were added to the resin. The manual microwave-irradiated coupling reaction was monitored by Kaiser test and was complete after 10 min. The peptide was then elongated under MW-SPPS conditions described above until final Glycine residue containing free amine group on *N*-terminus; the remaining steps performed manually. The resin was then treated with 60% hydrazine in methanol for 2 h. The resin was washed thoroughly with DMF (5 mL x 2), DCM (5 mL x 2) and MeOH (5 mL x 2) and then dried in *vacuo*. The resin was swelled in DCM (5 mL) for 1 h, after which it was treated with reagent B (TFA 88%, water 5%, phenol 5%, and TIS 2.5%; 10 mL) for 2 h. The resin was filtered and washed with neat TFA (2 mL). The filtrate was concentrated in *vacuo* approximately 1/3 of its original volume. The peptide was precipitated using diethyl ether (0 °C, 30 mL) and recovered by centrifugation at 3,000 rpm for 15 min. The crude glycopeptide was purified by HPLC on a Agilent semi preparative C-18 reversed phase column using a linear gradient of 0-100% solvent B in A over 40 min, and the appropriate fractions were lyophilized to afford pure glycopeptide. $C_{45}H_{77}N_{13}O_{20}S$, MALDI-ToF MS: observed [M+Na], 1174.3979Da; calculated [M+Na], 1174.5129 Da.

A solution of the glycopeptide in water was neutralised by additions of aqueous HCl, while monitoring with a pH meter. Once pH 7.0 was reached the solution was lyophilized to provide neutralised glycopeptide.

2. NMR studies on receptors 5 and 9

Eclipsed receptor 5

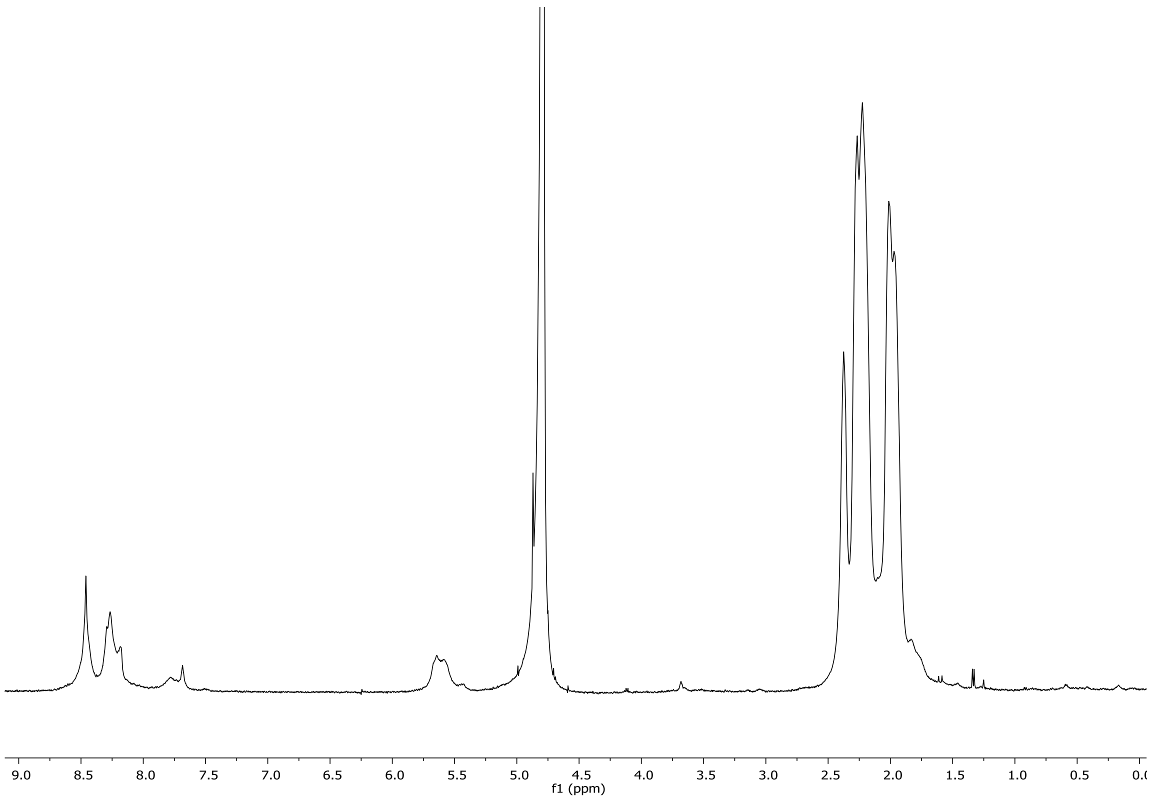

Figure S14. ^{1}H-NMR spectrum (500 MHz, 750 µM, D_2O) of eclipsed receptor **5** at 298 K

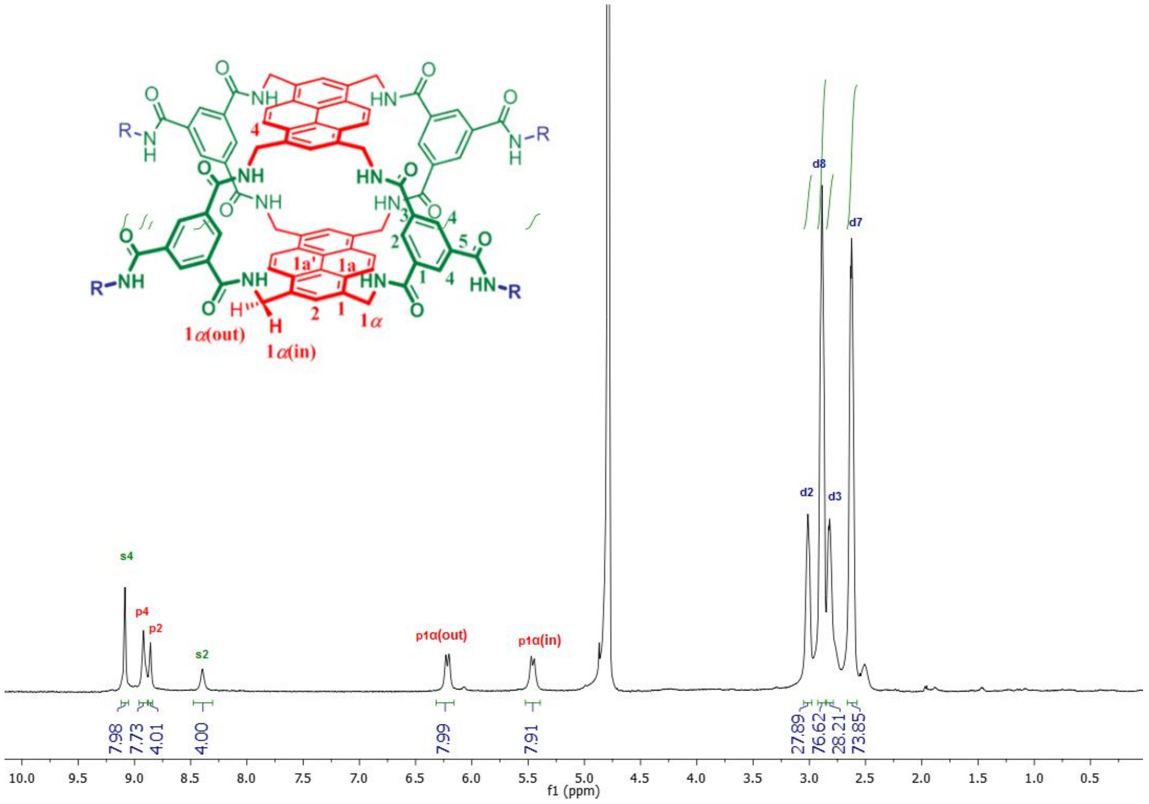

Figure S15. ^{1}H -NMR spectrum (500 MHz, 750 µM, D_2O) of eclipsed receptor **5** at 358 K.

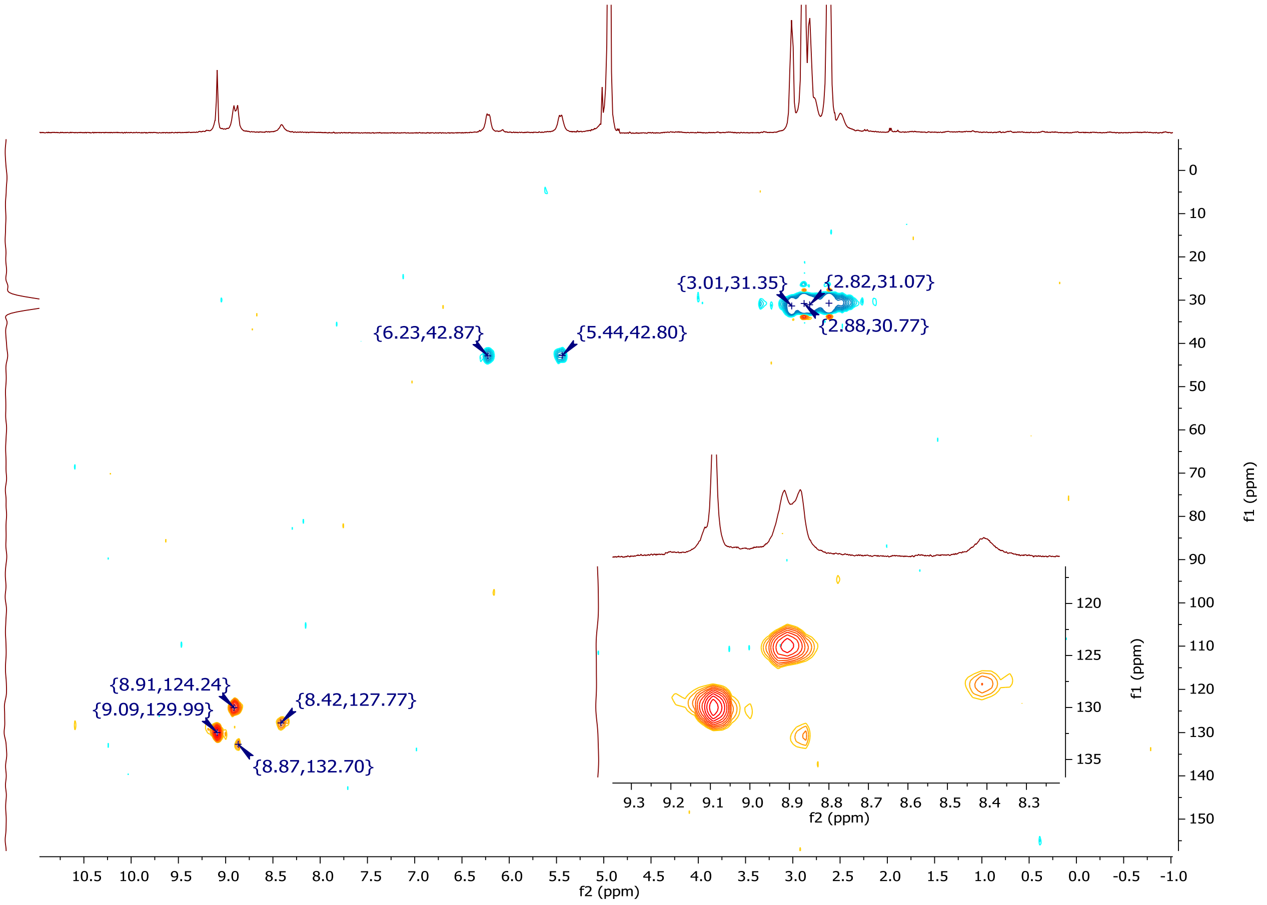

Figure S16. ^{1}H-^{13}C HSQC NMR spectrum (500 MHz, 750 µM, D_2O) of eclipsed receptor **5** at 343 K.

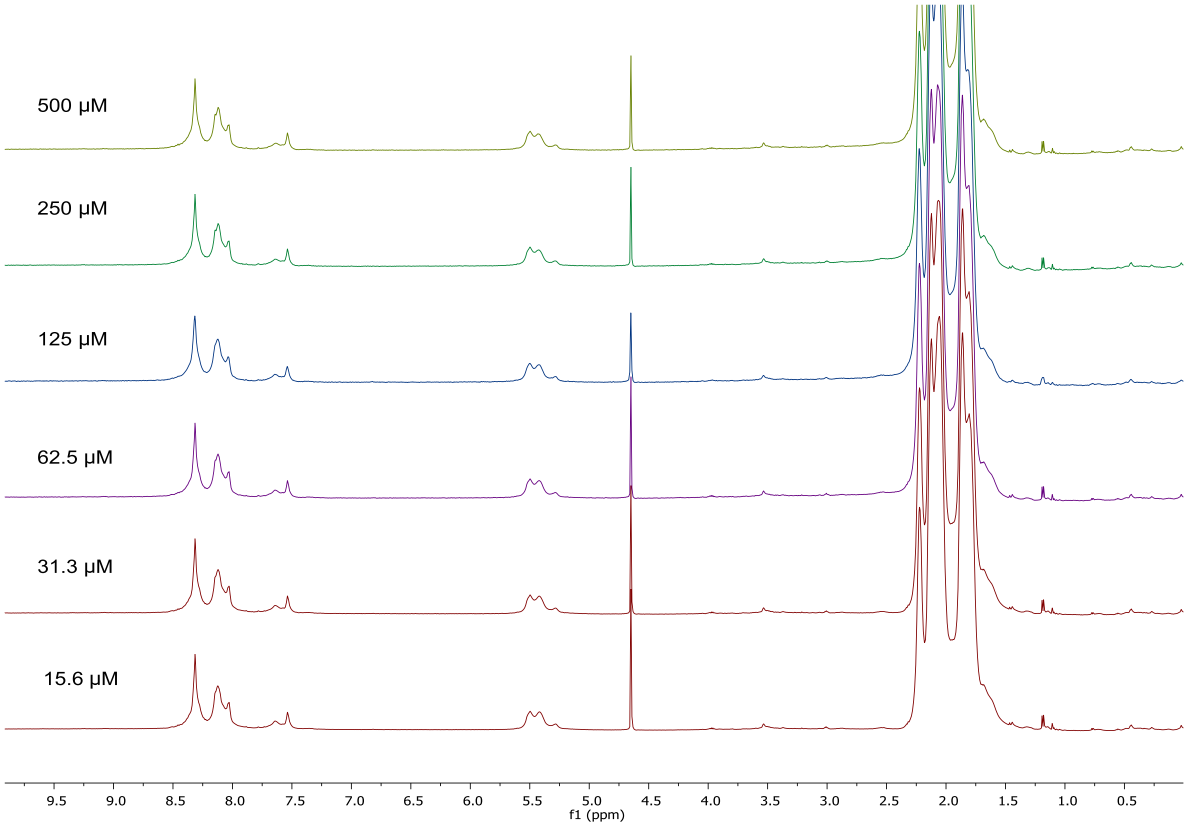

Figure S17. ^{1}H-NMR spectra of eclipsed receptor **5** at a range of concentrations, 15.5 µM to 500 µM in D_2O at 298 K.

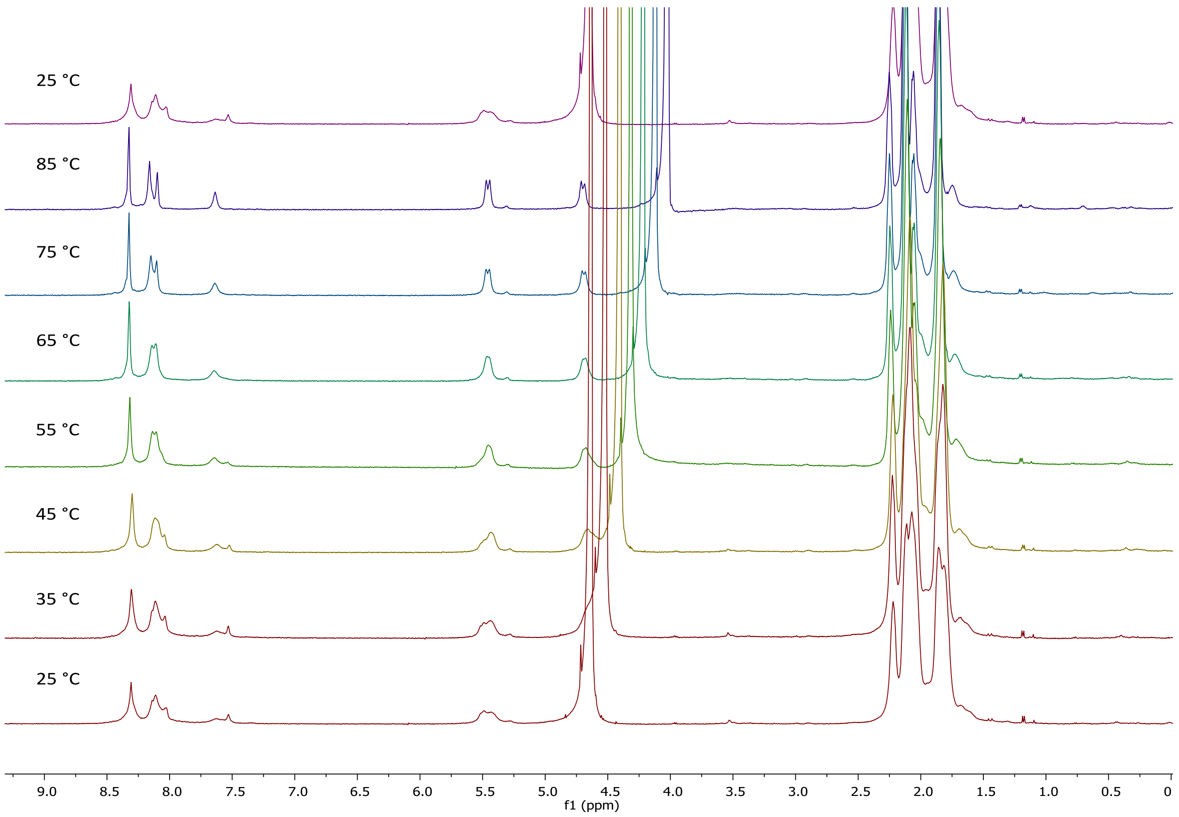

Figure S18. ^{1}H-NMR spectra of eclipsed receptor **5** at a range of temperatures, 25 °C to 85 °C in 10 °C steps before returning to 25 °C, 750 µM in D_2O. Peaks in the spectra are observed to sharpen and resolve as temperature increases, then return to their original state on cooling.

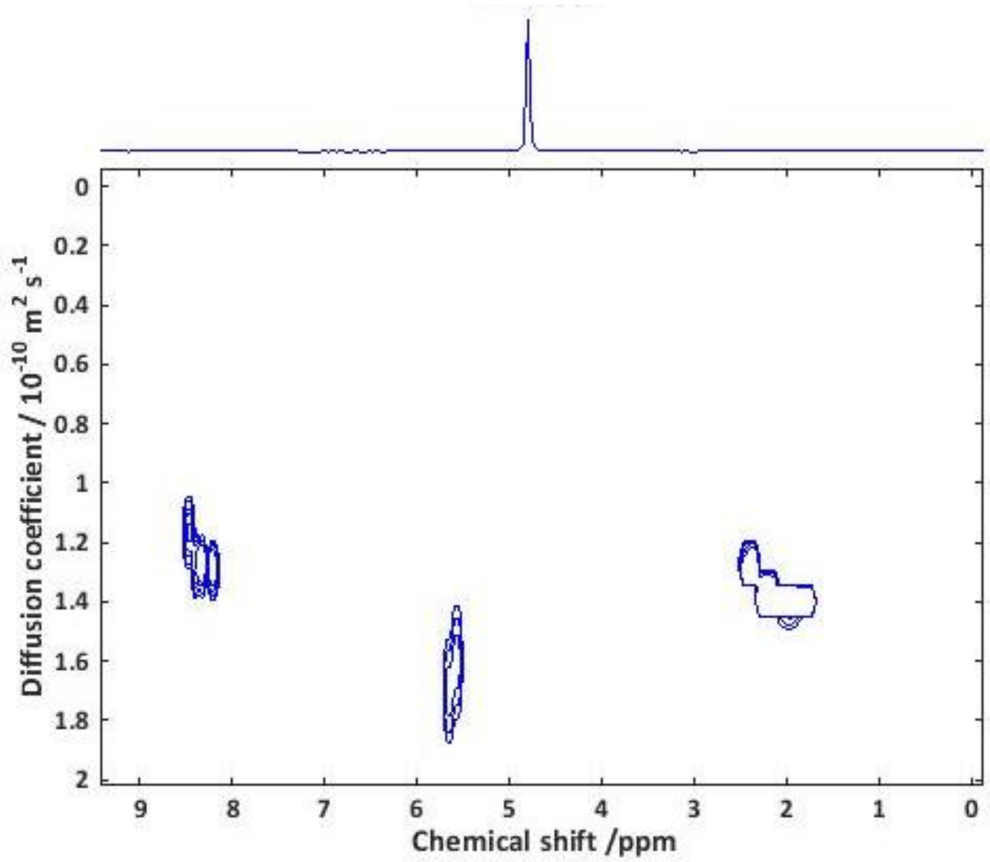

Figure S19. 2D DOSY NMR spectra of eclipsed receptor **5** at 500 µM in D_2O at 298 K. A diffusion coefficient of approx 1.05 x 10^{-10} to 1.90 x 10^{-10} m^2s^{-1}. Using the Stokes-Einstein equation[6] these values can be converted to the hydrodynamic radius, giving an estimated diameter between 3.78-2.09 nm.

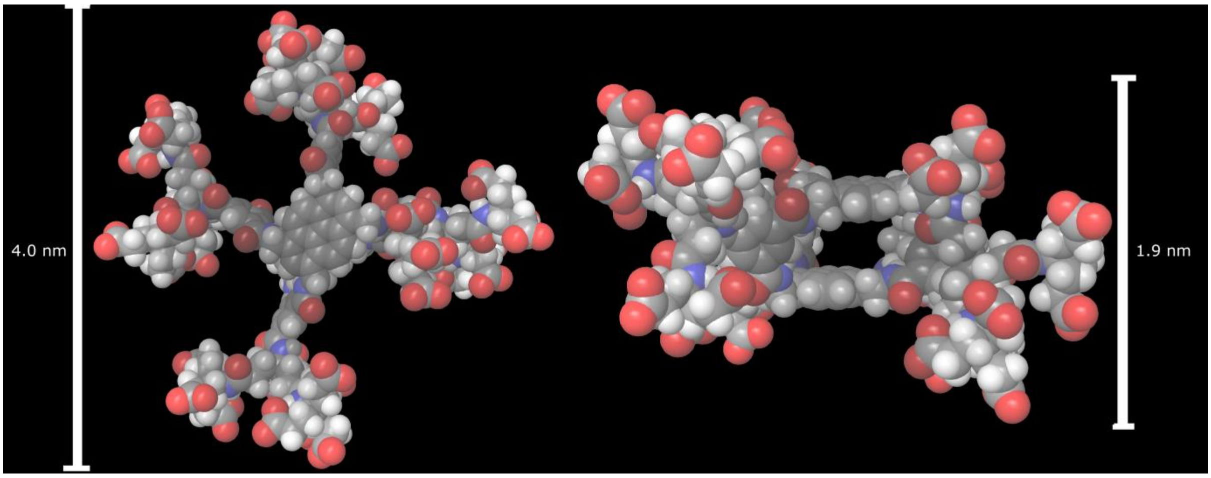

Figure S20. Dimensions of eclipsed receptor **5** as judged from by molecular model (Monte Carlo).

[6] The Stokes-Einstein equation relates diffusion coefficient in a solvent of a given viscosity to the size of a hard sphere diffusing in said solvent. Using this equation with the values obtained from DOSY NMR spectra, we can estimate the hydrodynamic radius of the molecules.

Stokes-Einstein equation:

$D = \frac{kT}{6\pi\eta R_H}$ or $R_H = \frac{kT}{6\pi\eta D}$

Where D = diffusion coefficient (from DOSY in m^2s^{-1}), k = Boltzmann's constant (1.38 x 10^{-23} m^2 kg s^{-2} K^{-1}), T = temperature (298 K), η = solvent viscosity (1.10 x 10^{-3} kg m^{-1} s^{-1} for D_2O), R_H = hydrodynamic Radius (m).

Staggered receptor 9

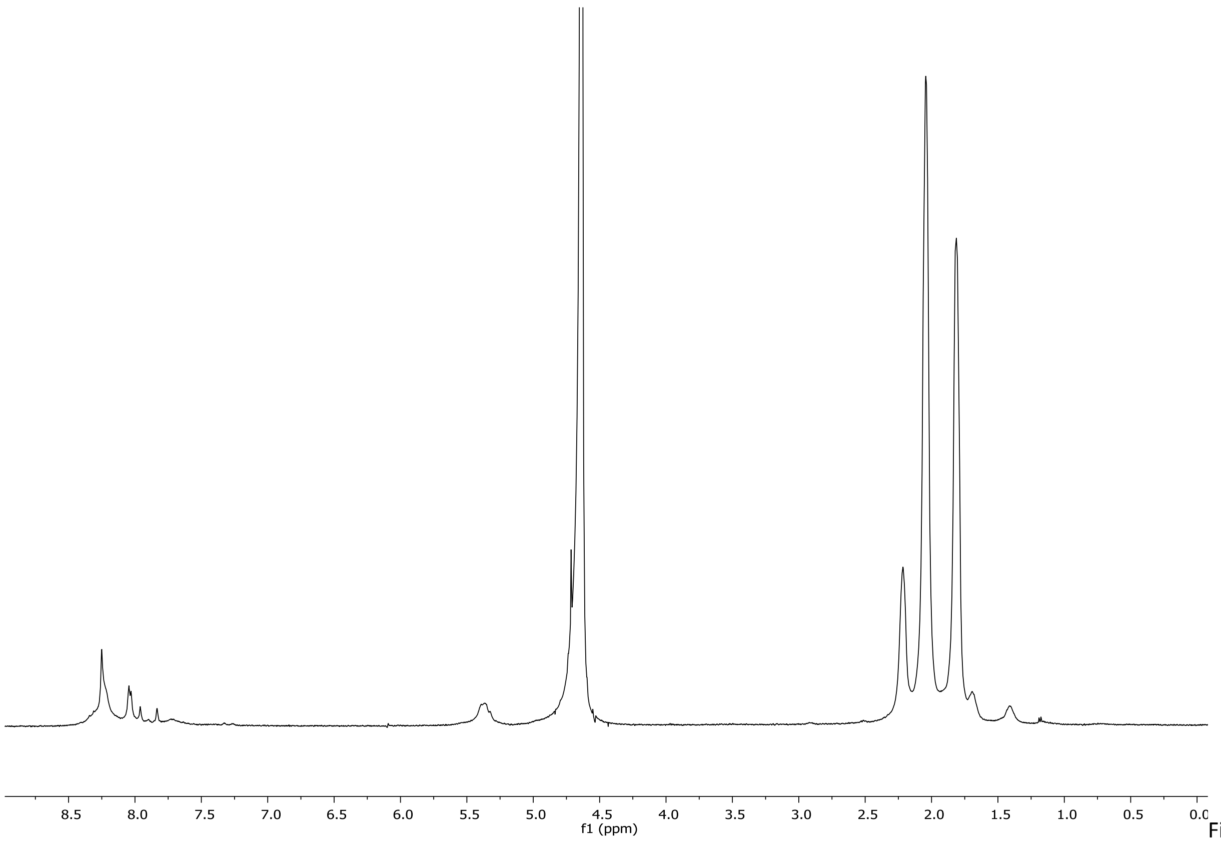

Figure S21. ^{1}H -NMR spectrum (500 MHz, 750 µM, D_2O) of staggered receptor **9** at 298 K.

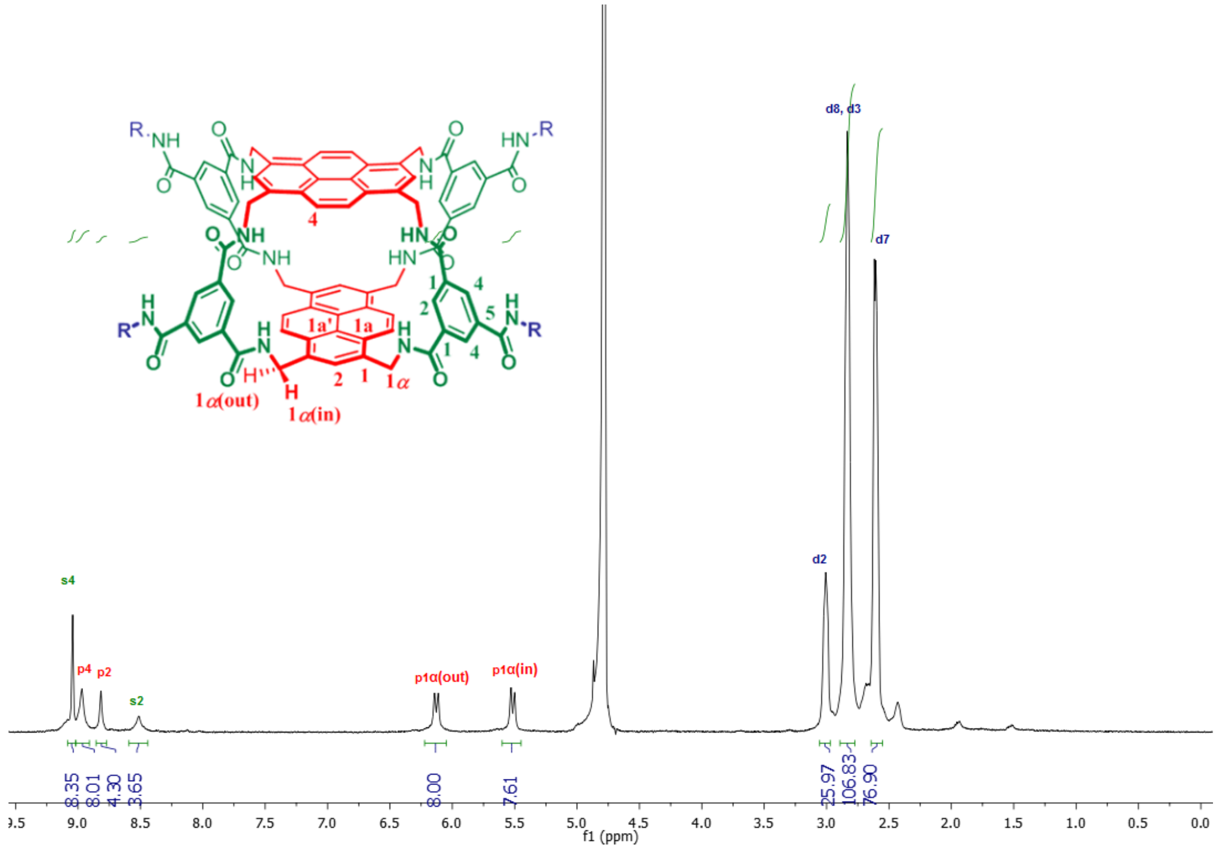

Figure S22. ^{1}H -NMR spectrum (500 MHz, 750 µM, D_2O) of staggered receptor **9** at 358 K.

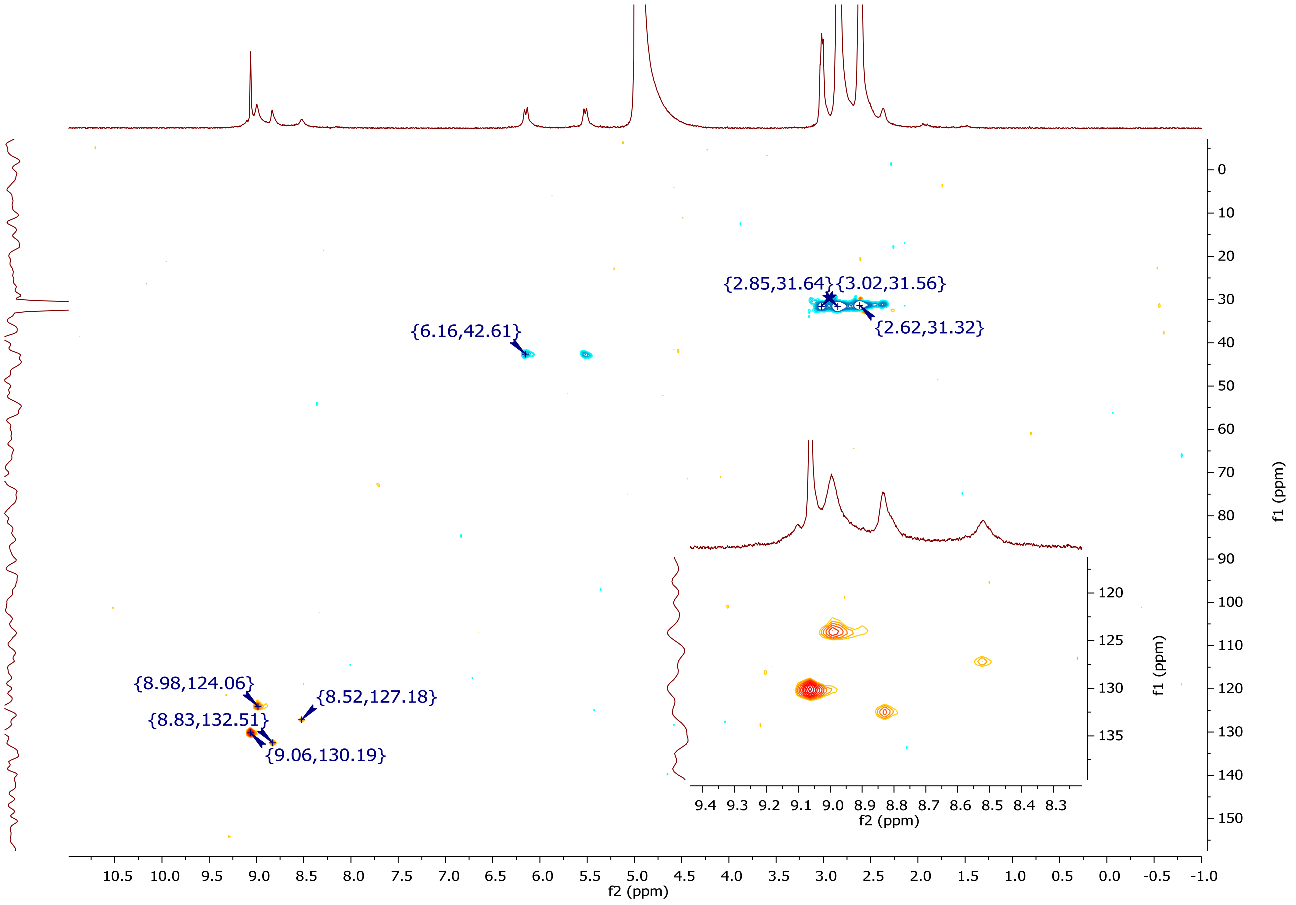

Figure S23. ^{1}H-^{13}C HSQC NMR spectrum (500 MHz, 750 µM, D_2O) of staggered receptor **9** at 343 K.

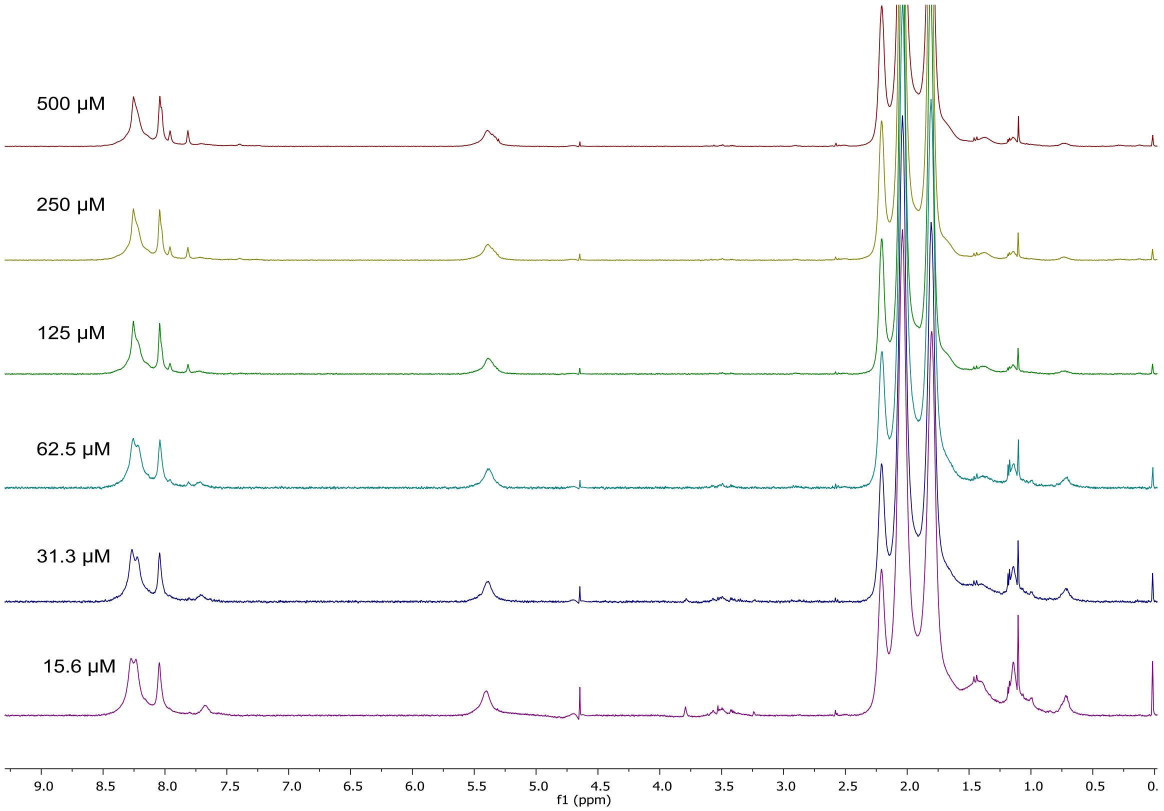

Figure S24. ^{1}H-NMR spectra of staggered receptor **9** at a range of concentrations, 15.6 μM to 500 μM in D_2O at 298 K.

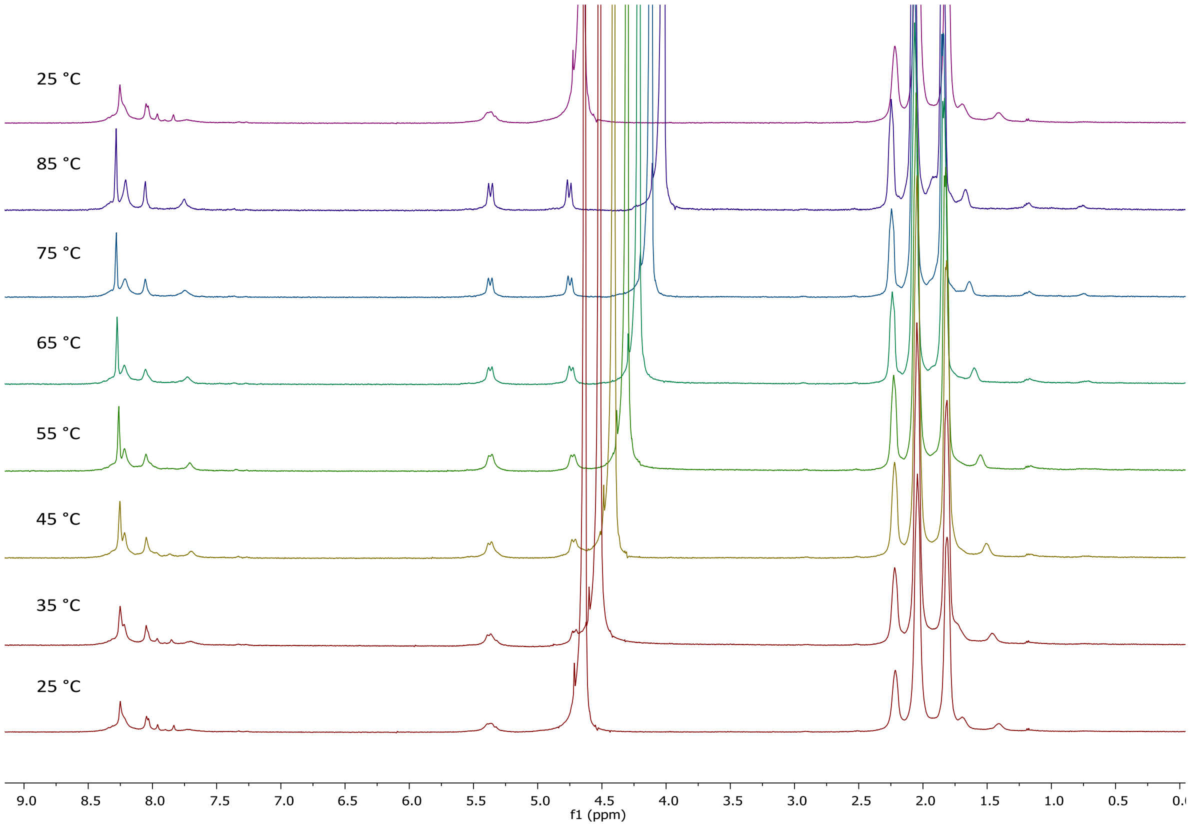

Figure S25. ^{1}H-NMR spectra of staggered receptor **9** at a range of temperatures, 25 °C to 85 °C in 10 °C steps before returning to 25 °C, 750 μM in D_2O. Peaks in the spectra are observed to sharpen and resolve as temperature increases, then return to their original state on cooling.

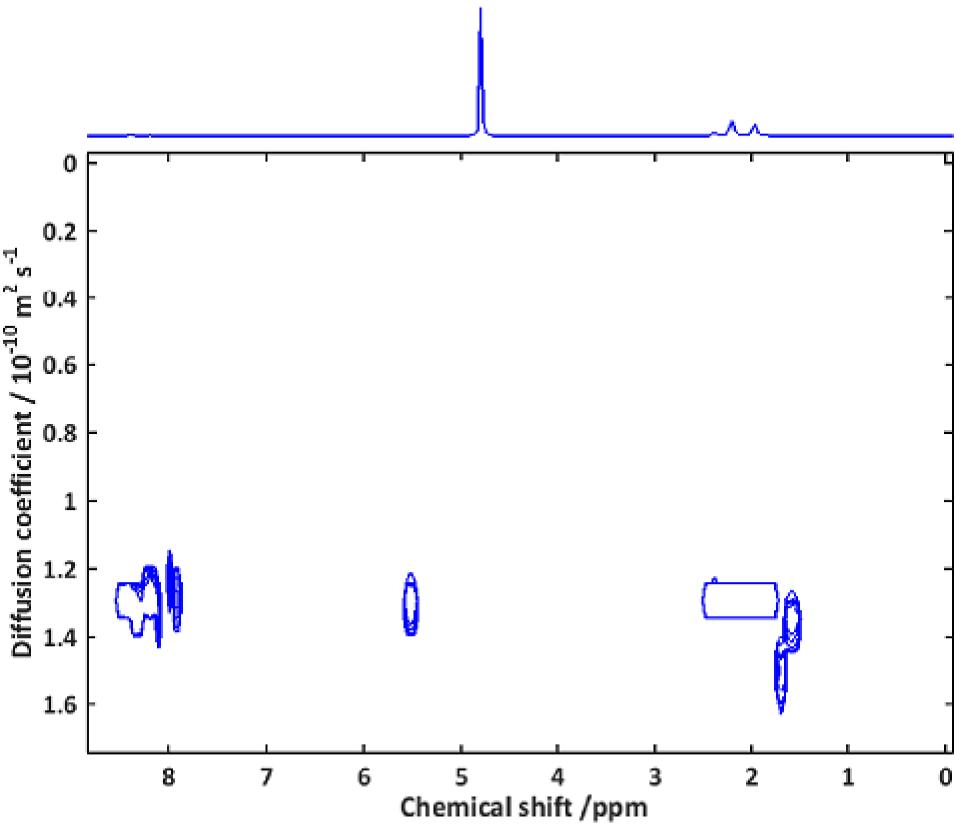

Figure S26. 2D DOSY NMR spectra of staggered receptor **9** at 500 µM in D_2O at 298 K. A diffusion coefficient of approx 1.15 x 10^{-10} to 1.55 x 10^{-10} m^2s^{-1} can be observed. Using the Stokes-Einstein equation these values can be converted to the hydrodynamic radius, giving an estimated diameter of 3.45-2.56 nm.

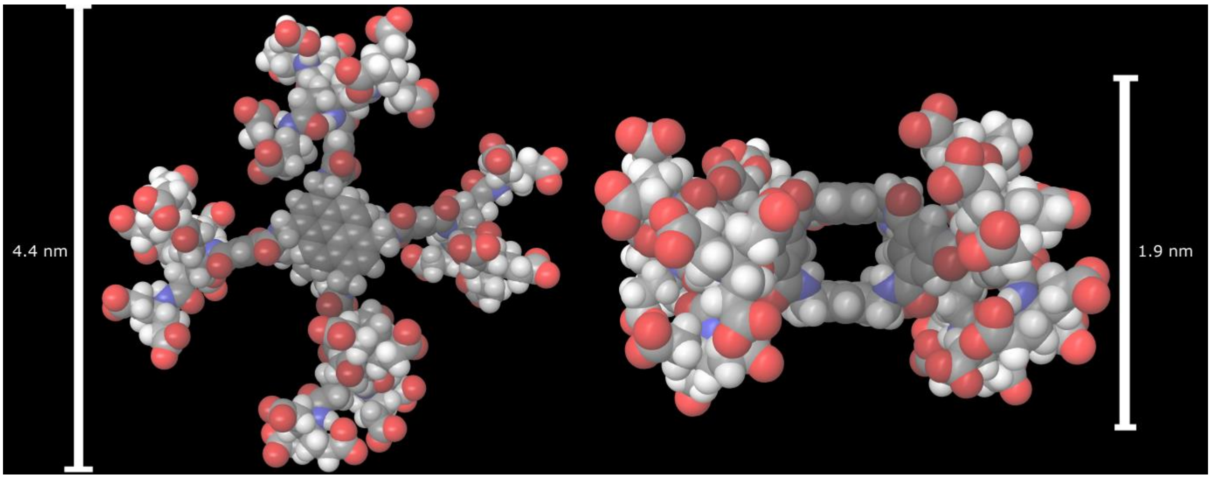

Figure S27. Dimensions of staggered receptor **9** as judged from by molecular model (Monte Carlo).

3. Binding Studies

^{1}H-NMR titrations. Solutions of saccharides in D_2O (99.9%), containing receptor at the concentration to be used in the experiment, were prepared and allowed to equilibrate overnight before use. Aliquots were then added to an NMR tube containing 400 μL of receptor solution. The receptor concentration was thereby held constant while the carbohydrate concentration was increased. The sample tube was shaken after each addition and ^{1}H-NMR spectra were acquired at 298 K.

If the receptor bound saccharide slower than the NMR sample rate ("slow exchange"), the K_a was determined by analysing the NMR integral of a peak assigned to the complex. The variable X was defined as the integral of an isolated resonance of the complex (typically in the aromatic region) divided by the integral of all the related resonances (typically the whole aromatic region). As X is proportional to fraction of host in the bound state, the change in X could be plotted as a function of the guest concentration to give a curve which could be fitted to a 1:1 binding model to yield the association constant K_a. Mathematically, the fitting process is essentially identical to that employed for binding with fast exchange, except that the integral of a peak due to the complex replaces the chemical shift of a peak due to bound + unbound receptor. The calculation was performed using a non-linear least squares curve-fitting programme implemented within Excel. The programme yields binding constants K_a and limiting X as output. K_a values are listed in Table 1 (main paper) and Table S1 below. An estimated error for K_a was obtained from individual data points by assuming the determined K_a and $X_{lim.}$. These errors are reported in Table S1 and are typically well below 5%.

Receptor **5** bound two substrates in slow exchange, the β-GlcNAc glycosides **2** and **3**. In both cases the limiting value of X was anomalously low and some signals due to receptor remained unchanged throughout the titration. However, on leaving **5** in the presence of either **2** or **3** for a long period (6 and 2 months respectively) the spectra evolved towards those attributed to the complex (see Figures S30 and S34). These results imply that a proportion of **5** is present in an inactive form, presumably a conformational isomer, which interconverts with the active form on a timescale of months. The titrations with **2** and **3** were performed within 24 hours, and showed no indication that significant conversion from inactive to active forms was occurring over this time scale (fits to the 1:1 binding model were good, with no sign of an upward drift in X towards the end of the experiment). This being the case, the presence of the inactive form should not affect the titration, which can be analysed to give the association constants of the two complexes. Although the concentration of active **5** was not known accurately, the analysis was insensitive to this parameter.[7]

In cases where the receptor bound saccharide at similar or faster rates than the NMR sample rate ("medium/fast exchange"), no attempt was made to estimate binding constants due to the complex nature

[7] This insensitivity results from the relatively low concentrations and binding constants. It was confirmed by varying the host concentration in the calculation by factors of 0.2 – 5, and showing that this did not significantly change fits or K_a values.

of the receptor spectra. Nonetheless, binding was clearly indicated in some cases, whereas in others there appeared to be little or no interaction between receptor and carbohydrate.

Spectra from the NMR titrations, and analysis curves where relevant, are included among Figures S28 – S67 below. Commentry on individual cases is given in the Figure captions.

Isothermal Titration Microcalorimetry (ITC). Isothermal Titration Microcalorimetry (ITC) experiments were carried out at 298 K on a MicroCal iTC200 microcalorimeter. Saccharide solutions were prepared in H_2O and allowed to equilibrate overnight, after which aliquots were added stepwise into a solution of the receptor in H_2O (0.25 or 0.5 mM) in the sample cell. The evolution of heat over the experiment is then measured as a function of time. Heats of dilution were measured by injecting the same saccharide solution into pure H_2O under identical conditions. The heats of dilution were then subtracted from the binding data, and the resulting peaks integrated to give the heat evolved by each addition. The total heat evolved (ΔH) was then plotted against the total concentration of guest. The data was then fitted to a 1:1 binding model using a non-linear least squares curve-fitting program implemented within Excel, to give a binding constant (K_a). Gibbs free energy of binding (ΔG) can then be derived from the binding constant (K_a), and the entropy of binding (ΔS) can be derived from ΔH and ΔG. The fitting procedure also yields errors in K_a as in the case of NMR described above. This method consistently produced more accurate fits than fitting the data to an S-curve, as in the MicroCal software (S-curves are typically not observed for binding constants below ~10^4 - 10^5 M^{-1}). It must be noted that, in the case of receptor **5**, the presence of inactive receptor should introduce errors to ΔH (and thus also ΔS). The K_a and derived ΔG values remain reliable, as discussed above for the ^{1}H NMR titrations.

ITC output and analysis curves are included among Figures S28 – S67. An overview of the binding data, including thermodynamic quantities and errors is given in Table S1 below.

Table S1. Summary of binding results for receptors **5** and **9** with carbohydrate substrates in aqueous solution at 298 K, including estimated errors derived from the fitting procedure and thermodynamic quantities from the ITC measurements.

Carbohydrate	Eclipsed receptor 5				
	NMR	ITC ($kJ.mol^{-1}$)			
	K_a (M^{-1})	K_a (M^{-1})	ΔG	ΔH[a]	TΔS[a]
**GlcNAc-β-OMe (2)**	2052 ± 1.7%	2,180 ± 6.6%	-19.06	-4.81	14.24
**Glc-β-OMe (15)**	-	1,438 ± 4.9%	-18.03	-2.16	15.86
**Glucose (16)**	-	121 ± 1.4%	-11.88	-6	5.89
**Glycopeptide 3**	66977 ± 3.8%		-	-	-

[a] Unreliable due to presence of inactive receptor.

Carbohydrate.	Staggered receptor 9				
	NMR	ITC ($kJ.mol^{-1}$)			
	K_a (M^{-1})	K_a (M^{-1})	ΔG	ΔH	TΔS
**GlcNAc-β-OMe (2)**	18179 ± 5.1%	16625 ± 3.3%	-24.1	-14.3	9.84
**GlcNAc-α-OMe (13)**	1549 ± 1.5%	1522 ± 5.3%	-18.2	-8.55	9.61
**GlcNAc (14)**	518 ± 1.6%	518 ± 4.2%	-15.5	-7.89	7.6
**Glc-β-OMe (15)**	1176 ± 2.2%	1,234 ± 2.4%	-17.65	-3.58	14.07
**Glucose (16)**		194 ± 0.6%	-13.06	-4.89	8.17

Eclipsed receptor 5

Methyl *N*-acetyl-β-D-glucosaminide (2)

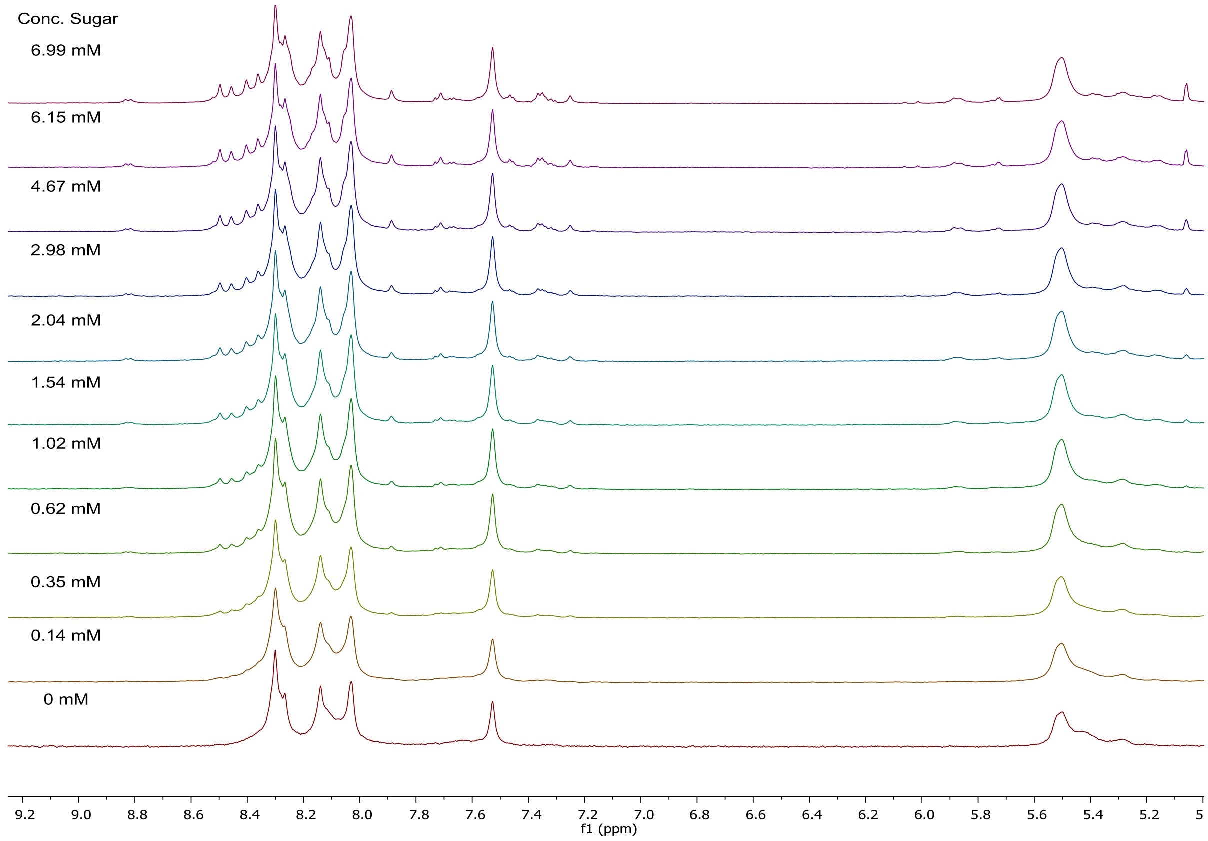

Figure S28. ^{1}H NMR binding study of eclipsed receptor **5** (0.15 mM) titrated with methyl *N*-acetyl-β-D-glucosaminide **2** (28.3 mM) in D_2O at 298 K. Spectra imply binding with slow exchange, allowing analysis of integrals to give K_a (see below).

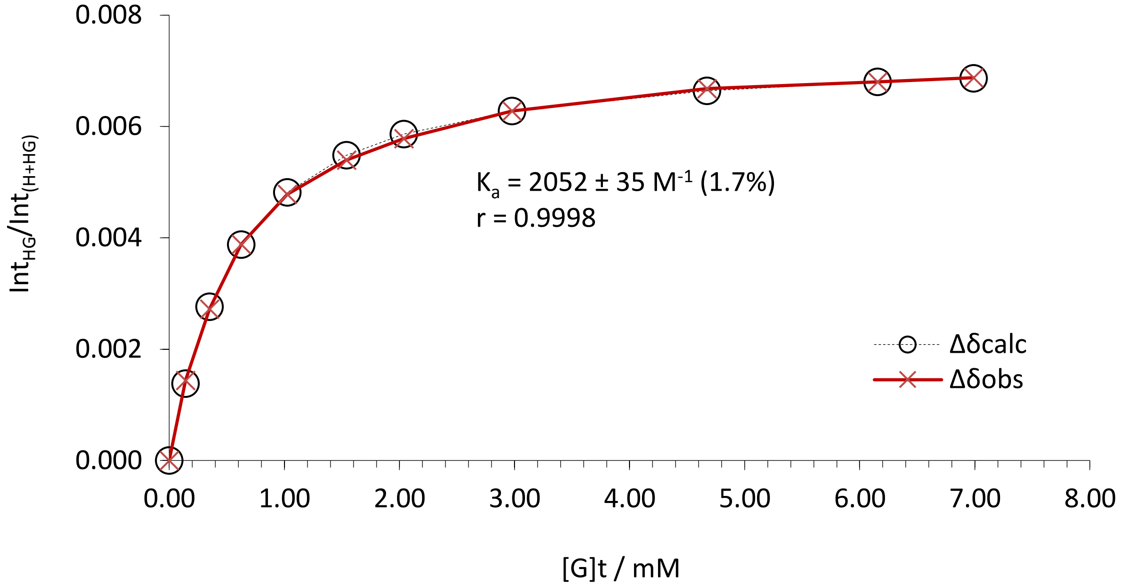

Figure S29. ^{1}H NMR binding study of eclipsed receptor **5** (0.15 mM) titrated with methyl *N*-acetyl-β-D-glucosaminide (**2**) (28.3 mM) in D_2O at 298 K. Plot of the observed integral (peak at 7.25 ppm integrated against 9.50-6.85 ppm) versus guest concentration (mM). The calculated values for the integrals are overlaid, predicting K_a = 2052 ± 35 M^{-1} (1.7%).

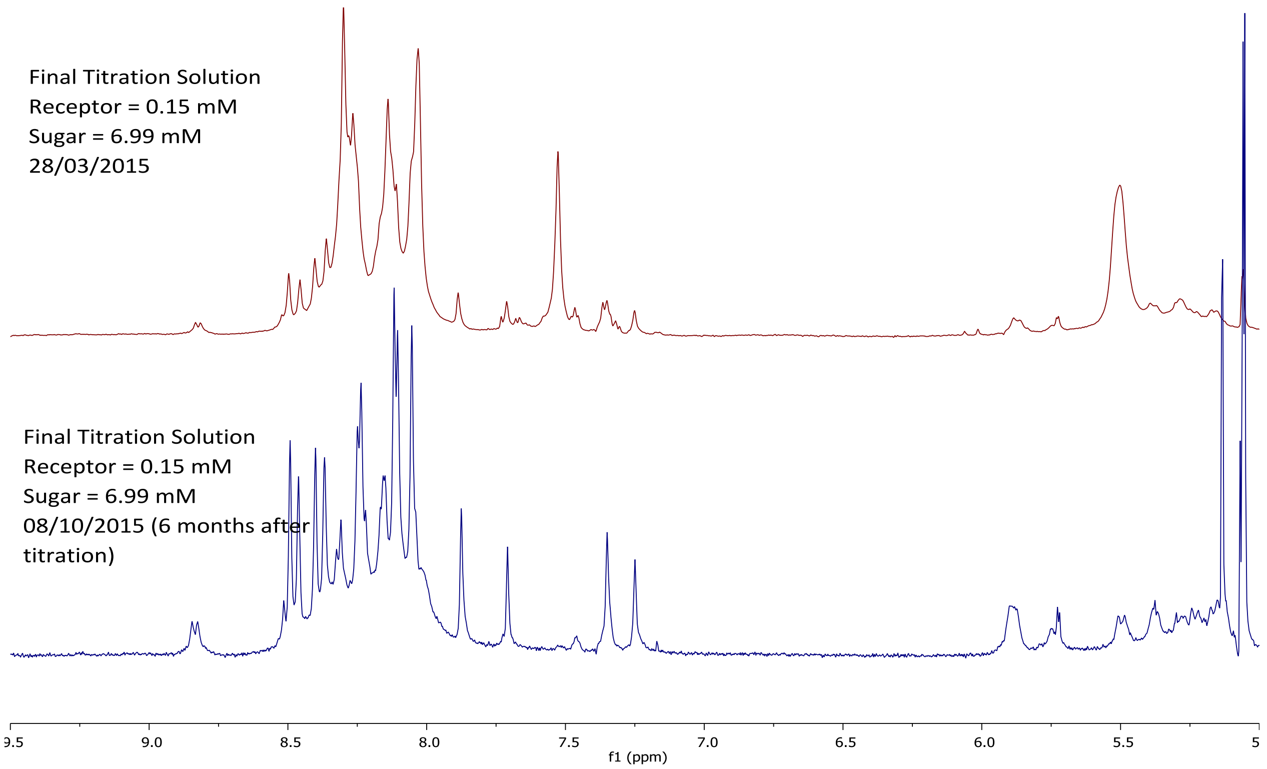

Figure S30. ^{1}H NMR spectra of the final solution resulting from the binding study of eclipsed receptor **5** (0.15 mM) titrated with methyl *N*-acetyl-β-D-glucosaminide (**2**) (28.3 mM) in D_2O at 298 K. Upper spectra (red) shows the solution on the day of titration and the lower spectra (blue) shows the same solution 6 months later. This shows that a part of the receptor exists in an inactive form, probably a conformer, which slowly converts to the complex.

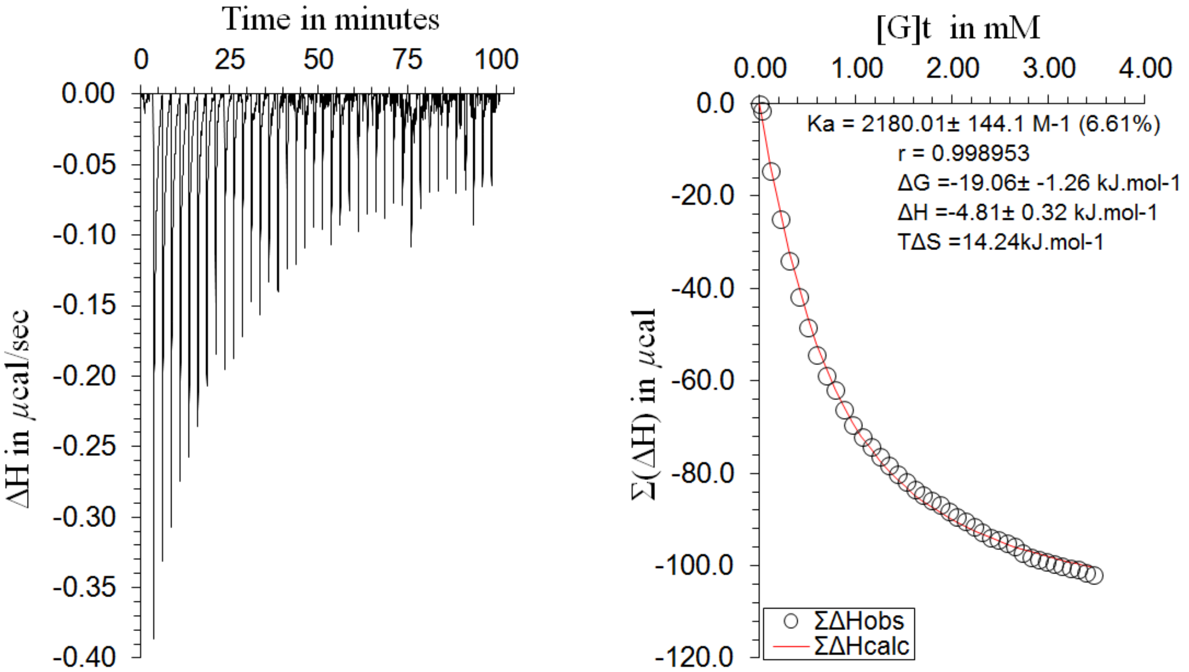

Figure S31. ITC binding study of eclipsed receptor **5** (0.50 mM) titrated with methyl *N*-acetyl-β-D-glucosaminide (**2**) (10 mM) in H_2O. The sum of heat evolution (µcal) was plotted as a function of the concentration of carbohydrate (mM) and fitted to a 1:1 binding model indicating K_a = 2180 ± 144 M^{-1} (6.6%) and r = 0.9990 (thermodynamic data also given in the figure).

Glycopeptide (3)

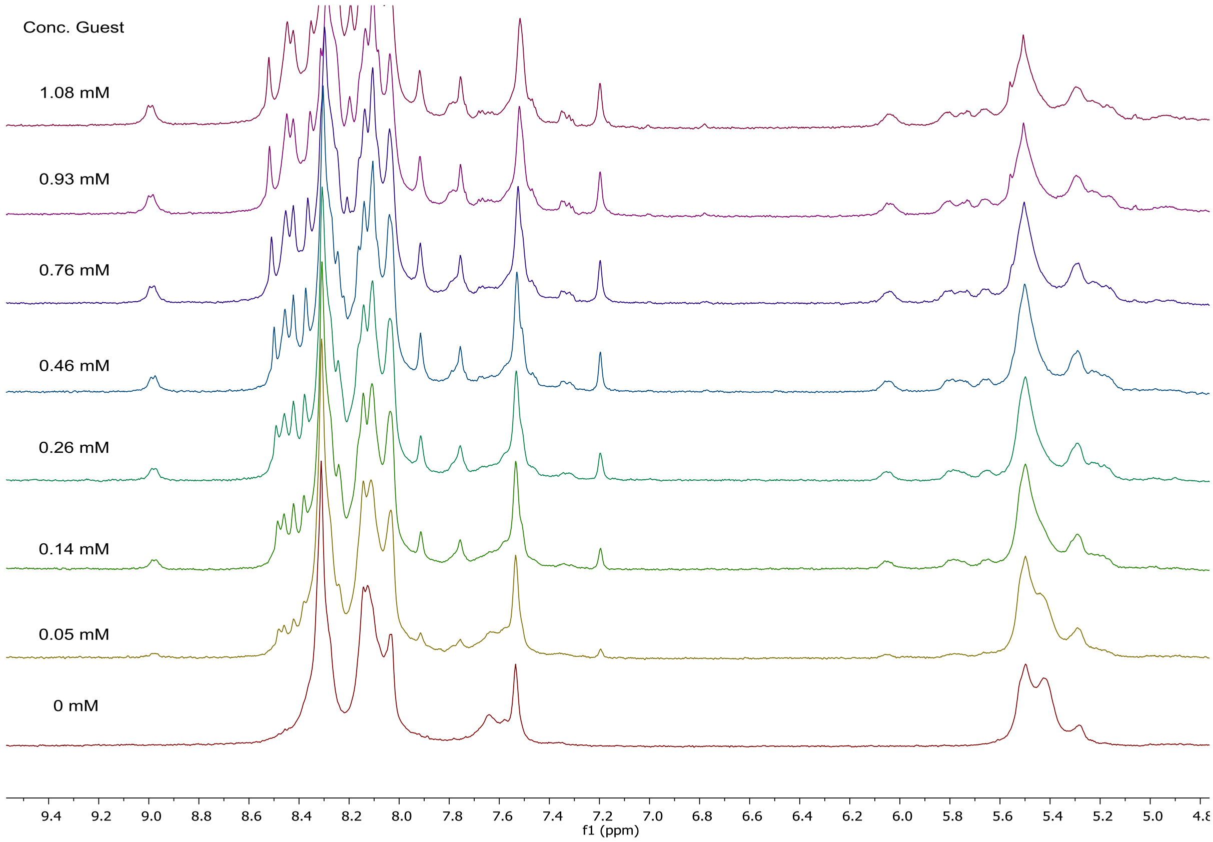

Figure S32. Partial ^{1}H NMR spectra from the binding study of eclipsed receptor **5** (0.25 mM) titrated with glycopeptide **3** (3.78 mM) in D_2O at 298 K. Spectra imply binding with slow exchange, allowing analysis of integrals to give K_a (see below).

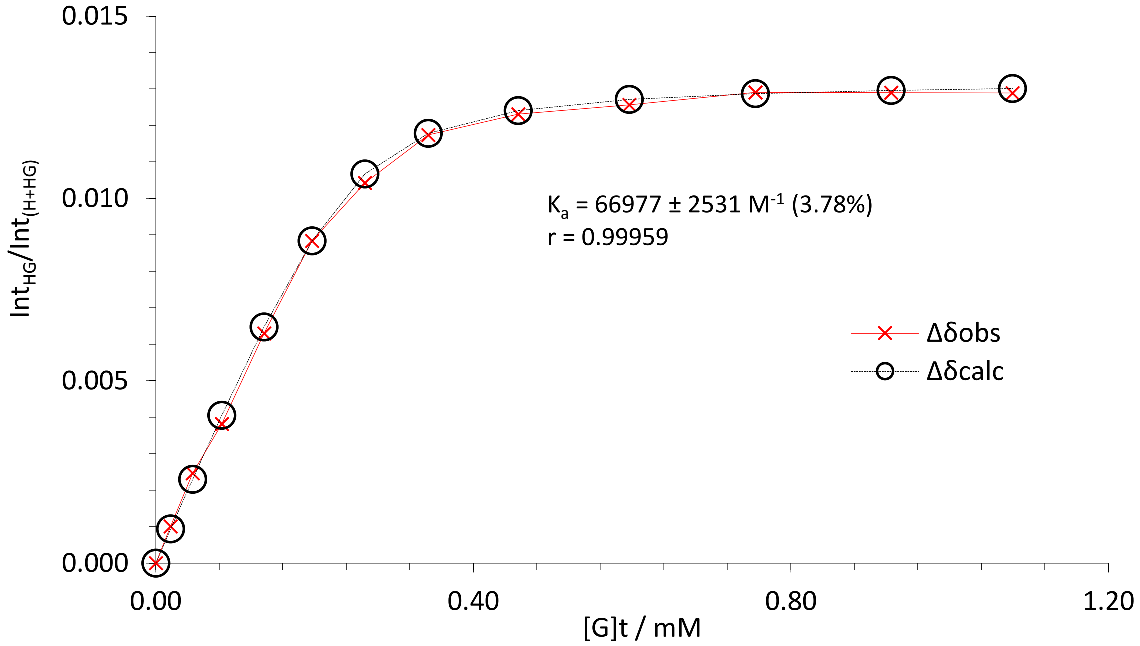

Figure S33. ^{1}H NMR binding study of eclipsed receptor **5** (0.25 mM) titrated with glycopeptide **3** (3.78 mM) in D_2O at 298 K. Plot of the observed integral (peak at 7.20 ppm integrated against 9.50-7.00 ppm) versus guest concentration (mM). The calculated values for the integrals are overlaid, predicting K_a = 66977 ± 2531 M-1 (3.78%). The points used in the slow exchange method of K_a calculation are labelled yellow.

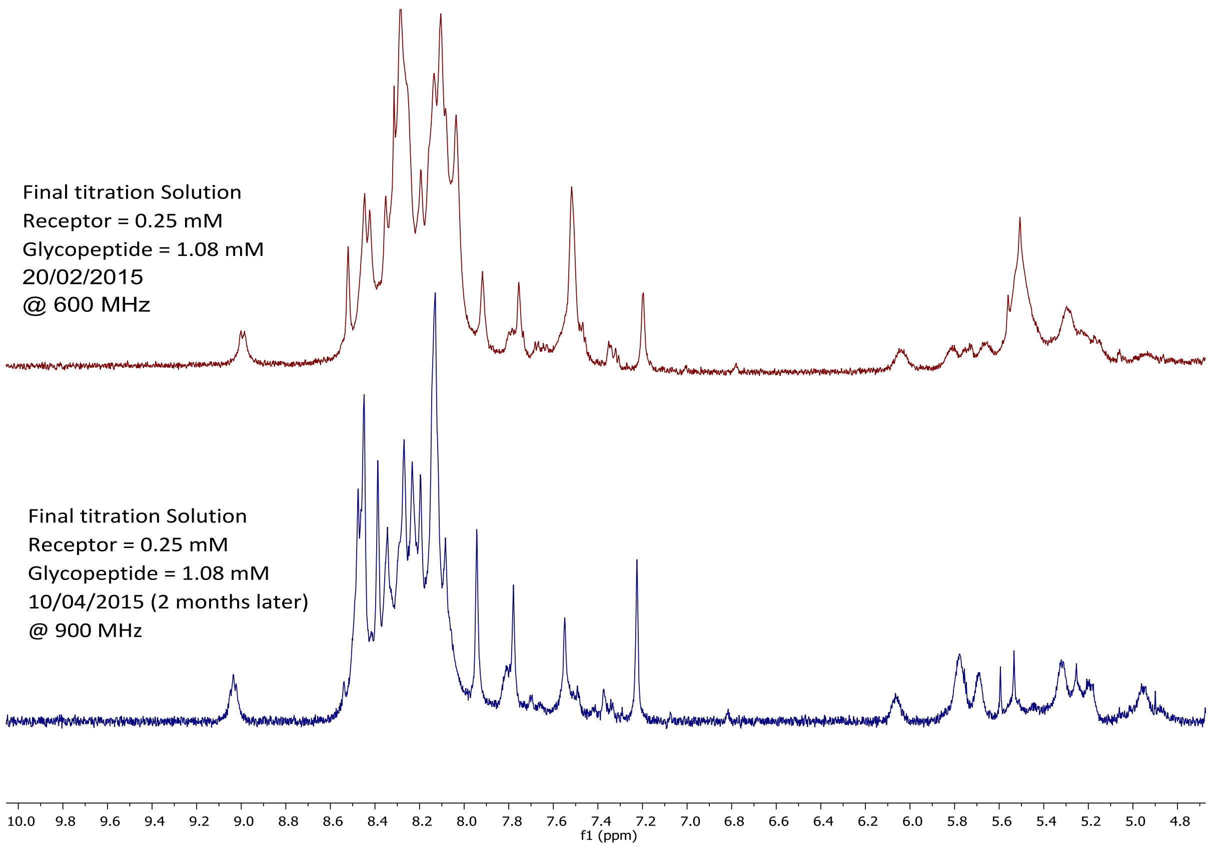

Figure S34. ^{1}H NMR spectra of the final solution resulting from the binding study of eclipsed receptor **5** (0.25 mM) titrated with glycopeptide **3** (3.78 mM) in D_2O at 298 K. Upper spectra (red) shows the solution on the day of titration and the lower spectra (blue) shows the solution 2 months later. This shows that part of the receptor exists in an inactive form, probably a conformer, which slowly converts to the complex.

Methyl *N*-acetyl-α-D-glucosaminide (13)

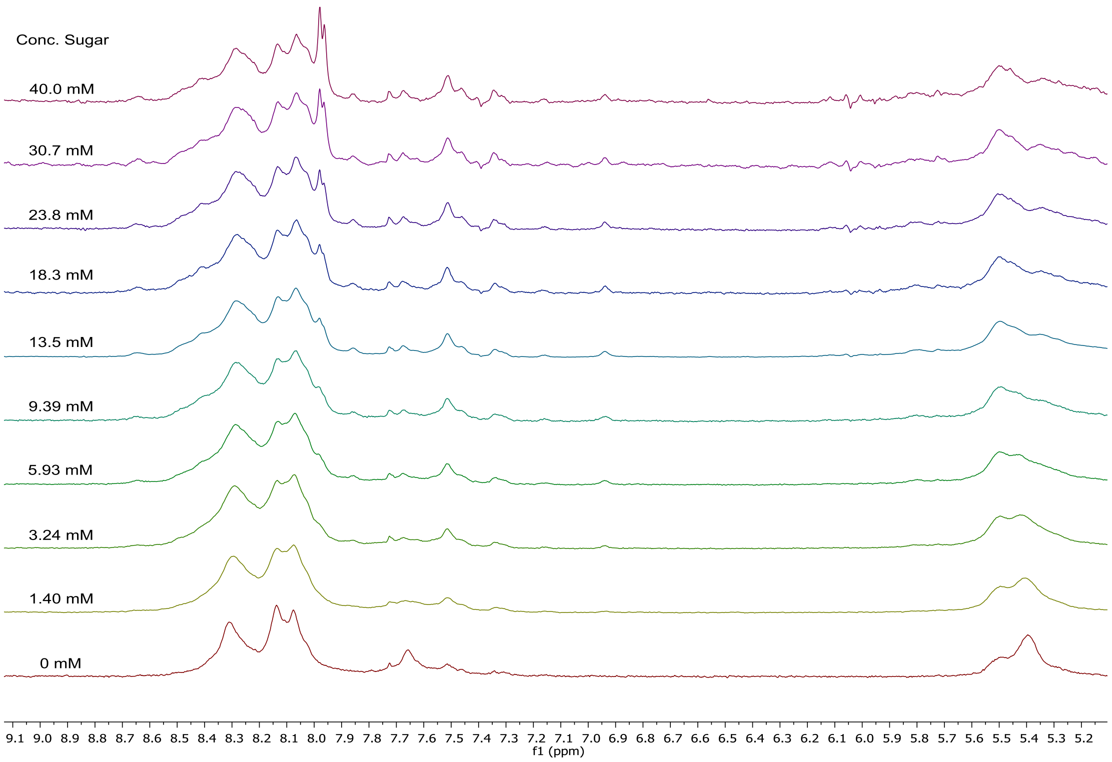

Figure S35. Partial ^{1}H NMR spectra from the binding study of eclipsed receptor **5** (0.15 mM) titrated with methyl *N*-acetyl-α-D-glucosaminide (**13**) (188 mM) in D_2O at 298 K. Spectra are difficult to interpret, although there are clear indications of binding.

N-Acetyl-D-glucosamine (14)

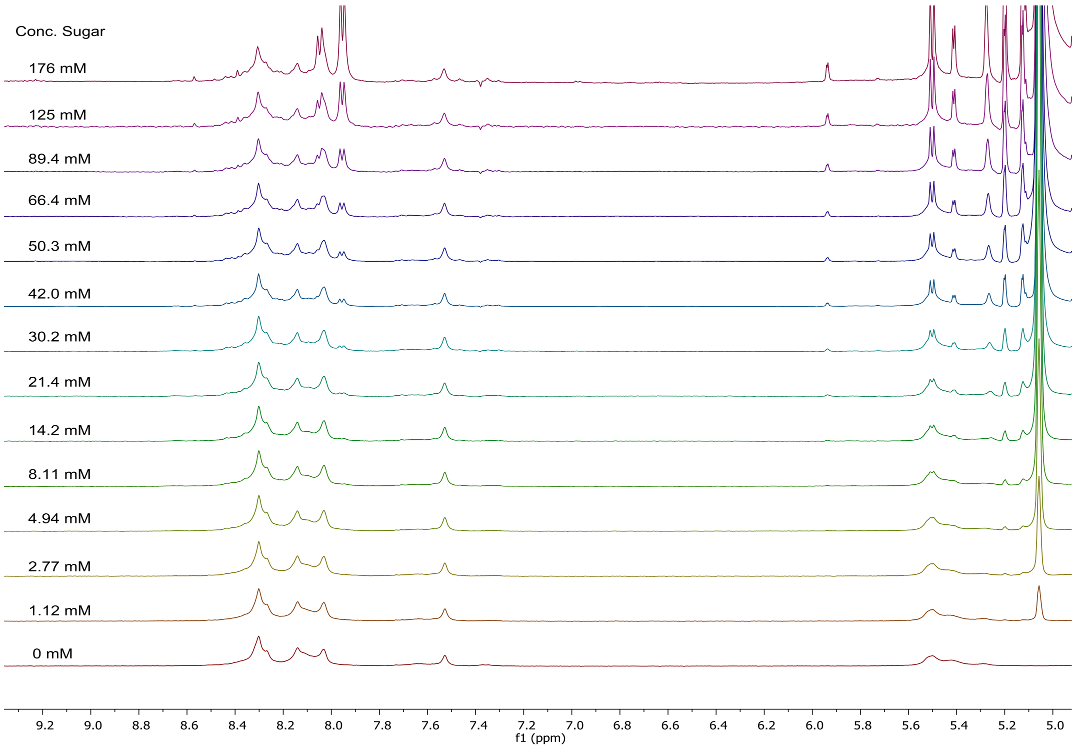

Figure S36. ^{1}H NMR binding study of eclipsed receptor **5** (0.15 mM) titrated with *N*-acetyl-β-D-glucosamine (**14**) (225 mM, then 867 mM) in D_2O at 298 K. New peaks around 8.0 p.p.m. are due to (**14**). No evidence for binding can be detected.

Methyl β-D-glucoside (15)

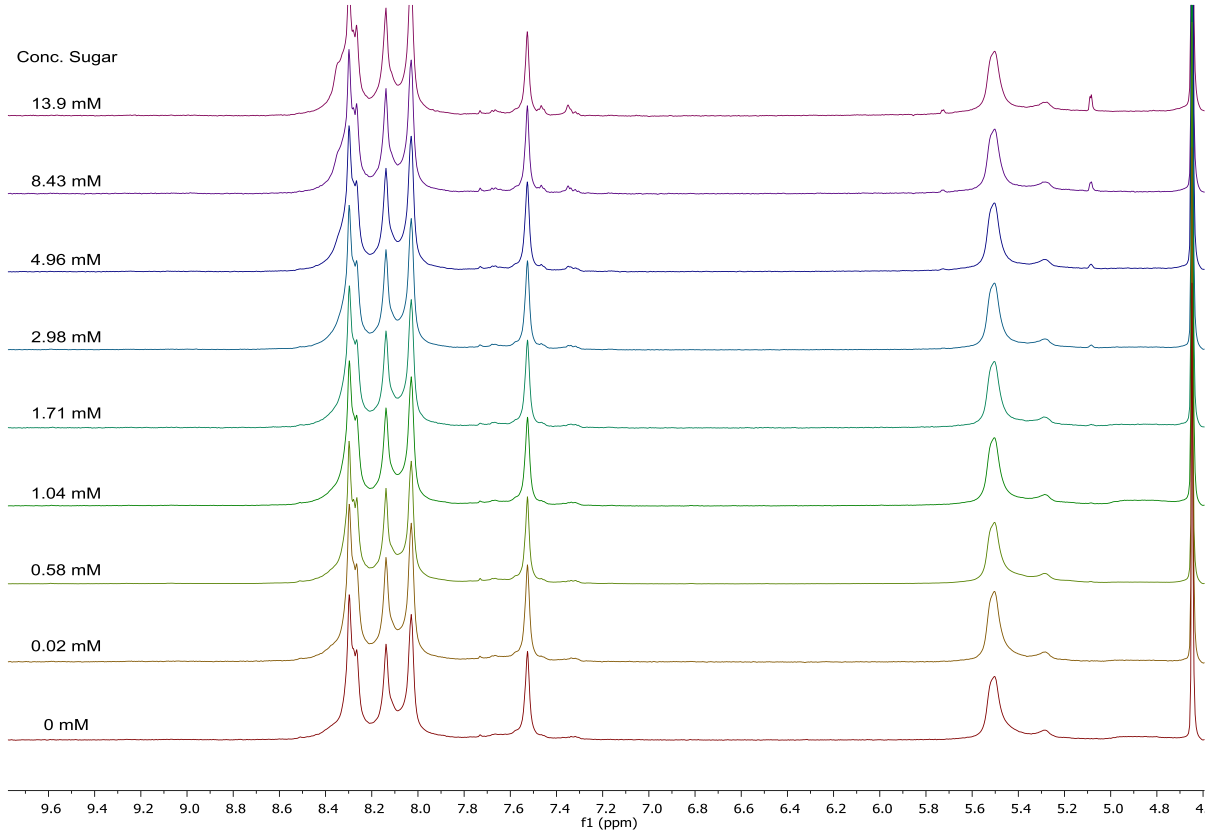

Figure S37. Partial ^{1}H NMR spectra from the titration of eclipsed receptor **5** (0.15 mM) with methyl-β-D-glucoside (**15**) (47 mM) in D_2O at 298 K. Spectra imply binding with medium/fast exchange, which could not be quantified from this study.

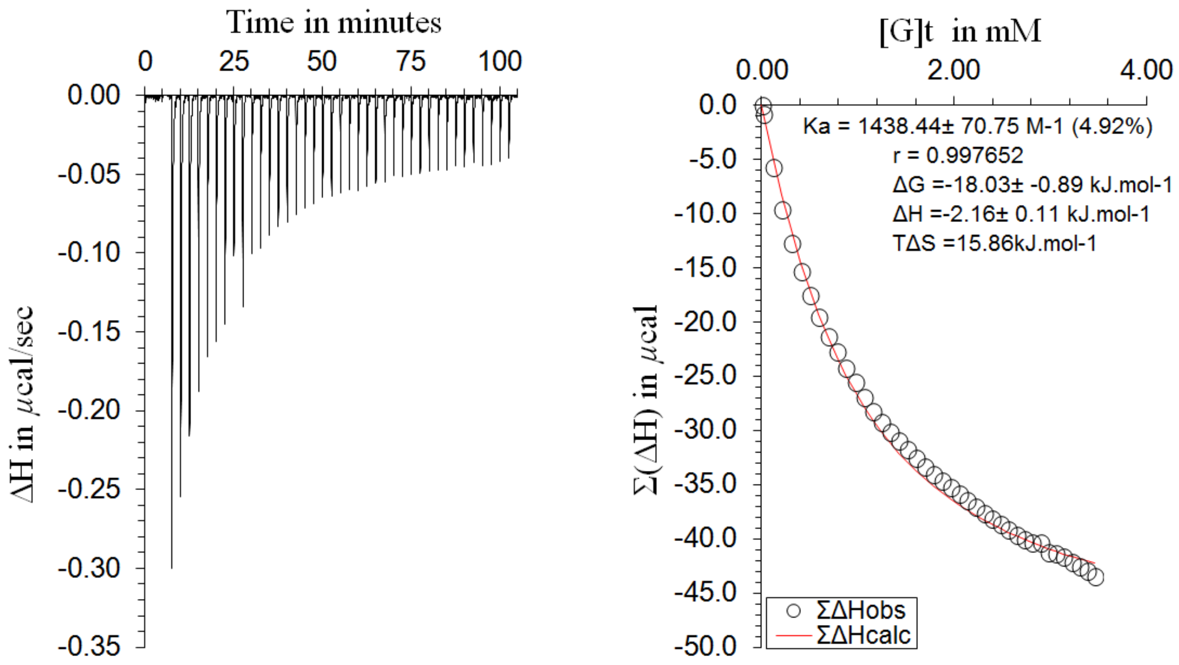

Figure S38. ITC binding study of eclipsed receptor **5** (0.50 mM) titrated with methyl-β-D-glucoside (**15**) (20 mM) in H_2O. The sum of heat evolution (µcal) was plotted as a function of the concentration of carbohydrate (mM) and fitted to a 1:1 binding model indicating K_a = 1438 ± 71 M^{-1} (4.9%) and r = 0.9977 (thermodynamic data also given in the figure).

D-Glucose (16)

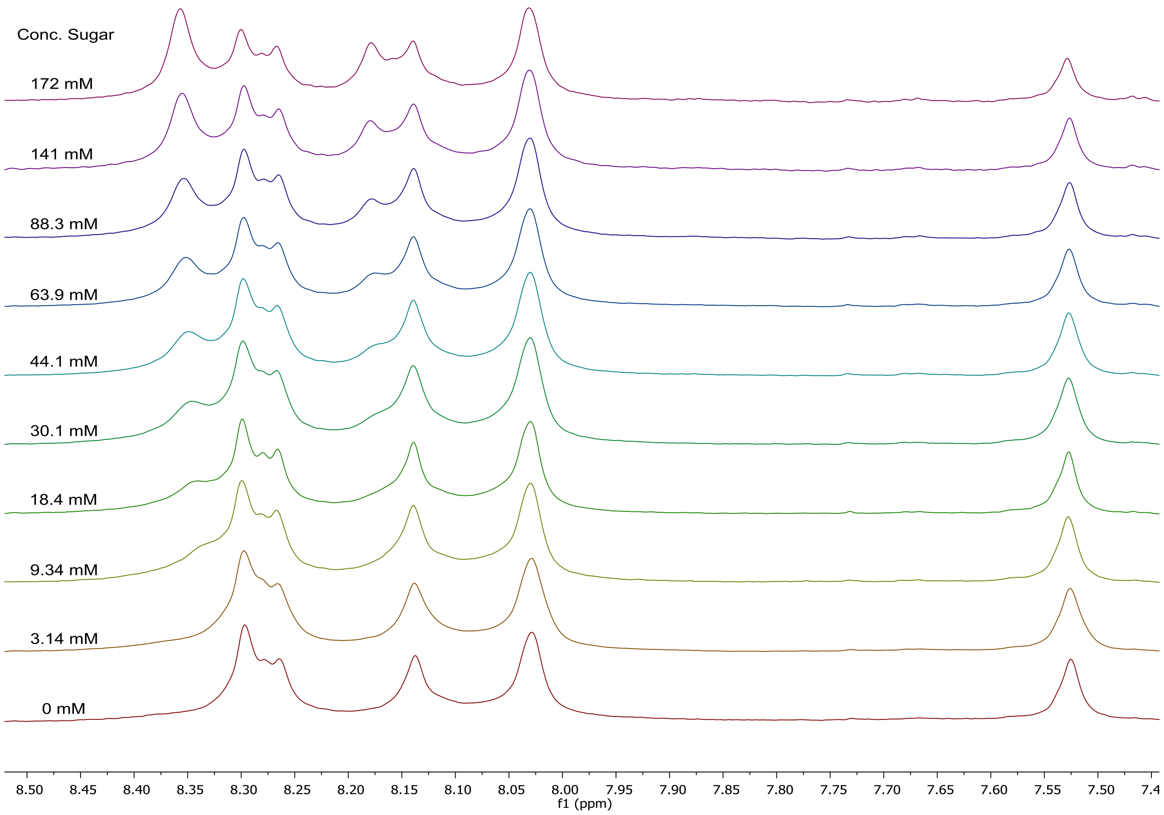

Figure S39. Partial ^{1}H NMR spectra from the titration of eclipsed receptor **5** (0.15 mM) with D-glucose (**16**) (632 mM) in D_2O at 298 K. Spectra imply binding with medium/fast exchange, which could not be quantified from this study.

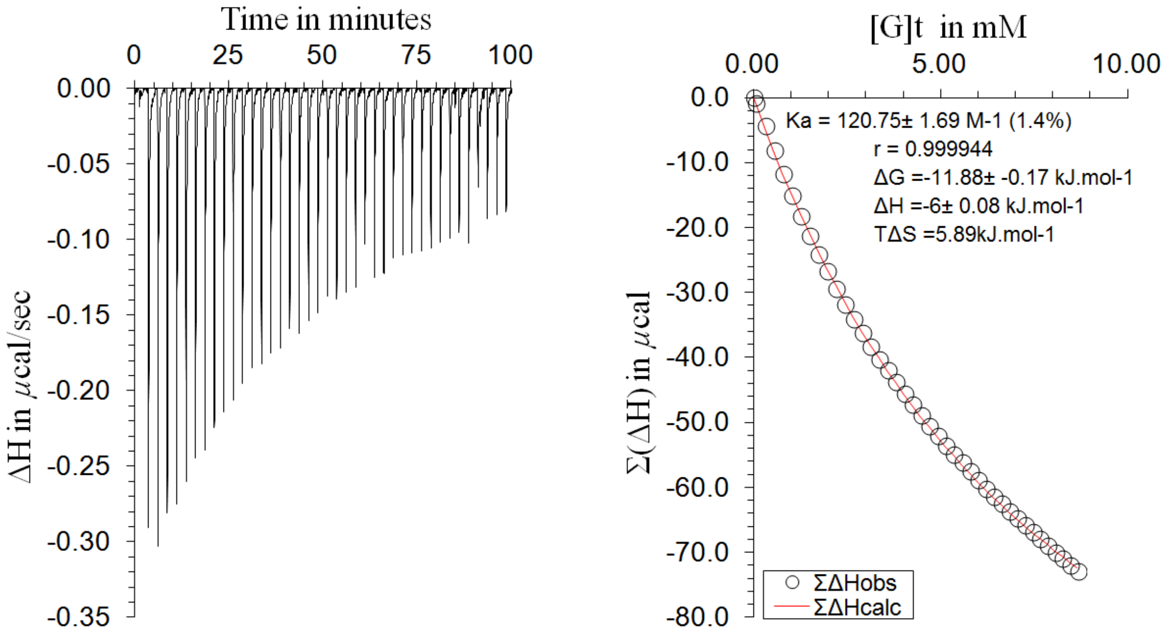

Figure S40. ITC binding study of eclipsed receptor **5** (0.50 mM) titrated with D-glucose (**16**) (20 mM) in H_2O. The sum of heat evolution (µcal) was plotted as a function of the concentration of carbohydrate (mM) and fitted to a 1:1 binding model indicating K_a = 121 ± 2 M^{-1} (1.4%) and r = 0.9999 (thermodynamic data also given in the figure).

D-Mannose

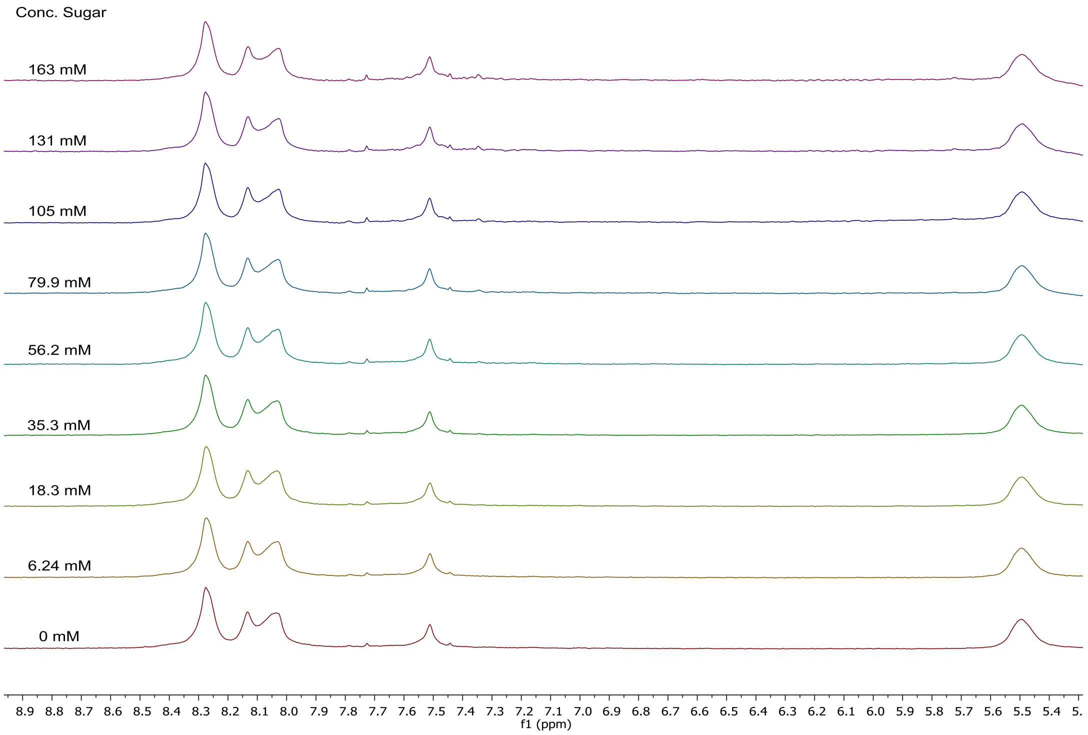

Figure S41. Partial ^{1}H NMR spectra from the titration of eclipsed receptor **5** (0.15 mM) with D-mannose (506 mM) in D_2O at 298 K. No evidence for binding can be detected.

D-Galactose

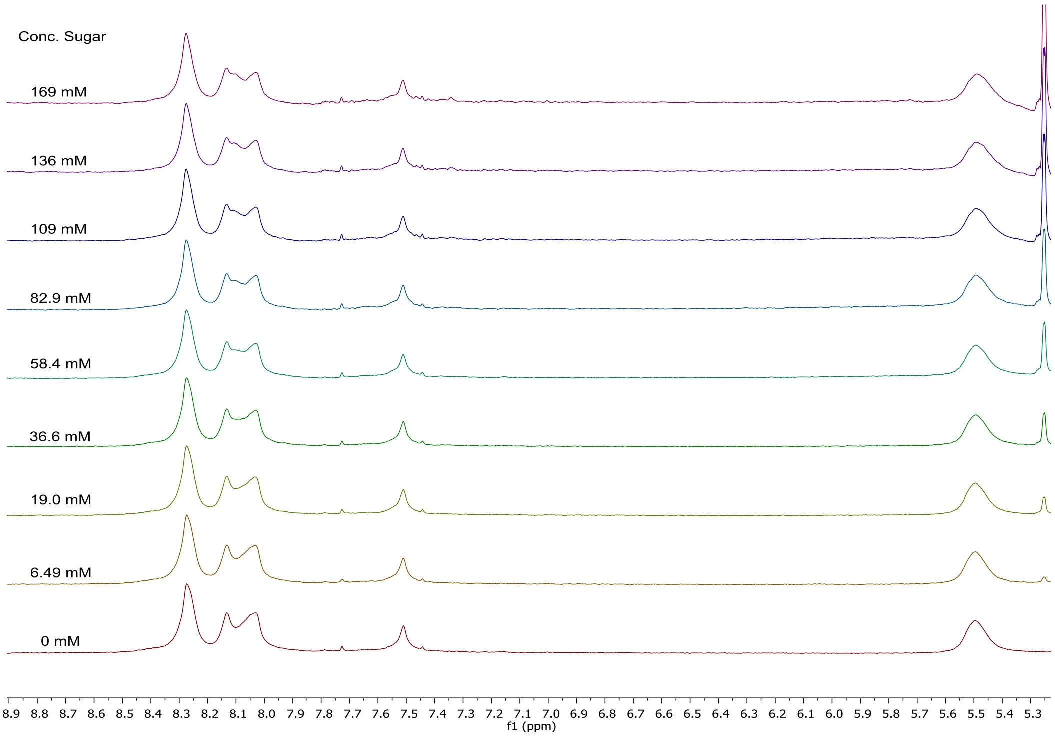

Figure S42. Partial ^{1}H NMR spectra from the titration of eclipsed receptor **5** (0.15 mM) with D-galactose (525 mM) in D_2O at 298 K. No evidence for binding can be detected.

D-Cellobiose

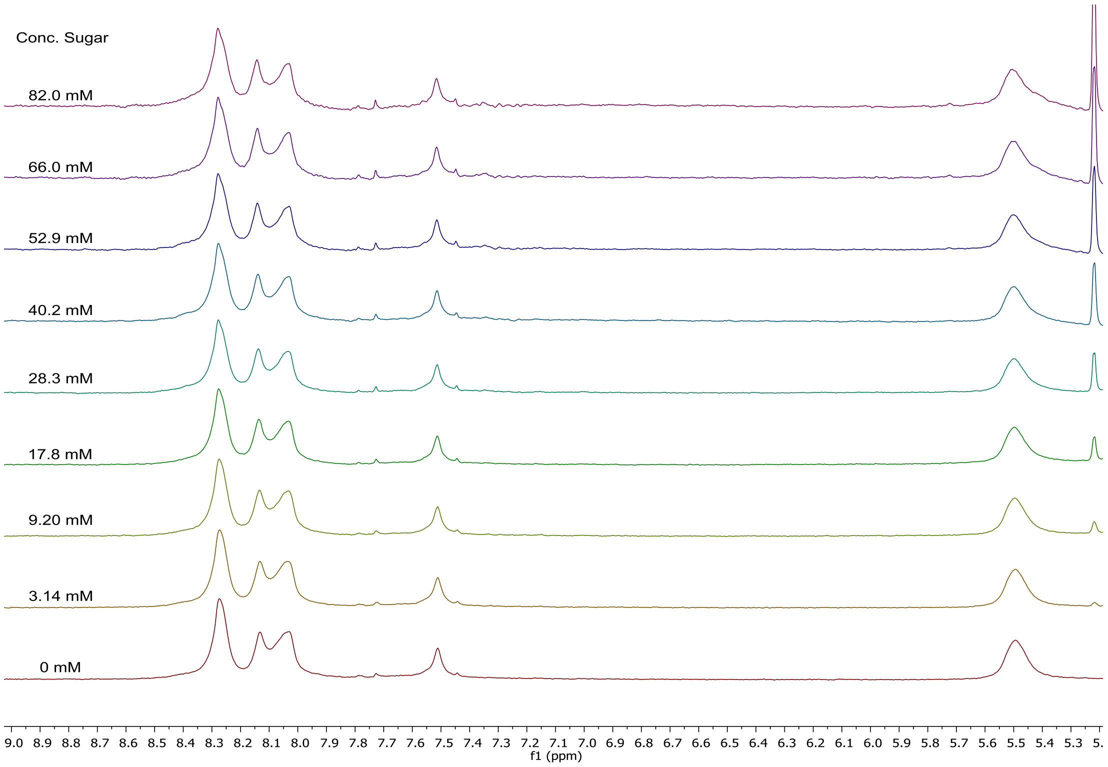

Figure S43. Partial ^{1}H NMR spectra from the titration of eclipsed receptor **5** (0.15 mM) with D-cellobiose (255 mM) in D_2O at 298 K. No evidence for binding can be detected.

N-Acetyl-D-galactosamine

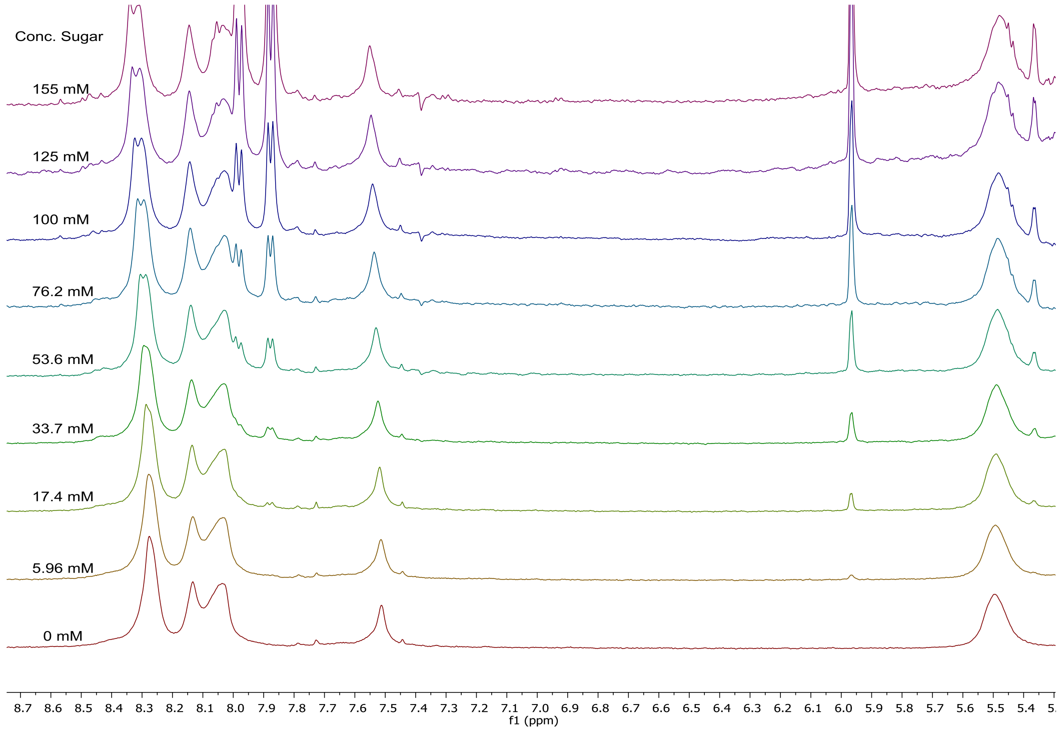

Figure S44. Partial ^{1}H NMR spectra from the titration of eclipsed receptor **5** (0.15 mM) with *N*-acetyl-D-galactosamine (482 mM) in D_2O at 298 K. Spectra show minor changes consistent with binding with medium/fast exchange, which could not however be quantified from this study.

N-Acetyl-D-mannosamine

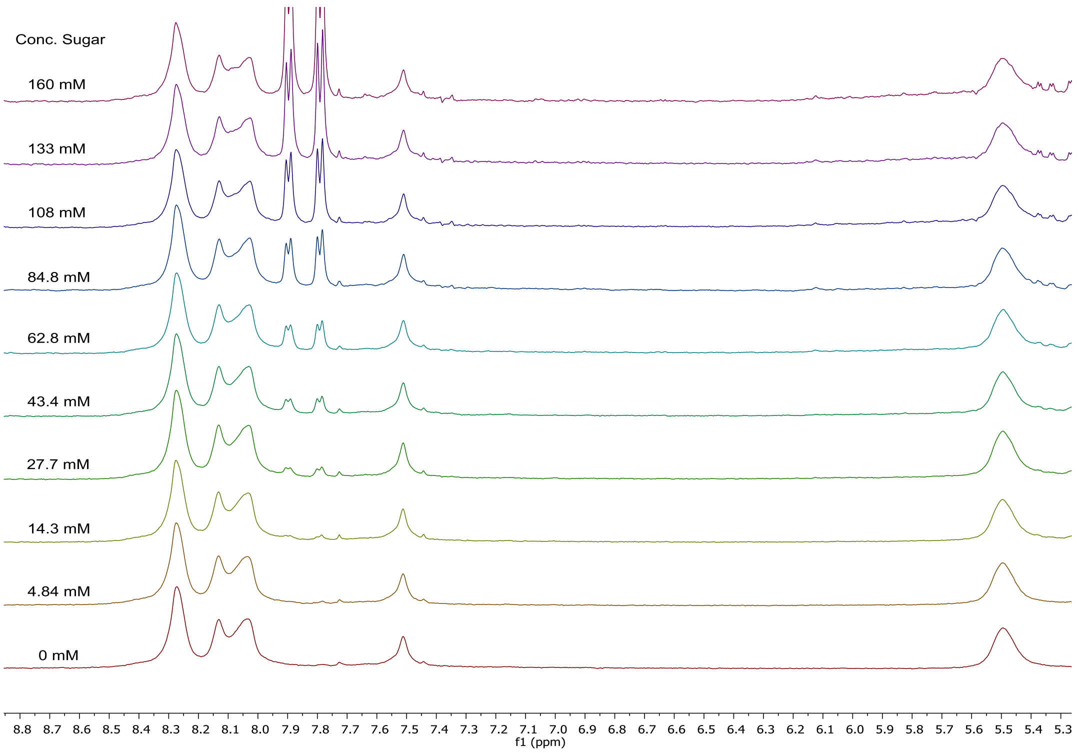

Figure S45. Partial ^{1}H NMR spectra from the titration of eclipsed receptor **5** (0.15 mM) with *N*-acetyl-D-mannosamine (489 mM) in D_2O at 298 K. Spectra show minor changes consistent with binding with medium/fast exchange, which could not however be quantified from this study.

N,N'-Diacetyl-D-chitobiose

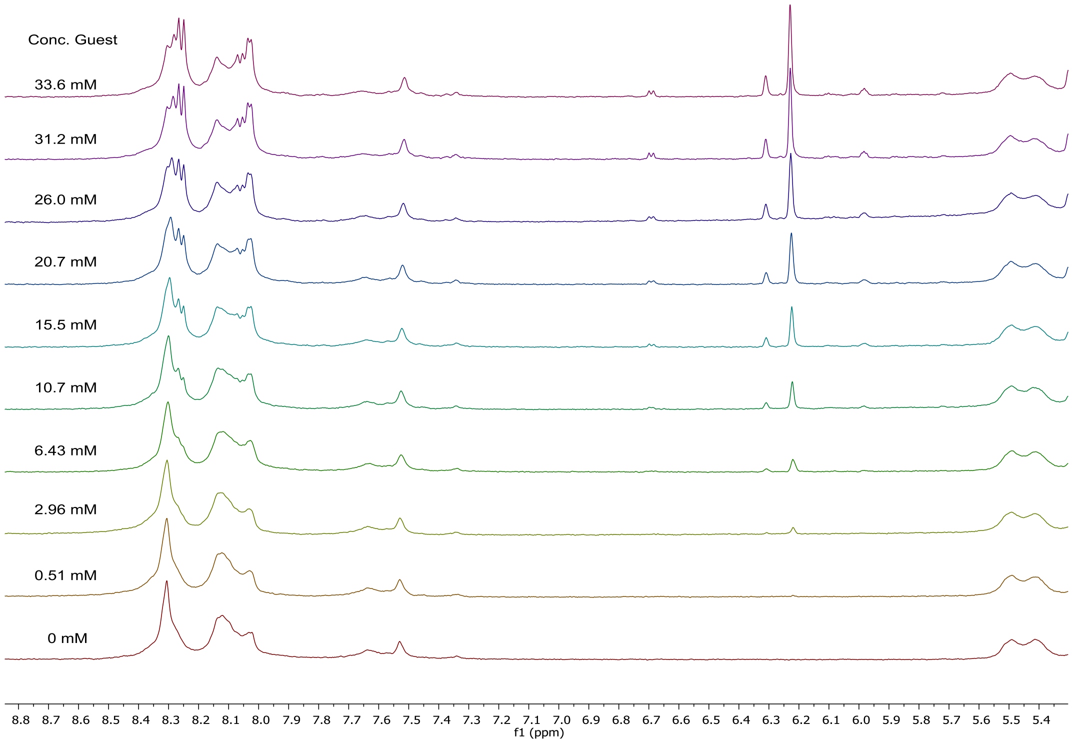

Figure S46. Partial ^{1}H NMR spectra from the binding study of eclipsed receptor **5** (0.2 mM) titrated with *N,N'*-diacetyl-D-chitobiose (102 mM) in D_2O at 298 K. No evidence for binding can be detected.

Staggered receptor 9

Methyl *N*-acetyl-β-D-glucosaminide (2)

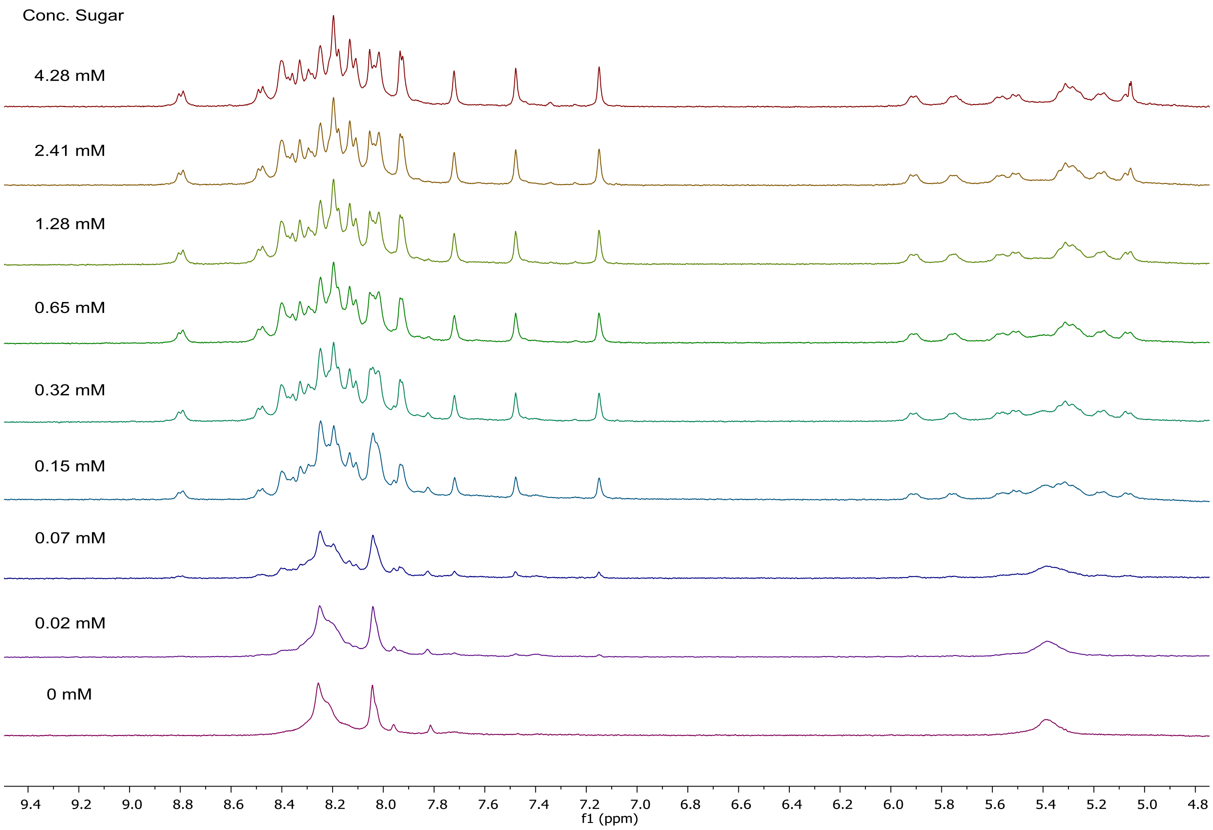

Figure S47. ^{1}H NMR binding study of staggered receptor **9** (0.15 mM) titrated with methyl *N*-acetyl-β-D-glucosaminide (**2**) (18.4 mM) in D_2O at 298 K. Spectra imply binding with slow exchange, allowing analysis of integrals to give K_a (see below).

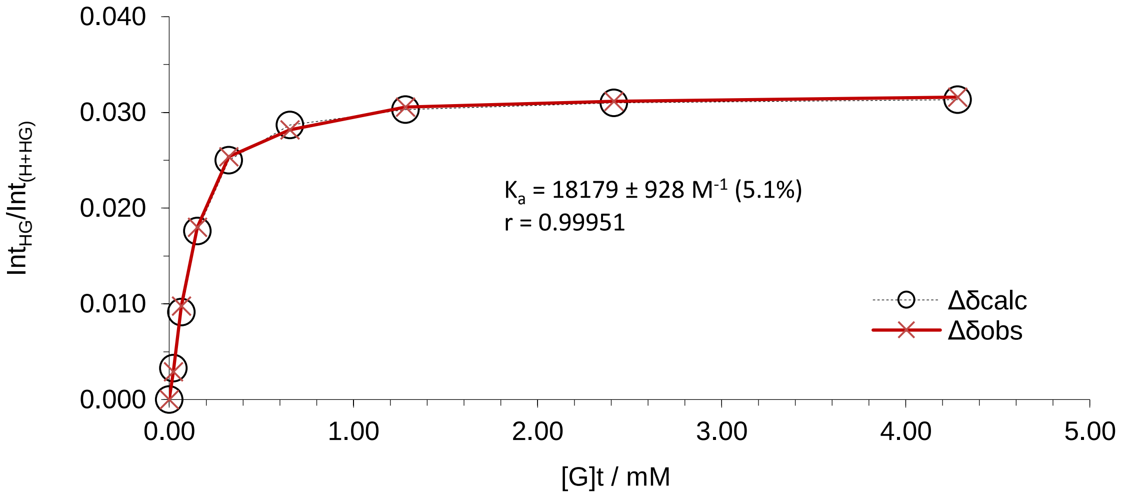

Figure S48. ^{1}H NMR binding study of staggered receptor **9** (0.15 mM) titrated with methyl *N*-acetyl-β-D-glucosaminide (**2**)(18.4 mM) in D_2O at 298 K. Plot of the observed integral (peak at 8.80 ppm integrated against 9.50-7.00 ppm) versus guest concentration (mM). The calculated values for the integrals are overlaid, predicting K_a = 18179 ± 928 M^{-1} (5.1%). The points used in the slow exchange method of K_a calculation are labelled yellow.

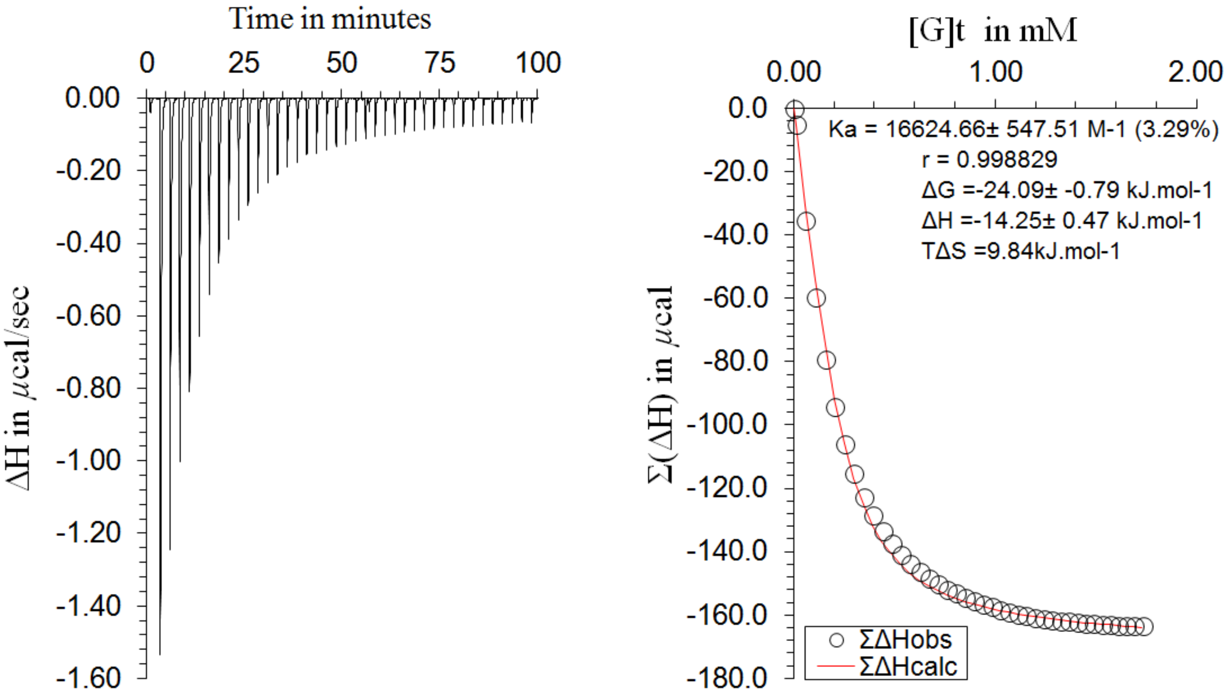

Figure S49. ITC binding study of staggered receptor **9** (0.25 mM) titrated with methyl *N*-acetyl-β-D-glucosaminide (**2**) (10 mM) in H_2O. The sum of heat evolution (µcal) was plotted as a function of the concentration of carbohydrate (mM) and fitted to a 1:1 binding model indicating K_a = 16625 ± 548 M^{-1} (3.3%) and r = 0.9988 (thermodynamic data also given in the figure).

Glycopeptide (3)

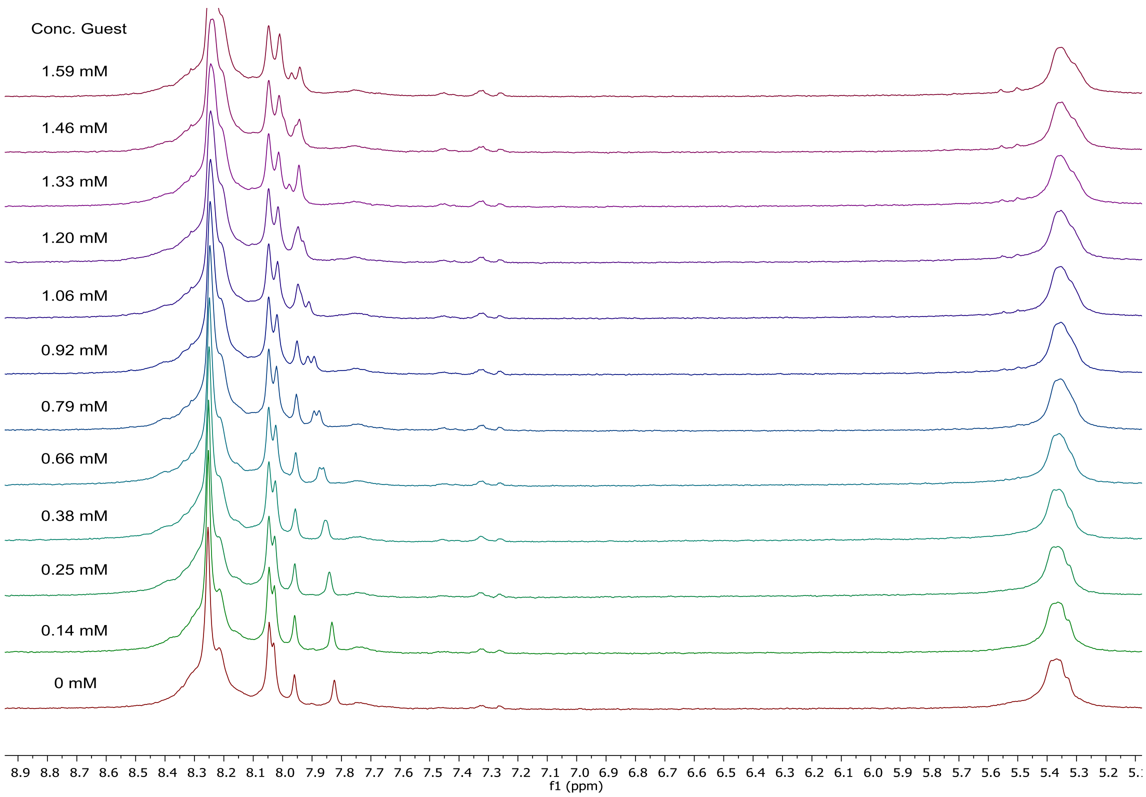

Figure S50. Partial ^{1}H NMR spectra from the binding study of staggered receptor **9** (0.25 mM) titrated with glycopeptide **3** (5.12 mM) in D_2O at 298 K. Spectra imply binding with medium/fast exchange, which could be relatively strong given that changes occur at low concentrations. However, quantification was not possible from this study.

Methyl *N*-acetyl-α-D-glucosaminide (13)

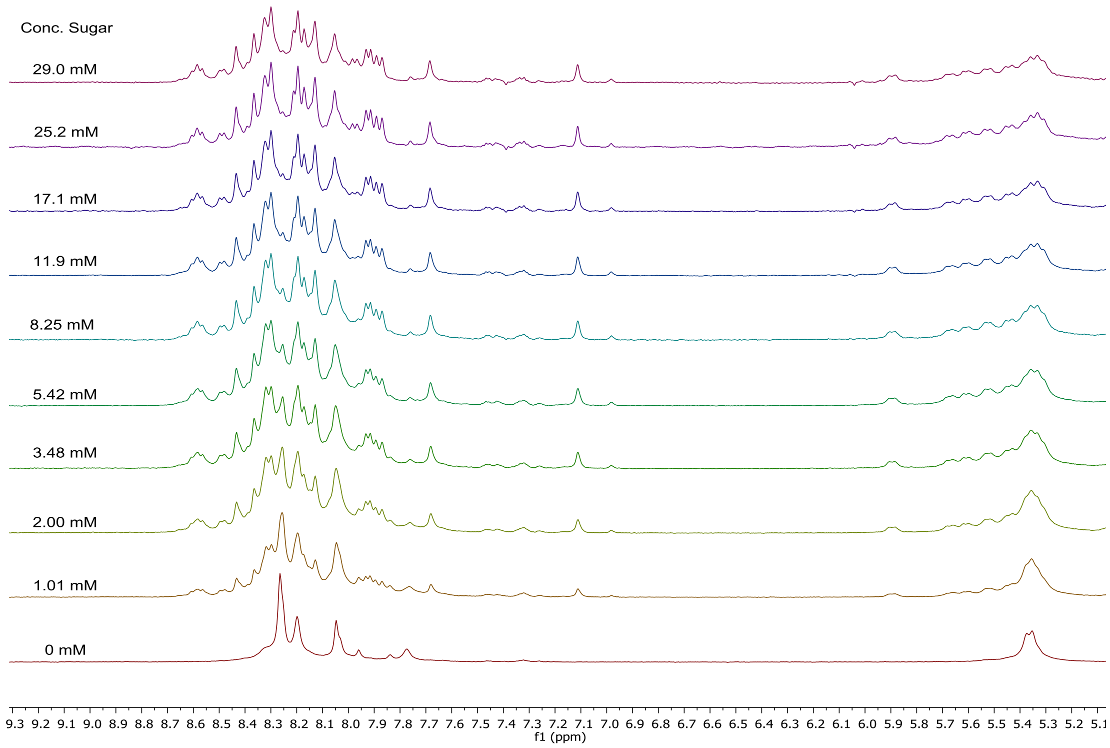

Figure S51. Partial ^{1}H NMR spectra from the binding study of staggered receptor **9** (0.15 mM) titrated with methyl *N*-acetyl-α-D-glucosaminide (**13**) (202 mM) in D_2O at 298 K. Spectra imply binding with slow exchange, allowing analysis of integrals to give K_a (see below).

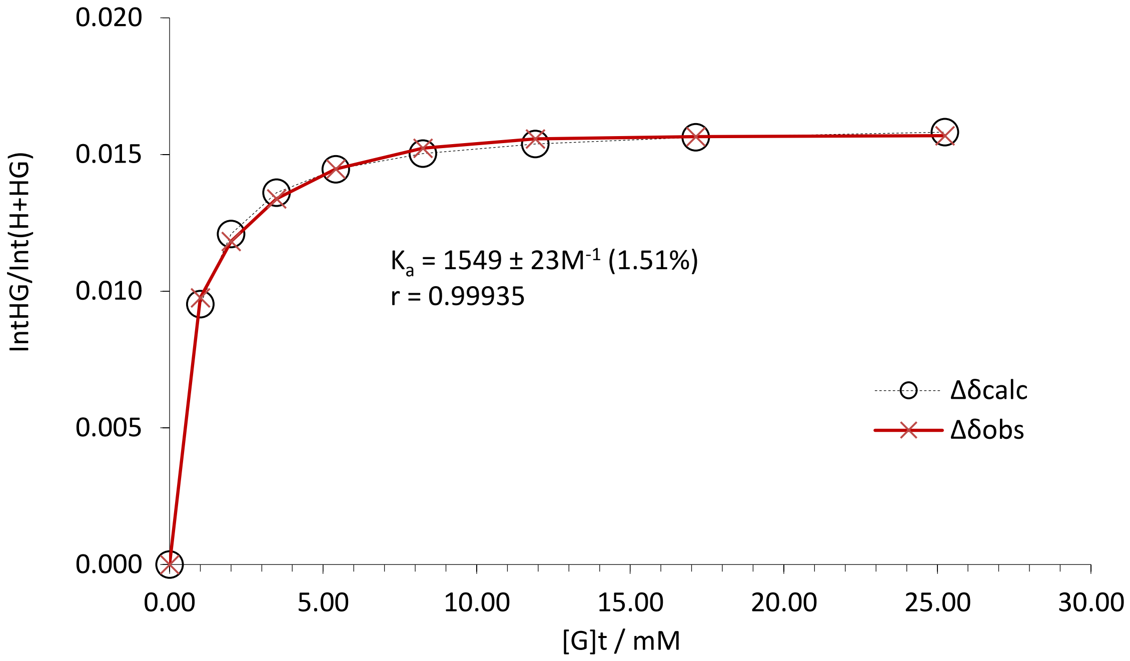

Figure S52. ^{1}H NMR binding study of staggered receptor **9** (0.15 mM) titrated with methyl *N*-acetyl-α-D-glucosaminide (**13**) (202 mM) in D_2O at 298 K. Plot of the observed integral (peak at 7.11 ppm integrated against 9.50-6.85 ppm) versus guest concentration (mM). The calculated values for the integrals are overlaid, predicting K_a = 1549 ± 23 M^{-1} (1.51%). The points used in the slow exchange method of K_a calculation are labelled yellow.

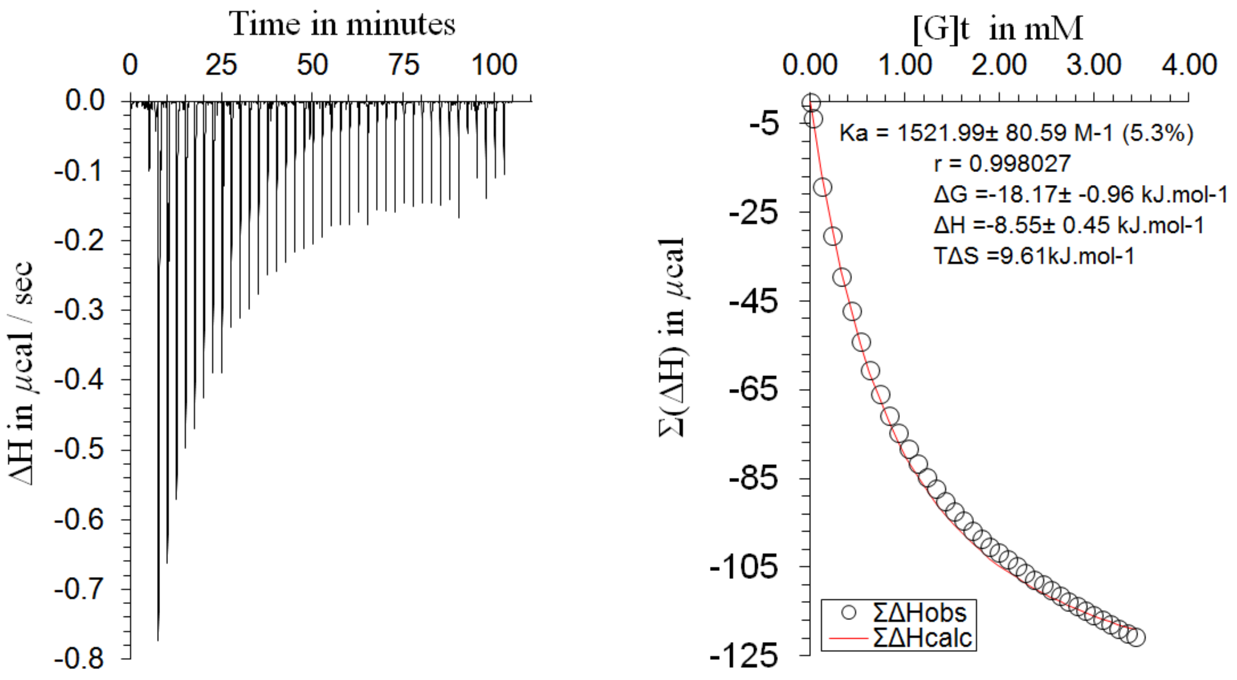

Figure S53. ITC binding study of staggered receptor **9** (0.30 mM) titrated with methyl *N*-acetyl-α-D-glucosaminide (**13**) (20 mM) in H_2O. The sum of heat evolution (µcal) was plotted as a function of the concentration of carbohydrate (mM) and fitted to a 1:1 binding model indicating K_a = 1522 ± 81 M^{-1} (5.3%) and r = 0.9980 (thermodynamic data also given in the figure).

N-Acetyl-D-glucosamine (14)

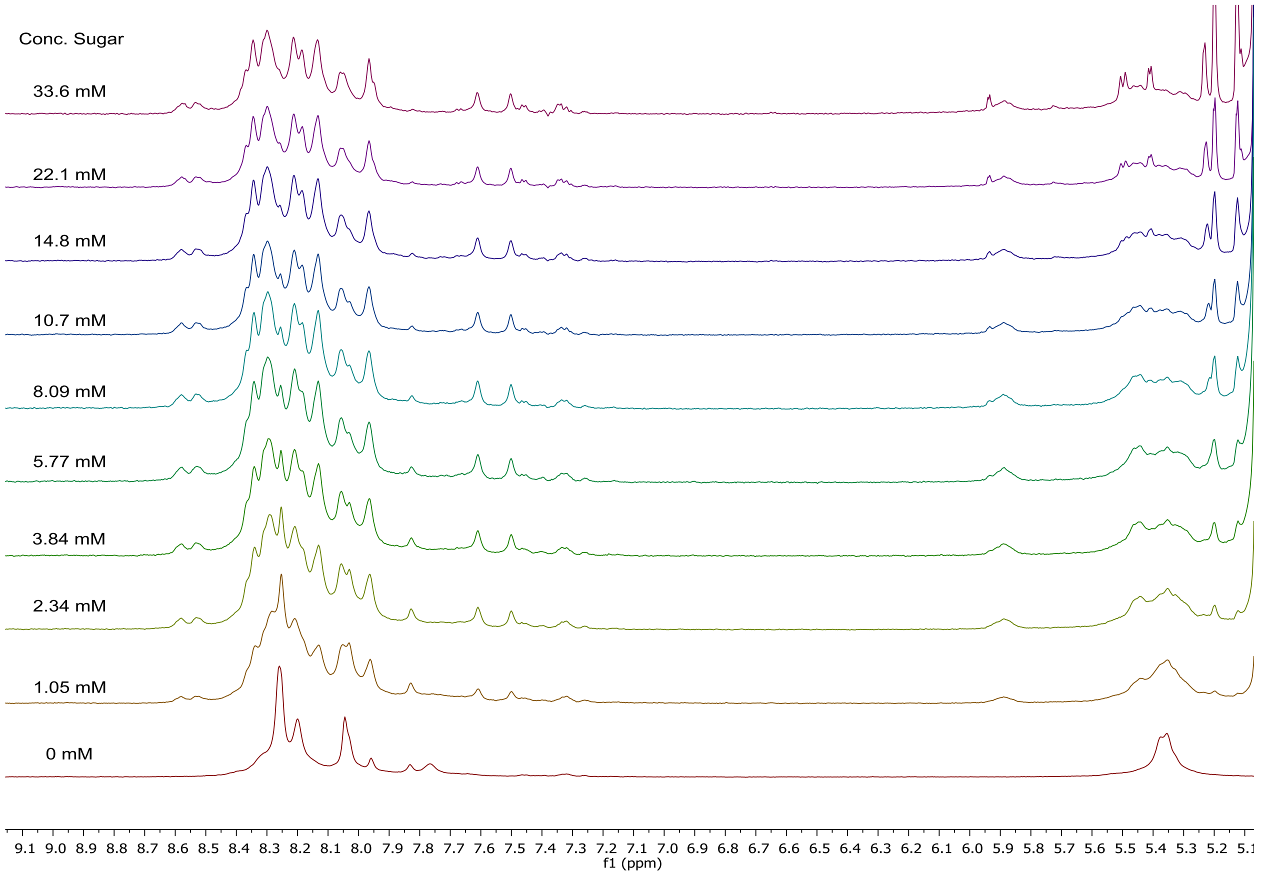

Figure S54. ^{1}H NMR binding study of staggered receptor **9** (0.15 mM) titrated with *N*-acetyl-D-glucosamine (**14**) (106 mM) in D_2O at 298 K. Spectra imply binding with slow exchange, allowing analysis of integrals to give K_a (see below).

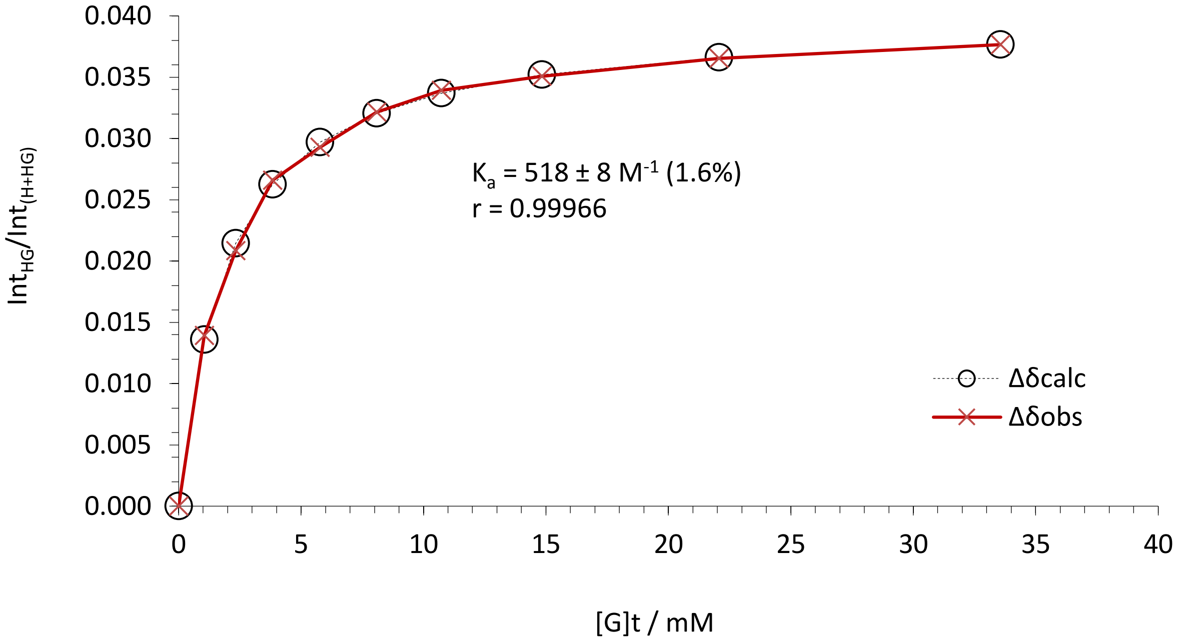

Figure S55. ^{1}H NMR binding study of staggered receptor **9** (0.15 mM) titrated with *N*-acetyl-D-glucosamine (**14**) (106 mM) in D_2O at 298 K. Plot of the observed integral (peak at 5.89 ppm integrated against 9.50-7.00 ppm) versus guest concentration (mM). The calculated values for the integrals are overlaid, predicting K_a = 518 ± 8 M^{-1} (1.6%).

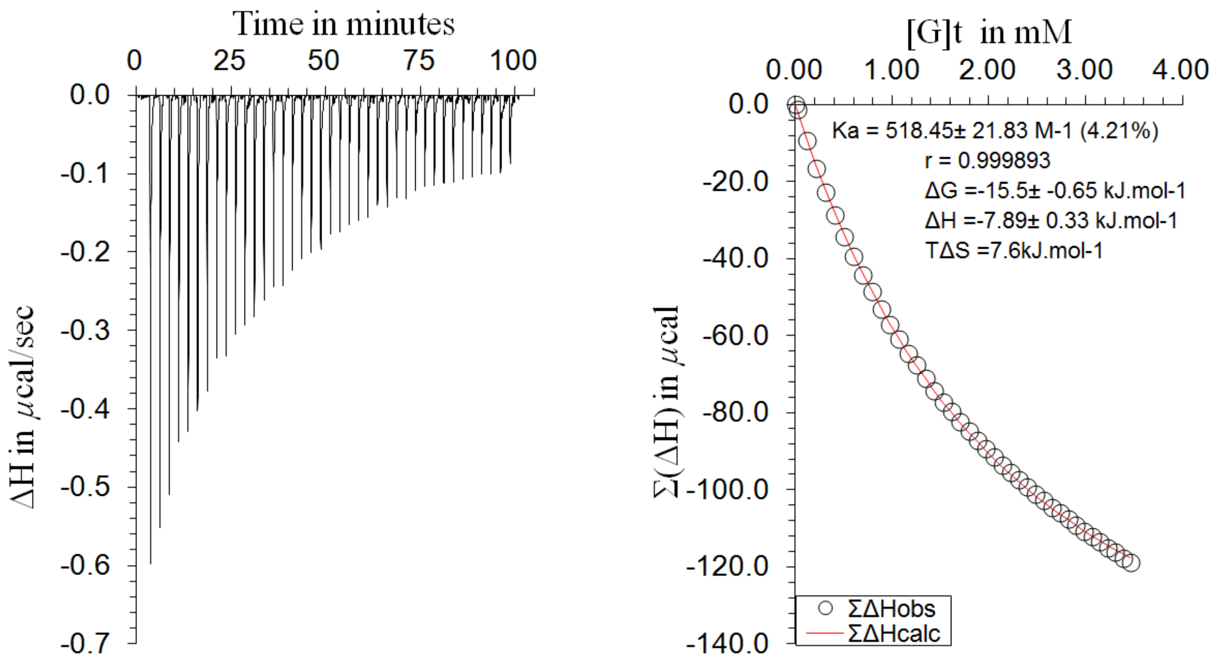

Figure S56. ITC binding study of staggered receptor **9** (0.50 mM) titrated with *N*-acetyl-D-glucosamine (**14**) (20 mM) in H_2O. The sum of heat evolution (µcal) was plotted as a function of the concentration of carbohydrate (mM) and fitted to a 1:1 binding model indicating K_a = 519 ± 22 M^{-1} (4.2%) and r = 0.9999 (thermodynamic data also given in the figure).

Methyl β-D-glucoside (15)

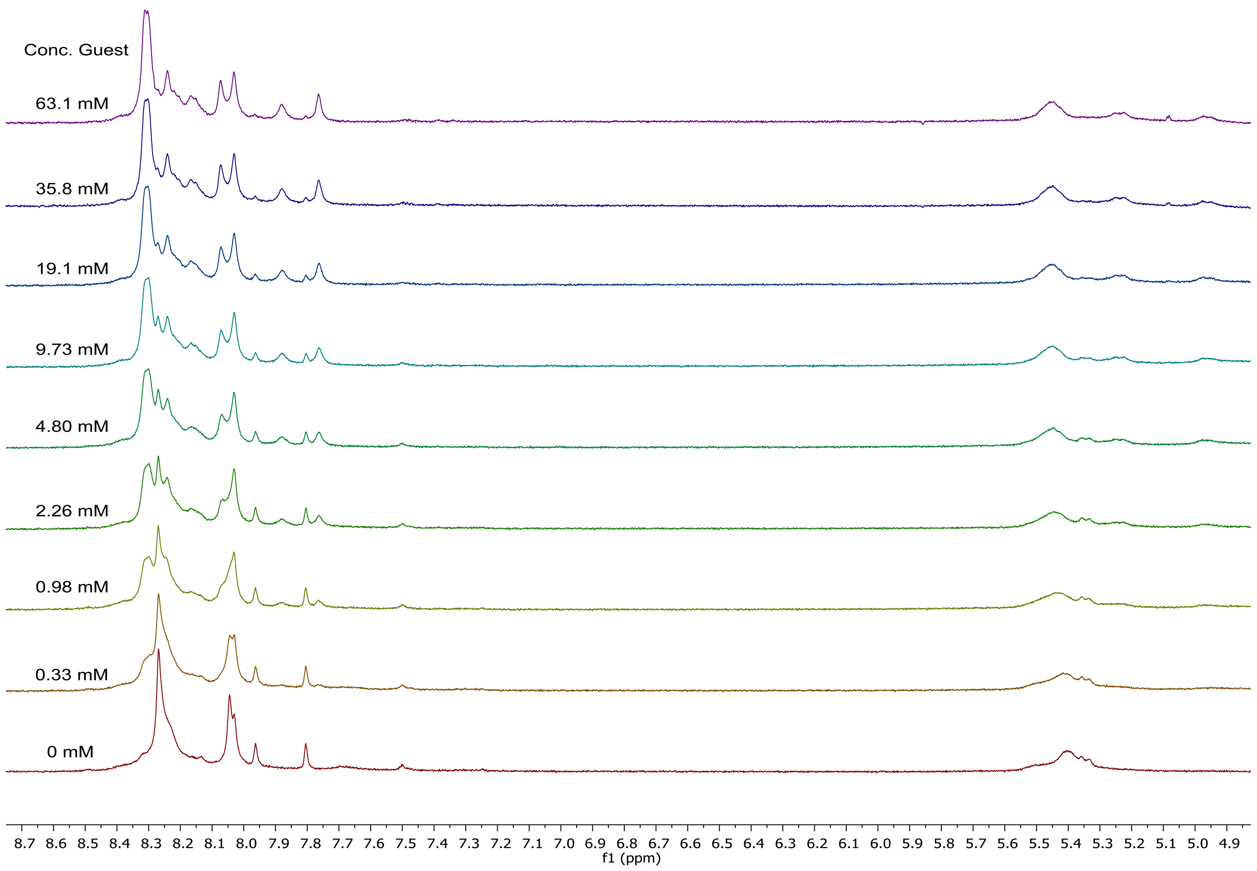

Figure S57. Partial ^{1}H NMR spectra from the titration of staggered receptor **9** (0.25 mM) with methyl β-D-glucoside (**15**) (261 mM) in D_2O at 298 K. Spectra imply binding with slow exchange, allowing analysis of integrals to give K_a (see below).

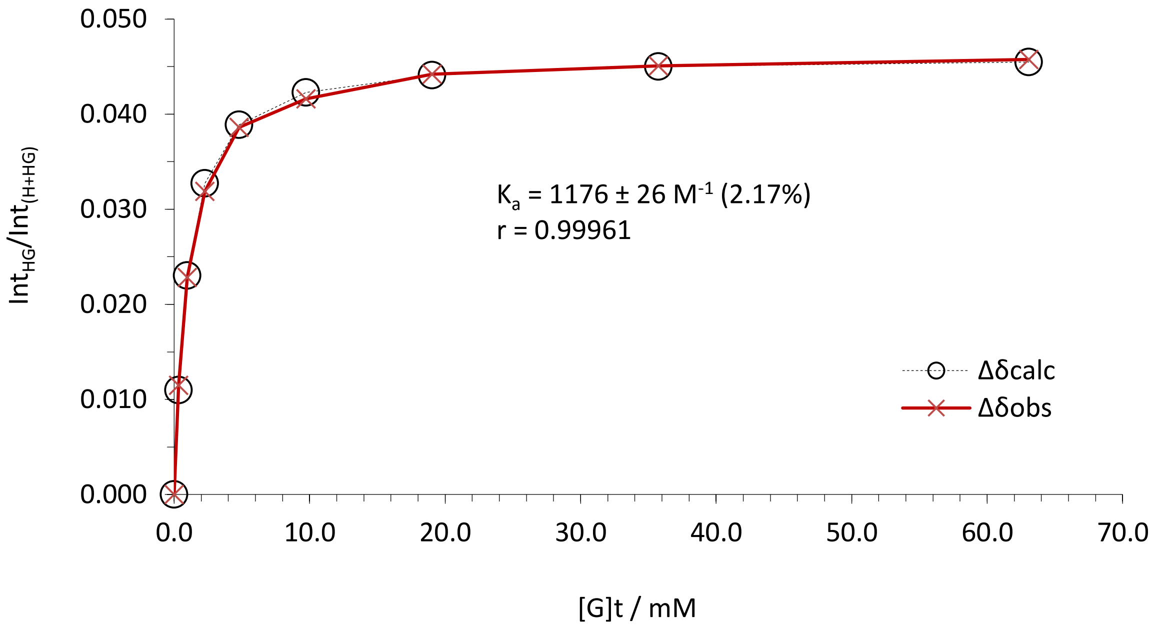

Figure S58. ^{1}H NMR binding study of staggered receptor **9** (0.25 mM) titrated with methyl β-D-glucoside (**15**) (261 mM) in D_2O at 298 K. Plot of the observed integral (7.76 ppm peak against 9.50-7.00 ppm) versus guest concentration (mM). The calculated values for the integrals are overlaid, predicting K_a = 1176 ± 26 M^{-1} (2.17%).

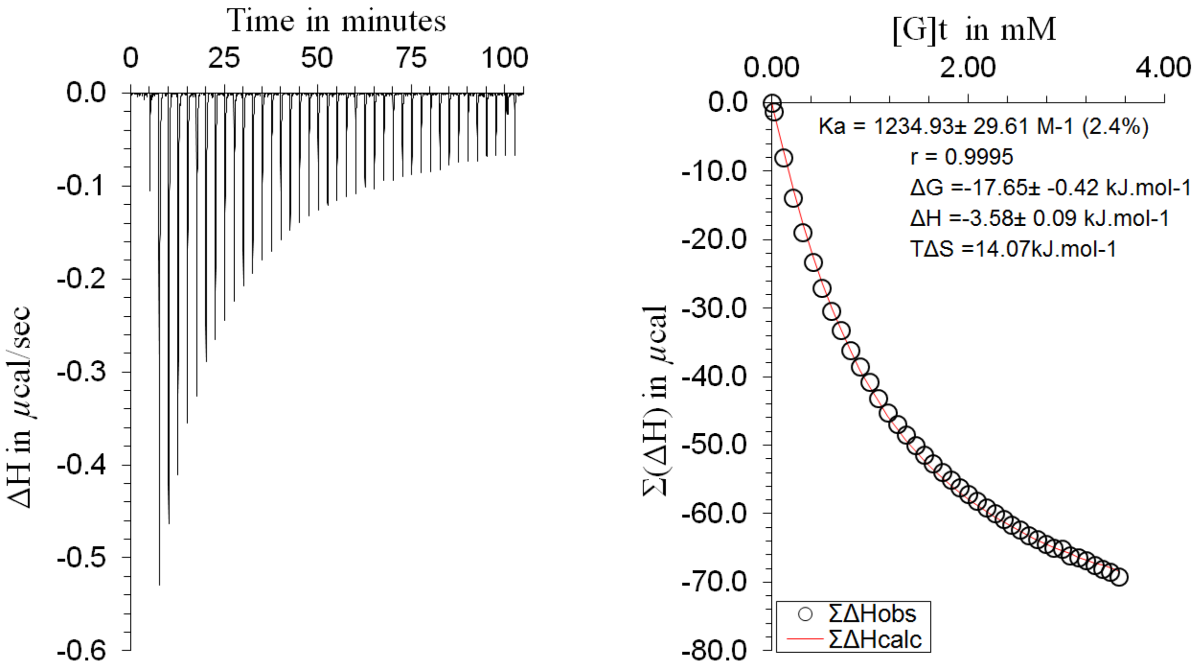

Figure S59. ITC binding study of staggered receptor **9** (0.50 mM) titrated with methyl β-D-glucoside (**15**) (20 mM) in H_2O. The sum of heat evolution (µcal) was plotted as a function of the concentration of carbohydrate (mM) and fitted to a 1:1 binding model indicating K_a = 1235 ± 30 M^{-1} (2.4%) and r = 0.9995 (thermodynamic data also given in the figure).

D-Glucose (16)

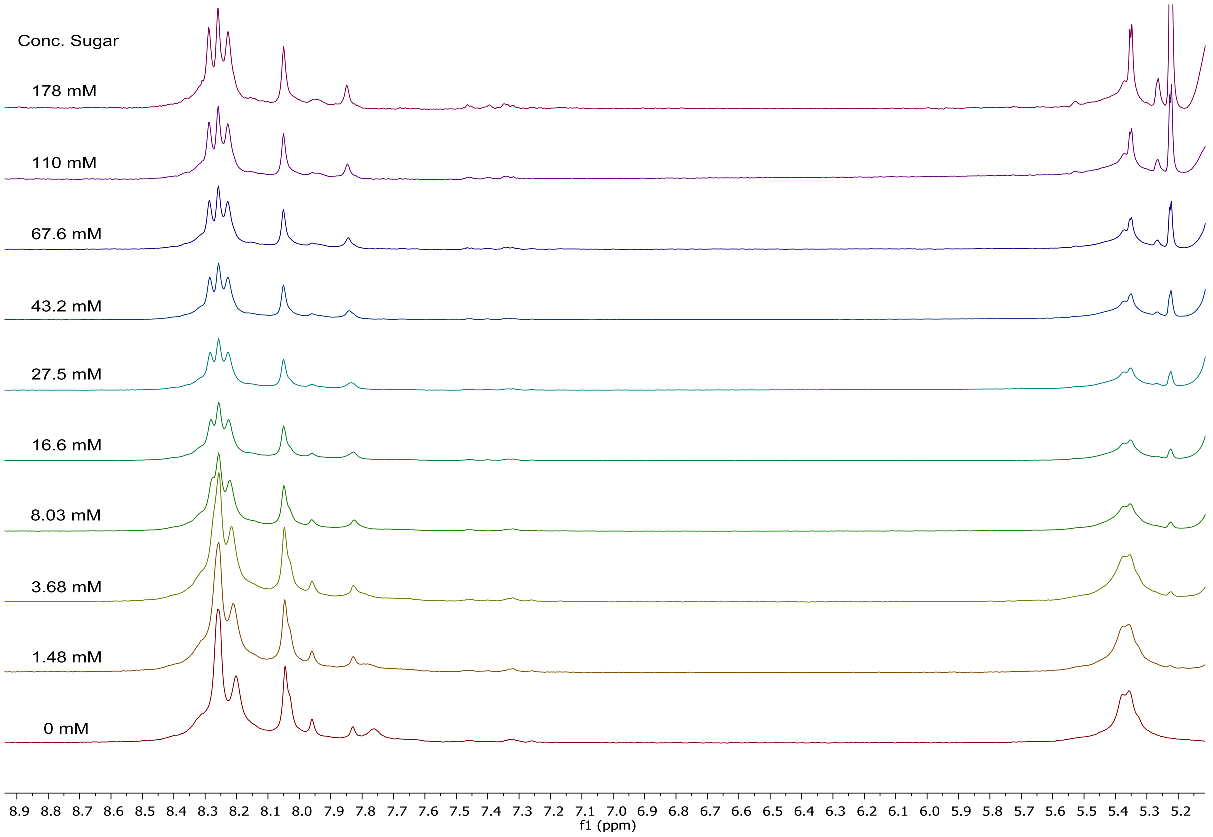

Figure S60. Partial ^{1}H NMR spectra from the titration of staggered receptor **9** (0.15 mM) with D-glucose (**16**) (592 mM) in D_2O at 298 K. Spectra imply binding with medium/fast exchange, which could not be quantified from this study.

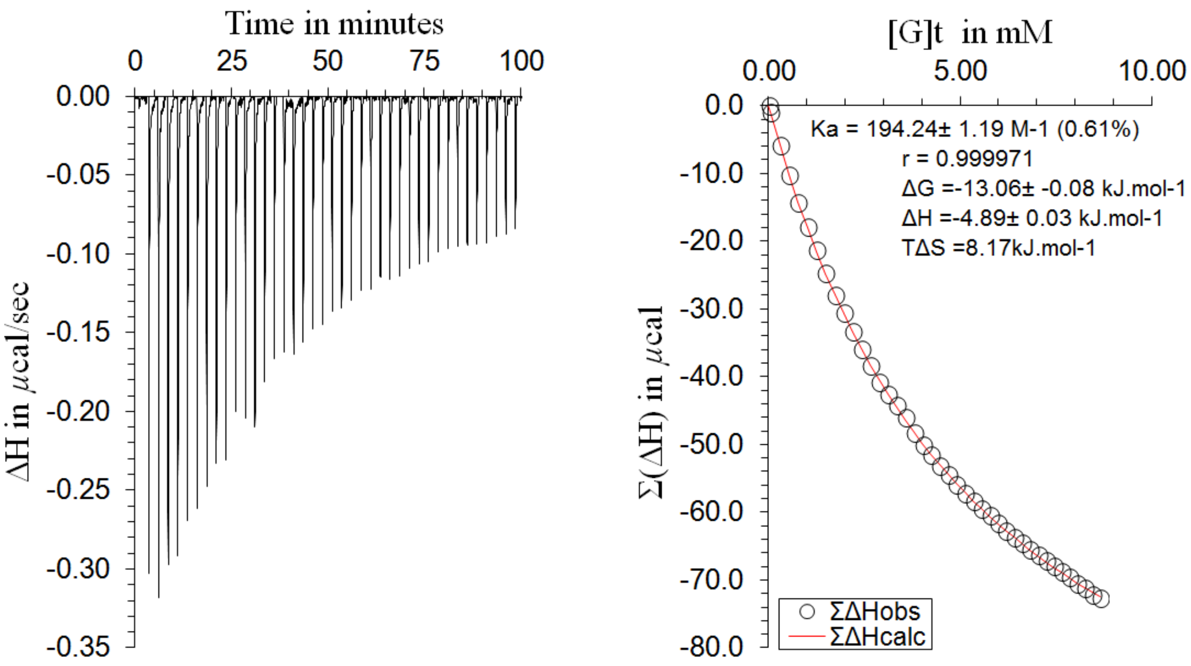

Figure S61. ITC binding study of staggered receptor **9** (0.50 mM) titrated with D-glucose (**16**) (20 mM) in H_2O. The sum of heat evolution (µcal) was plotted as a function of the concentration of carbohydrate (mM) and fitted to a 1:1 binding model indicating K_a = 194 ± 1 M^{-1} (0.6%) and r = 0.99997 (thermodynamic data also given in the figure).

D-Mannose

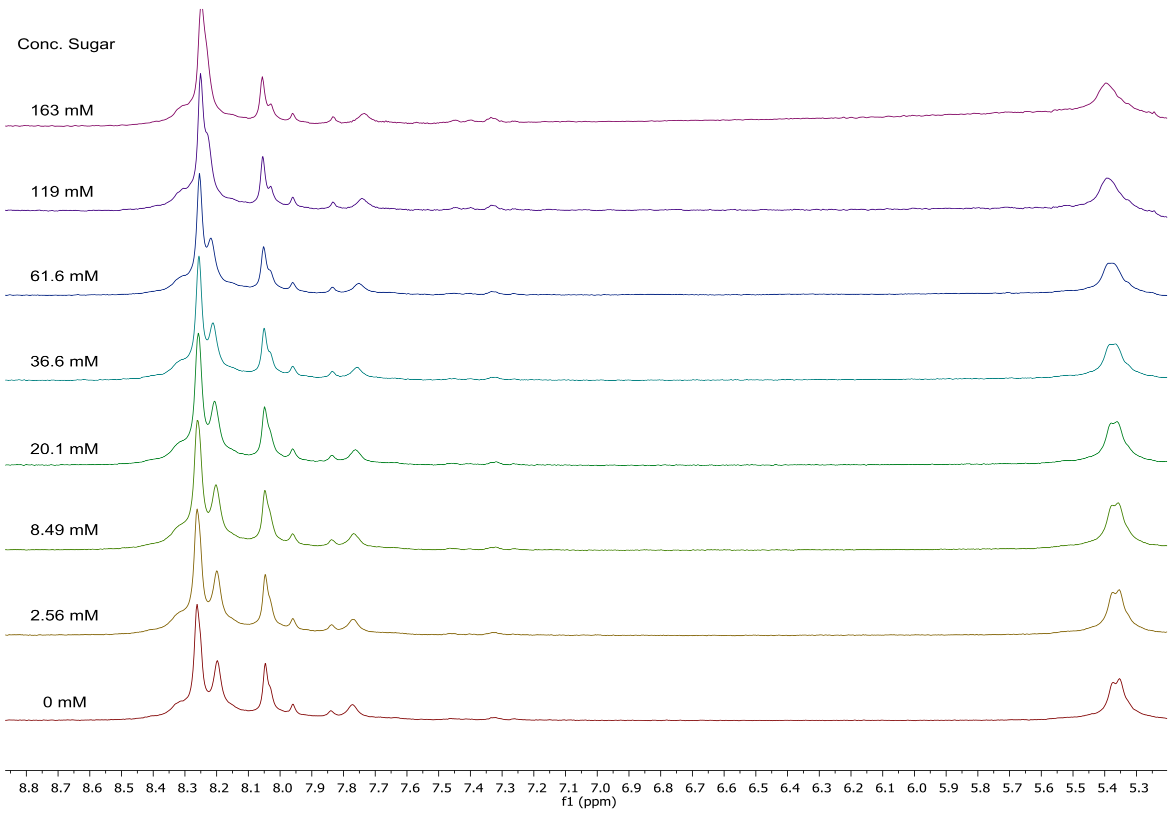

Figure S62. Partial ^{1}H NMR spectra from the titration of staggered receptor **9** (0.15 mM) with D-mannose (494 mM) in D_2O at 298 K. Spectra imply binding with medium/fast exchange, which could not be quantified from this study.

D-Galactose

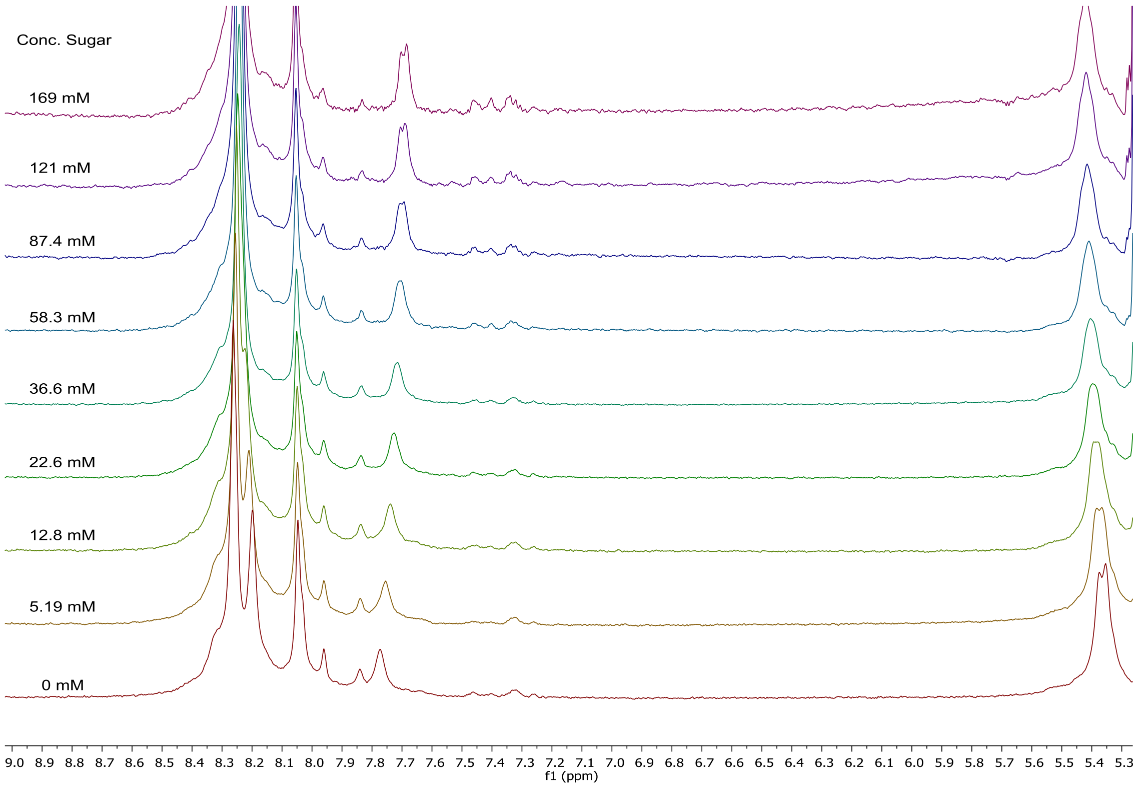

Figure S63. Partial ^{1}H NMR spectra from the titration of staggered receptor **9** (0.15 mM) with D-galactose (524 mM) in D_2O at 298 K. Spectra imply binding with medium/fast exchange, which could not be quantified from this study.

D-Cellobiose

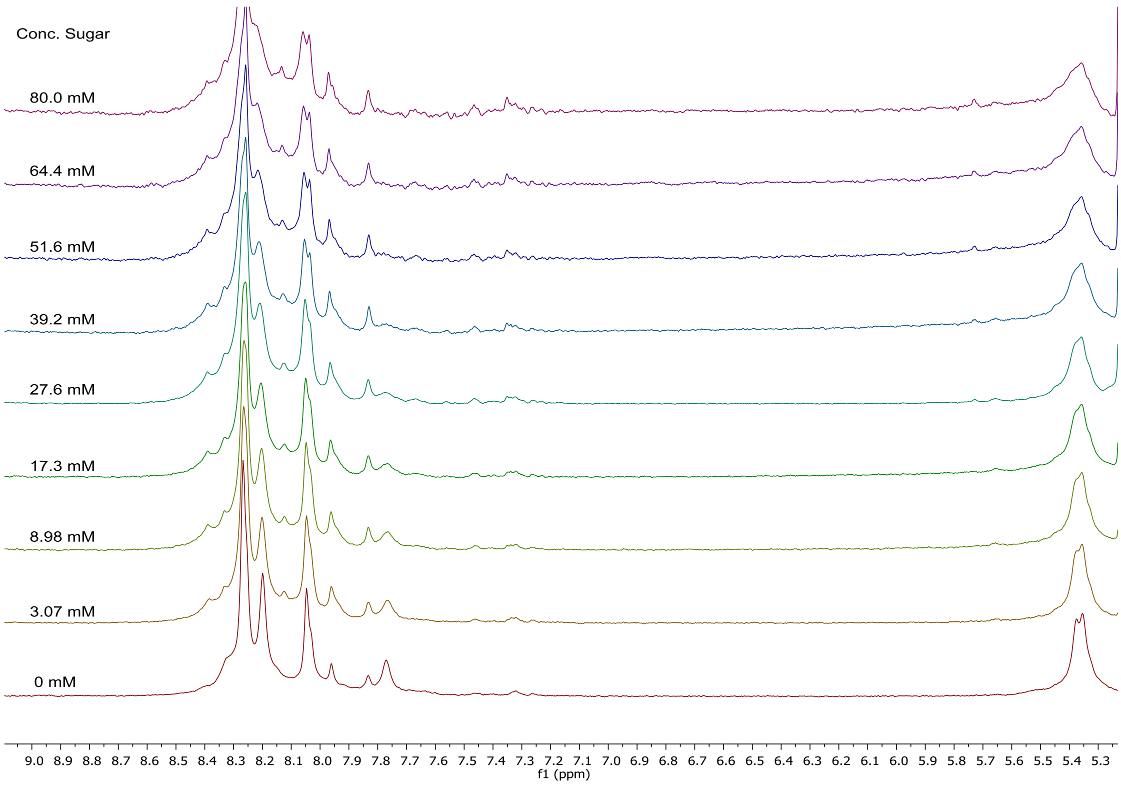

Figure S64. Partial ^{1}H NMR spectra from the titration of staggered receptor **9** (0.15 mM) with D-cellobiose (248 mM) in D_2O at 298 K. Spectra imply binding with medium/fast exchange, which could not be quantified from this study.

N-Acetyl-D-galactosamine

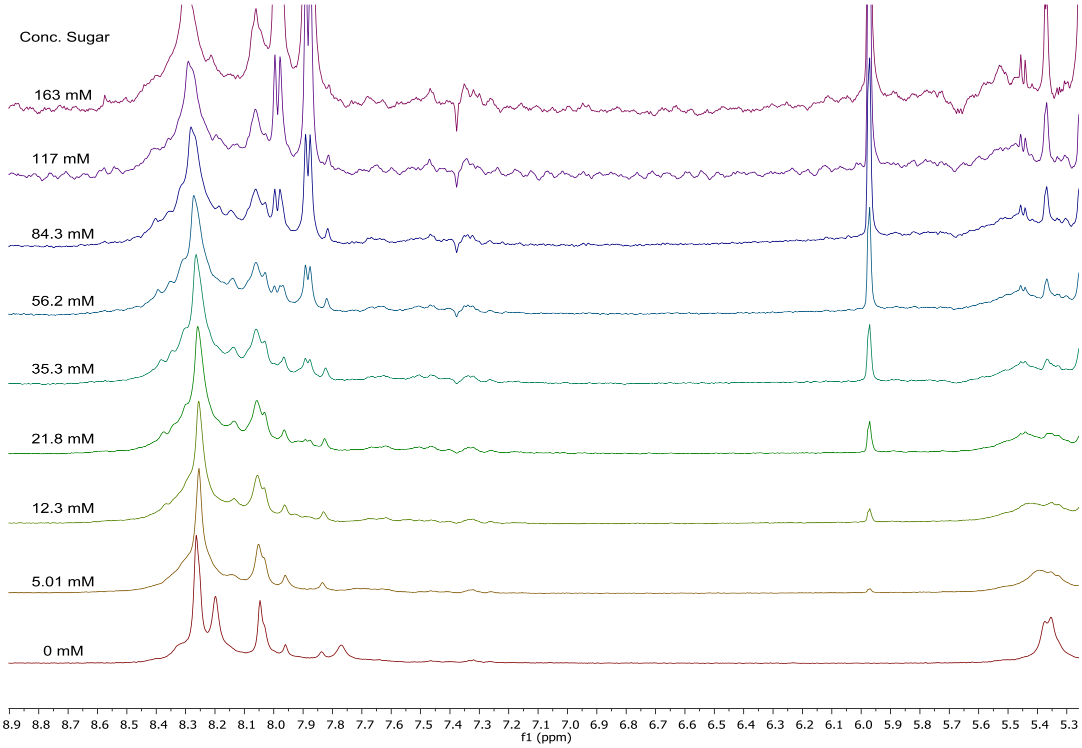

Figure S65. Partial ^{1}H NMR spectra from the titration of staggered receptor **9** (0.15 mM) with *N*-acetyl-D-galactosamine (506 mM) in D_2O at 298 K. Spectra imply binding with medium/fast exchange, which could not be quantified from this study.

N-Acetyl-D-mannosamine

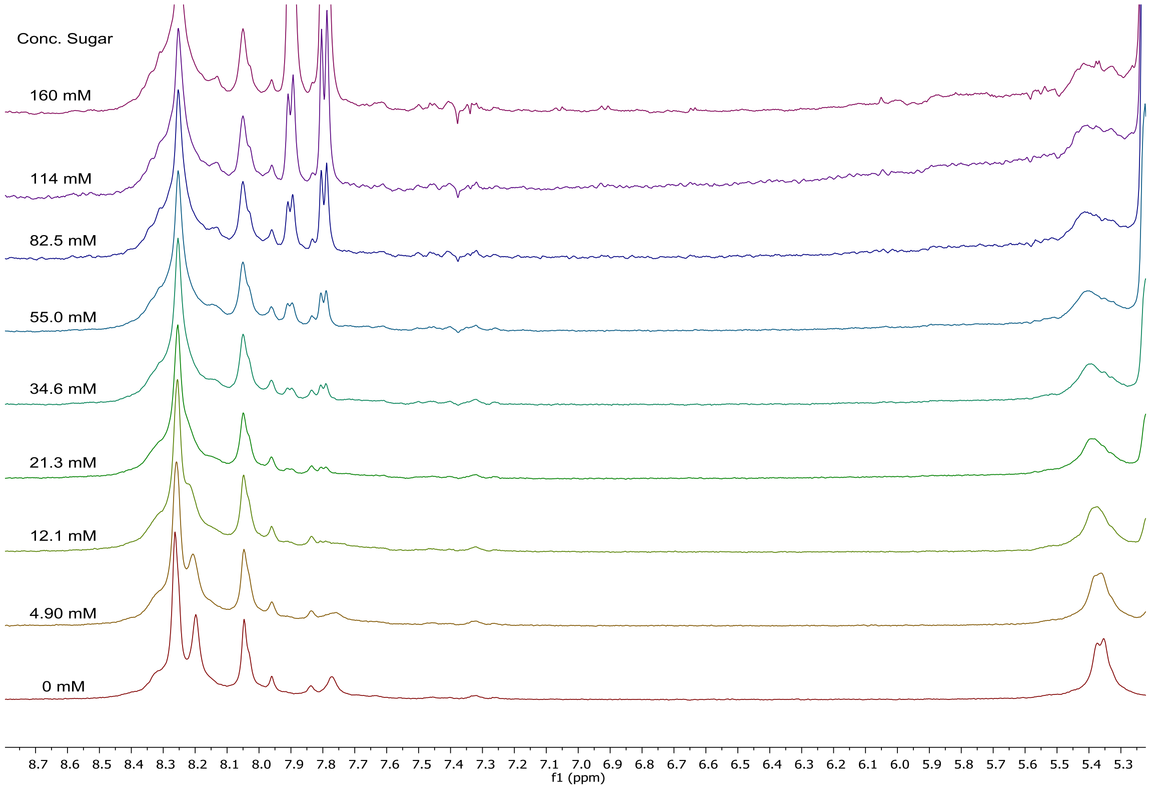

Figure S66. Partial ^{1}H NMR spectra from the titration of staggered receptor **9** (0.15 mM) with *N*-acetyl-D-mannosamine (495 mM) in D_2O at 298 K. Spectra imply binding with medium/fast exchange, which could not be quantified from this study.

N,N'-Diacetyl-D-chitobiose

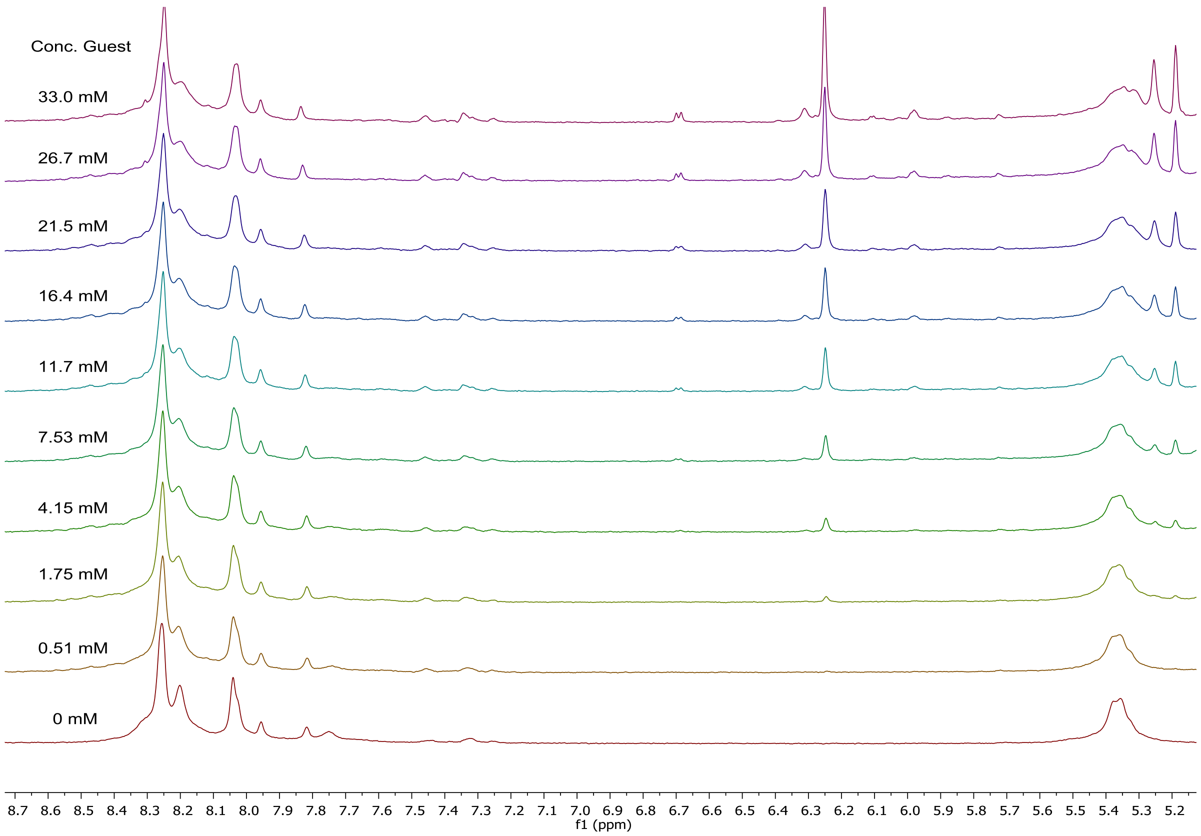

Figure S67. Partial ^{1}H NMR spectra from the binding study of staggered receptor **9** (0.13 mM) titrated with *N,N'*-diacetyl-D-chitobiose (18.4 mM) in D_2O at 298 K. Spectra imply binding with medium/fast exchange, which could not be quantified from this study.

4. Structural studies on receptor-guest complexes

General procedure to obtain 3D NMR structures

NMR structures were obtained for the four complexes for which slow exchange between free and bound states resulted in well-resolved spectra of the complexes (i.e. **5·2**, **5·2**, **9·2** and **9·13**). Clear cross peaks were observed in {^{1}H-^{1}H}-NOESY, -TOCSY and -COSY spectra of samples in 10% D_2O in H_2O.[8] Those involving the sugar protons and the protons of the pyrenyl cage (i.e. all except the dendrimeric side-chains) were assigned to obtain a self-consistent interpretation of each NMR data-set. To minimize the risk of spin diffusion, the NOESY spectra were all recorded with a short mixing time (150 ms).[9] Isolated nOe cross-peaks were integrated and each integral (I_x) was converted to an interatomic distance (d_x) using the two spin approximation:[10]

$$d_x = d_{ref} \sqrt[6]{\frac{I_{ref}}{I_x}}$$

One of the cross peaks between pyrenyl protons p4/p5 or p9/p10 was chosen as reference integral (I_{ref}), assuming that the average interatomic distance between such pyrene protons -as found in pyrene-containing crystal structures- can be considered a reliable reference (i.e., d_{ref} = 2.28 Å).[11]

The obtained distances were used as constraints in an initial model that was energy-minimised using Batchmin v10.3, accessed via Maestro 9.7 and energy minimized using the MMFFs forcefield, GBSA water solvation and ±20% tolerances on the constrained distances. A few constraints which appeared unrealistically short, possibly due overlaps in the NOESY spectra, were ignored during the minimisations. Annotated spectra, distance tables and images of the structures are given in the following pages. Final coordinates are presented as .cif files accompanying this Supporting Information, with core (assigned) protons labelled as herein.

The chemical shift changes observed for GlcNAc-β-OMe (**2**) on complexation with **5**, **9** and (for comparison) **4** are listed in Table S2 below. It can be seen that all carbohydrate protons undergo substantial upfield shifts on complexation, and that these are generally much larger for pyrene-based **5** and **9**.

---

[8] Data was collected on a 600 MHz VNMRS spectrometer equipped with a 5mm cryogenically cooled probe or when further resolution was required, at 900 MHz.

[9] T. J. Mooibroek, J. M. Casas-Solvas, R. L. Harniman, C. M. Renney, T. S. Carter, M. P. Crump, A. P. Davis, *Nature Chem.* **2016**, *8*, 69.

[10] D. Neuhaus and M. P. Williamson, '*The NOE in structural and conformational analysis*', 2nd Ed., Wiley-VCH, 2000.

[11] The Cambridge Structure Database version 5.36 including updates until November 2014 contains 966 crystal structures with a pyrenyl moiety that has at least two p4/p5 or p9/p10 protons. These structures contain 1256 such interatomic distances with an average value of 2.284 ± 0.065 Å.

Table S2. Chemical shifts (δ in ppm) in 9:1 H_2O:D_2O of GlcNAc-β-OMe (**2**) unbound and when bound to receptors **4**, **5**, or **9**. The values in brackets denote the change in chemical shift (Δδ) on complex formation. All CH protons of the sugar's core are much more shielded when bound by bis-pyrenyls **5** and **9** (up to -4.73) compared to bis-biphenyl **4** (up to-2.40).

**H**	unbound **2**	biphenyl-based receptor **4** + **2**		eclipsed receptor **5** + **2**		staggered receptor **9** + **2**	
1	4.46	3.02	(- 1.44)	0.37	(- 4.09)	- 0.19	(- 4.65)
2	3.70	1.82	(- 1.88)	- 0.0	(- 3.70)	0.02	(- 3.68)
3	3.53	2.38	(- 1.15)	- 0.65	(- 4.18)	- 0.94	(- 4.47)
4	3.47	2.23	(- 1.24)	- 0.39	(- 3.86)	- 0.46	(- 3.93)
5	3.47	1.15	(- 2.32)	- 1.26	(- 4.73)	- 0.03	(- 3.50)
6	3.94	2.89	(- 1.05)	2.28	(-1.66)	2.07	(- 1.87)
6'	3.78	1.38	(- 2.40)	- 0.35	(-4.13)	- 0.51	(- 4.29)
OMe	3.53	2.57	(- 0.96)	0.73	(- 1.25)	1.59	(- 1.94)
Ac	2.06	0.20	(- 1.86)	1.56	(- 1.33)	1.04	(- 1.02)
NH	8.22	3.13	(-5.09)	2.28	(- 6.66)	3.59	(- 4.63)

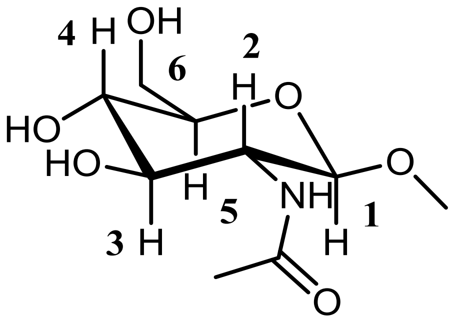

Eclipsed receptor 5 with methyl *N*-acetyl-β-D-glucosaminide (2)

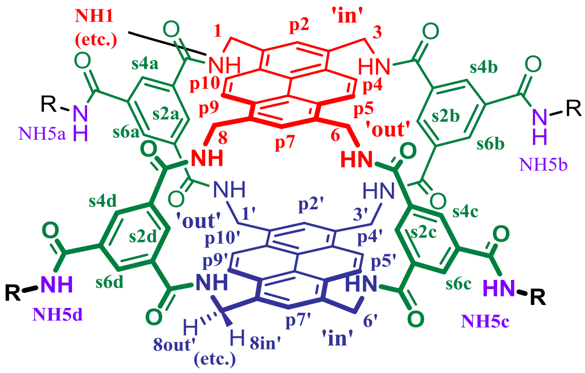

'eclipsed' **5**

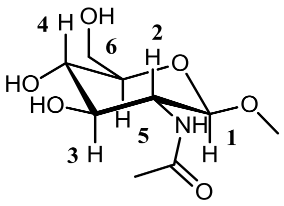

Me-β-GlcNAc **2**

Figure S68. Structures of eclipsed receptor **5** and methyl *N*-acetyl-β-D-glucosaminide with numbering using for structural assignment.

Assignment of ^{1}H NMR peaks

sx = 'spacer'

px = 'pyrene, top'

px' = 'pyrene, bottom'

Other = sugar

Type	Label	δ (ppm)	Type	Label	δ (ppm)	Type	Label	δ (ppm)
CH2	1in	4.905	CH	p10'	8.292	CH	s2a	7.428
CH2	1out	5.359	CH	p9'	8.292	CH	s4a	8.07
CH2	3in	4.668	CH	p7'	8.088	CH	s6a	8.219
CH2	3out	5.486	CH	p5'	8.184	CH	s2b	7.197
CH2	6in	4.598	CH	p4'	8.135	CH	s4b	7.997
CH2	6out	5.703	CH	p2'	7.898	CH	s6b	8.219
CH2	8in	4.88	NH	NH1	8.99	CH	s2c	8.167
CH2	8out	5.154	NH	NH3	8.839	CH	s4c	8.326
CH	p10	8.548	NH	NH6	8.414	CH	s6c	8.427
CH	p9	8.211	NH	NH8	8.181	CH	s2d	7.662
CH	p7	8.048	NH	NH1'	7.809	CH	s4d	8.266
CH	p5	8.271	NH	NH8'	7.157	CH	s6d	8.353
CH	p4	8.767	NH	NH6'	8.39	CH	1	0.372
CH	p2	8.151	NH	NH3'	9.159	CH	2	-0.003
CH2	1in'	4.844	NH	NH5a	7.949	CH	3	-0.647
CH2	1out'	5.193	NH	NH5b	7.949	CH	4	-0.385
CH2	3in'	4.669	NH	NH5c	8.01	CH	5	-1.257
CH2	3out'	5.229	NH	NH5d	8.01	CH2	6	-0.347
CH2	6in'	4.566	NH	d2NHa	-	CH2	6'	2.279
CH2	6out'	5.728	NH	d2NHb	-	CH3	Ac	0.732
CH2	8in'	4.566	NH	d2NHc	-	NH	NH	1.555
CH2	8out'	5.751	NH	d2NHd	-	CH3	OMe	2.279

2D ^{1}H NMR Spectra with assignments (600 MHz)

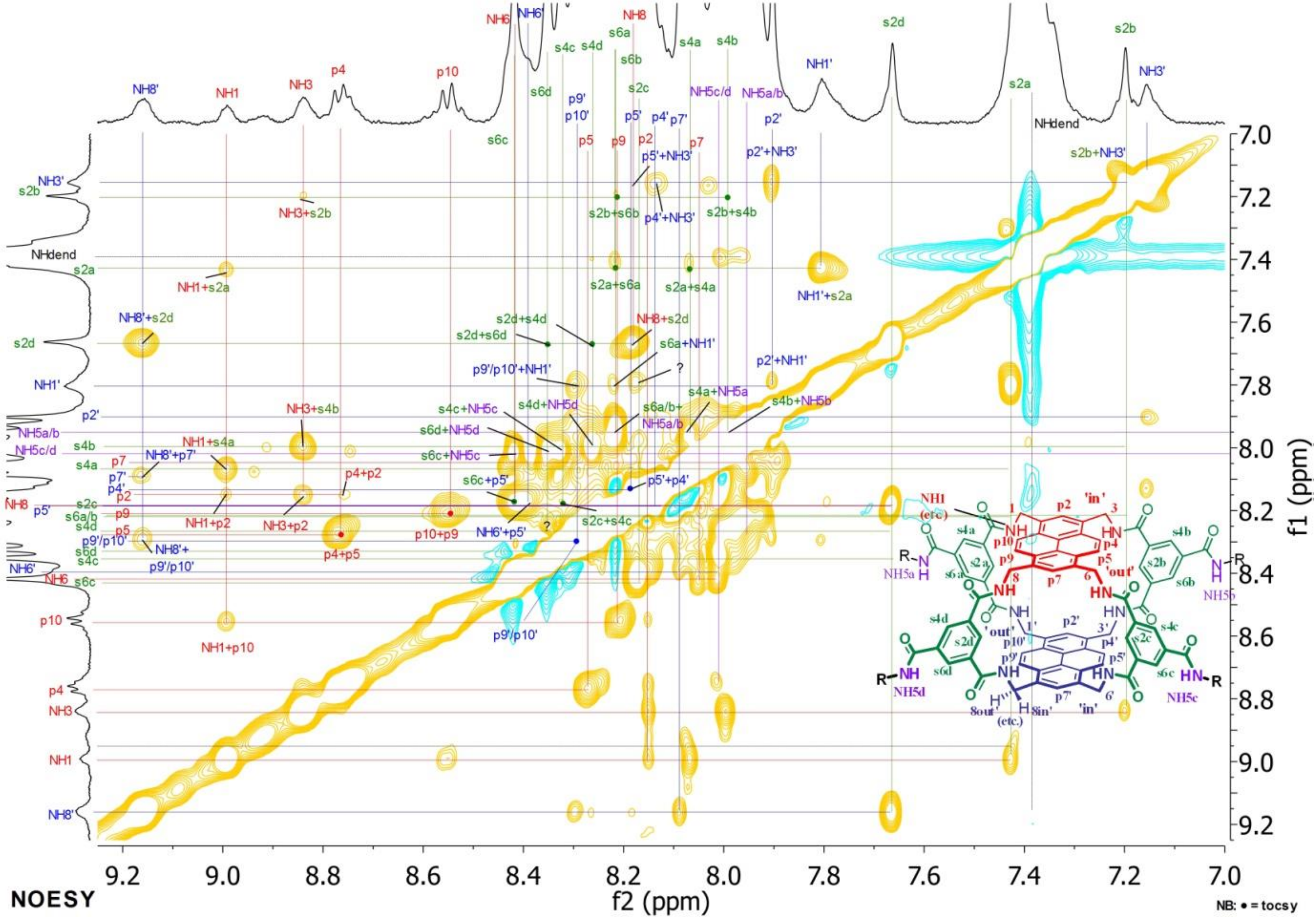

Figure S69. Partial ^{1}H NOESY NMR spectrum of the eclipsed receptor **5** (0.60 mM) complexed with methyl *N*-acetyl-β-D-glucosaminide (**2**) (10 mM) in 1:9 D_2O/H_2O at 298 K.

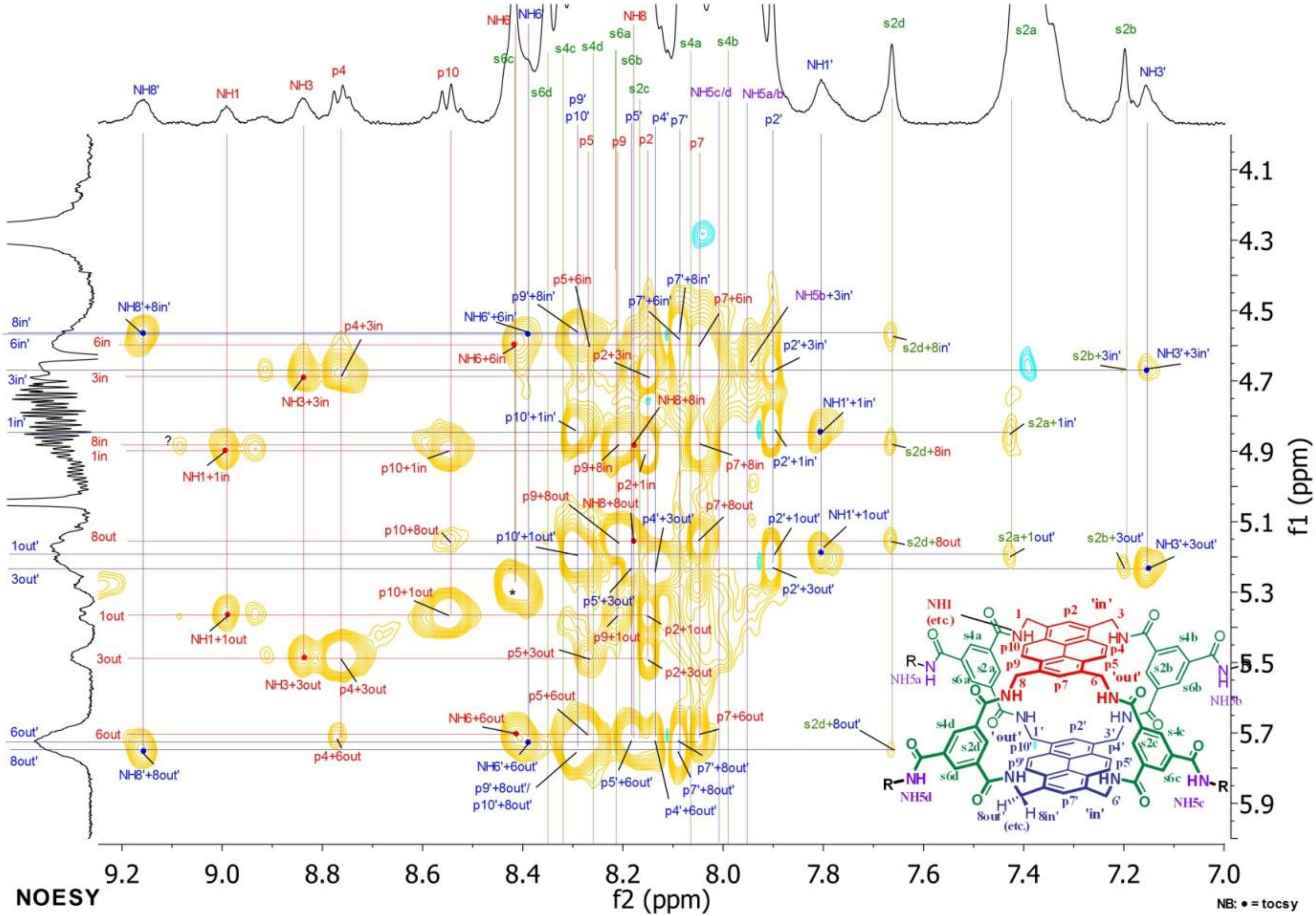

Figure S70. Partial ^{1}H NOESY NMR spectrum of the eclipsed receptor **5** (0.60 mM) complexed with methyl *N*-acetyl-β-D-glucosaminide (**2**) (10 mM) in 1:9 D_2O/H_2O at 298 K.

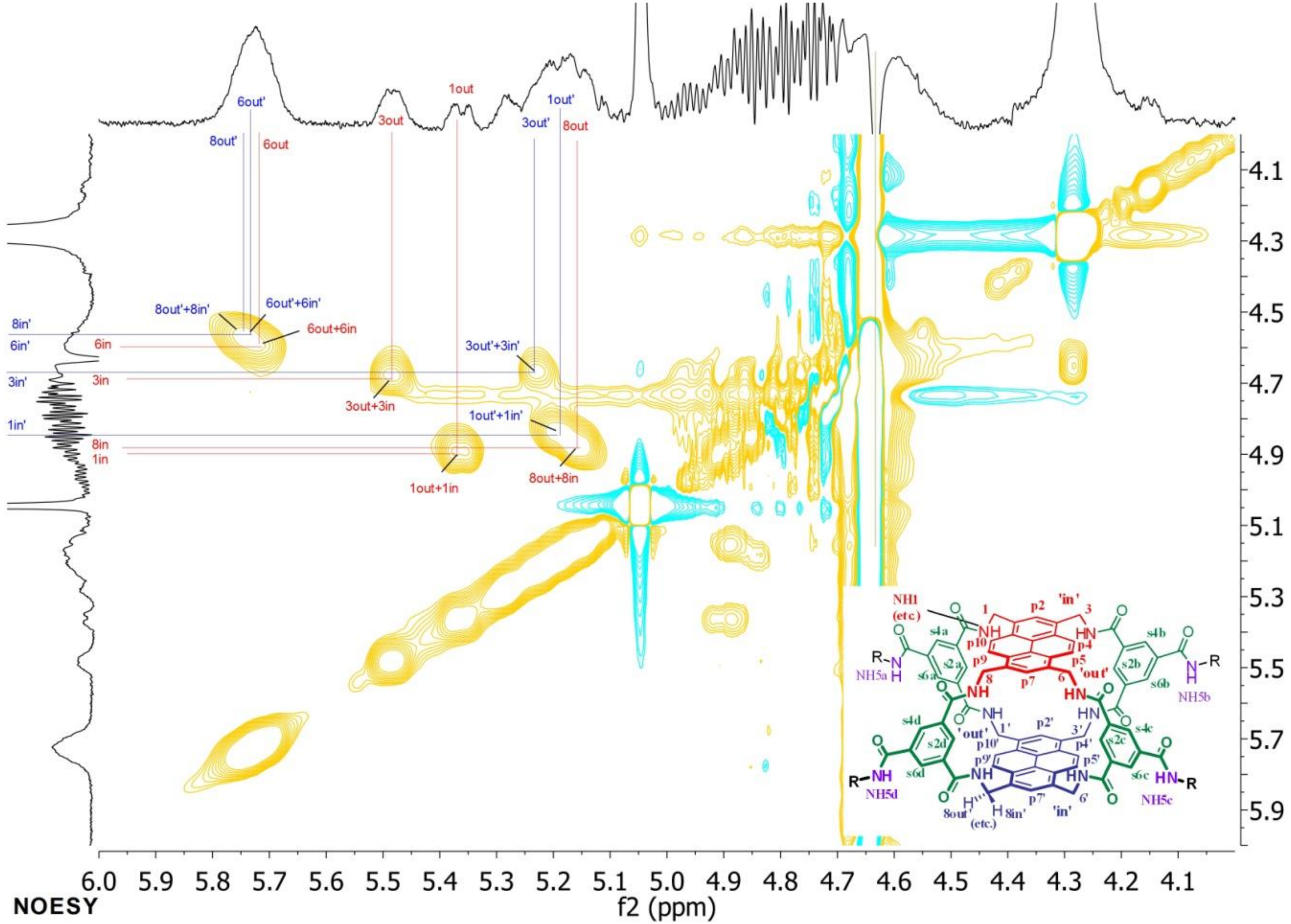

Figure S71. Partial ^{1}H NOESY NMR spectrum of the eclipsed receptor **5** (0.60 mM) complexed with methyl *N*-acetyl-β-D-glucosaminide (**2**) (10 mM) in 1:9 D_2O/H_2O at 298 K.

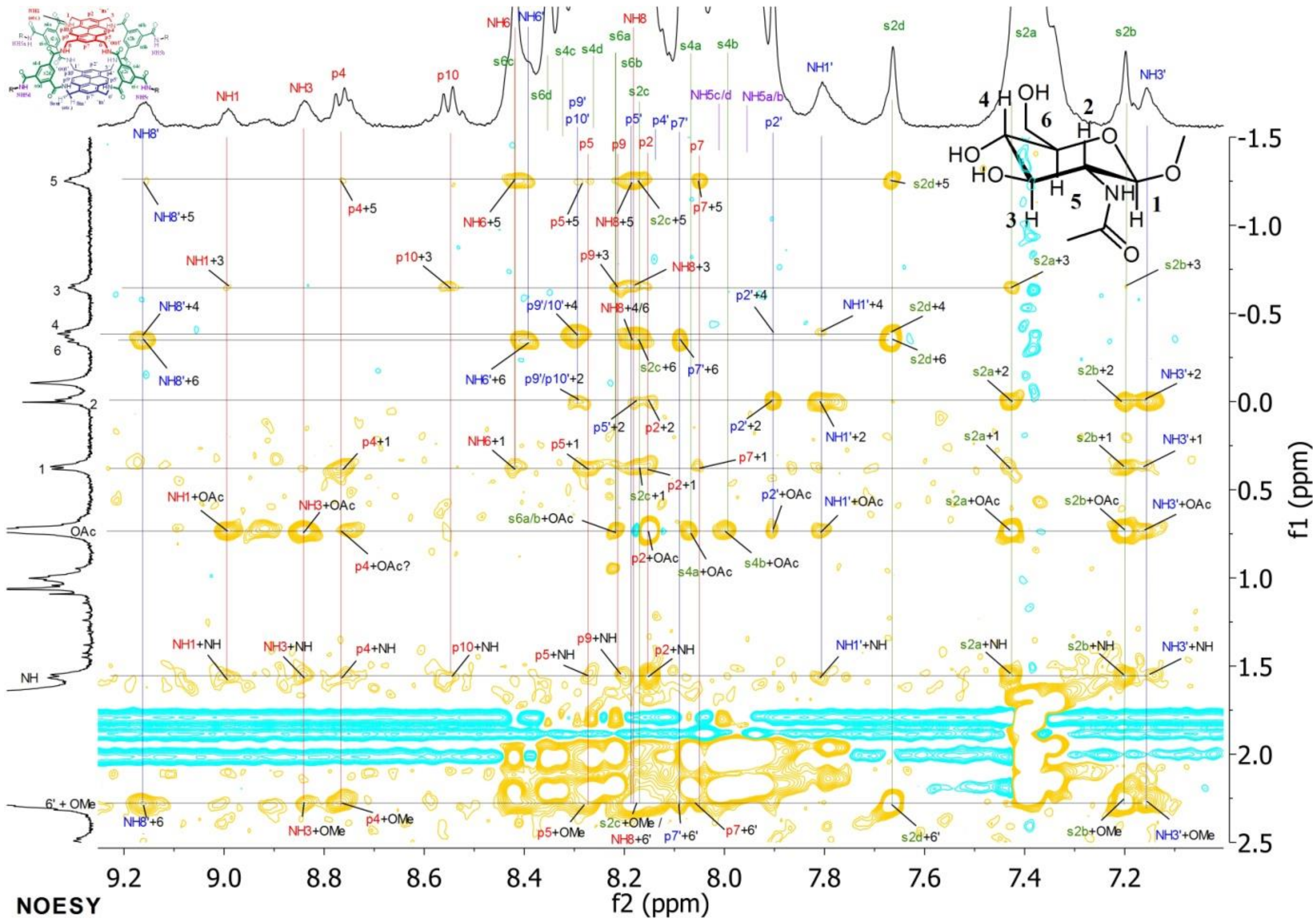

Figure S72. Partial ^{1}H NOESY NMR spectrum of the eclipsed receptor **5** (0.60 mM) complexed with methyl *N*-acetyl-β-D-glucosaminide (**2**) (10 mM) in 1:9 D_2O/H_2O at 298 K.

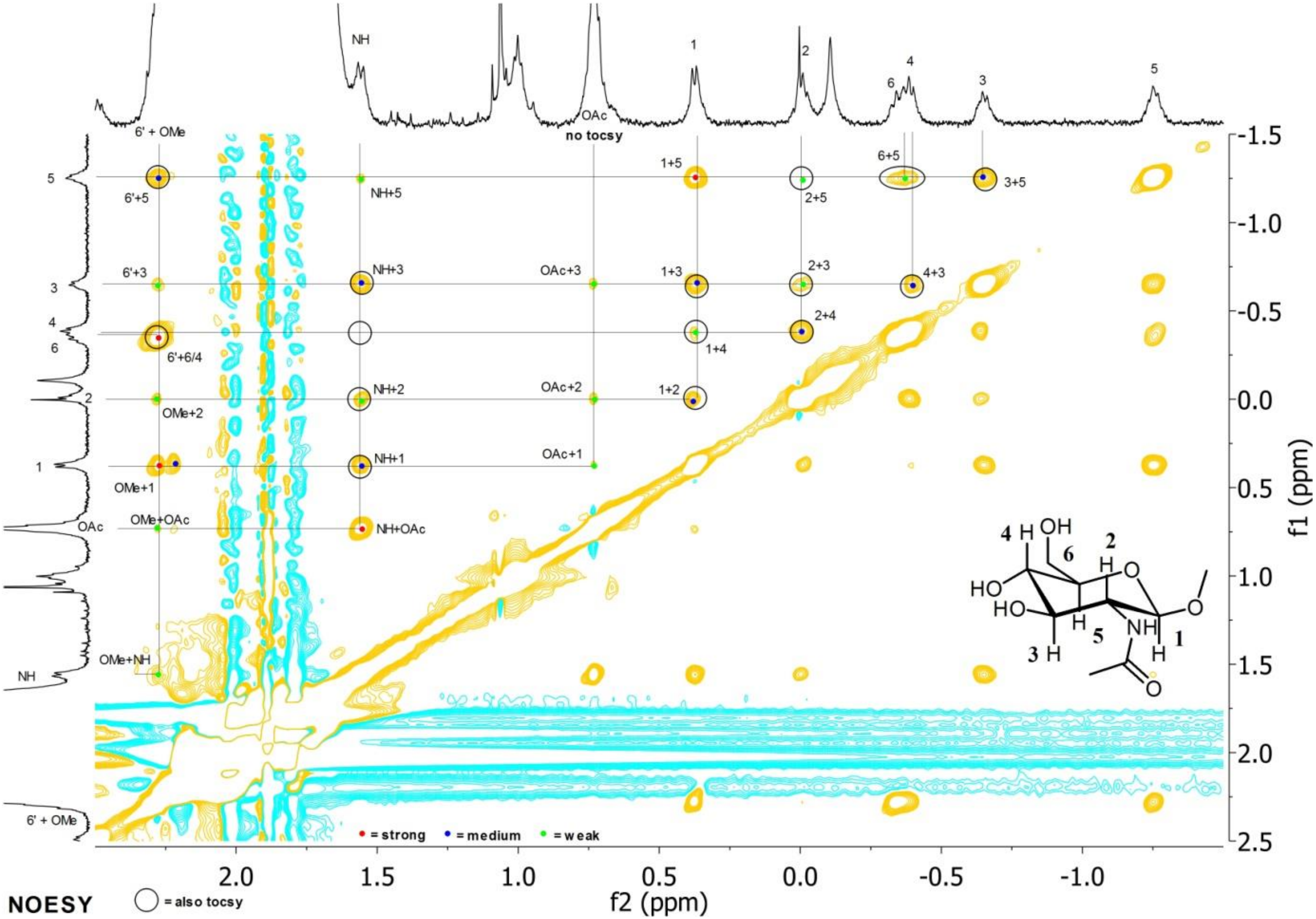

Figure S73. Partial ^{1}H NOESY NMR spectrum of the eclipsed receptor **5** (0.60 mM) complexed with methyl *N*-acetyl-β-D-glucosaminide (**2**) (10 mM) in 1:9 D_2O/H_2O at 298 K.

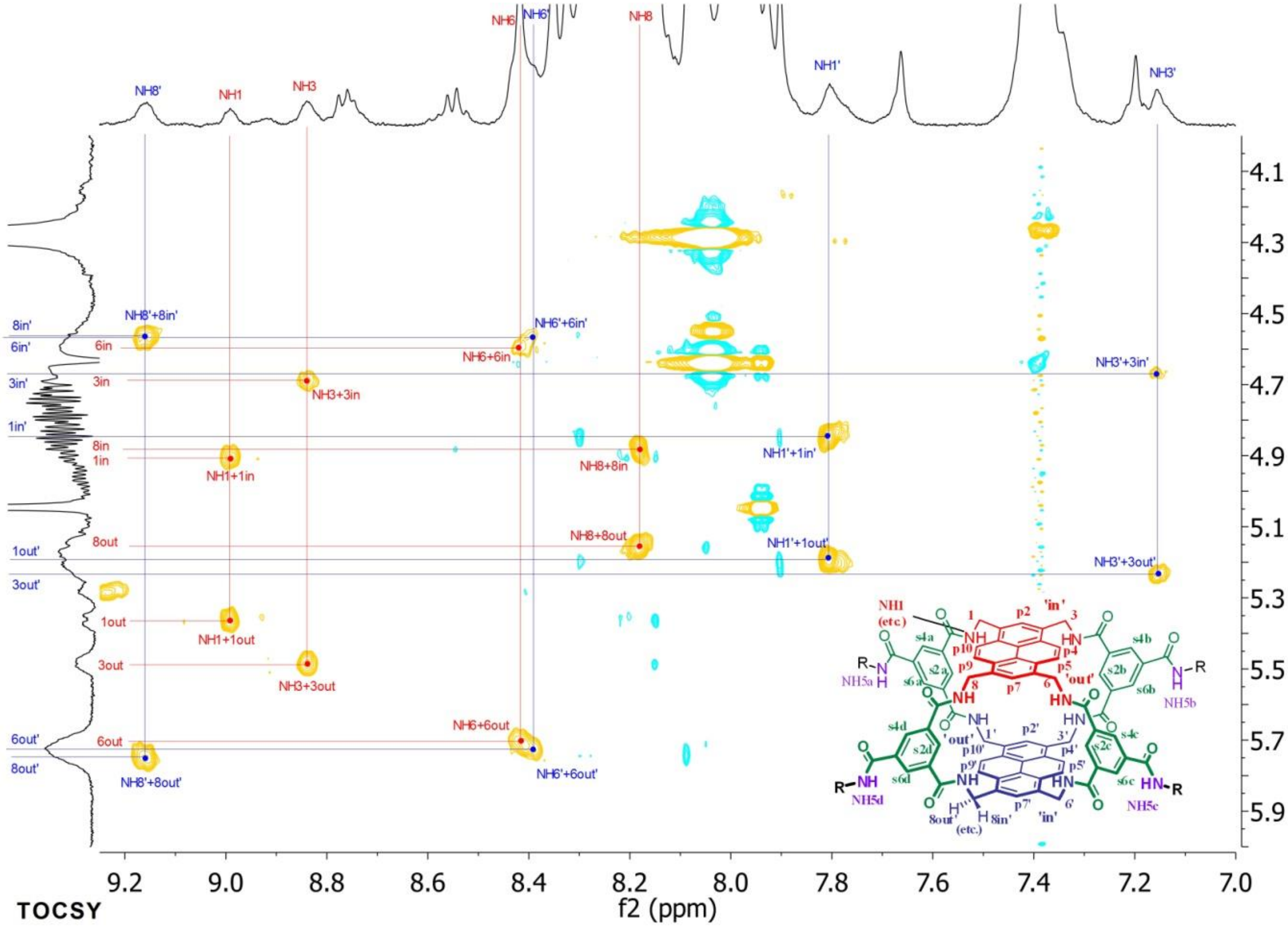

Figure S74. Partial ^{1}H TOCSY NMR spectrum of the eclipsed receptor **5** (0.60 mM) complexed with methyl *N*-acetyl-β-D-glucosaminide (**2**) (10 mM) in 1:9 D_2O/H_2O at 298 K.

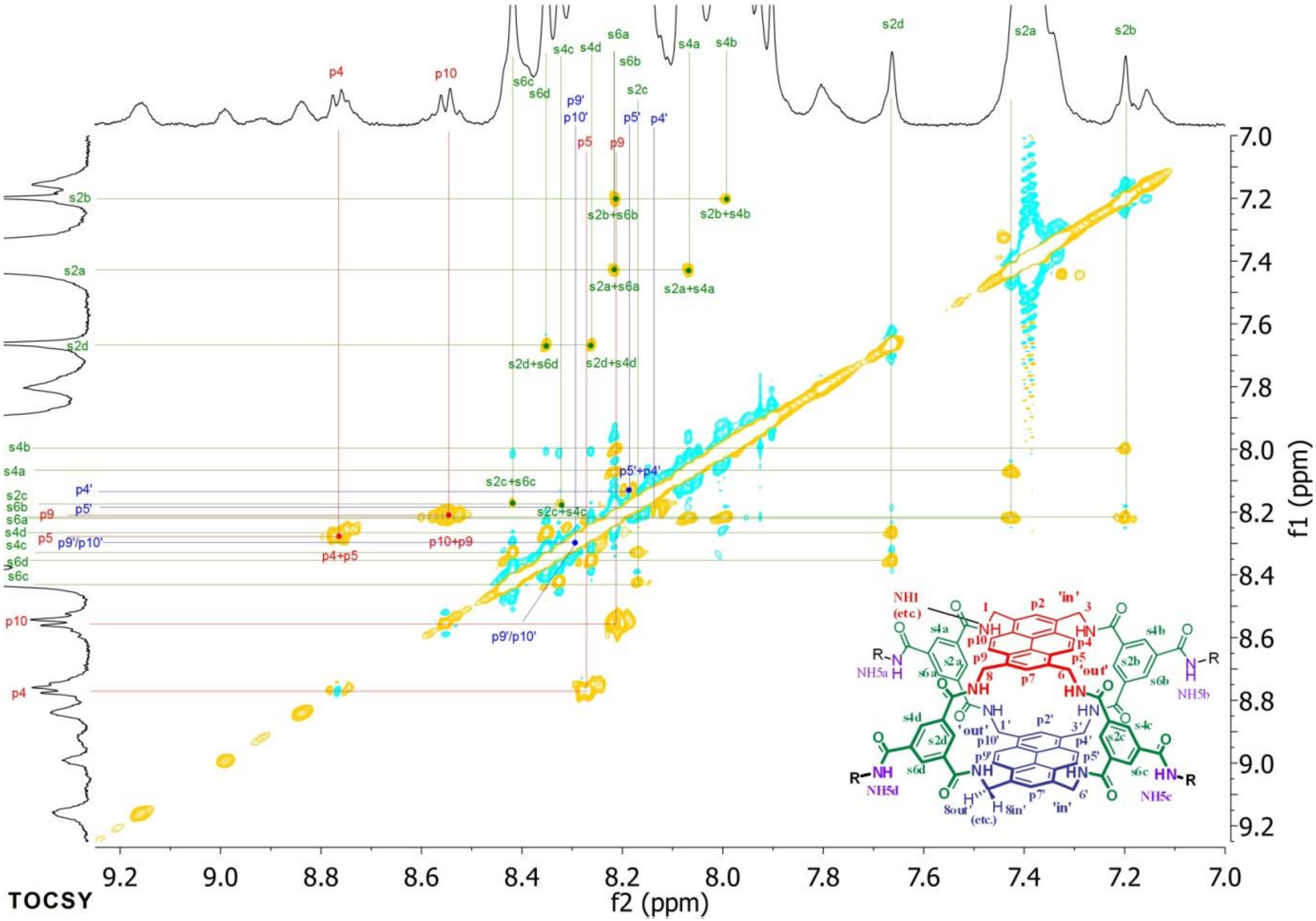

Figure S75. Partial ^{1}H TOCSY NMR spectrum of the eclipsed receptor **5** (0.60 mM) complexed with methyl *N*-acetyl-β-D-glucosaminide (**2**) (10 mM) in 1:9 D_2O/H_2O at 298 K.

Distances obtained from NOE data and subsequent molecular modeling

73 Distances RMSD = 0.461 Å
Receptor 5 + GlcNAc-β-OMe (2)

Labels		Distance (A)	
**f2**	**f1**	**nOe**	**model**
5	NH6	3.987	3.208
5	p5	5.133	4.177
5	p7	4.482	3.776
5	s2d	4.300	4.494
NH1	s2a	3.954	4.339
NH1	p10	3.649	4.451
NH1	1in	2.877	2.256
NH1	1out	3.072	2.867
NH1	3	5.703	5.399
NH1	Ac'	3.777	3.013
NH1	NH	4.475	4.248
NH1'	1in'	2.906	2.785
NH1'	1out'	2.906	2.879
NH1'	4	5.445	5.090
NH1'	2	3.696	3.290
NH1'	Ac'	4.441	3.674
NH1'	NH	4.770	4.716
NH3	s2b	4.108	4.125
NH3	3in	2.773	2.298
NH3	3out	2.825	2.949
NH3	Ac	3.378	2.806
NH3	NH	4.379	3.926
NH3	OMe	4.361	5.343
NH3'	2	3.654	3.591
NH3'	1	5.295	4.906
NH3'	Ac''	4.230	3.360
NH3'	NH	4.573	4.440
NH3'	OMe'	3.643	3.752
NH6	1	4.428	4.263
NH6'	6	3.865	3.027
NH8'	s2d	2.887	2.291
NH8'	p7'	3.767	3.844
NH8'	p9'	3.528	2.737
NH8'	8in'	2.764	2.902
NH8'	8out'	2.838	2.776
NH8'	6'	3.588	3.211
p10	1out	2.150	2.025
p10	NH	4.259	4.643
p2	3in	2.280	2.567
p2	1in	2.300	2.496
p2	Ac	2.905	2.636
p2	NH	3.620	3.543
p2'	3in'	2.459	2.351
p2'	1in'	2.299	2.396
p2'	Ac''	4.669	5.053
p4	5	5.741	5.028
p4	1	3.925	3.594
p4	NH	4.759	4.298
p4	OMe	3.704	3.235
p4'	NH3'	3.336	3.375
p5	1	4.089	3.731
p5'	2	5.031	5.333
p7	8in	2.188	2.319
p7	1	5.133	5.712
p7'	6	4.012	3.153
s2a	3	4.770	4.180
s2a	2	3.993	3.777
s2a	Ac'	3.260	3.605
s2a	NH	3.790	4.277
s2b	3in'	4.410	4.741
s2b	3out'	3.743	4.385
s2b	3	6.281	5.922
s2b	2	4.104	4.241
s2b	1	4.115	4.022
s2b	Ac''	3.240	3.226
s2b	NH	3.682	3.810
s2b	OMe'	2.960	2.631
S2c	OMe''	2.841	2.691
s2d	8in'	3.644	4.405
s2d	8out'	4.136	4.809
s2d	6'	2.979	2.418
s4a	Ac'	4.139	3.853
s4b	Ac''	4.026	4.370

Images of molecular model of 5 + GlcNAc-β-OMe (2)

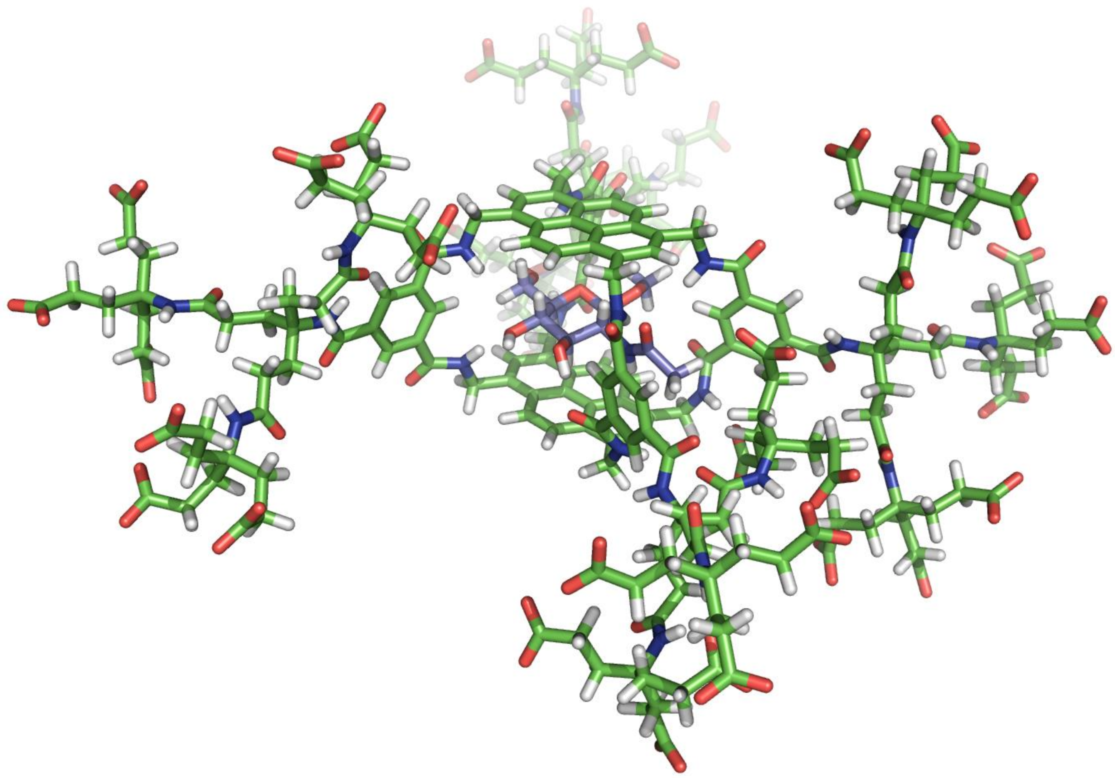

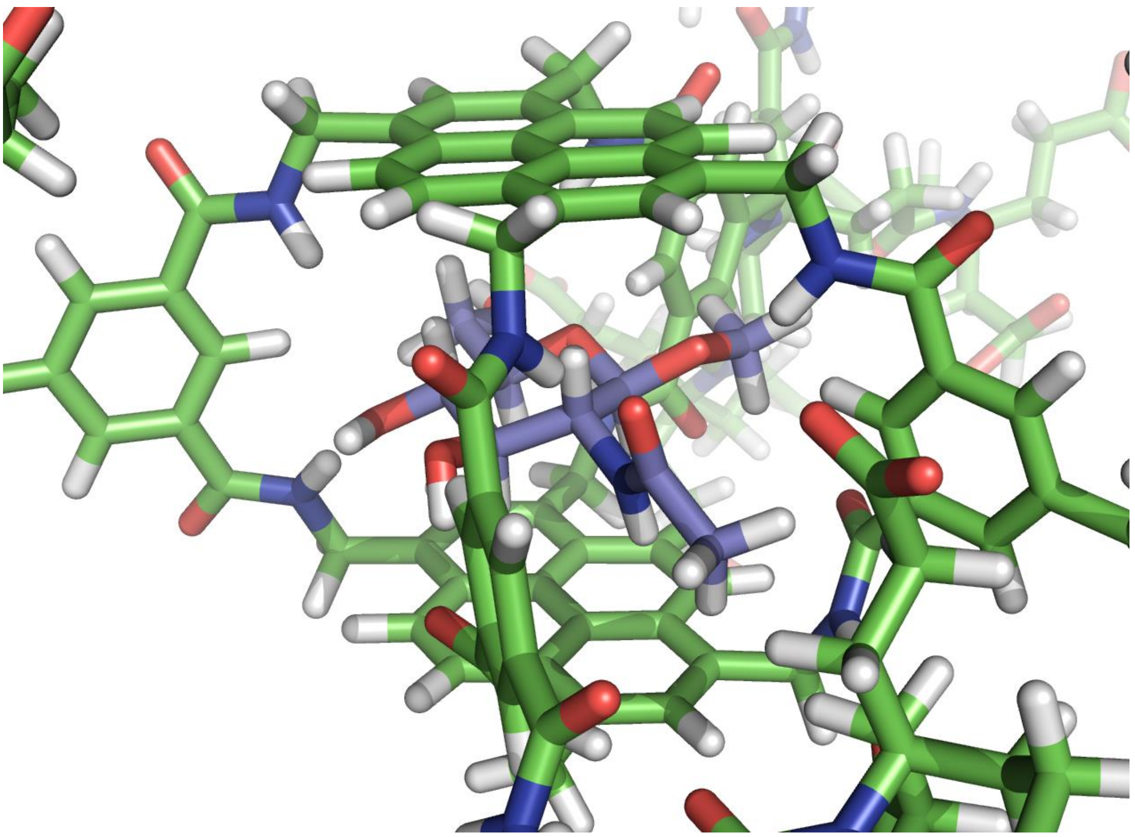

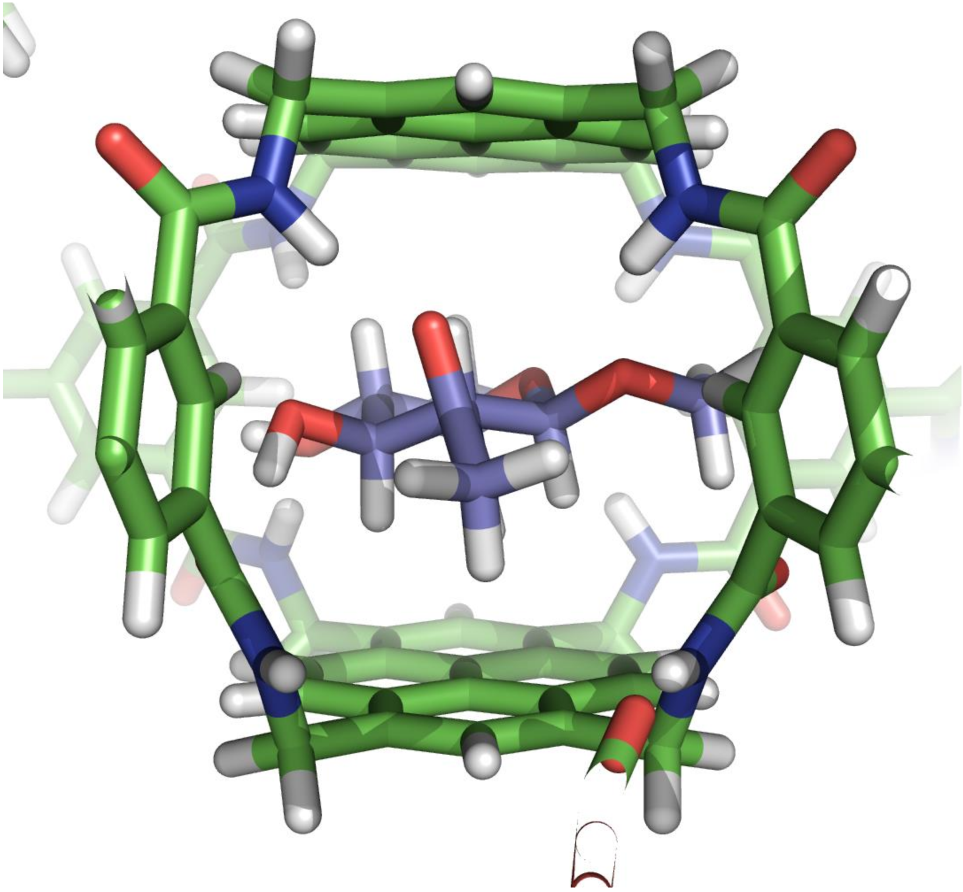

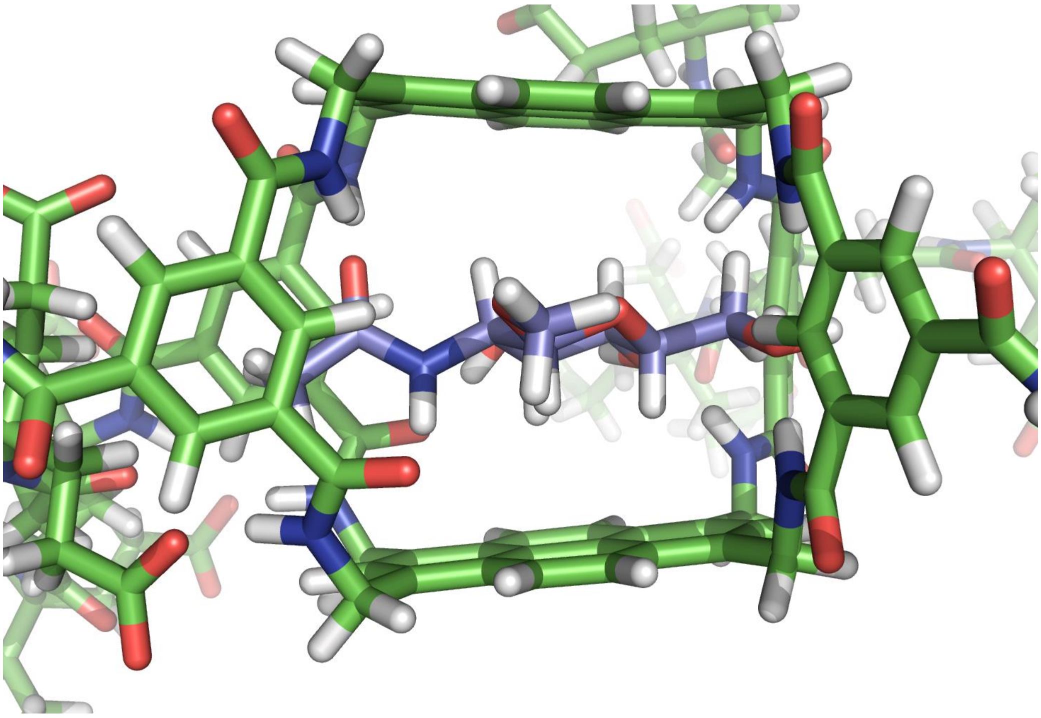

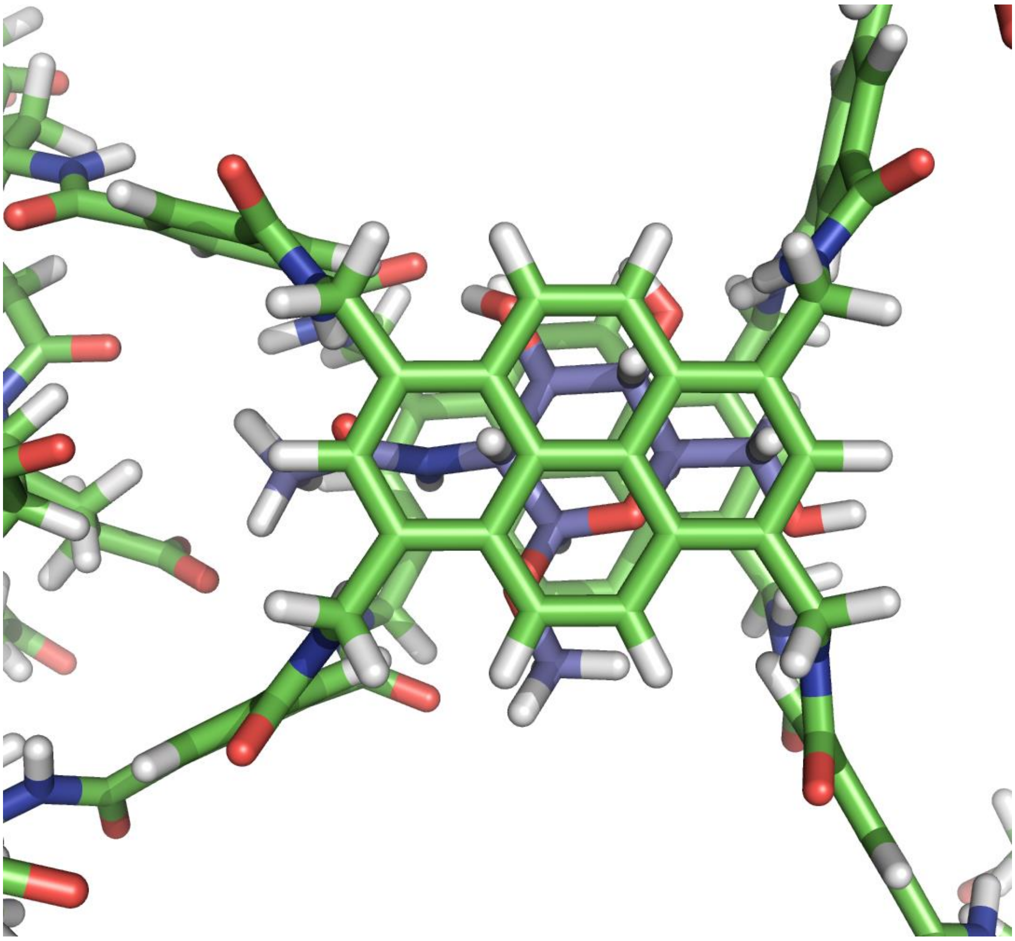

Eclipsed receptor 5 with glycopeptide 3

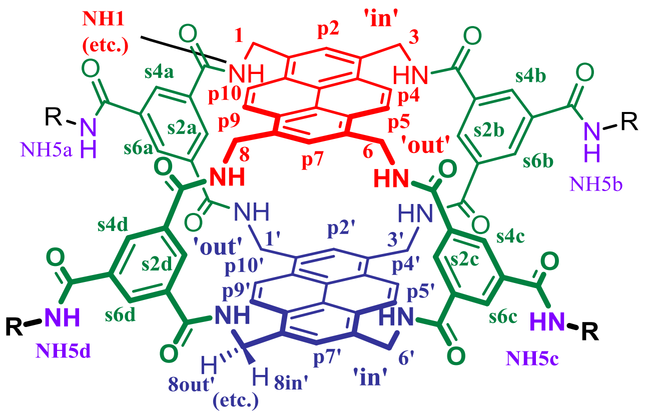

'eclipsed' **5**

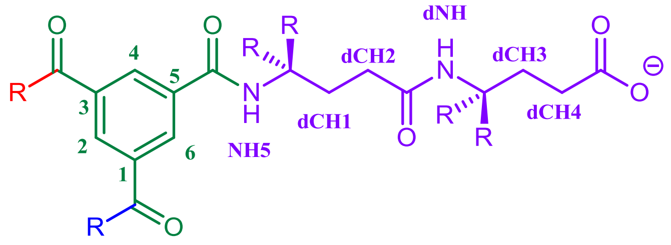

Dendrimer

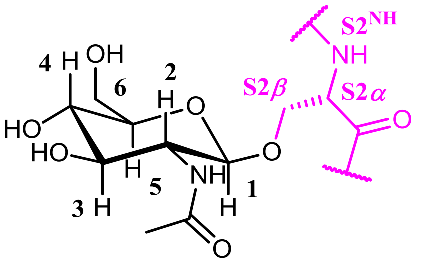

Glycopeptide **3**

Figure S76. Structures of eclipsed receptor **5** and the sugar fragment of glycopeptide **3** with attached residue. The numbering used for structural assignment is also given.

Assignment of ^{1}H NMR peaks

sx = 'spacer'
px = 'pyrene, top'
px' = 'pyrene, bottom'
Other = sugar

Type	Label	δ (ppm)	Type	Label	δ (ppm)	Type	Label	δ (ppm)
CH2	1in	4.643	NH	NH1	9.214	CH	2	0.097
CH2	1out	5.764	NH	NH3	8.528	CH	3	-0.773
CH2	3in	4.51	NH	NH6	7.9541	CH	4	-0.449
CH2	3out	6.065	NH	NH8	7.801	CH	5	-1.136
CH2	6in	4.728	NH	NH1'	8.185	CH2	6	0.171
CH2	6out	4.9	NH	NH8'	8.365	CH2	6'	2.553
CH2	8in	4.827	NH	NH6'	9.746	CH3	Ac	0.592
CH2	8out	5.279	NH	NH3'	8.557	NH	NH	1.538
CH	p10	8.362	NH	NH5a	8.074	CH3	OMe	-
CH	p9	8.336	NH	NH5b	7.993	CH	Ser2a	7.366
CH	p7	7.954	NH	NH5c	7.954	CH2	Ser2b	6.95
CH	p5	7.82	NH	NH5d	8.009	NH	Ser2NH	7.298
CH	p4	8.488	NH	d2NHa	7.447	CH2	dCH1a	2.08
CH	p2	8.13	NH	d2NHb	7.456	CH2	dCH2a	2.267
CH2	1in'	5.01	NH	d2NHc	7.45	CH2	dCH3a	1.845
CH2	1out'	5.177	NH	d2NHd	7.429	CH2	dCH4a	2.112
CH2	3in'	4.502	CH	s2a	7.766	CH2	dCH1b	2.08
CH2	3out'	5.821	CH	s4a	8.316	CH2	dCH2b	2.267
CH2	6in'	4.543	CH	s6a	8.44	CH2	dCH3b	1.845
CH2	6out'	5.693	CH	s2b	8.171	CH2	dCH4b	2.112
CH2	8in'	5.028	CH	s4b	8.415	CH2	dCH1c	2.047
CH2	8out'	5.303	CH	s6b	8.502	CH2	dCH2c	2.222
CH	p10'	8.274	CH	s2c	7.556	CH2	dCH3c	1.85
CH	p9'	8.458	CH	s4c	8.169	CH2	dCH4c	2.08
CH	p7'	8.154	CH	s6c	8.037	CH2	dCH1d	2.026
CH	p5'	9.058	CH	s2d	7.225	CH2	dCH2d	2.222
CH	p4'	8.143	CH	s4d	8.253	CH2	dCH3d	1.829
CH	p2'	8.113	CH	s6d	8.106	CH2	dCH4d	2.08
NH	NH1	9.214	CH	1	0.631			

2D ^{1}H NMR Spectra with assignments (900 MHz)

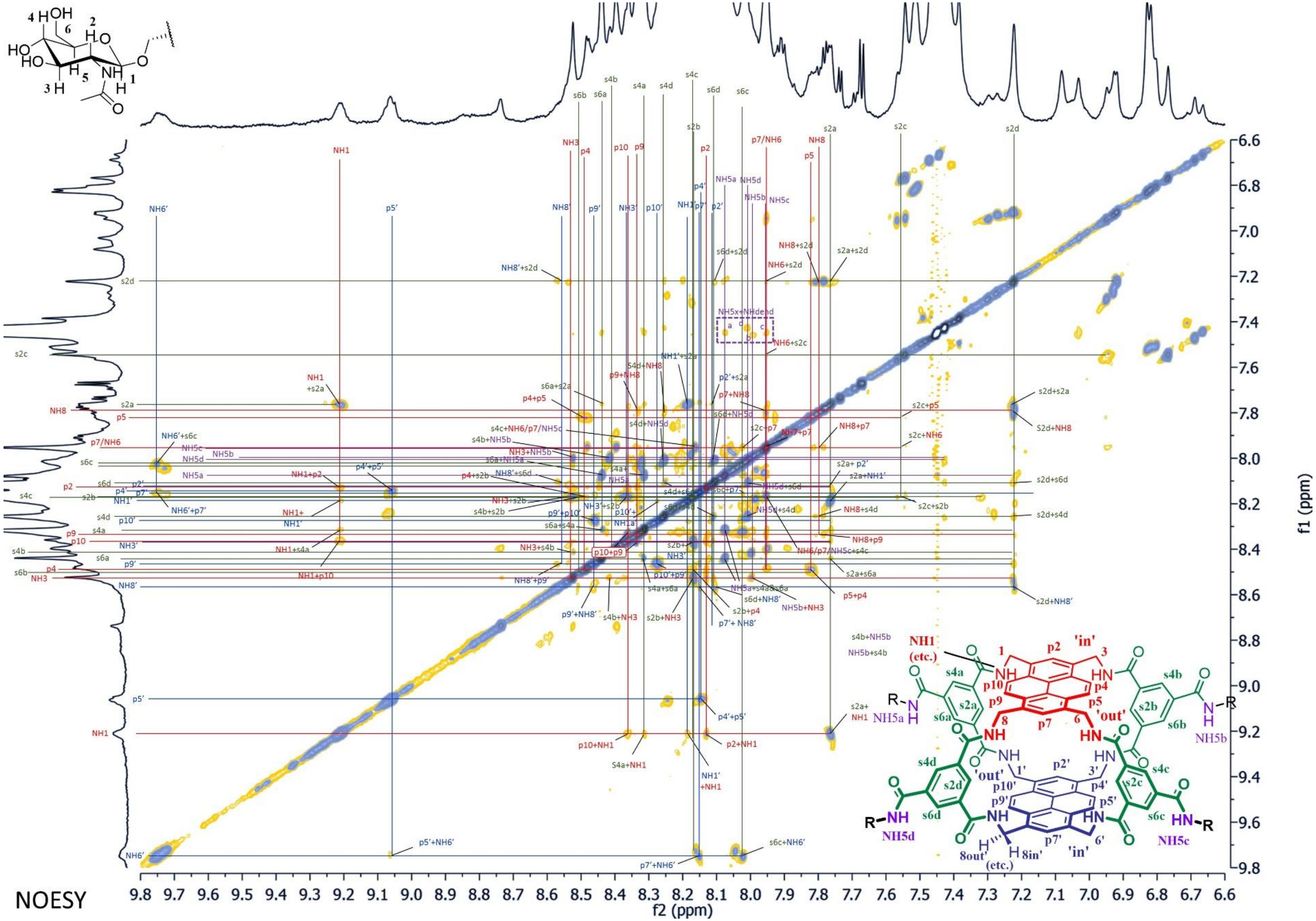

Figure S77. Partial ^{1}H NOESY NMR spectrum of the eclipsed receptor **5** (0.41 mM) complexed with glycopeptide **3** (1.57 mM) in 1:9 D_2O/H_2O at 298 K.

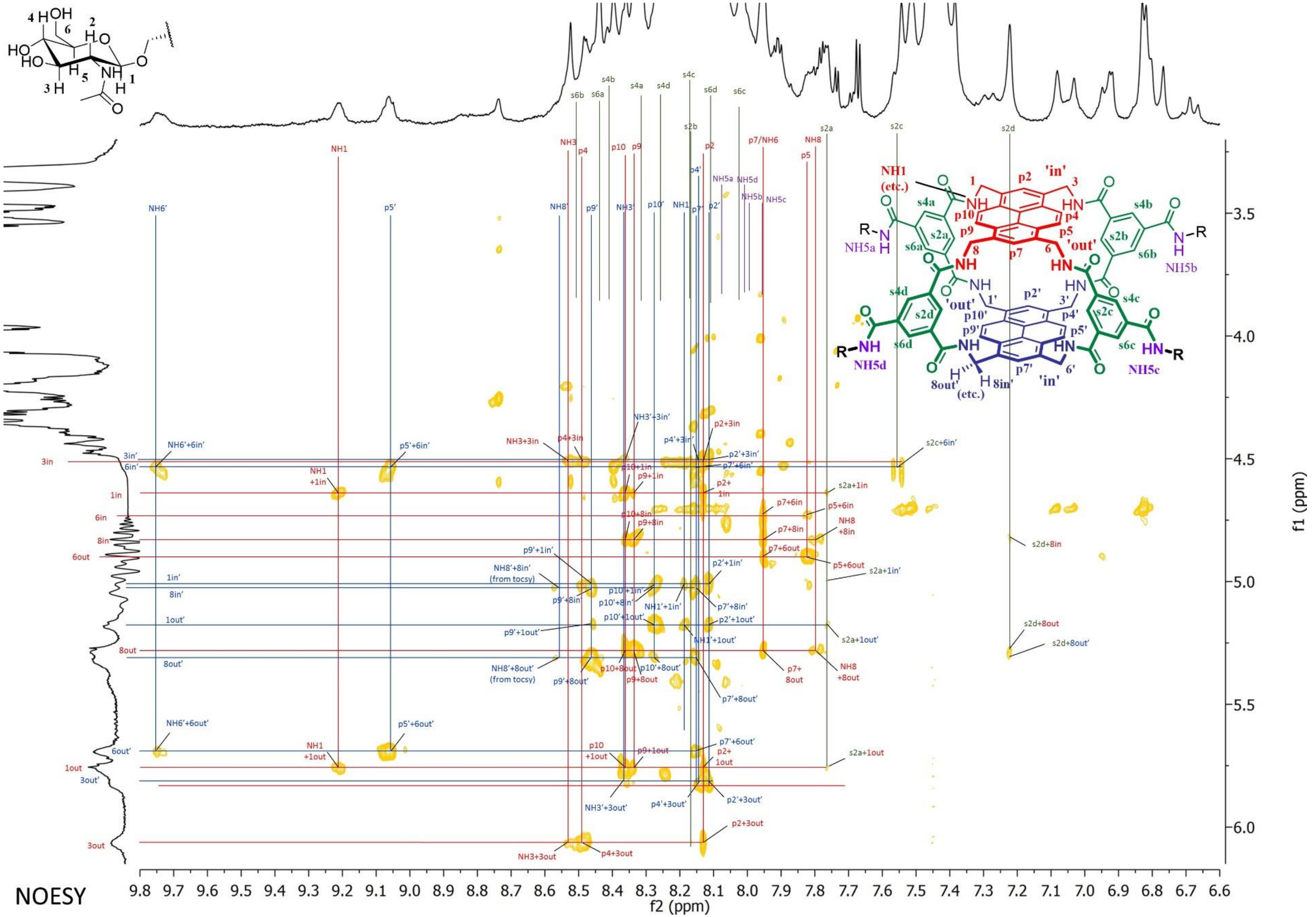

Figure S78. Partial ^{1}H NOESY NMR spectrum of the eclipsed receptor **5** (0.41 mM) complexed with glycopeptide **3** (1.57 mM) in 1:9 D_2O/H_2O at 298 K.

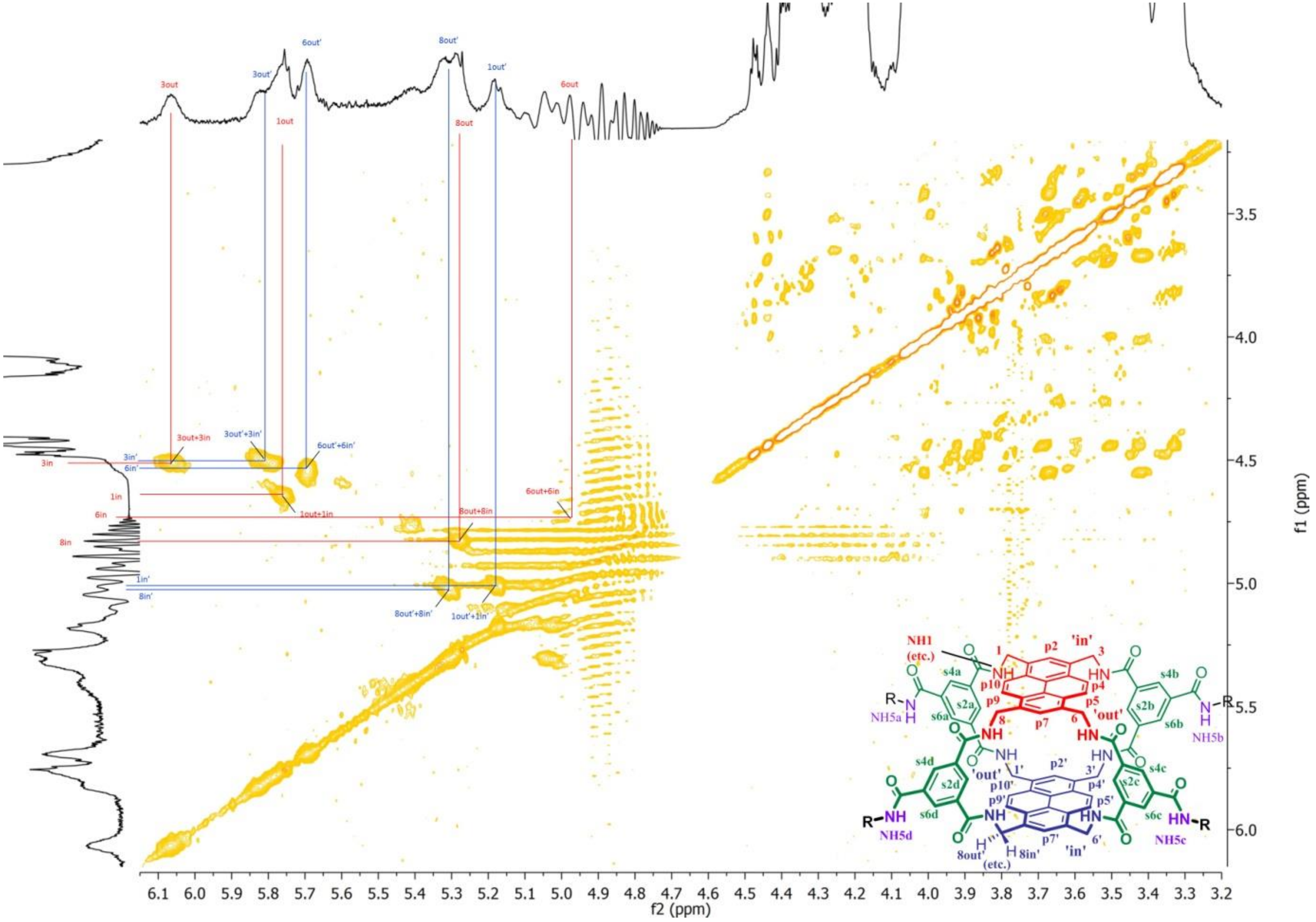

Figure S79. Partial ^1H NOESY NMR spectrum of the eclipsed receptor **5** (0.41 mM) complexed with glycopeptide **3** (1.57 mM) in 1:9 D_2O/H_2O at 298 K.

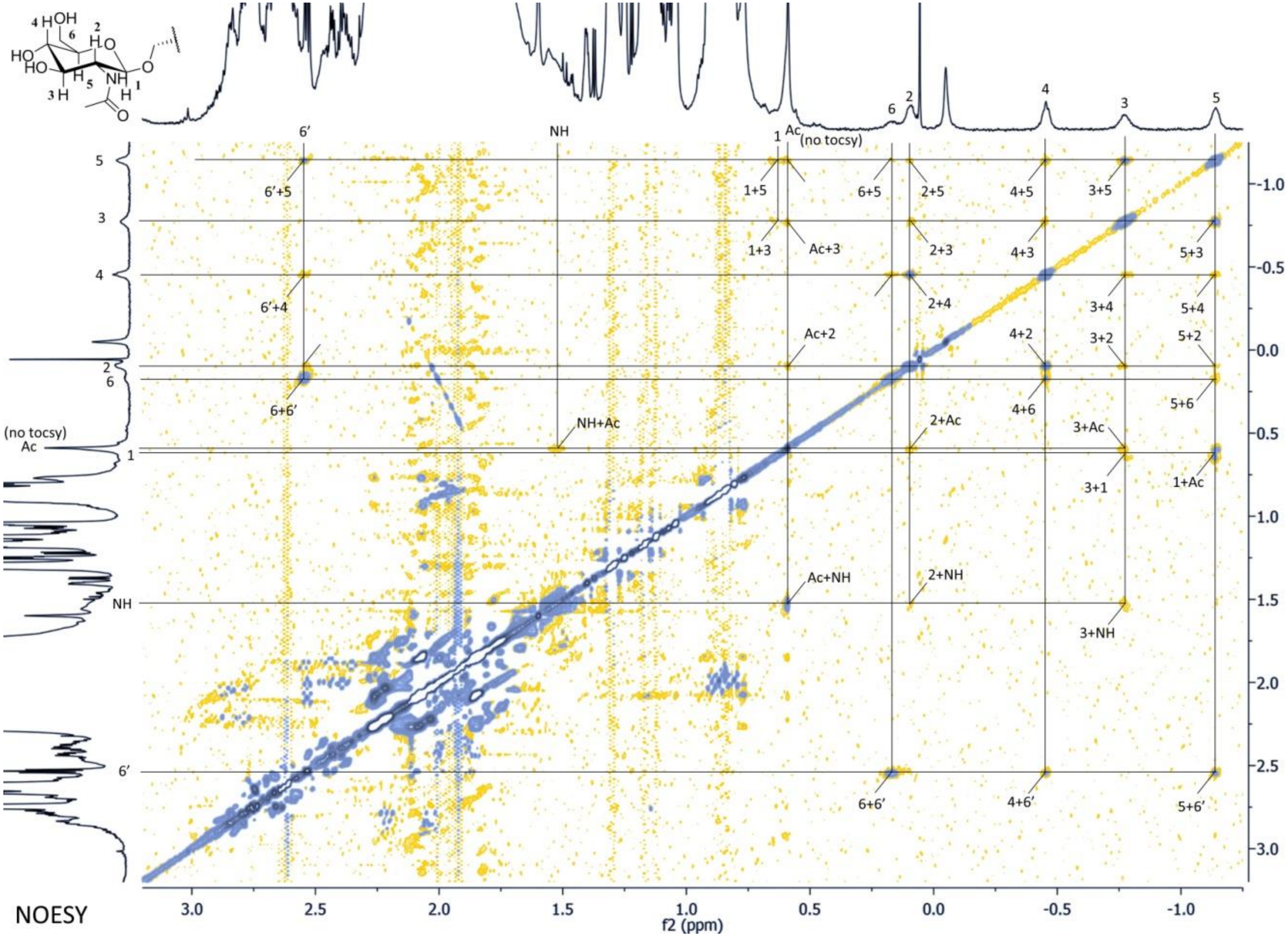

Figure S80. Partial ^{1}H NOESY NMR spectrum of the eclipsed receptor **5** (0.41 mM) complexed with glycopeptide **3** (1.57 mM) in 1:9 D_2O/H_2O at 298 K.

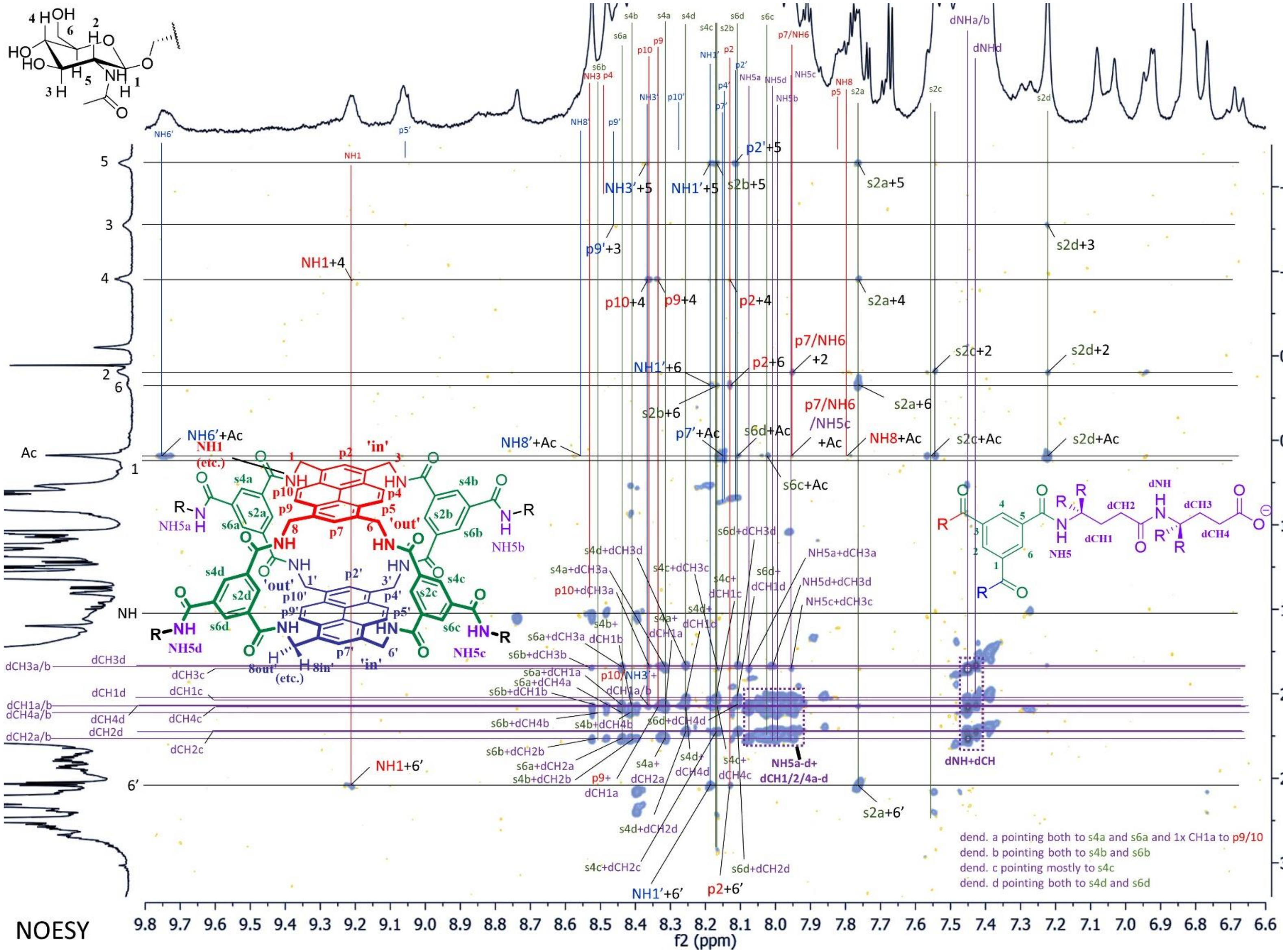

Figure S81. Partial ^{1}H NOESY NMR spectrum of the eclipsed receptor **5** (0.41 mM) complexed with glycopeptide **3** (1.57 mM) in 1:9 D_2O/H_2O at 298 K.

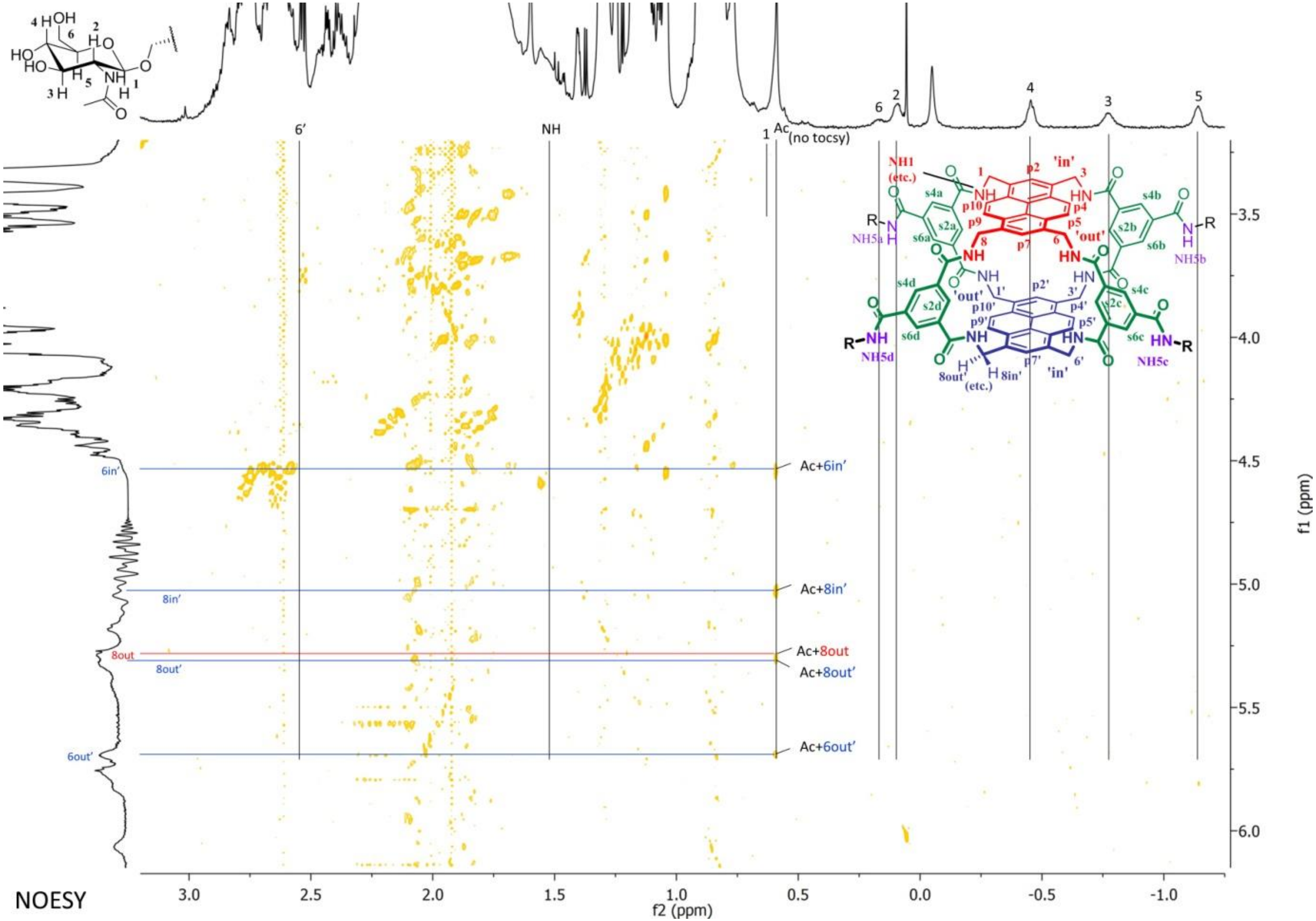

Figure S82. Partial ^{1}H NOESY NMR spectrum of the eclipsed receptor **5** (0.41 mM) complexed with glycopeptide **3** (1.57 mM) in 1:9 D_2O/H_2O at 298 K.

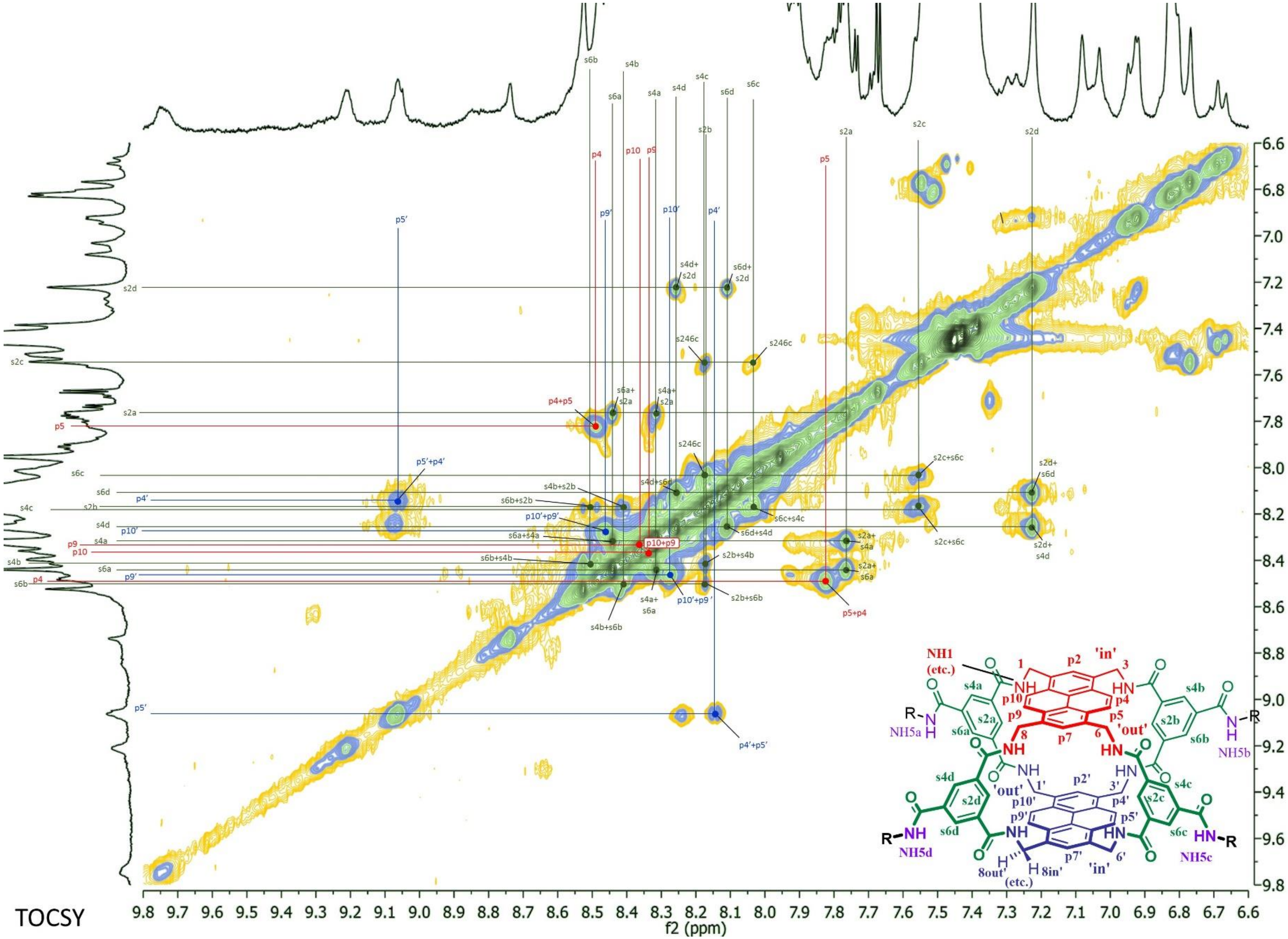

Figure S83. Partial ^1H TOCSY NMR spectrum of the eclipsed receptor **5** (0.41 mM) complexed with glycopeptide **3** (1.57 mM) in 1:9 D_2O/H_2O at 298 K.

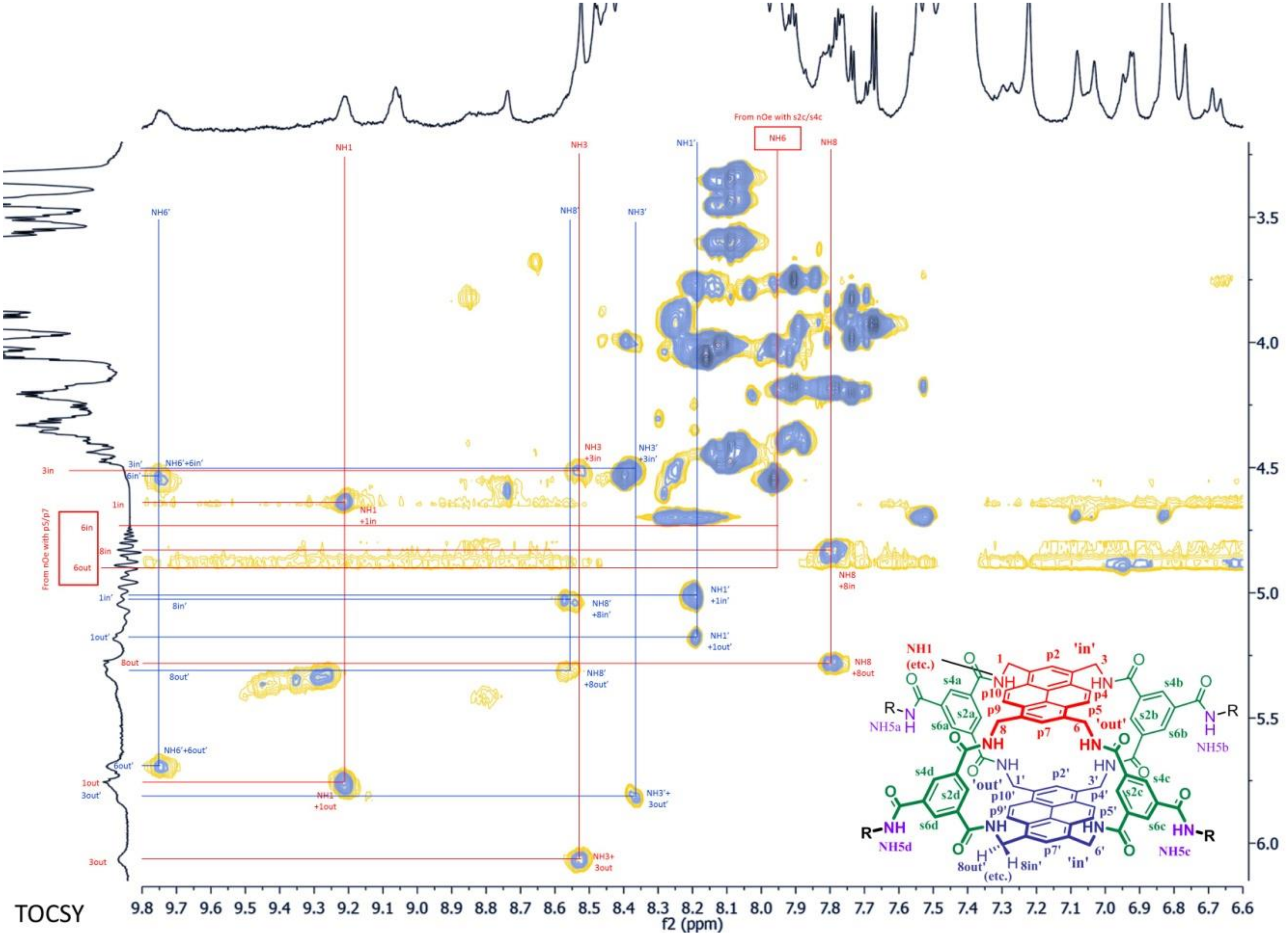

Figure S84. Partial ^{1}H TOCSY NMR spectrum of the eclipsed receptor **5** (0.41 mM) complexed with glycopeptide **3** (1.57 mM) in 1:9 D_2O/H_2O at 298 K.

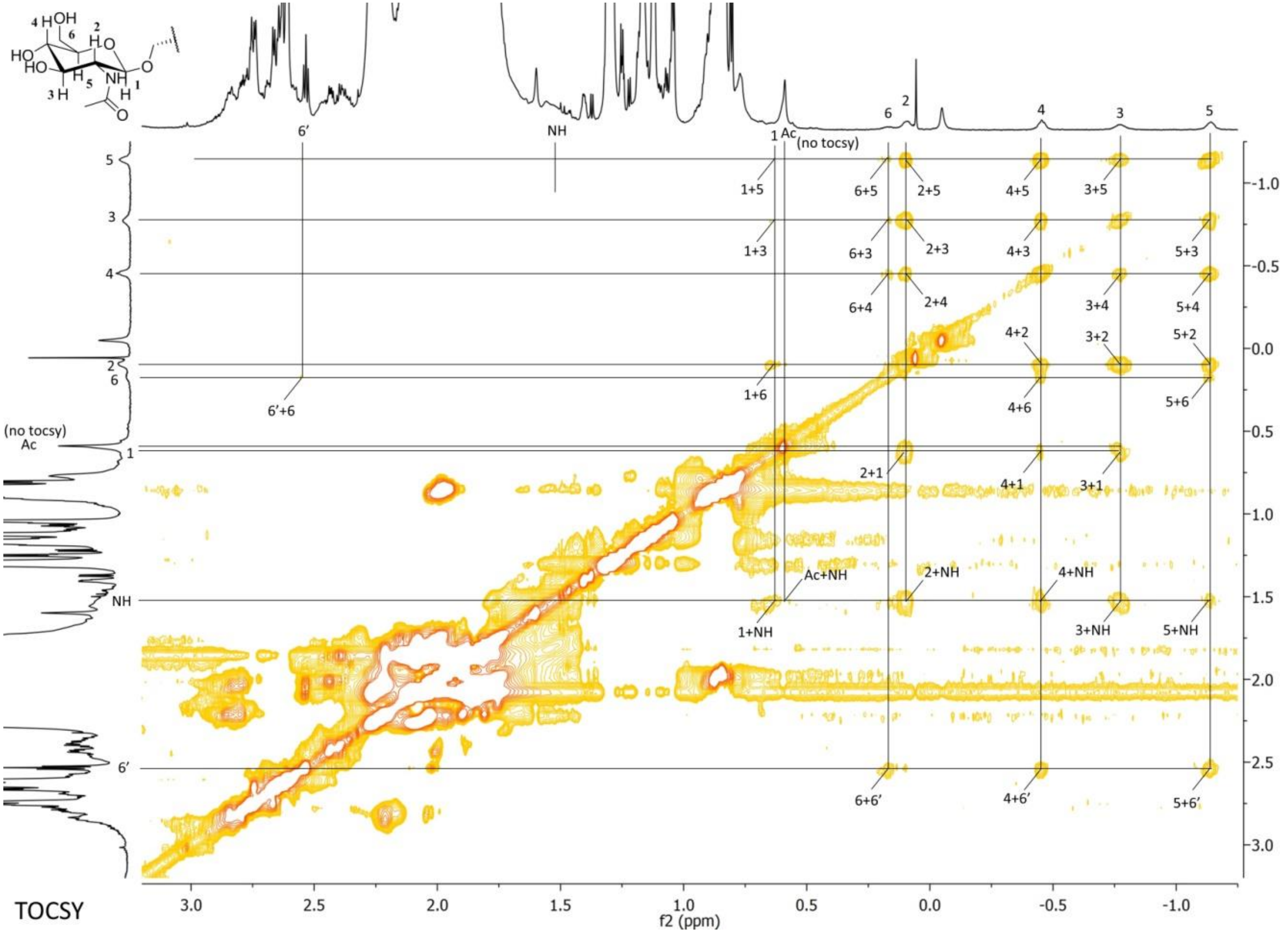

Figure S85. Partial ^{1}H TOCSY NMR spectrum of the eclipsed receptor **5** (0.41 mM) complexed with glycopeptide **3** (1.57 mM) in 1:9 D_2O/H_2O at 298 K.

Distances obtained from NOE data and subsequent molecular modeling

81 Distances RMSD = 0.503 Å
Receptor 5 + Glycopeptide 3

Labels		Distance (A)	
f2	f1	nOe	model
NH1	p10	2.859	3.559
NH1	NH1'	3.656	4.244
NH1	p2	2.933	3.061
NH1	s2a	2.271	2.049
NH1	1out	2.530	2.956
NH1	1in	2.447	2.500
NH1	6'	2.956	2.560
NH1	4	4.028	3.725
NH1'	s2a	2.093	2.328
NH1'	1out'	2.628	2.725
NH1'	1in'	2.842	2.981
NH1'	5	3.190	2.784
NH1'	6	3.582	2.726
NH1'	6'	2.739	3.753
NH3	s4b	4.181	4.395
NH3	s2b	2.262	2.267
NH3	3out	2.531	2.908
NH3	3in	2.463	2.786
NH3'	s2b	2.152	2.414
NH3'	3in'	3.077	2.599
NH3'	5	3.370	2.994
NH6'	p7'	2.528	2.938
NH6'	6out'	2.582	2.995
NH6'	6in'	2.316	2.390
NH6'	Ac''	2.672	2.131
NH8	s2d	2.458	2.064
NH8	8out	2.645	2.880
NH8	8in	2.575	2.745
NH8	Ac'	3.656	3.290
NH8'	p9'	3.115	2.700
NH8'	s2d	2.862	2.694
NH8'	8out'	2.961	2.317
NH8'	8in'	3.033	2.926
NH8'	Ac'	3.591	4.364
p10	1out	1.973	2.219
p10	4	3.101	3.155
p10'	1out'	1.937	2.111
p2	4	4.022	5.025
p2	6'	3.296	3.304
p2'	1in'	2.090	2.324
p2'	3in'	2.116	2.416
p2'	5	3.238	3.472
p4	p5	2.390	2.375
p4	3out	2.149	2.054
p4'	3out'	2.274	2.112
p5	6out	2.063	2.076
p5'	p4'	2.309	2.367
p5'	6out'	1.915	2.092
p7	NH8	2.626	3.036
p7	8in	1.988	2.441
p7	6in	2.166	2.418
p7	2	3.388	4.031
p7	Ac''	4.192	4.694
p7'	8in'	2.023	2.359
p7'	Ac	2.174	2.565
p9	NH8	3.156	3.684
p9	8out	2.003	2.100
p9	4	3.418	3.494
p9'	p10'	3.156	2.401
p9'	8out'	1.860	2.148
p9'	3	3.669	3.313
s2a	1out	3.417	4.515
s2a	5	3.107	3.932
s2a	4	3.241	3.333
s2a	6	2.544	2.399
s2a	6'	2.228	2.860
s2b	5	3.164	4.257
s2b	6	3.439	4.071
s2c	p7	3.603	4.719
s2c	NH6'	2.389	3.202
s2c	2	3.172	3.969
s2c	Ac''	2.768	2.797
s2d	8in	3.606	4.589
s2d	3	3.738	3.768
s2d	2	3.432	4.094
s2d	Ac'	2.400	2.761
s4b	NH5b	2.150	1.950
s4c	NH5c	2.263	2.314
s6c	Ac''	3.574	4.516
s6d	NH5d	2.309	2.117
s6d	Ac'	3.574	4.423

Images of molecular model of 5 + glycopeptide 3

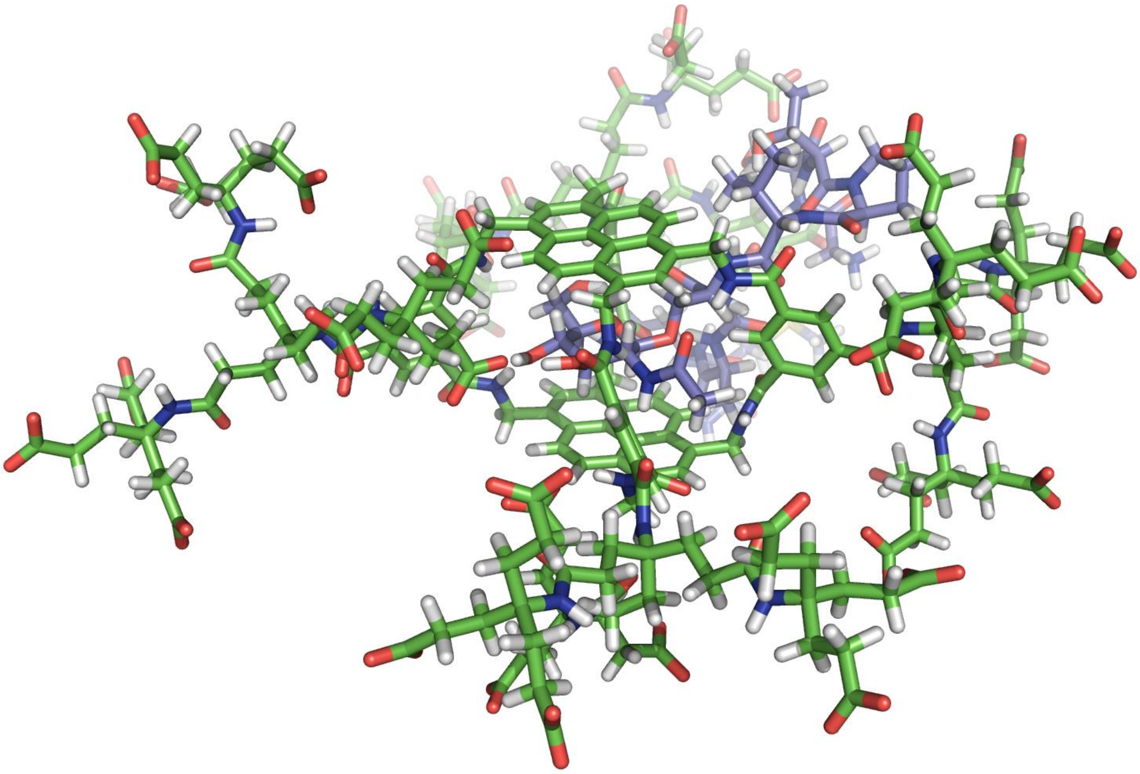

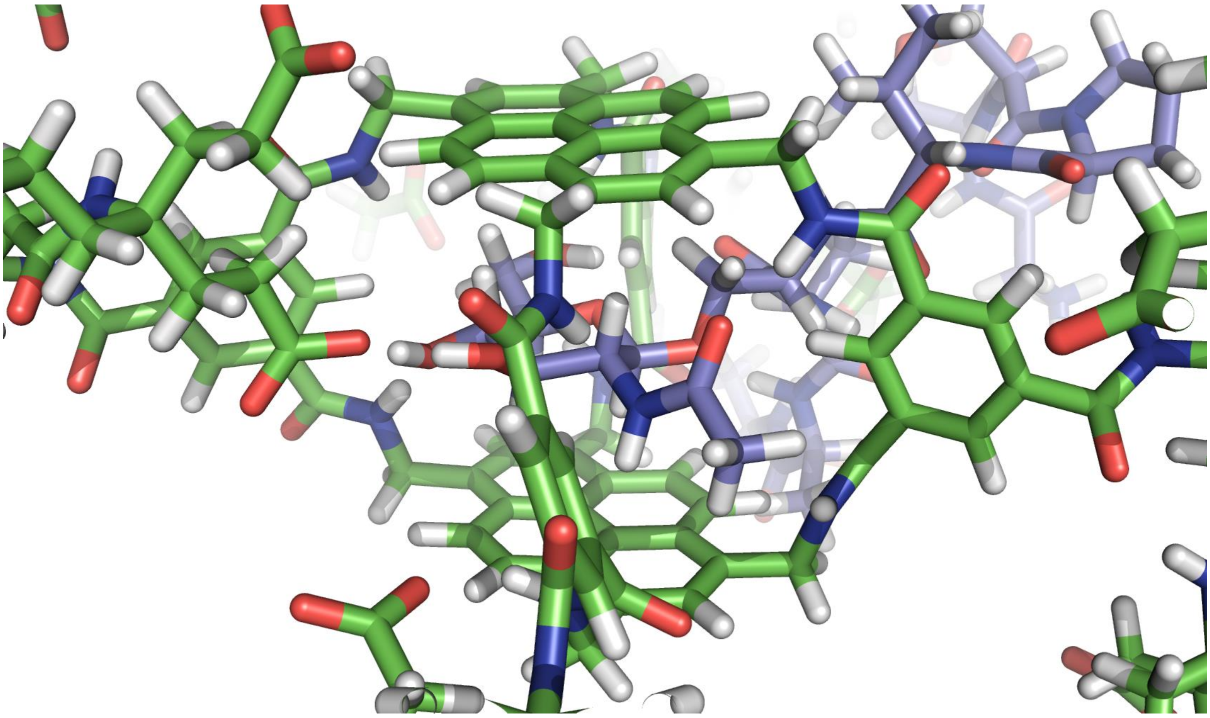

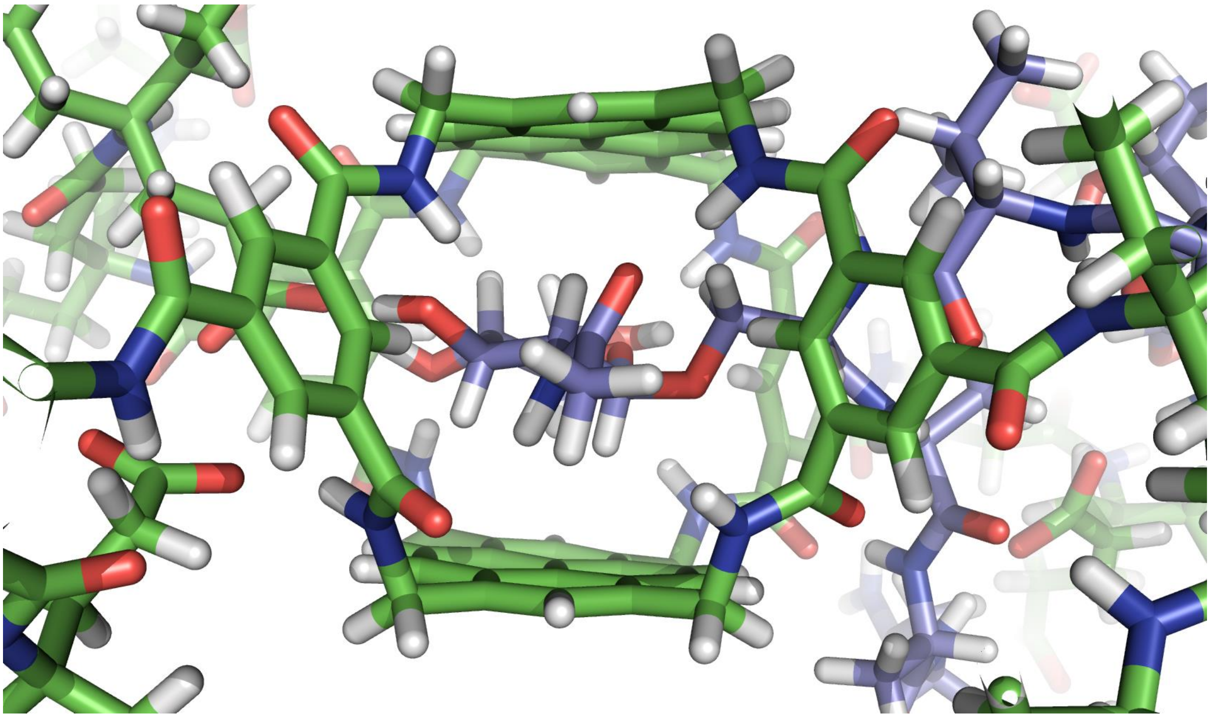

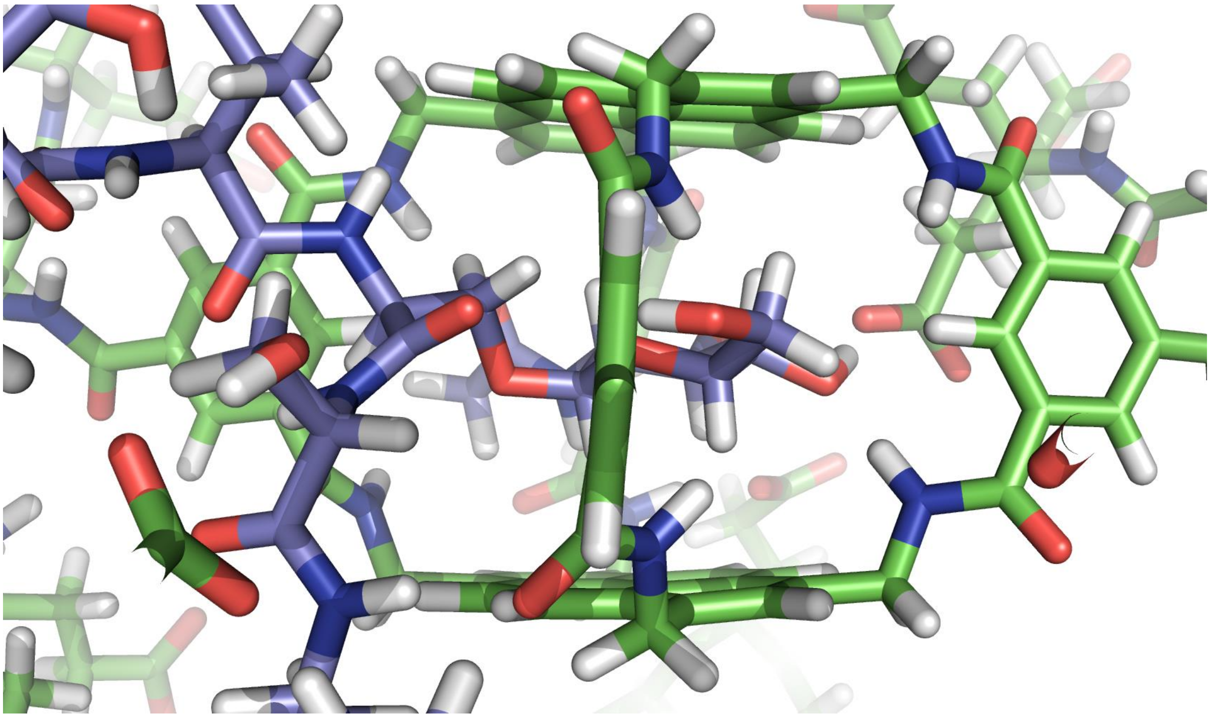

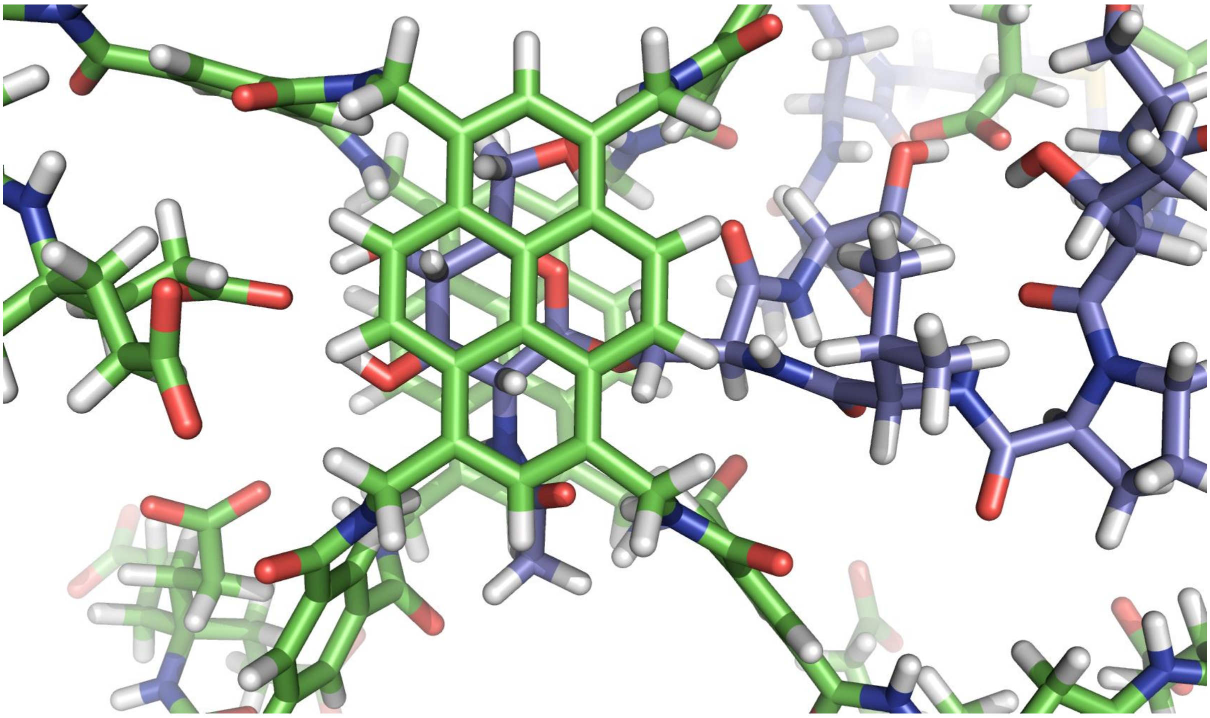

Staggered receptor 9 with methyl *N*-acetyl-β-D-glucosaminide

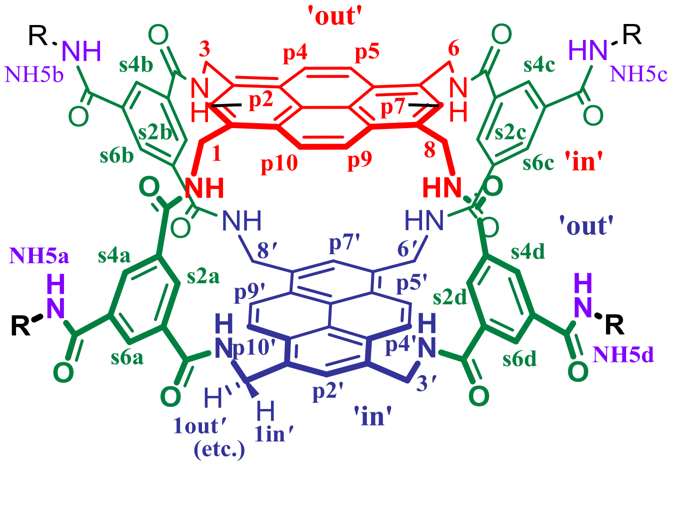

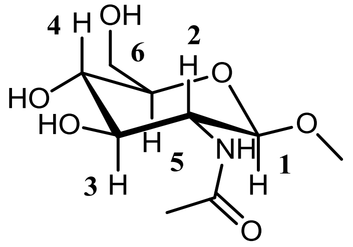

Figure S86. Structures of staggered receptor **9** and methyl *N*-acetyl-β-D-glucosaminide (**2**) with numbering using for structural assignment.

Assignment of ^{1}H NMR peaks

sx = 'spacer'
px = 'pyrene, top'
px' = 'pyrene, bottom'
Other = sugar

Type	Label	δ (ppm)	Type	Label	δ (ppm)	Type	Label	δ (ppm)
CH2	1in	4.54	CH	p10'	7.991	CH	s2a	7.123
CH2	1out	5.701	CH	p9'	8.086	CH	s4a	8.013
CH2	3in	4.683	CH	p7'	8.116	CH	s6a	8.231
CH2	3out	5.296	CH	p5'	8.385	CH	s2b	7.457
CH2	6in	4.673	CH	p4'	8.277	CH	s4b	8.106
CH2	6out	5.315	CH	p2'	7.902	CH	s6b	8.206
CH2	8in	4.436	NH	NH1	8.915	CH	s2c	7.724
CH2	8out	5.895	NH	NH3	9.112	CH	s4c	8.333
CH	p10	8.766	NH	NH6	8.238	CH	s6c	8.201
CH	p9	8.448	NH	NH8	8.606	CH	s2d	7.915
CH	p7	8.04	NH	NH1'	7.329	CH	s4d	8.267
CH	p5	8.171	NH	NH8'	8.806	CH	s6d	8.399
CH	p4	8.414	NH	NH6'	9.274	CH	1	-0.185
CH	p2	8.165	NH	NH3'	7.961	CH	2	0.015
CH2	1in'	4.605	NH	NH5a	-	CH	3	-0.939
CH2	1out'	5.237	NH	NH5b	-	CH	4	-0.455
CH2	3in'	5.162	NH	NH5c	-	CH	5	-0.028
CH2	3out'	5.043	NH	NH5d	-	CH2	6	2.065
CH2	6in'	4.705	NH	d2NHa	-	CH2	6'	-0.509
CH2	6out'	5.516	NH	d2NHb	-	CH3	Ac	1.586
CH2	8in'	4.572	NH	d2NHc	-	NH	NH	1.044
CH2	8out'	5.574	NH	d2NHd	-	CH3	OMe	3.594

2D ^{1}H NMR Spectra with assignments (600 MHz)

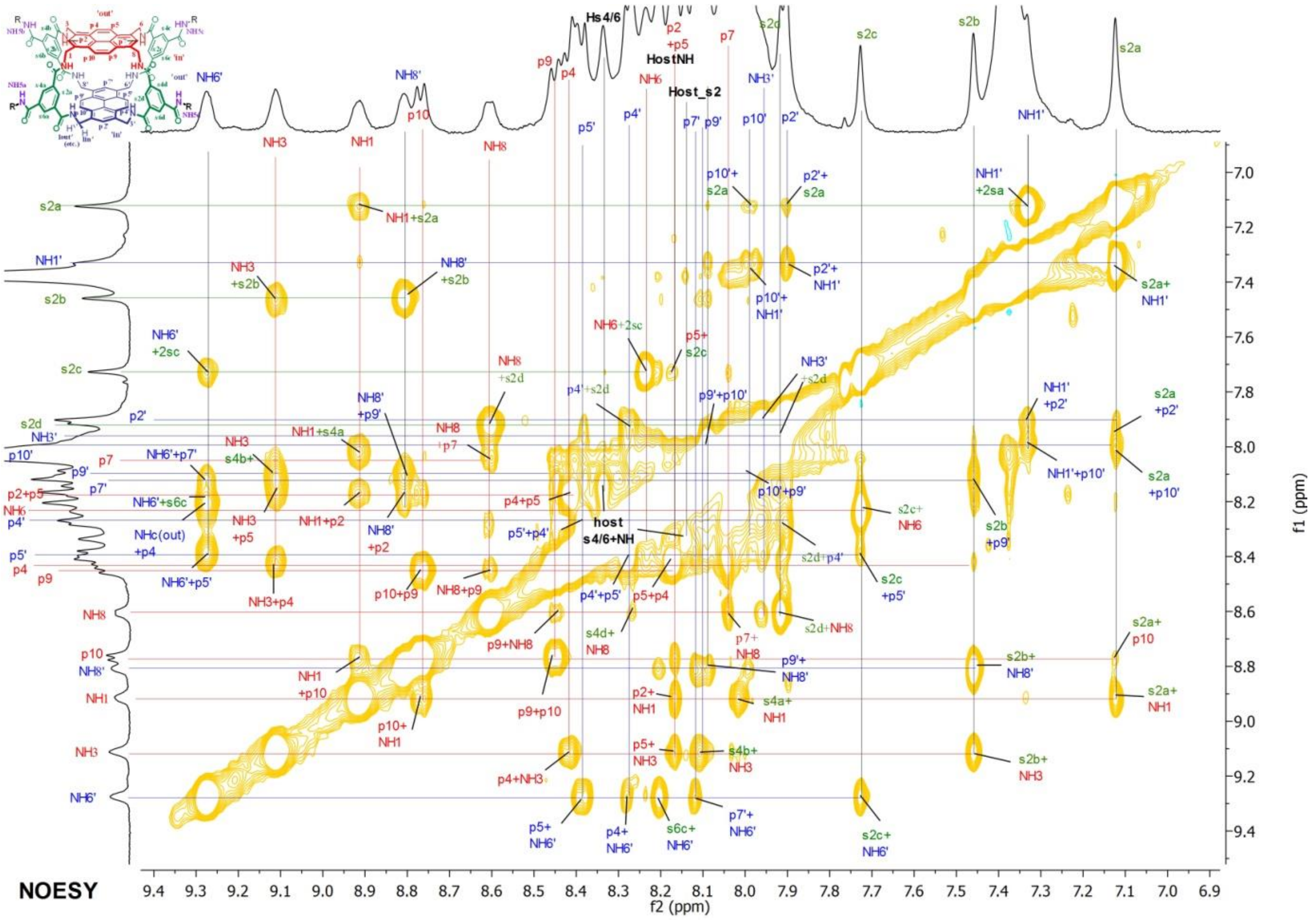

Figure S87. Partial ^{1}H NOESY NMR spectrum of the staggered receptor **9** (0.40 mM) complexed with methyl *N*-acetyl-β-D-glucosaminide (**2**) (4.50 mM) in 1:9 D_2O/H_2O at 298 K.

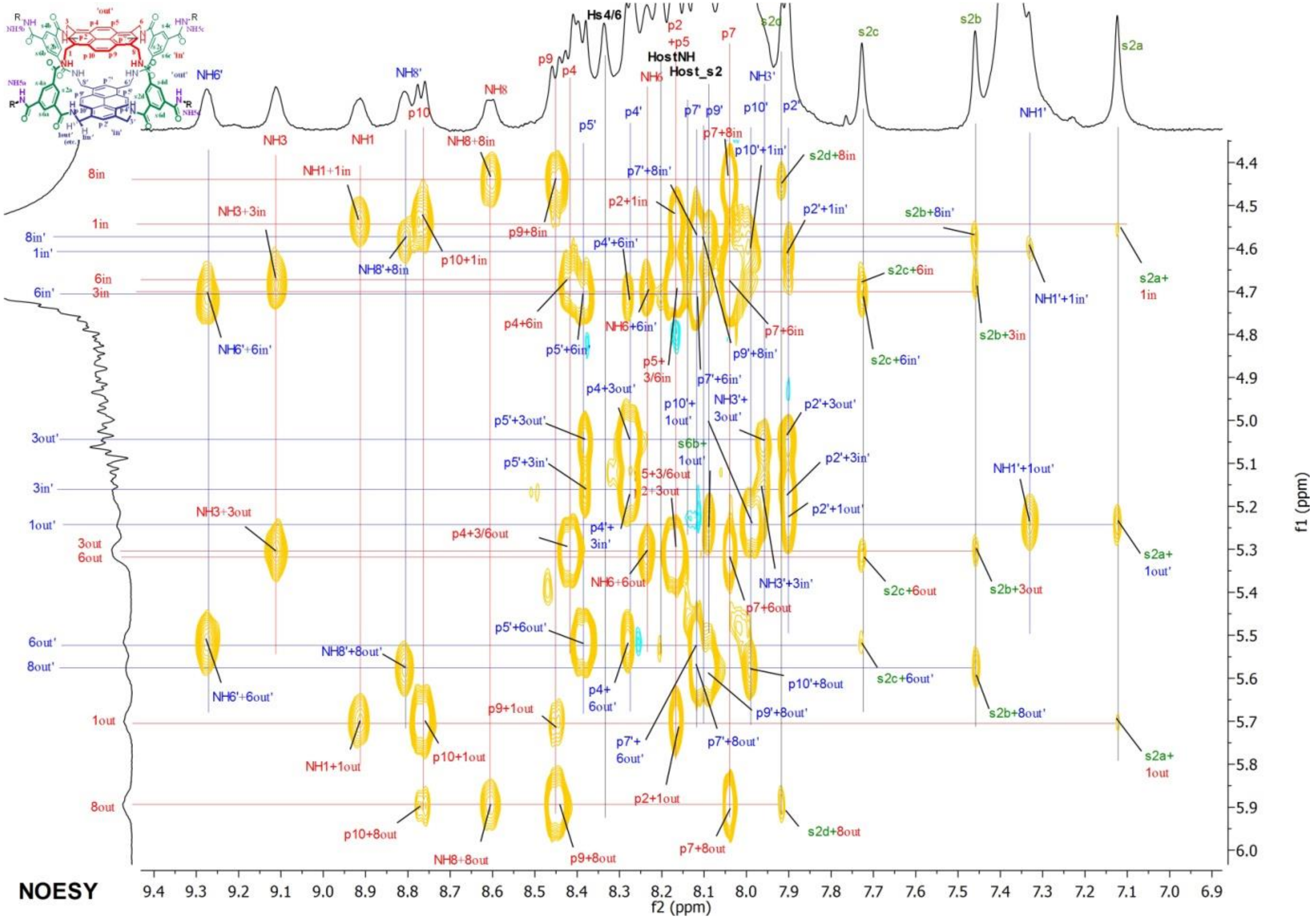

Figure S88. Partial ^{1}H NOESY NMR spectrum of the staggered receptor **9** (0.40 mM) complexed with methyl *N*-acetyl-β-D-glucosaminide (**2**) (4.50 mM) in 1:9 D_2O/H_2O at 298 K.

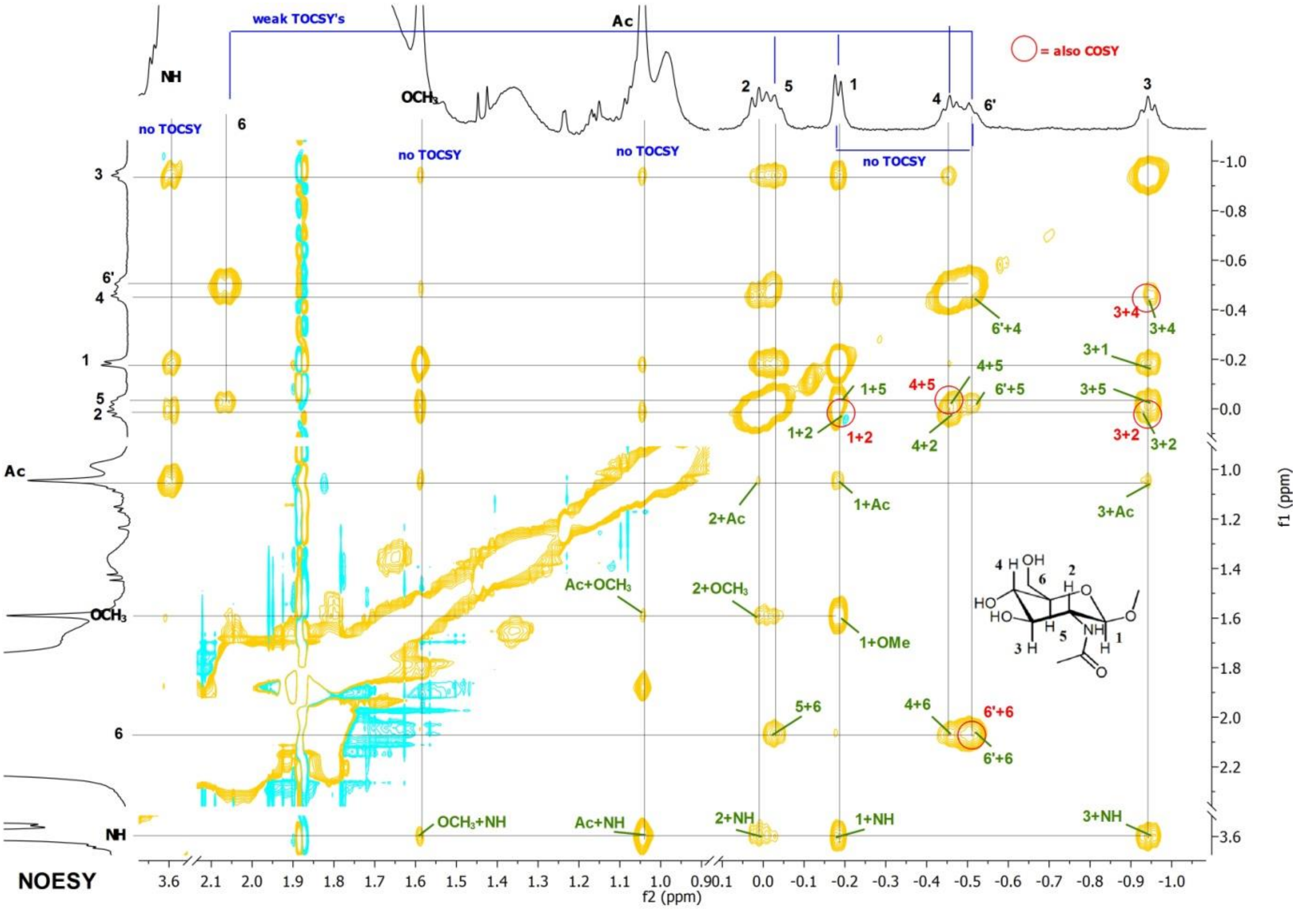

Figure S89. Partial ^{1}H NOESY NMR spectrum of the staggered receptor **9** (0.40 mM) complexed with methyl *N*-acetyl-β-D-glucosaminide (**2**) (4.50 mM) in 1:9 D_2O/H_2O at 298 K.

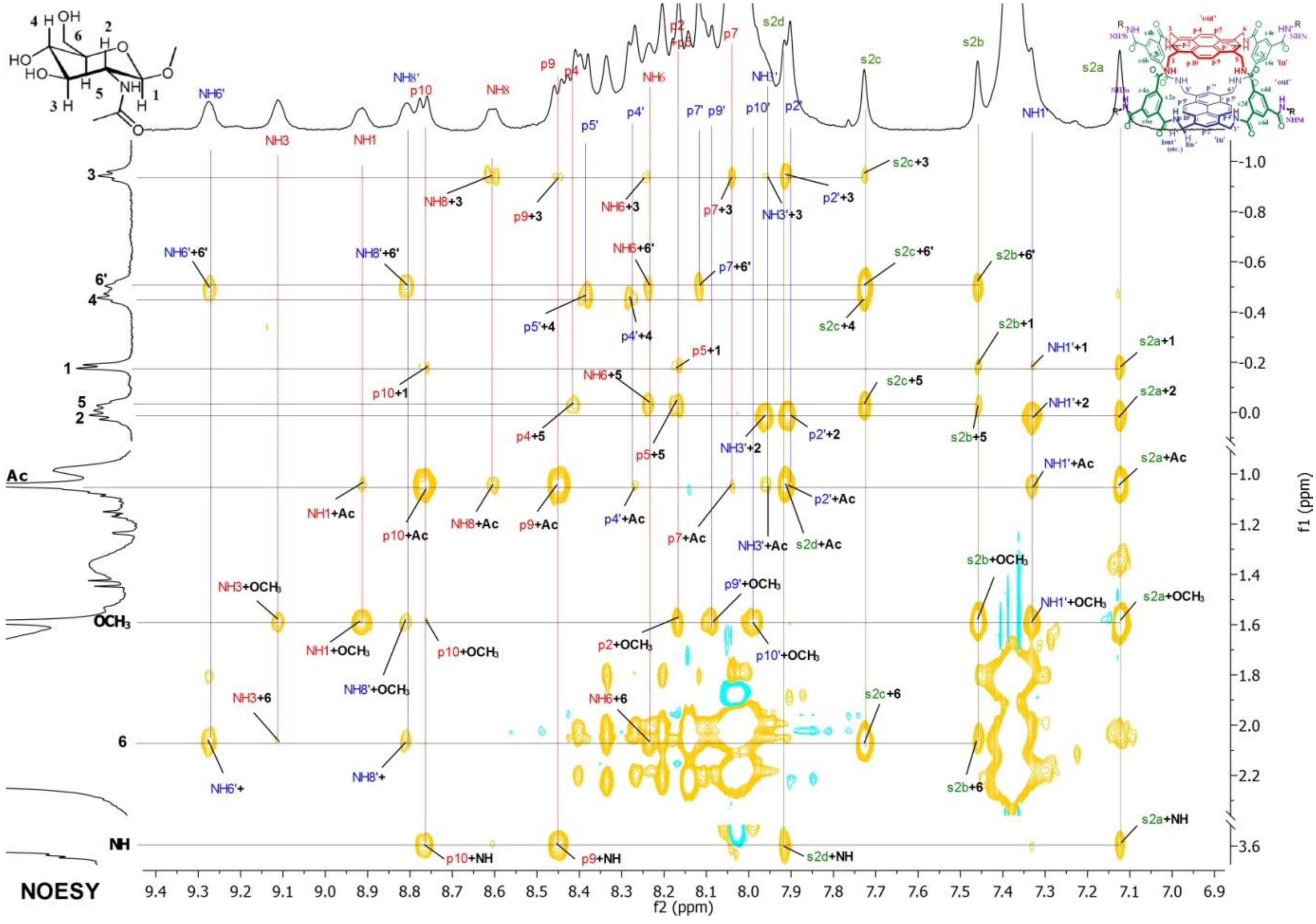

Figure S90. Partial ^{1}H NOESY NMR spectrum of the staggered receptor **9** (0.40 mM) complexed with methyl *N*-acetyl-β-D-glucosaminide (**2**) (4.50 mM) in 1:9 D_2O/H_2O at 298 K.

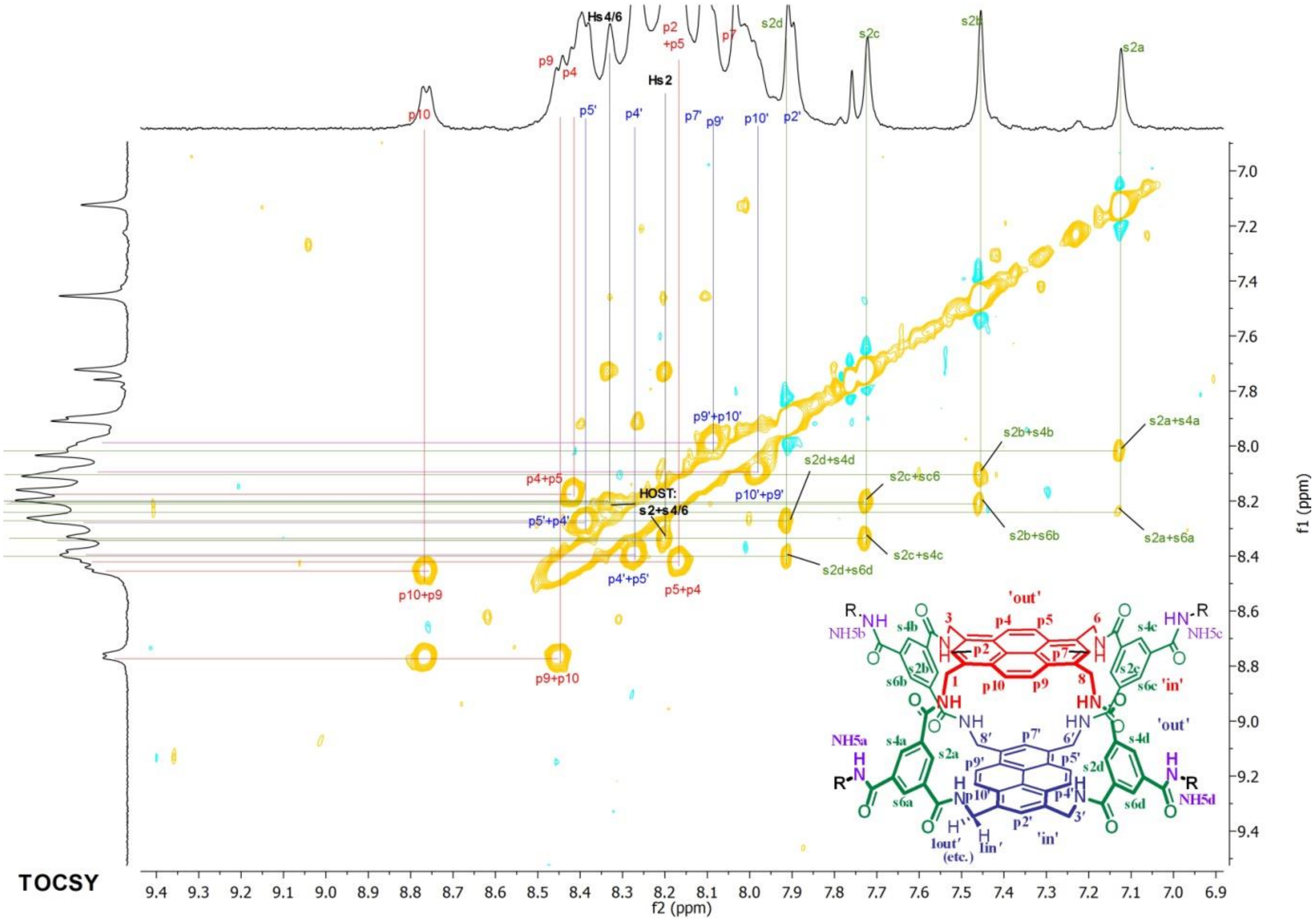

Figure S91. Partial ^{1}H TOCSY NMR spectrum of the staggered receptor **9** (0.40 mM) complexed with methyl *N*-acetyl-β-D-glucosaminide (**2**) (4.50 mM) in 1:9 D_2O/H_2O at 298 K.

Distances obtained from NOE data and subsequent molecular modelling

105 Distances RMSD = 0.606 Å

Receptor 9 + GlcNAc-β-OMe (2)

Labels f2	Labels f1	Distance (A) nOe	Distance (A) model	Labels f2	Labels f1	Distance (A) nOe	Distance (A) model
				8in	NH8	2.703	2.645
				8in	p7	2.206	2.310
1	NH1'	4.686	4.598	8in	s2d	3.567	4.595
1	p10	4.370	4.458	8in'	s2b	3.837	4.548
1	p5	4.209	4.545	8out	NH8	2.832	2.956
1	s2a	3.545	3.979	8out	p9	2.195	1.959
1	s2b	4.236	5.231	8out	s2d	3.757	4.520
2	NH1'	2.882	3.253	8out'	NH3'	2.840	2.924
2	NH3'	3.021	2.819	8out'	s2b	3.642	4.693
2	p2'	2.947	3.492	Ac''	NH1	3.944	4.233
2	s2a	3.202	4.106	Ac''	NH1'	3.505	3.857
3	NH3'	4.363	4.080	Ac	NH8	3.793	3.311
3	NH6	4.065	3.406	Ac	NH8'	3.896	4.688
3	NH8	3.614	2.853	Ac''	p10	2.671	2.865
3	p7	3.972	3.486	Ac	p9	2.562	2.354
3	p9	4.100	4.517	Ac''	s2a	2.706	2.883
3	s2c	4.200	4.981	NH	p10	3.202	3.719
3	s2d	3.472	3.795	NH	p9	2.925	2.954
4	p4'	3.616	3.660	NH	s2a	3.443	4.217
4	p5'	3.352	3.444	NH	s2d	3.231	3.170
5	NH6	3.626	3.158	NH1'	p2'	2.924	2.702
5	p4	3.735	3.682	OMe'	NH1	3.081	3.030
5	p5	3.432	2.685	OMe'	NH1'	2.902	3.517
5	s2b	4.004	4.718	OMe	NH3	3.659	3.109
5	s2c	3.234	3.783	OMe''	NH3'	3.787	3.915
6	NH3	3.701	4.545	OMe	p10	4.880	4.554
6	NH6'	3.281	4.082	OMe'	p10'	2.999	3.699
6	s2c	2.461	2.040	OMe	p2	3.286	3.741
1in	NH1	2.830	2.376	OMe''	p9'	3.131	3.927
1in	p2	2.363	2.318	OMe'	s2a	2.368	2.446
1in	s2a	4.441	5.278	OMe''	s2b	2.515	3.426
1in'	NH1'	4.149	2.944	p10	NH1	2.928	3.857
1out	NH1	2.775	2.986	p2	NH1	2.817	3.282
1out	p10	2.223	2.037	p4	NH3	2.708	2.452
1out	s2a	4.670	5.071	p4'	NH6'	3.042	4.855
1out'	NH1'	2.867	2.777	p5	NH3	2.833	4.144
1out'	s2a	3.639	3.985	p5'	NH6'	2.542	3.012
3in	NH3	2.778	2.952	p7	NH8	2.899	3.105
3in	s2b	3.983	4.980	p7'	NH6'	2.889	3.947
3out	NH3	2.620	2.641	p9	NH8	3.377	3.754
3out	s2b	3.941	4.795	p9	p10	2.347	2.328
3out'	NH8'	2.997	2.639	p9'	NH3'	2.596	3.109
6'	NH3'	3.480	2.735	s2a	NH1	2.782	3.113
6'	NH6	3.875	4.283	s2a	NH1'	2.110	2.034
6'	NH6'	3.500	3.761	s2a	p10'	3.586	4.767
6'	p7'	3.557	3.126	s2a	p2'	3.718	4.671
6'	s2b	3.417	4.258	s2b	NH3	2.649	2.657
6in'	NH6'	2.602	2.939	s2b	NH3'	2.292	2.195
6out	NH6	2.740	2.964	s2c	NH6'	2.754	3.080
6out	p7	2.447	3.393	s2d	NH8	2.314	2.123
6out	s2c	3.776	4.615	s4a	NH1	2.631	3.529
6out'	NH6'	2.513	2.329	s4b	NH3	2.656	3.824
6out'	p5'	2.190	2.262	s6c	NH6'	2.541	3.388
6out'	s2c	4.347	5.206				

Images of molecular model of 9 + GlcNAc-*β*-OMe (2)

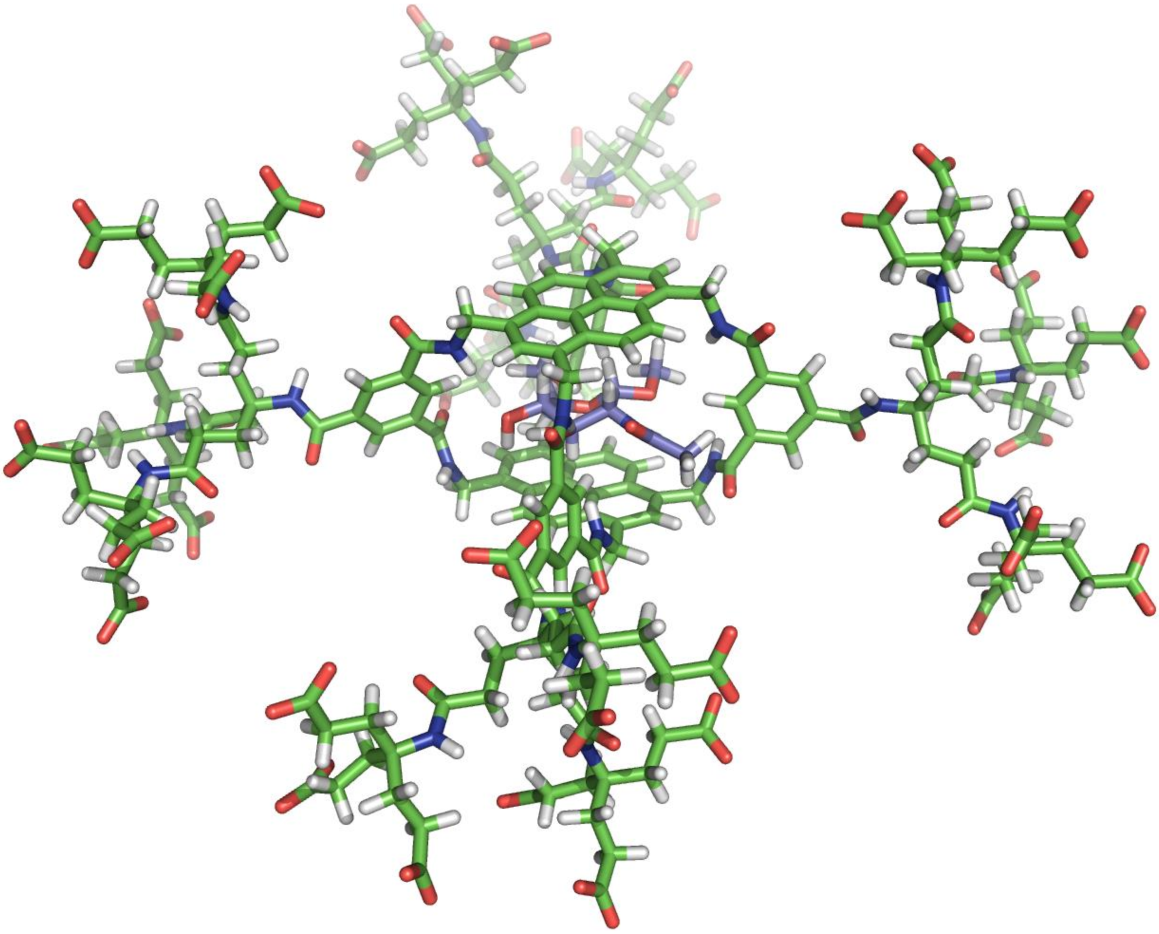

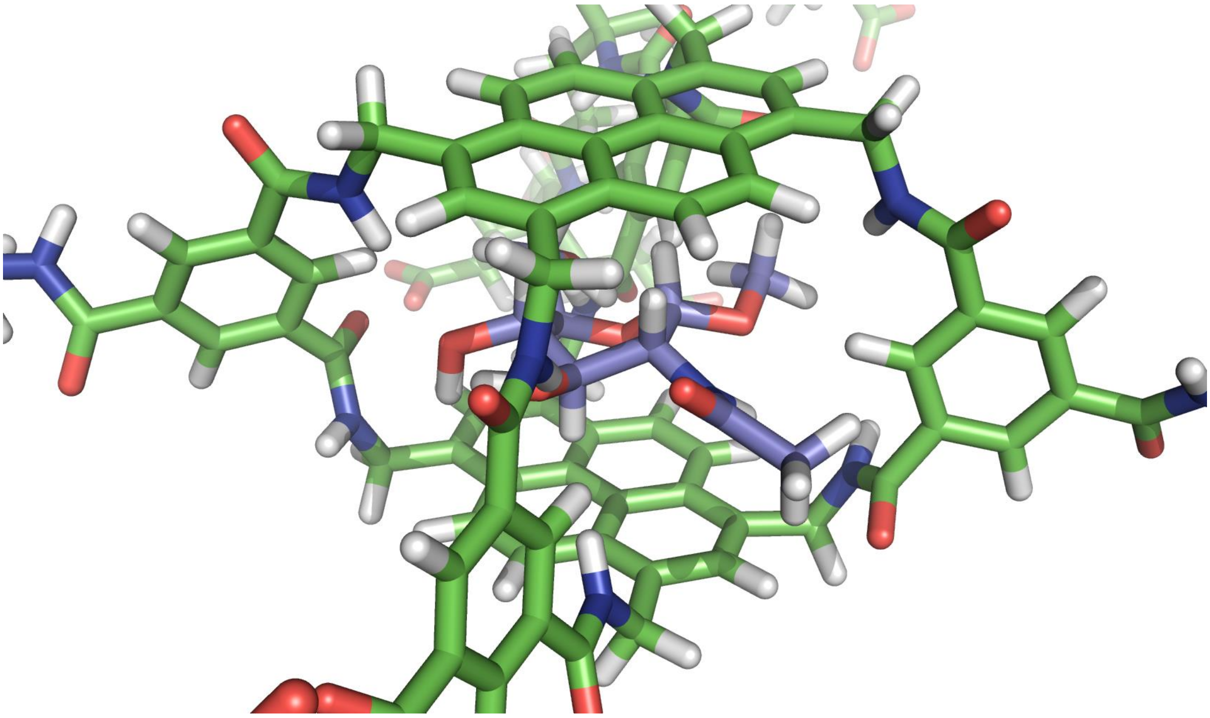

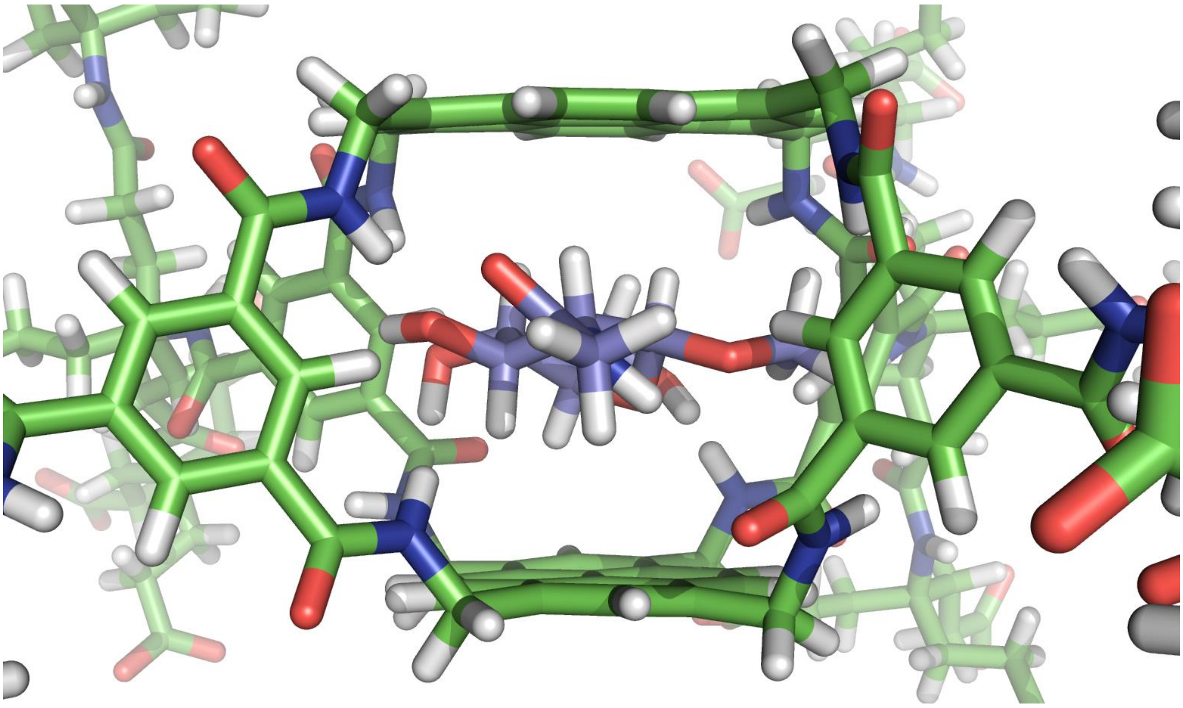

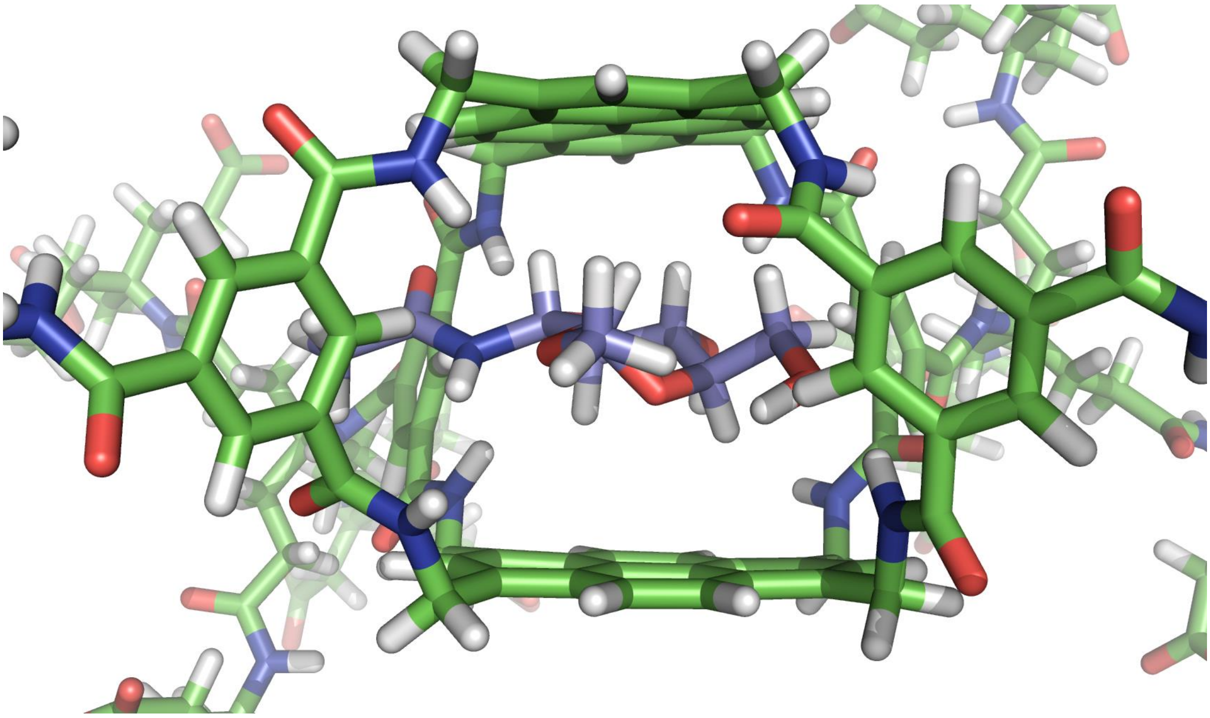

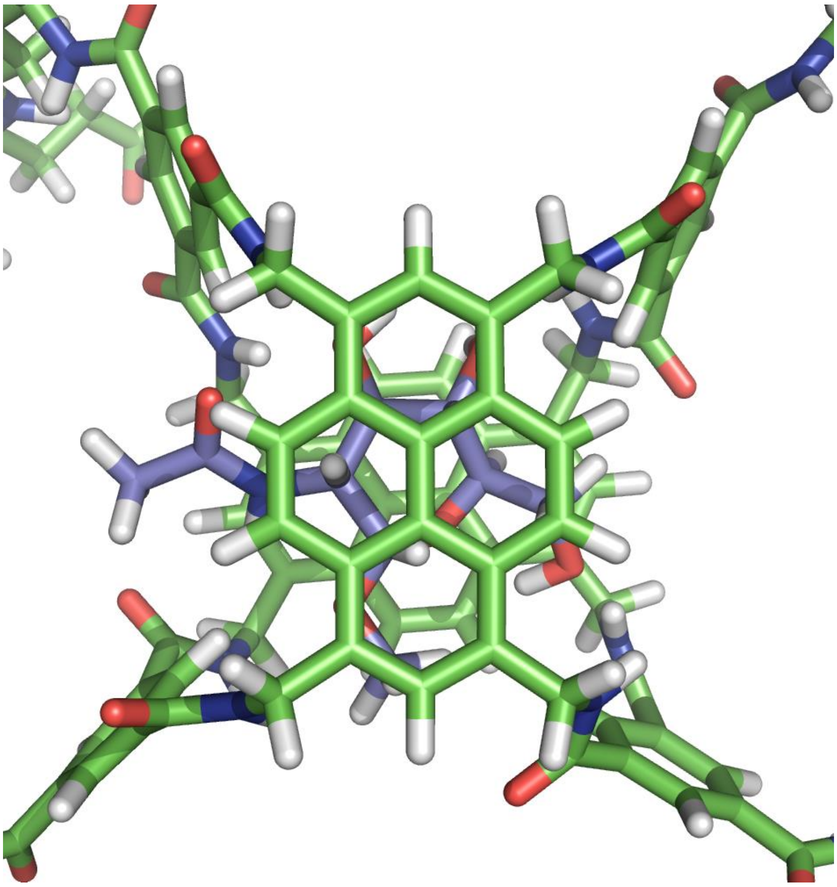

Staggered receptor 9 with methyl *N*-acetyl-α-D-glucosaminide (13)

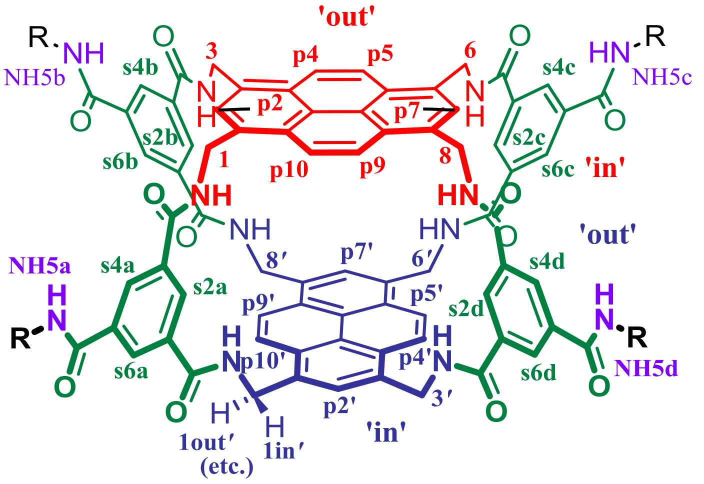

'staggered' **9**

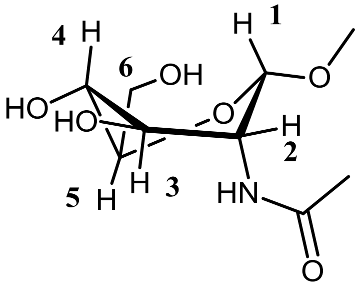

Me-α-GlcNAc **13**

Figure S92. Structures of staggered receptor **9** and methyl *N*-acetyl-α-D-glucosaminide with numbering using for structural assignment.

Assignment of ^{1}H NMR peaks

sx = 'spacer'
px = 'pyrene, top'
px' = 'pyrene, bottom'
Other = sugar

Type	Label	δ (ppm)	Type	Label	δ (ppm)	Type	Label	δ (ppm)
CH2	1in	4.661	CH	p10'	8.018	CH	s2a	7.666
CH2	1out	5.506	CH	p9'	8.571	CH	s4a	8.028
CH2	3in	4.513	CH	p7'	7.898	CH	s6a	8.303
CH2	3out	5.881	CH	p5'	8.109	CH	s2b	7.092
CH2	6in	4.72	CH	p4'	8.462	CH	s4b	8.186
CH2	6out	5.316	CH	p2'	8.182	CH	s6b	8.105
CH2	8in	4.538	NH	NH1	9.26	CH	s2c	7.872
CH2	8out	5.304	NH	NH3	9.094	CH	s4c	8.421
CH	p10	8.554	NH	NH6	8.472	CH	s6c	8.279
CH	p9	8.035	NH	NH8	8.555	CH	s2d	7.857
CH	p7	7.912	NH	NH1'	8.679	CH	s4d	8.352
CH	p5	8.302	NH	NH8'	9.14	CH	s6d	8.147
CH	p4	8.295	NH	NH6'	8.402	CH	1	2.023
CH	p2	8.156	NH	NH3'	9.277	CH	2	-0.003
CH2	1in'	4.545	NH	NH5a	-	CH	3	-1.05
CH2	1out'	5.656	NH	NH5b	-	CH	4	-0.055
CH2	3in'	4.93	NH	NH5c	-	CH	5	0.19
CH2	3out'	5.337	NH	NH5d	-	CH2	6	2.113
CH2	6in'	4.591	NH	d2NHa	-	CH2	6'	-0.489
CH2	6out'	5.592	NH	d2NHb	-	CH3	Ac	0.808
CH2	8in'	4.401	NH	d2NHc	-	NH	NH	3.757
CH2	8out'	5.425	NH	d2NHd	-	CH3	OMe	0.924

2D ¹H NMR Spectra with assignments (600 MHz)

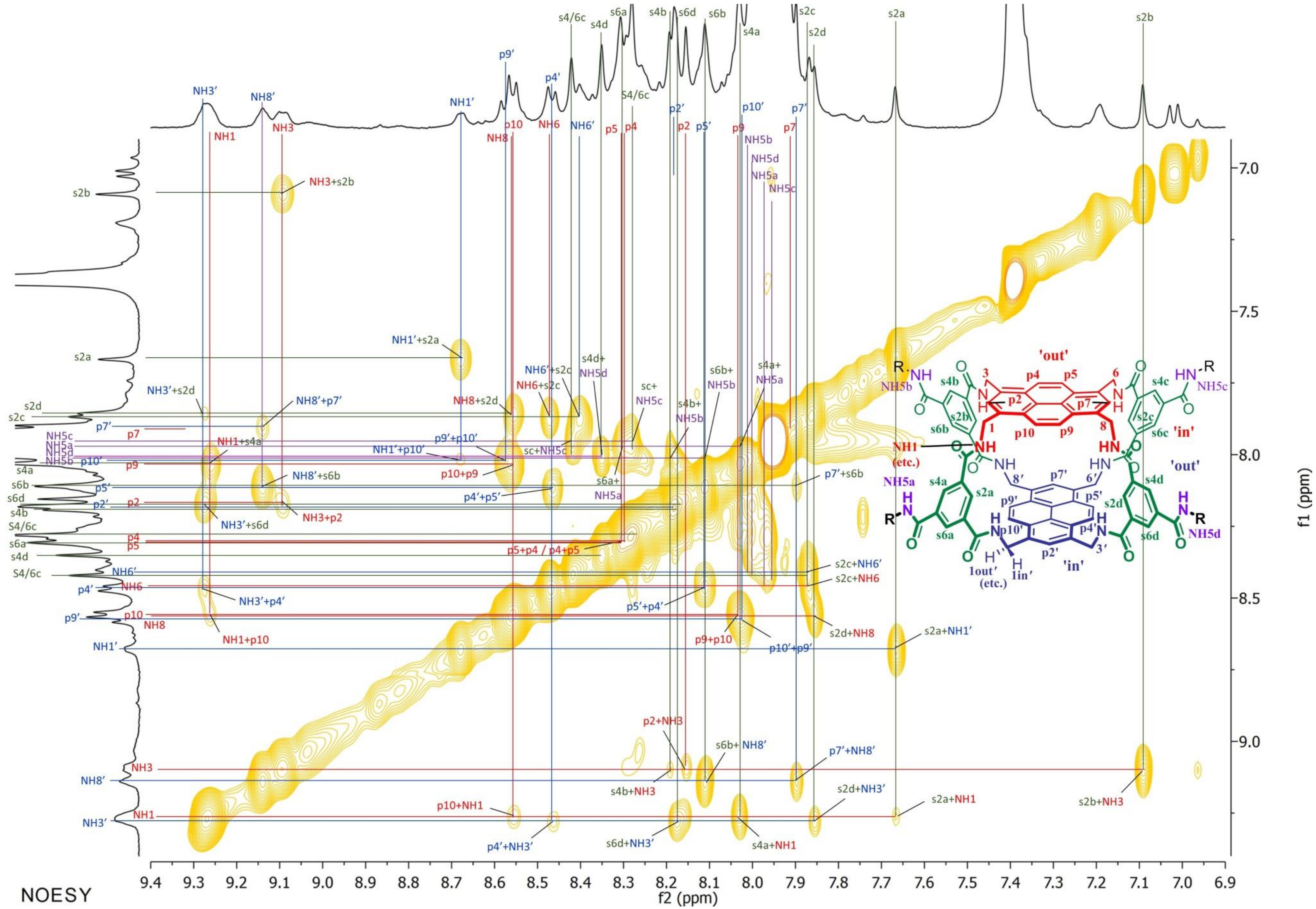

Figure S93. Partial ¹H NOESY NMR spectrum of the staggered receptor **9** (0.60 mM) complexed with methyl *N*-acetyl-α-D-glucosaminide (**13**) (30 mM) in 1:9 D_2O/H_2O at 298 K.

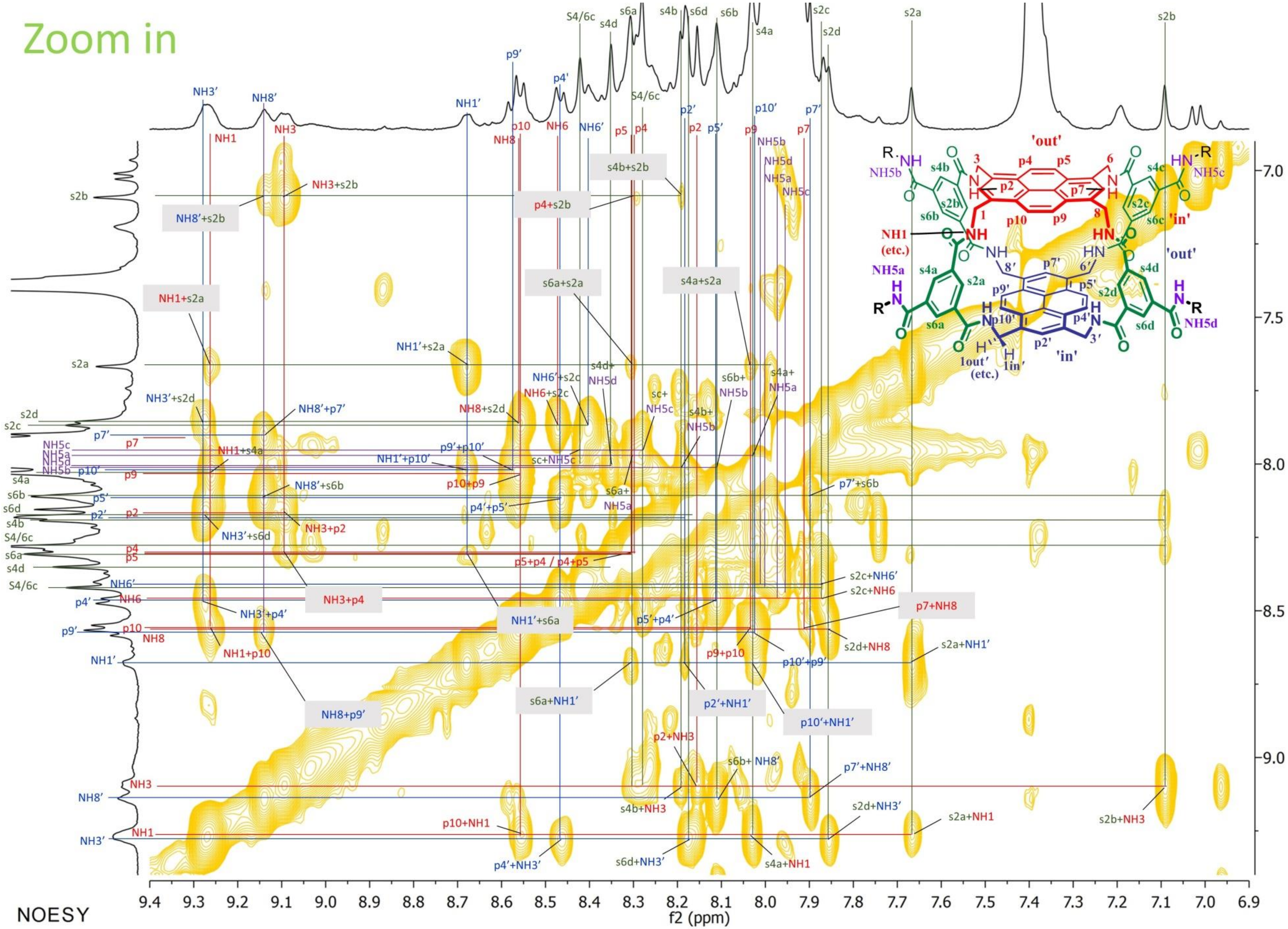

Figure S94. Partial ^{1}H NOESY NMR spectrum of the staggered receptor **9** (0.60 mM) complexed with methyl *N*-acetyl-α-D-glucosaminide (**13**) (30 mM) in 1:9 D_2O/H_2O at 298 K.

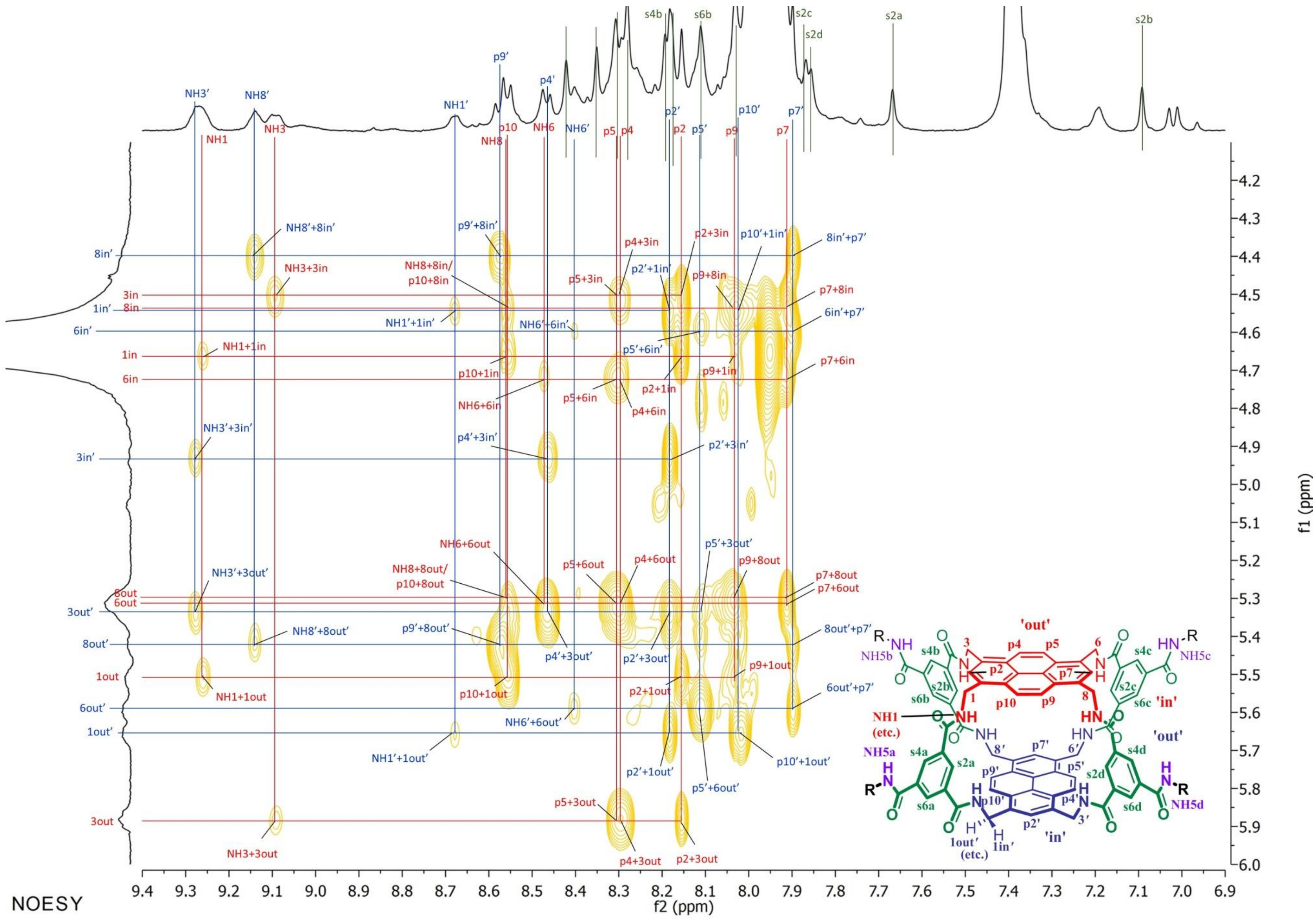

Figure S95. Partial ^{1}H NOESY NMR spectrum of the staggered receptor **9** (0.60 mM) complexed with methyl *N*-acetyl-α-D-glucosaminide (**13**) (30 mM) in 1:9 D_2O/H_2O at 298 K.

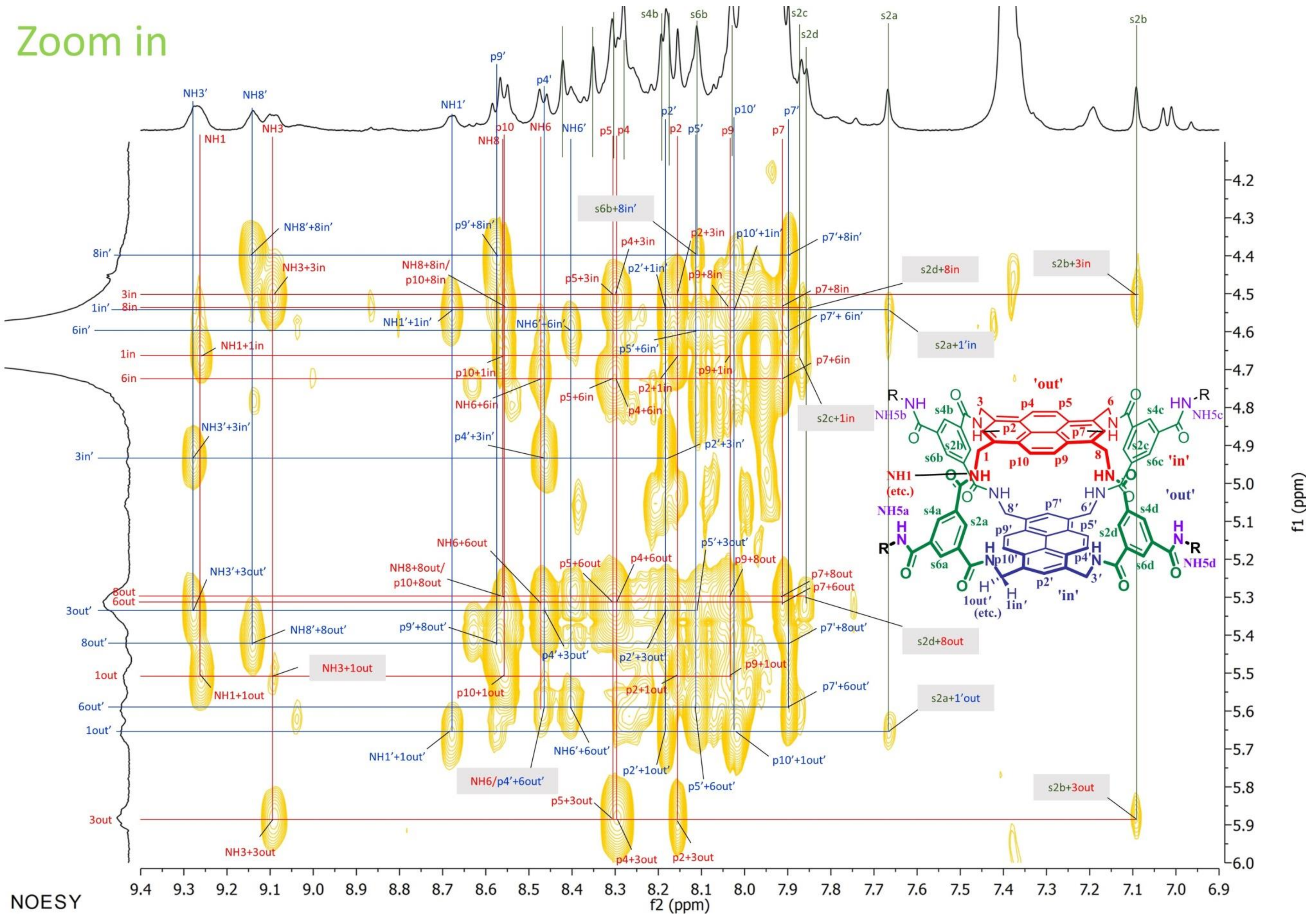

Figure S96. Partial ^{1}H NOESY NMR spectrum of the staggered receptor **9** (0.60 mM) complexed with methyl *N*-acetyl-α-D-glucosaminide (**13**) (30 mM) in 1:9 D_2O/H_2O at 298 K.

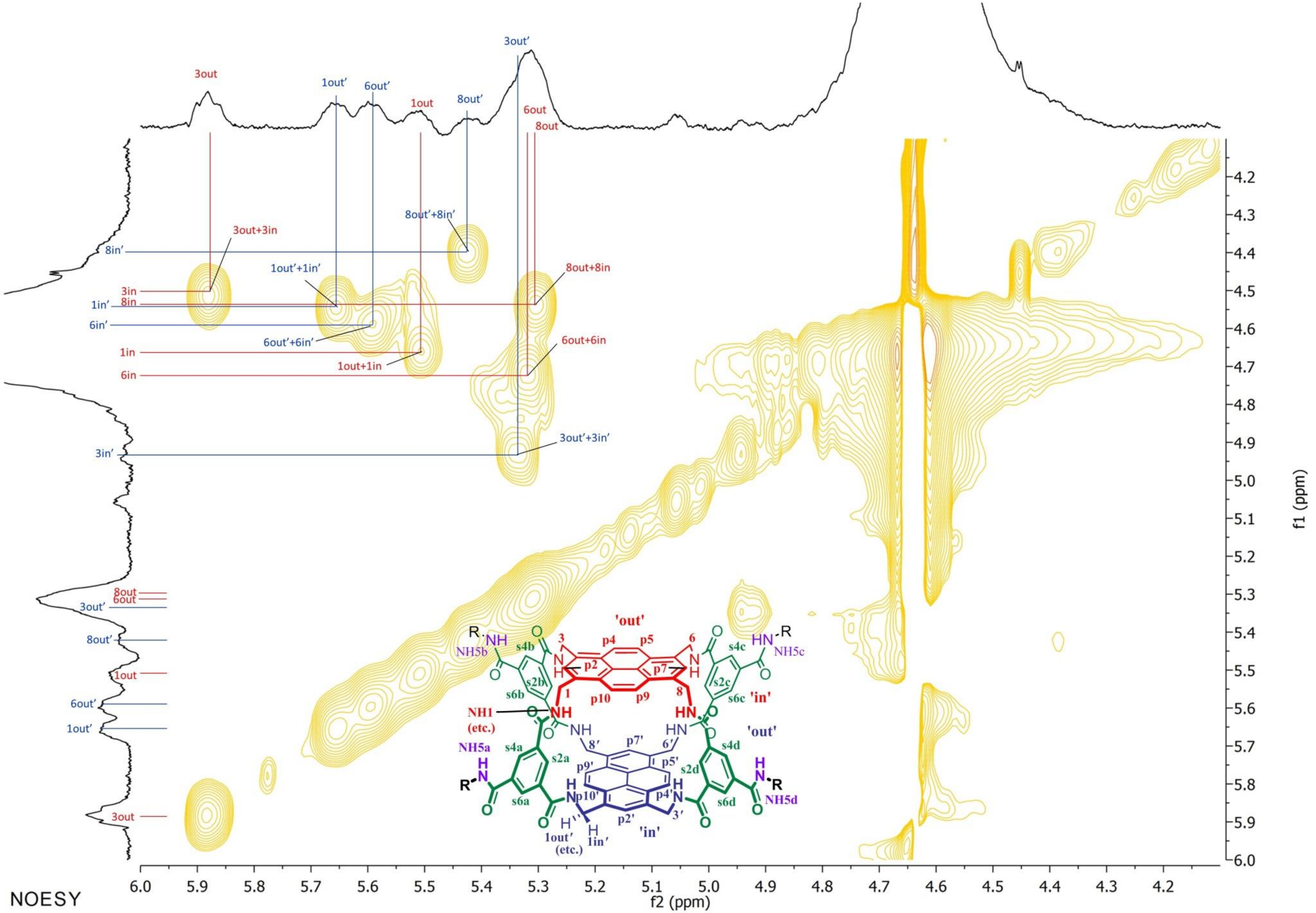

Figure S97. Partial ^{1}H NOESY NMR spectrum of the staggered receptor **9** (0.60 mM) complexed with methyl *N*-acetyl-α-D-glucosaminide (**13**) (30 mM) in 1:9 D_2O/H_2O at 298 K.

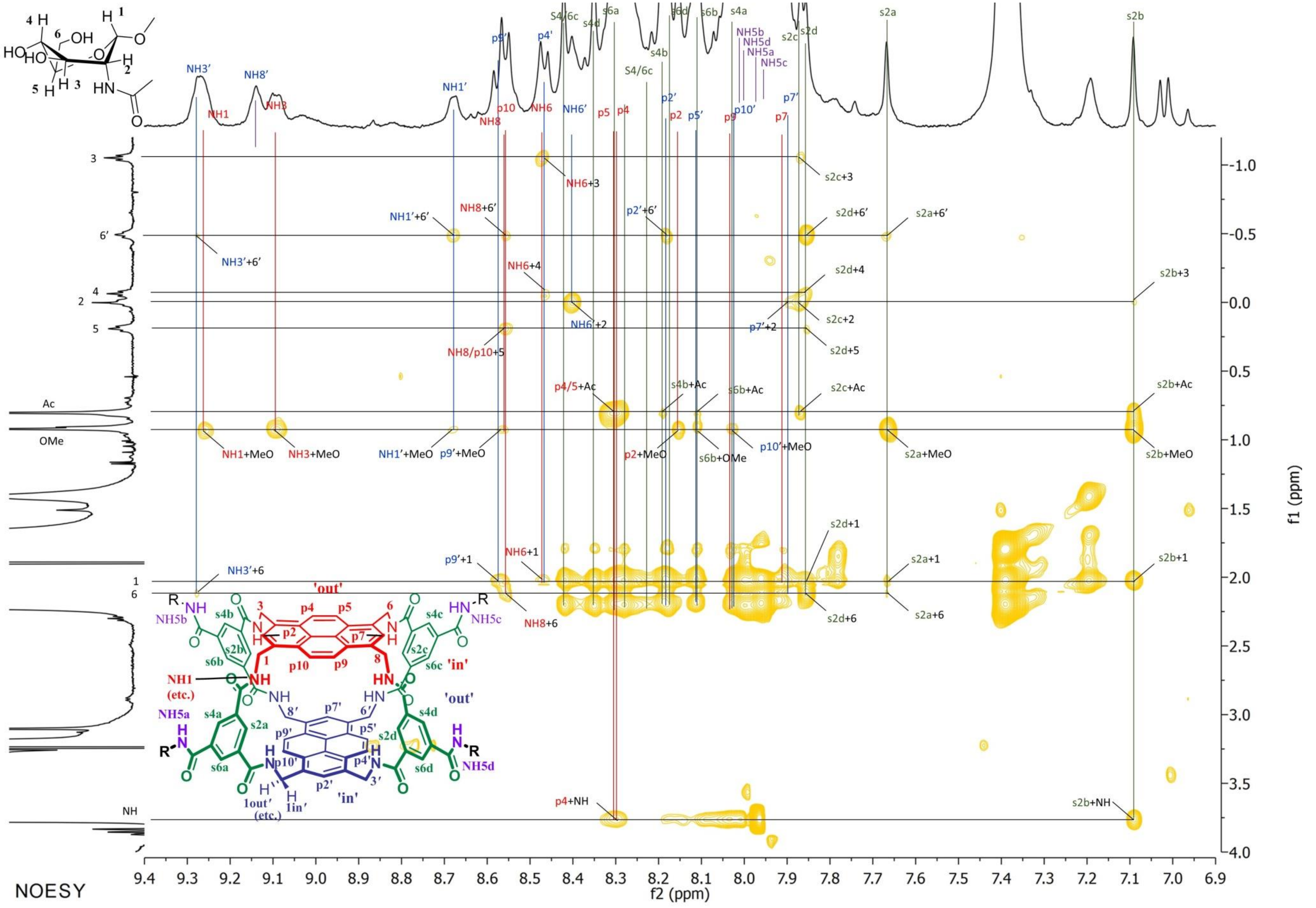

Figure S98. Partial ^{1}H NOESY NMR spectrum of the staggered receptor **9** (0.60 mM) complexed with methyl *N*-acetyl-α-D-glucosaminide (**13**) (30 mM) in 1:9 D_2O/H_2O at 298 K.

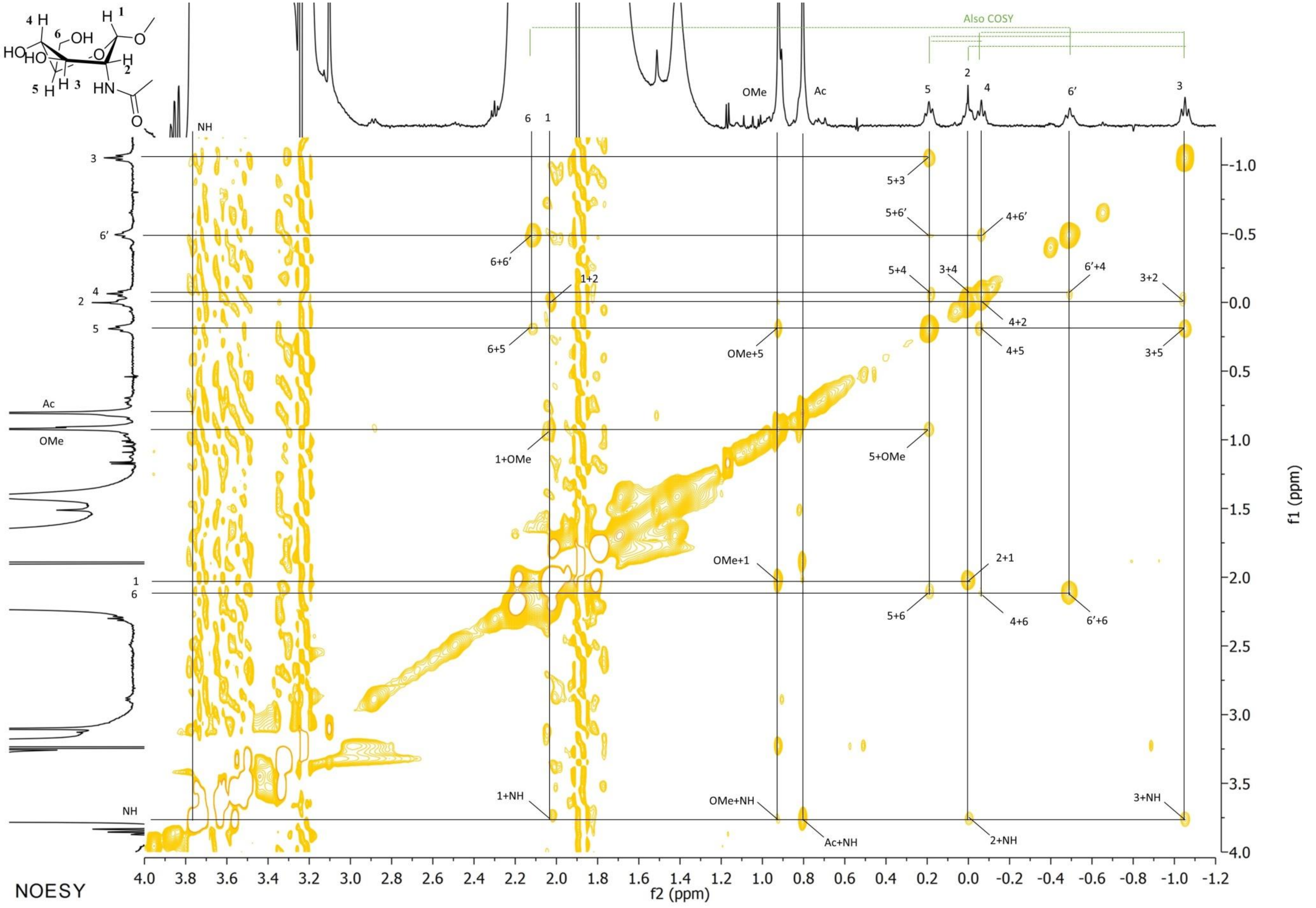

Figure S99. Partial ^{1}H NOESY NMR spectrum of the staggered receptor **9** (0.60 mM) complexed with methyl *N*-acetyl-α-D-glucosaminide (**13**) (30 mM) in 1:9 D_2O/H_2O at 298 K.

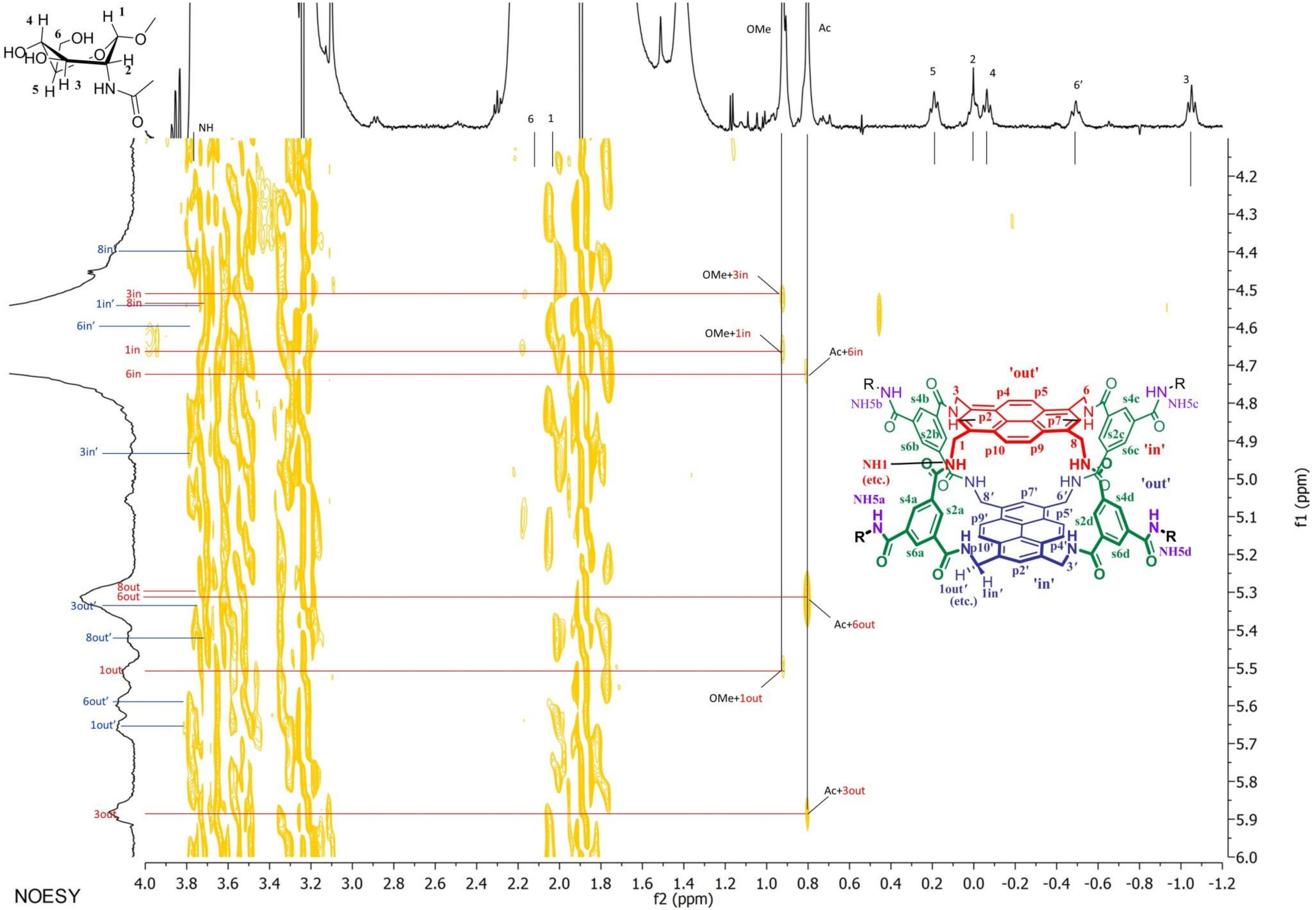

Figure S100. Partial ^{1}H NOESY NMR spectrum of the staggered receptor **9** (0.60 mM) complexed with methyl *N*-acetyl-α-D-glucosaminide (**13**) (30 mM) in 1:9 D_2O/H_2O at 298 K.

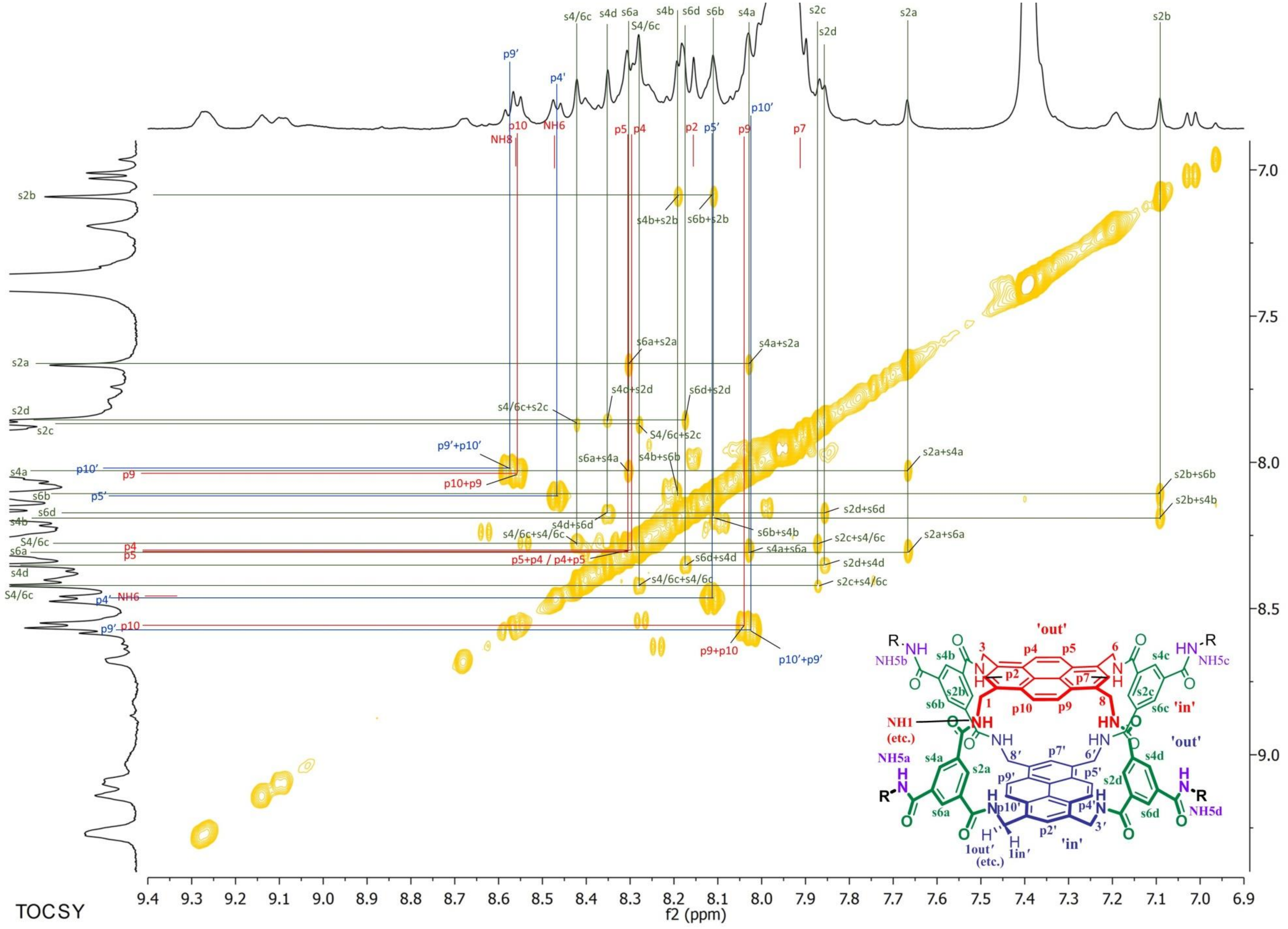

Figure S101. Partial ^{1}H TOCSY NMR spectrum of the staggered receptor **9** (0.60 mM) complexed with methyl *N*-acetyl-α-D-glucosaminide (**13**) (30 mM) in 1:9 D_2O/H_2O at 298 K.

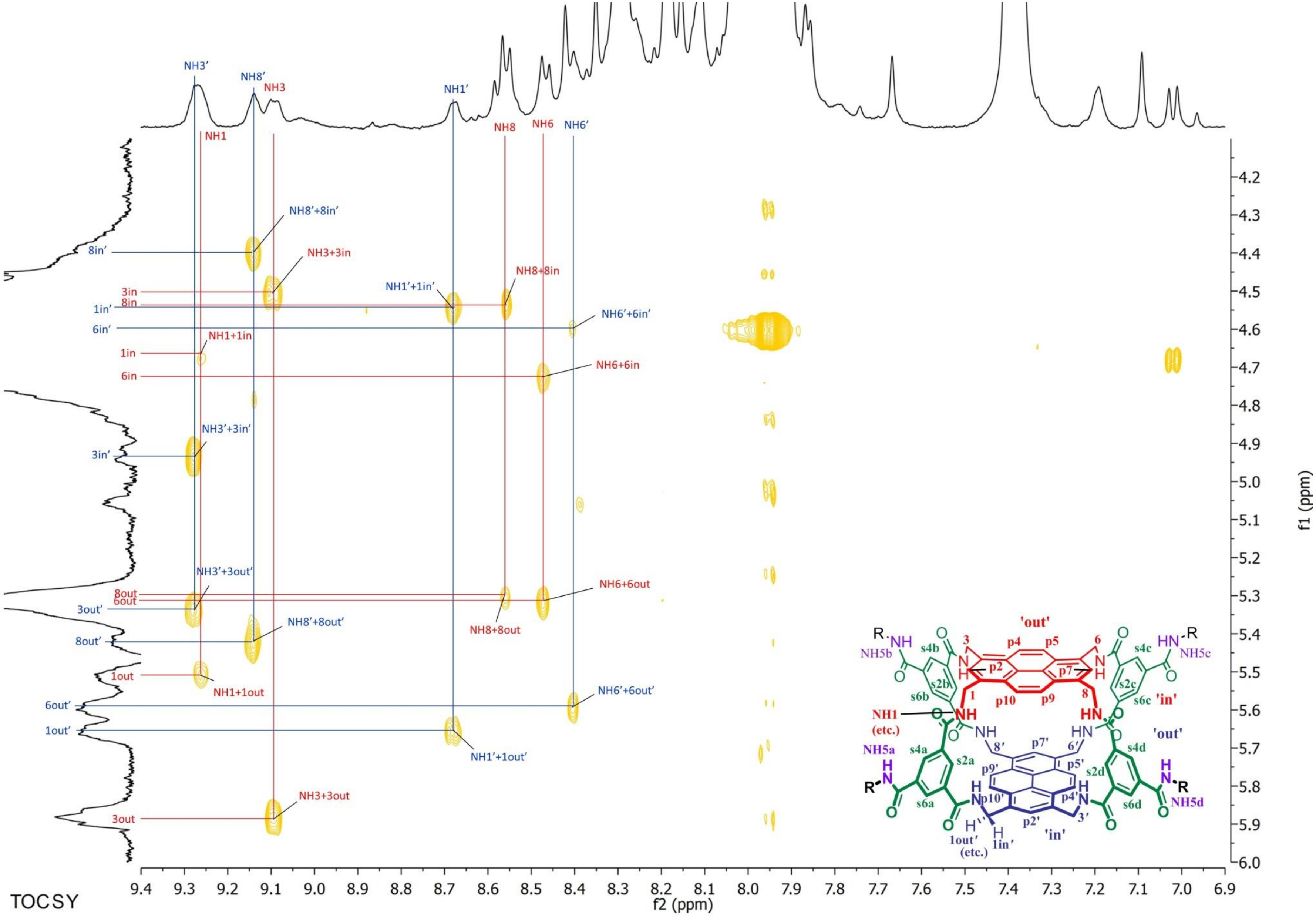

Figure S102. Partial ^{1}H TOCSY NMR spectrum of the staggered receptor **9** (0.60 mM) complexed with methyl *N*-acetyl-α-D-glucosaminide (**13**) (30 mM) in 1:9 D_2O/H_2O at 298 K.

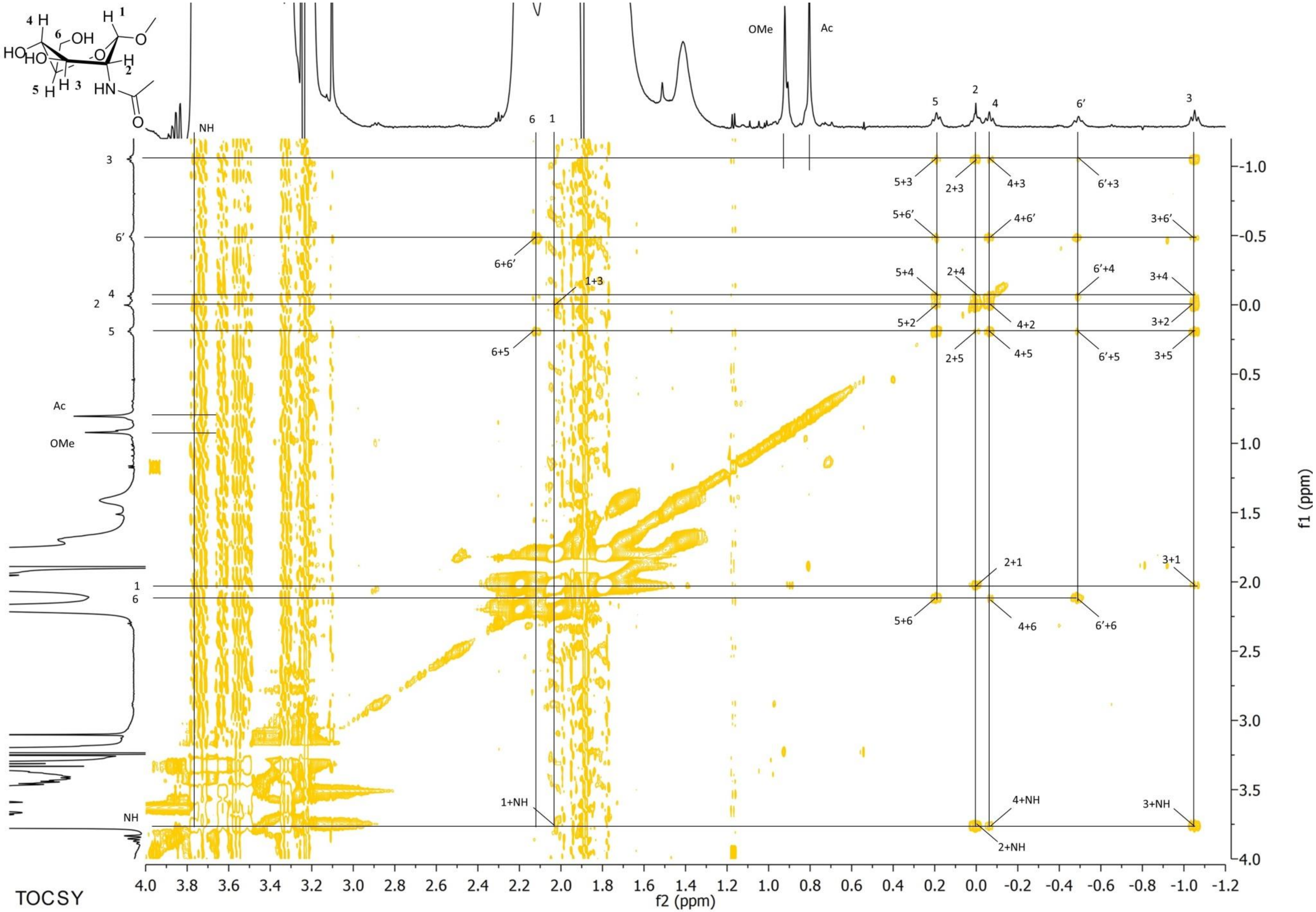

Figure S103. Partial ^{1}H TOCSY NMR spectrum of the staggered receptor **9** (0.60 mM) complexed with methyl *N*-acetyl-α-D-glucosaminide (**13**) (30 mM) in 1:9 D_2O/H_2O at 298 K.

Distances obtained from NOE data and subsequent molecular modeling

79 Distances	RMSD = 0.582 Å		
Receptor 9 + GlcNAc-α-OMe (13)			
Labels		Distance (A)	
f2	f1	nOe	model
6out	Ac'	4.037	4.985
Ac''	3out	4.453	5.411
NH1	1in	3.162	2.897
NH1	1out	2.904	2.300
NH1	OMe''	3.437	4.234
NH1	p10	3.042	2.587
NH1	s4a	2.541	3.137
NH1'	1in'	3.085	2.665
NH1'	1out'	3.113	2.963
NH1'	6'	3.745	3.591
NH1'	OMe''	4.185	4.028
NH1'	p10'	3.282	3.241
NH1'	s2a	2.473	2.402
NH1'	s6a	3.897	4.259
NH3	3in	2.785	2.572
NH3	3out	3.035	2.984
NH3	OMe'	3.181	2.461
NH3	p2	2.976	3.184
NH3	s2b	2.506	2.371
NH3'	3in'	2.983	2.838
NH3'	3out'	2.886	2.280
NH3'	p4'	3.055	2.567
NH3'	s2d	3.233	3.742
NH3'	s6d	2.554	2.991
NH6	3	3.637	3.822
NH6	4	3.914	4.065
NH6	6in	3.102	2.964
NH6	s2c	2.633	2.099
NH6'	2	3.215	4.453
NH6'	6in'	3.275	2.811
NH6'	6out'	3.073	2.878
NH6'	s2c	2.300	2.131
NH8	6	3.651	4.365
NH8	6'	4.088	3.804
NH8	s2d	2.483	2.260
NH8'	8in'	2.739	2.850
NH8'	8out'	2.947	2.277
NH8'	p7'	2.961	4.041
NH8'	p9'	3.581	3.031

Labels		Distance (A)	
f2	f1	nOe	model
NH8'	s6b	2.408	3.262
OMe''	1in	4.613	5.106
OMe''	1out	4.620	5.595
OMe'	3in	4.388	4.138
p10'	1out'	2.452	2.308
p10'	OMe''	3.931	4.060
p2	1in	2.689	2.282
p2	OMe'	3.475	3.082
p2'	3in'	2.459	2.342
p2'	6'	3.681	4.501
p2'	NH1'	4.062	3.510
p4	NH	3.544	4.523
p4'	3in'	2.693	3.603
p4'	p5'	2.550	2.422
p5'	6out'	2.362	2.452
p7'	2	4.164	5.949
p7'	6out'	2.778	3.448
p7'	8in'	2.566	2.408
p9	8out	2.316	2.198
p9'	8in'	2.609	3.525
p9'	OMe	3.952	3.750
s2a	1	4.164	4.456
s2a	6	4.715	3.851
s2a	6'	4.050	4.574
s2a	NH1	3.318	3.543
s2a	OMe''	2.862	2.462
s2b	1	3.112	4.080
s2b	Ac''	3.500	2.707
s2b	NH	3.222	2.483
s2b	OMe	3.000	3.168
s2c	3	3.996	3.540
s2c	Ac'	3.762	3.687
s2d	5	4.294	3.836
s2d	6'	3.197	2.596
s4a	NH1	2.541	3.137
s4b	Ac''	4.262	5.284
s4b	NH3	3.410	4.150
s4d	dNHd	2.545	2.326
s6b	8in'	3.689	4.071
s6b	Ac''	4.488	5.117

Images of Molecular Model of 9 + GlcNAc-*α*-OMe (13)

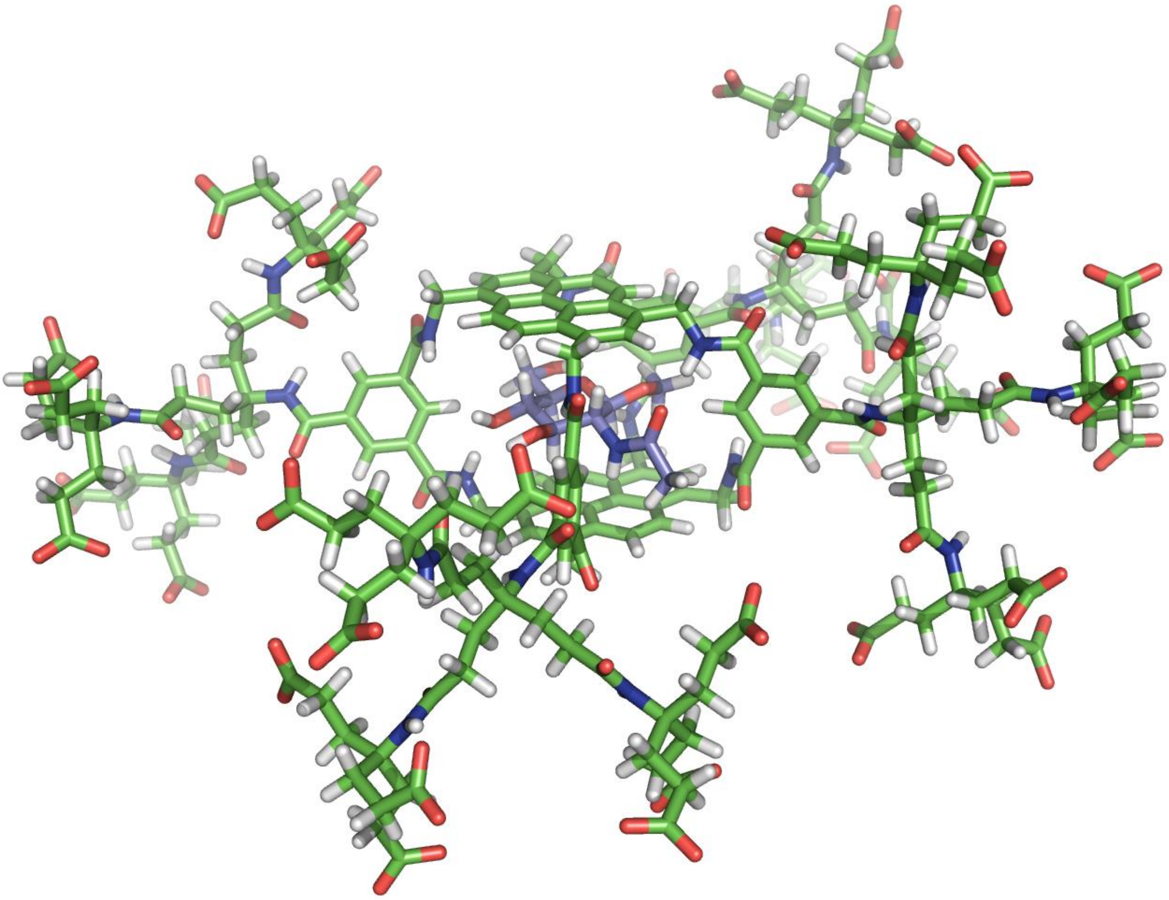

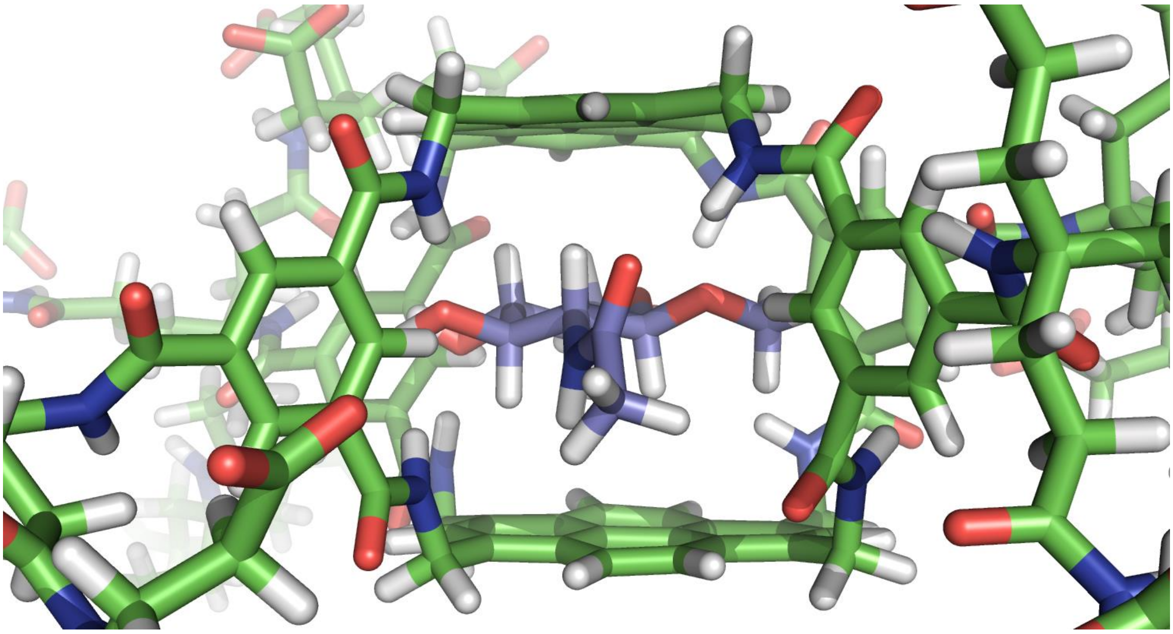

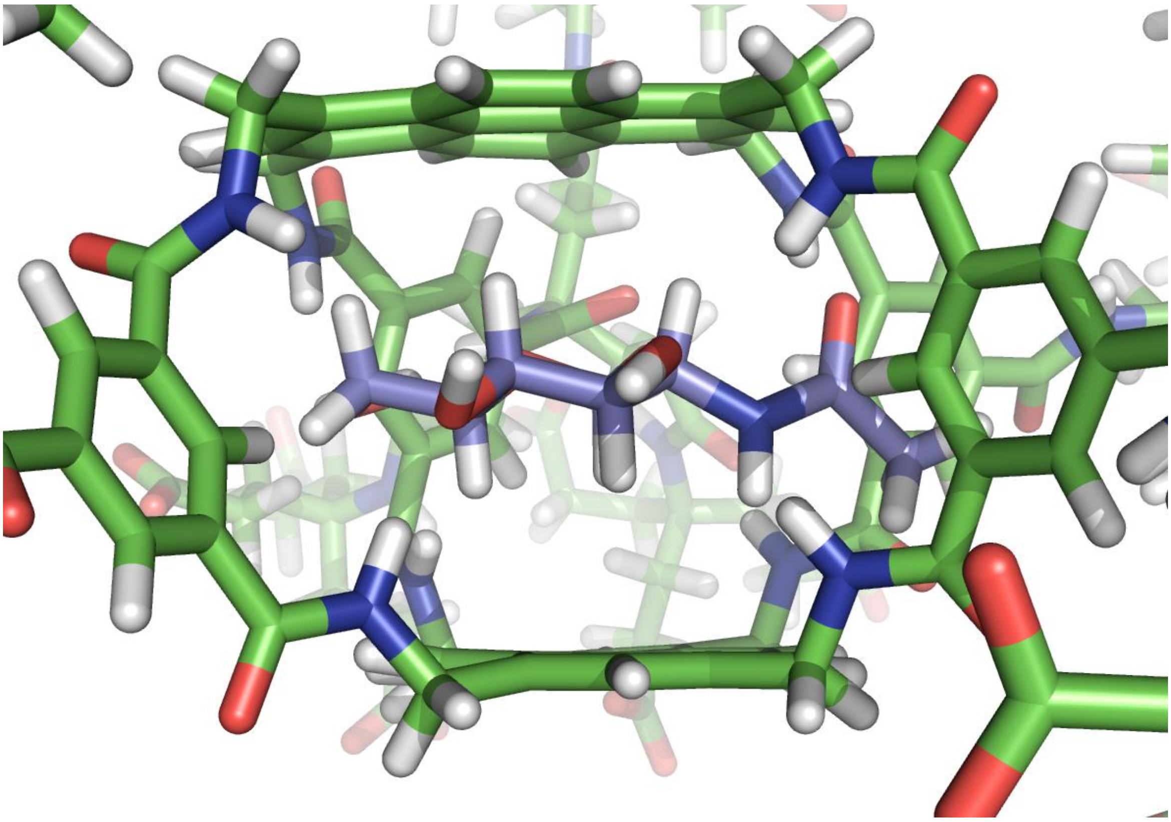

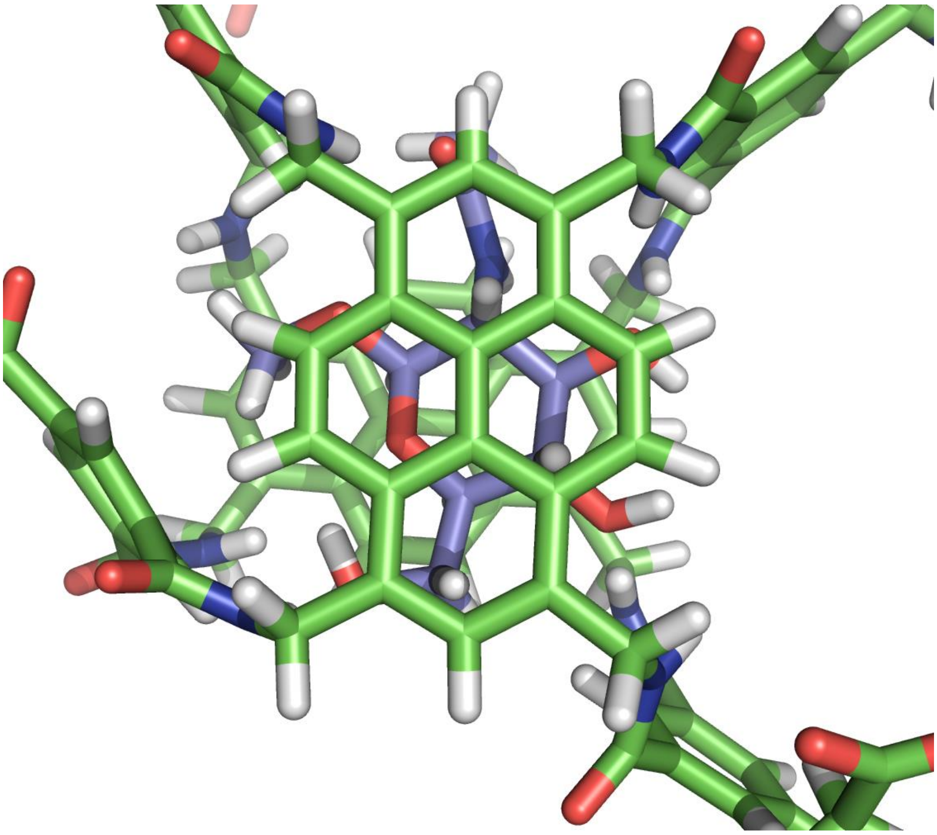

Supplement: Supplementary file 1 — Supplementary [file ANIE-55-3387-s001.pdf]
